# The Genome-Wide Early Temporal Response of *Saccharomyces cerevisiae* to Oxidative Stress Induced by Cumene Hydroperoxide

**DOI:** 10.1371/journal.pone.0074939

**Published:** 2013-09-20

**Authors:** Wei Sha, Ana M. Martins, Reinhard Laubenbacher, Pedro Mendes, Vladimir Shulaev

**Affiliations:** 1 Virginia Bioinformatics Institute, Virginia Tech, Blacksburg, Virginia, United States of America; 2 Bioinformatics Research Division, University of North Carolina at Charlotte, Kannapolis, North Carolina, United States of America; 3 Department of Applied Biology, University of Sharjah, Sharjah, United Arab Emirates; 4 Center for Quantitative Medicine, University of Connecticut Health Center, Farmington, Connecticut, United States of America; 5 School of Computer Science and Manchester Centre for Integrative Systems Biology, University of Manchester, Manchester, United Kingdom; 6 Department of Biological Sciences, College of Arts and Sciences, University of North Texas, Denton, Texas, United States of America; University of Cambridge, United Kingdom

## Abstract

Oxidative stress is a well-known biological process that occurs in all respiring cells and is involved in pathophysiological processes such as aging and apoptosis. Oxidative stress agents include peroxides such as hydrogen peroxide, cumene hydroperoxide, and linoleic acid hydroperoxide, the thiol oxidant diamide, and menadione, a generator of superoxide, amongst others. The present study analyzed the early temporal genome-wide transcriptional response of *Saccharomyces cerevisiae* to oxidative stress induced by the aromatic peroxide cumene hydroperoxide. The accurate dataset obtained, supported by the use of temporal controls, biological replicates and well controlled growth conditions, provided a detailed picture of the early dynamics of the process. We identified a set of genes previously not implicated in the oxidative stress response, including several transcriptional regulators showing a fast transient response, suggesting a coordinated process in the transcriptional reprogramming. We discuss the role of the glutathione, thioredoxin and reactive oxygen species-removing systems, the proteasome and the pentose phosphate pathway. A data-driven clustering of the expression patterns identified one specific cluster that mostly consisted of genes known to be regulated by the Yap1p and Skn7p transcription factors, emphasizing their mediator role in the transcriptional response to oxidants. Comparison of our results with data reported for hydrogen peroxide identified 664 genes that specifically respond to cumene hydroperoxide, suggesting distinct transcriptional responses to these two peroxides. Genes up-regulated only by cumene hydroperoxide are mainly related to the cell membrane and cell wall, and proteolysis process, while those down-regulated only by this aromatic peroxide are involved in mitochondrial function.

## Introduction

Several processes expose cells to reactive oxygen species (ROS) that cause severe damage to proteins, DNA and lipids, impairing cell function. Mitochondrial respiration is a major contributor to oxidative stress, generating ROS as side products, but these are also generated by other processes. Oxidative stress is also a mechanism used by immune cells to fight pathogens, a weapon that properly directed is beneficial to the host, but that can easily cause damage to other host cells as well. ROS have been recognized as important pathophysiological agents, being involved in the processes of aging [1] and apoptosis [2], and human diseases [3] like Alzheimer’s [4], cancer [5] and diabetes [6].

Given the ubiquitous presence of low levels of ROS in every respiring cell and the exposure to higher levels of ROS in many other situations, cells have evolved several protective mechanisms against oxidative stress. Superoxide dismutases (Sod1p, Sod2p) and catalases (Cta1p, Ctt1p) directly transform some ROS into compounds of lower toxicity. Peroxidases promote the reduction of ROS through the oxidation of important antioxidant metabolites: reduced glutathione (GSH), thioredoxin, and ascorbic acid. Secondary plant metabolites such as tocopherols, carotenoids and flavonoids are also strong antioxidants and can reduce ROS directly. In *Saccharomyces cerevisiae*, the major eukaryotic model for studies of oxidative stress response (OSR), ascorbic acid seems to be absent [7] and the major antioxidant is GSH (even though D-erythroascorbic acid is present [8] and could have a role similar to that of L-ascorbic acid, its action in oxidative stress resistance is limited [9]). This is similar to mammals where ascorbic acid exists in much lower concentrations than GSH, the latter being the major antioxidant metabolite.

The OSR is tightly regulated at the transcriptional level. Several transcription factors have been described to be involved in this response, and perhaps the best known in yeast isYap1p, which plays a central role in the regulation of oxidative stress-responding genes in *S. cerevisiae* [10]. It belongs to a family of eight basic leucine zipper proteins that are believed to be involved in transcriptional regulation [11]. The action of Yap1p has been reported to occur at the post-translational level through regulated nuclear export: the oxidized form of Yap1p is unable to exit the nucleus where its levels increase causing increased binding to the promoter region of target genes [12]. The pathway upstream of Yap1p that transduces the oxidant signal includes Gpx3p and Ybp1p, although their order is not yet clear [10]. Yap1p is reduced by the thioredoxin Trx2p, whose gene is also induced by Yap1p, forming a negative feedback loop [13]. Skn7p is a transcription factor that is also involved in the response to oxidative stress [14,15]. Skn7p was first described as being part of a two-component signal pathway in response to osmotic stress [16,17]. The involvement in the response to oxidative stress proceeds through a different mechanism and involves different DNA sequence elements in the target genes [18]. Additionally, a large number of genes induced under adverse environmental conditions, such as nutrient starvation, entry into stationary phase and several types of stresses (oxidative, heat, salt, etc) are under the control of the transcription factors Msn2p and Msn4p, and have been termed the “common environmental stress response genes” [19]

The genome-wide temporal transcriptional yeast OSR has been described in previous studies [19,20,21,22,23,24,25,26,27] and much has been learned from them. In addition to global gene expression analyses, a considerable body of knowledge about the transcriptional OSR has been obtained through traditional biochemical and molecular biology methods (e.g. [28,29,30]), chromatin immunoprecipitation-DNA microarray (ChIP-chip) assays [31,32], proteomics [33] and bioinformatics [34,35,36]. Regarding previous genome-wide transcriptional response studies, we note that some technical issues complicate interpretation of their results: some do not include time-dependent controls [19,20,21] (and in one case [19] not even biological replicates); many were carried out in conditions where oxygen and other important environmental factors are not controlled, e.g. by growth in shaker flasks [19,20,22,23,24,25,26,27]. Despite this, they have already shown how important it is to carry out temporal transcriptome analysis after oxidative stress perturbations to reveal its complex transcriptional regulation. Most studies included a minimum of 10 min incubation with the oxidative agent before gene expression profiles were measured, but Lucau-Danila et al. [25] recorded mRNA levels starting from 30 second after addition of the drug benomyl. This revealed that the transcriptional response to benomyl is fast, with the levels of some transcripts already significantly altered at that time.

Different ROS cause distinct transcriptional responses [37]. A systematic screen of the yeast deletion strains has shown that specific genes are essential to provide constitutive protection against oxidative stress caused by H_2_O_2_, linoleic acid 13-hydroperoxide, diamide, menadione, and CHP [38]. Among the 5,000 mutants screened only 2 were sensitive to all 5 oxidants and 12 to at least 4 out of the 5 [38]. Previous studies of temporal transcriptional OSR focused on endogenous ROS such as H_2_O_2_ [19,20,22] and lipid hydroperoxides [23], and environmental stressors like arsenic [24], or drugs like benomyl [25].

A critical step in the degradation of lignin by fungi is peroxidation releasing intermediate phenolic peroxides such as CHP [39]. While *S. cerevisiae* does not degrade lignin, there is evidence that it is capable of growing in ligninolytic environments [40,41,42], perhaps as a relic of its evolutionary past. Thus CHP represents a class of phenolic peroxides that are physiologically relevant to many fungi causing a unique oxidative stress transcriptional response [38]. Here we study the dynamics of the transcriptional response of *S. cerevisiae* to oxidative stress induced by CHP. Experiments were carried out in triplicates, with cultures in mid-exponential growth phase growing at constant temperature, pH and aeration. Appropriate controls consisted of cultures to which no CHP was added and their gene expression was also monitored in triplicates along time, side by side with the CHP-treated cultures. Our analysis reveals early transcriptional events induced by CHP and identifies a set of genes previously not implicated in the OSR.

## Materials and Methods

### Yeast strain

The *Saccharomyces cerevisiae* strain used in this work was *BY4743* (*[4741/4742*]* MAT**a**/MATα his3Δ1/his3Δ1 leu2Δ0/leu2Δ0 lys2Δ0/+ met15Δ0/+ ura3Δ0/ura3Δ0*). This strain, purchased from American Type Culture Collection (ATCC #201390), was constructed by the yeast deletion consortium [43,44] and is a derived from the S288C strain. Cultures were kept in long-term storage frozen at -80°C in glycerol stocks. Work cultures were kept at 4°C in YPS (yeast extract 0.1% (w/v), peptone 0.5% (w/v) and sucrose 2% (w/v)) agar plates.

### Cell growth and oxidative stress conditions

An initial culture was batch grown overnight at 30°C, 150 rpm, in minimal medium with 2% (w/v) sucrose (MMS; yeast nitrogen base without amino acids, Difco + 2% (w/v) sucrose) supplemented with uracil 20 mg/l, L-leucine 60 mg/l and L-histidine 20 mg/l. This culture was used to inoculate fermentors (1 l capacity, New Brunswick BioFlo) containing MMS 4% (w/v) supplemented with uracil 40 mg/l, L-leucine 120 mg/l and L-histidine 40 mg/l, to an initial OD_600_ of 0.3. Cultures were grown at 30°C, pH 6.0 and dO_2_ > 80%, to mid-exponential phase (OD_600_ ~ 1.5). Oxidative stress was applied by adding a solution of CHP in 95% (w/v) ethanol (with a concentration previously determined by HPLC) to 3 of the fermentors to obtain a final concentration of 190 µM. Controls without CHP were made by adding the same volume of 95% (v/v) ethanol (the solvent for CHP) to the other 3 fermentors.

### Sample collection and processing

Samples were collected immediately before the addition of CHP (or ethanol) and at 3, 6, 12, 20, 40, 70 and 120 min thereafter. Samples (60 ml of culture) were collected directly from the fermentors, in a tricine-buffered methanol solution, kept at -40°C using a dry ice-ethanol bath, as described [45].

Samples were centrifuged for 3 min at 1000× *g* and -10°C. Temperature was monitored after centrifugation to ensure that it was below -35°C. Supernatant (media in buffered methanol solution) was stored at -20°C and the pellets were washed with buffered methanol, freeze-dried for 48 h using a Labconco Freeze Dry System and stored at -80°C until use. This sample collection procedure allowed for the preservation of nucleic acids, proteins and metabolites. The same samples are now being analyzed for their protein and metabolite profiles, which will be the object of a future publication.

### RNA extraction

RNA was extracted with a procedure modified from the hot phenol protocol [46], as described earlier [45]. The quality of the RNA obtained was evaluated by UV-spectroscopy and by capillary electrophoresis in an Agilent 2100 Bioanalyzer lab-on-a-chip system.

### Probe preparation, microarray hybridization and data acquisition

For transcript profiling we used the Affymetrix GeneChip^®^ system with the Yeast Genome S98 arrays (Affymetrix, Santa Clara, CA). RNA samples that passed a quality control check were amplified using the GeneChip^®^ One-Cycle cDNA synthesis kit, as recommended by the manufacturer. Hybridization of labeled targets was performed against S98 arrays following the manufacturer’s protocols. All arrays passed the manufacturer’s standard quality metrics for hybridization, staining and overall chip performance.

### Determination of CHP and cumyl alcohol (COH) concentrations in samples

The concentration of the solution of CHP to be applied to the cultures was determined by HPLC with a Photodiode Array Detector. The concentrations of both CHP and its product, COH, were also determined in the medium/methanol samples collected after the centrifugation of the cultures. Samples were analyzed in a Shimadzu HPLC system, using a Prevail C18 column (150 x 4.6 mm). The solvent was 35:65 acetonitrile: phosphate buffer (5 mM, pH 7.0). Detection was made at 202 nm, using a Surveyor Photodiode Array Detector from Thermo Finnigan.

### Data analysis

Robust Multichip Average (RMA) [47,48] was used for microarray data summarization and normalization of all 48 arrays simultaneously. To assess the significance of differences between transcripts across two time points, we used 2-way ANOVA gene-by-gene model (using SAS version 9, SAS Institute Inc., Cary, NC, USA):

$$y_{ijk}=\mu +T_{i}+V_{j}+{\left(TV\right)}_{ij}+\varepsilon_{ijk}$$

where *y*_*i,j*_,_*k*_ is the intensity measured on the array for time *i* (in this case, *i* = 0, 1, 2, ..., 7), treatment *j* (in this case, treatment is control or CHP) and replicate *k*; μ is the overall mean intensity of this gene across all samples; *T*_*i*_ is the effect of the *i* ^th^ time; *V*_*j*_ is the effect of the *j* ^th^ treatment; (*TV*)_*i,j*_ is the interaction effect between time *i* and treatment *j*; *ε*_*i,j*_,_*k*_ is the residual for time *i*, treatment *j*, replicate *k*.

The positive False Discovery Rate (pFDR, cutoff 0.05) multiple-testing adjustment [49] was applied to correct *p*-values. Coefficients of variation (CV) among each set of three replicates were calculated as a measure of reproducibility. Results of the *p*FDR-corrected ANOVA results are available through a database system (DOME) that allows querying different types of comparisons through a simple web interface at the URL http://calvin.vbi.vt.edu/DOME/DOMESC/.

Genes with similar expression pattern were grouped by *k*-means clustering, using the TIGR (The Institute for Genomic Research) Multiexperiment Viewer version 3.0.1 [50].

To reveal pathways that were significantly affected by the oxidative stress, data was processed with the Database for Annotation, Visualization and Integrated Discovery (DAVID, version 2) [51] with *p*<0.01. To reveal which gene ontology (GO) [52] categories were significantly affected by the oxidative stress, we used the GoMiner software [49], however we only considered the categories that are part of GO Slim [53]; GO Slim categories with *p*-value adjusted to FDR<0.01 in the GoMiner result were selected and are shown in the heat map.

Heat maps were made with the TreeView software [54] and display the logarithm of the ratio of median value of a time point divided by the median value of the same gene for time zero.

Lists of genes documented to be under control of several transcription factors were obtained from the Yeast Search for Transcriptional Regulators And Consensus Tracking (YEASTRACT) database [36].

Microarray raw data (cel files) were deposited in the National Center for Biotechnology Information Gene Expression Omnibus (GEO) with the accession number GSE7645 (http://www.ncbi.nlm.nih.gov/geo/query/acc.cgi?acc=GSE7645).

## Results and Discussion

### Physiological response

**Figure 1:**
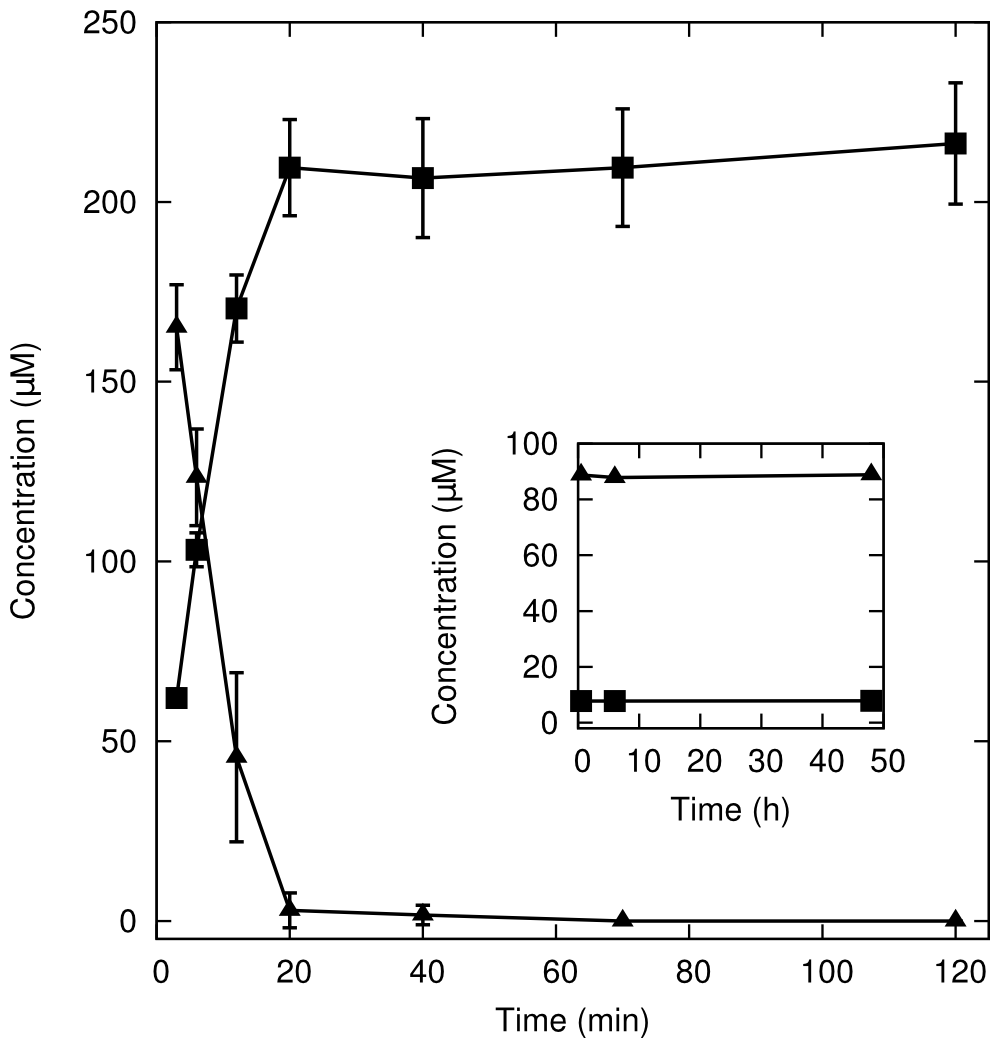
Time course of oxidant and its product. Time-dependent concentration of CHP (triangles) and its product, COH (squares) in cell culture medium after the addition of CHP (*t* = 0). Results plotted are median with standard deviation as error bars of biological triplicates. The inset shows the stability of CHP and COH in culture medium without yeast cells.

Part of the yeast physiological response to oxidative stress is to transform the oxidant to a less harmful product. In the case of CHP, the two-electron reduction product is cumyl alcohol (COH). Both CHP and COH can be separated and quantified by HPLC [55], allowing direct measure of changes in their concentrations. We observed that exposure of exponentially grown yeast cultures to CHP resulted in a rapid conversion of this compound to COH, with most of the CHP gone within 20 min (Figure 1). This result provides a time scale for the physiological response that happens downstream from signaling events and resulting transcriptional response.

### Overview of the transcriptional response

**Figure 2:**
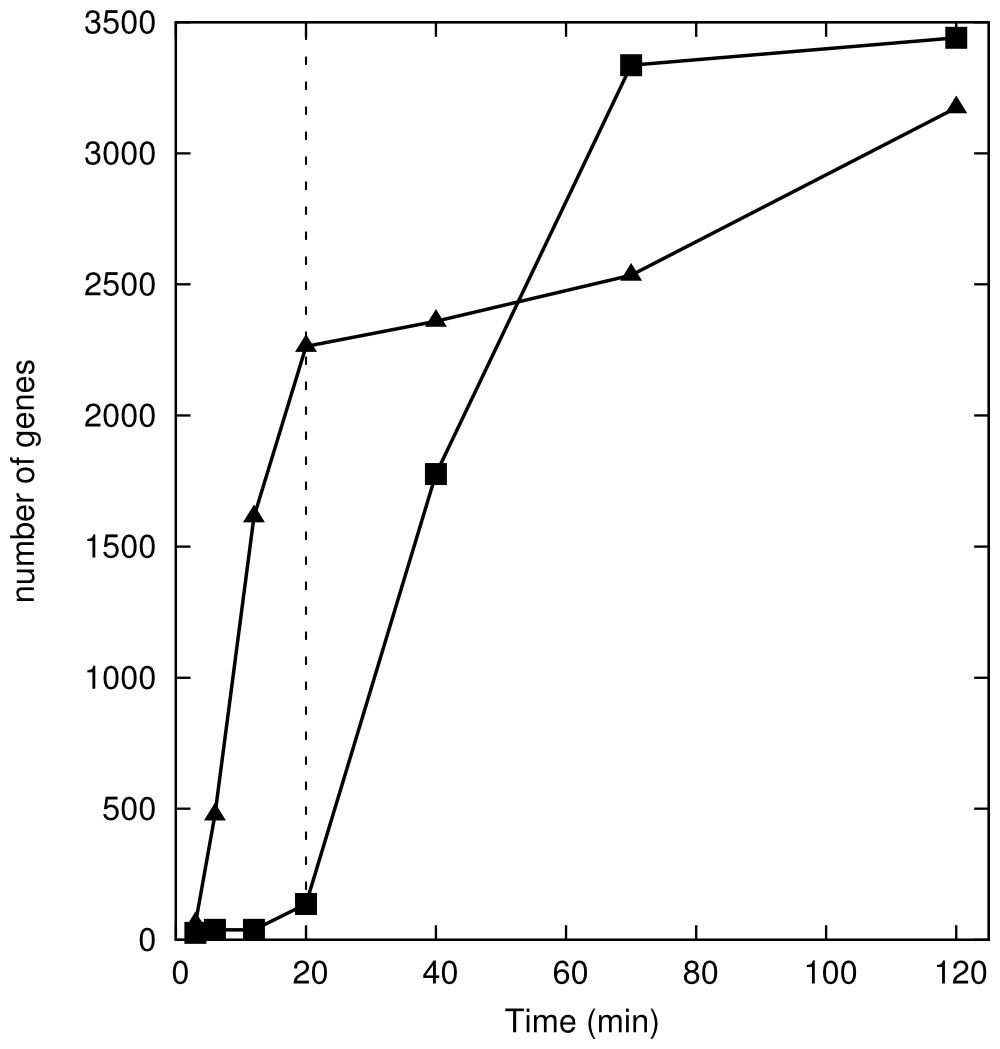
Summary of gene expression changes. Number of genes that have changed expression level compared with the control at *t* = 0 in control (squares) and CHP-treated cultures (triangles). Note that after *t* = 20 min the controls indicate that something drastic is happening in the cultures. Only data from samples to the left of the vertical line (*t* ≤ 20 min) were used in subsequent analysis.

Analysis of gene expression data shows a large number of transcripts significantly changed (positive false discovery rate, pFDR<0.05) in the control cultures starting 40 min post-CHP treatment (Figure 2). The role of these temporal controls is to detect possible artifactual changes that are caused by factors unrelated to oxidative stress. As so many genes changed in these cultures that were unexposed to CHP, we conclude that the last three time points are unreliable for the analysis of the OSR and therefore have not considered them for that purpose. Hence, and since the physiological response happens earlier than 40 min, as seen in the previous section, we proceed with the analysis of the results up to the 20 min time point for purposes of dissecting the transcriptional response to CHP. The complete set of data including the late time points has, nevertheless, been submitted to the GEO (Gene Expression Omnibus) database.

A number of previously reported genome-wide temporal OSR studies [19,20,21] have not included controls. Instead, by comparing all changes to the time zero expression levels (i.e. before addition of oxidant) they assume that no genes change in the absence of the oxidative stress agent. However, taking into account the results reported here, caution should be used in interpreting results obtained without proper controls. Another feature that is extremely important in quantitative studies is to carry out a number of biological replicates in order to be able to assess reproducibility of the procedures (both biological and technical) [56]. In this study we used 3 biological replicates for all cultures.

**Figure 3:**
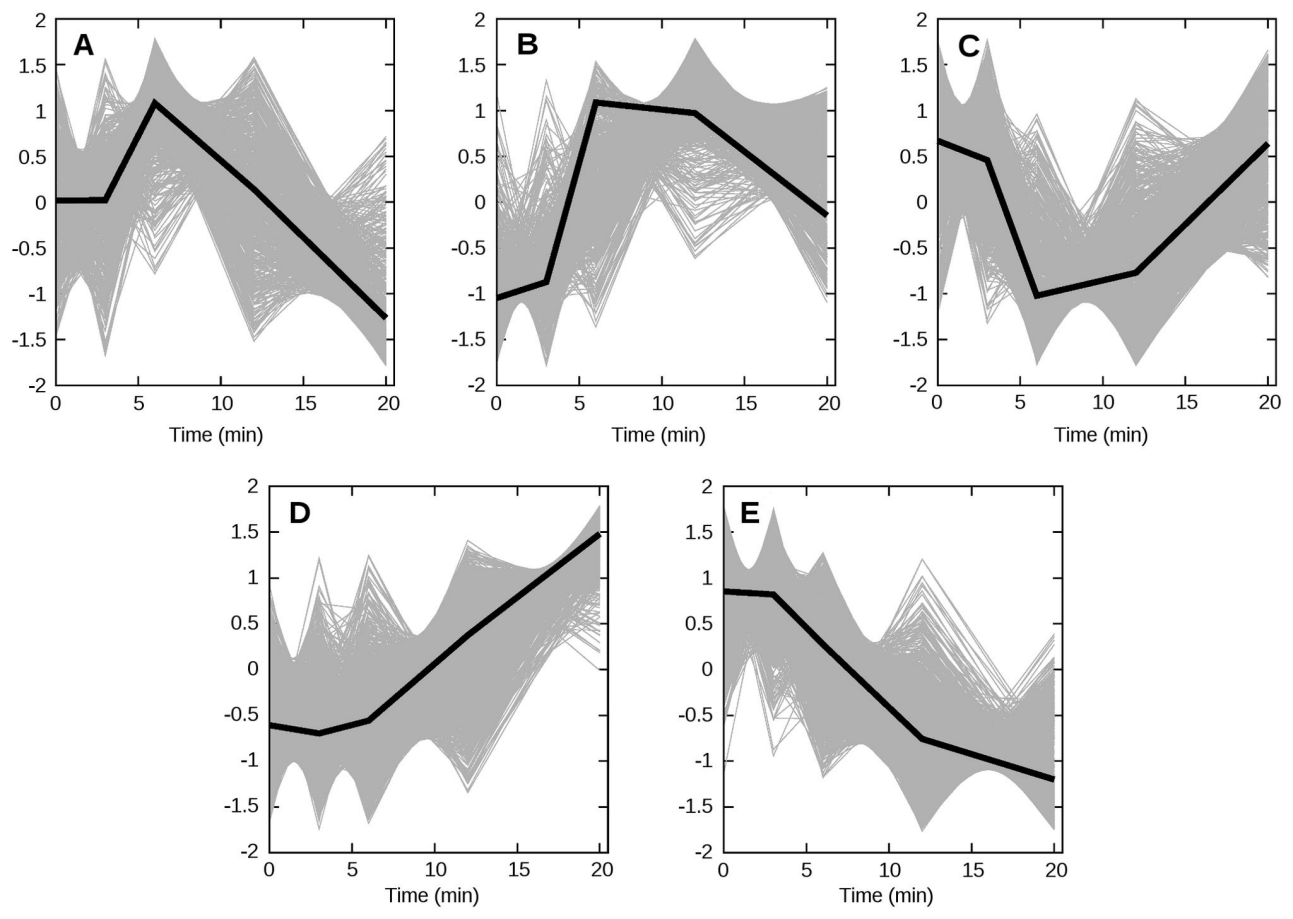
Overall gene expression patterns. Gene expression data was clustered with the *k*-means algorithm using the MeV software [50]. Each gene is represented by a thin gray line, while the median centroids of clusters are represented by thick black lines. Expression pattern for each gene is plotted in a scale of unit standard deviation. Complete lists of genes in each cluster are available in Table S1.

To summarize the overall gene expression response we clustered the data to identify the major patterns of change. We determined the best number of clusters by visually observing the results of *k*-means clustering, and concluded that 5 clusters provide the best summary of global changes in gene expression. The dynamics of these 5 clusters after exposure to CHP are depicted in Figure 3. Cluster A contains 570 genes transiently up-regulated, with a peak at 6 min, while cluster B contains 723 transiently up-regulated genes but with a later peak, at 12 min. The 777 genes included in cluster C show a pattern of transient down-regulation with a minimum between 6 and 12 min. Cluster D, with 1732 genes, displays a slower up-regulation, while cluster E (1850 genes) is its mirror image with a slow down-regulation. Since the clustering process does not take into account any biological knowledge, an interesting question is whether any of these clusters may represent well-defined functions. We analyzed the cluster compositions for pathways that may be significantly over-represented (*p*<0.01) in each cluster and the results are summarized in Table 1. Cluster D, which shows a pattern of up-regulation starting at 20 min, includes the proteasome, ubiquitin-mediated proteolysis and the mitogen-activated protein kinase (MAPK) pathway, i.e., the machinery needed for cellular remodeling: the targeted decomposition of cellular proteins needed to switch from growth to defense against oxidative stress. Cluster E includes the ribosome, cell cycle, RNA polymerase, purine and pyrimidine biosynthetic pathways – clearly all of these are involved in production of proteins and nucleic acids essential for cell division in a growing culture. Down-regulation of cluster E is in agreement with the arrest of growth of the culture when challenged with an oxidant (time course plots of these genes are supplied in Figures S1, S2 and S3). These data clearly show a global response of growth-arrest and protein degradation, which is likely to remodel the functionality of these cells. Cluster B also has two pathways significantly changed: galactose, and starch and sucrose metabolism. This is probably related with increase of trehalose production (trehalose metabolism is not a specific pathway in KEGG (Kyoto Encyclopedia of Genes and Genomes) but rather a part of the “starch and sucrose metabolism” map), a fact supported by the up-regulation of several genes involved in trehalose metabolism.

**Table 1:** Pathway analysis of gene expression clusters.

**Cluster**	**Pathway**	***P*-value**
A	Oxidative phosphorylation	0.0025
B	Galactose metabolism	0.0042
	Starch and sucrose metabolism	0.0062
C	ATP synthesis	9.1 x 10^-4^
D	Proteasome	1.2 x 10^-16^
	Ubiquitin-mediated proteolysis	0.0059
	MAPK signaling	0.0081
E	Ribosome	1.8 x 10^-14^
	Cell cycle	1.3 x 10^-5^
	RNA polymerase	6.1 x 10^-5^
	Purine metabolism	1.2 x 10^-4^
	Pyrimidine metabolism	6.3 x 10^-4^

Cluster gene membership lists were processed with the DAVID resource [51] and KEGG pathways with *p*<0.01 are listed (corrected for multiple comparison, as described in Methods).

### Early transient response

**Figure 4:**
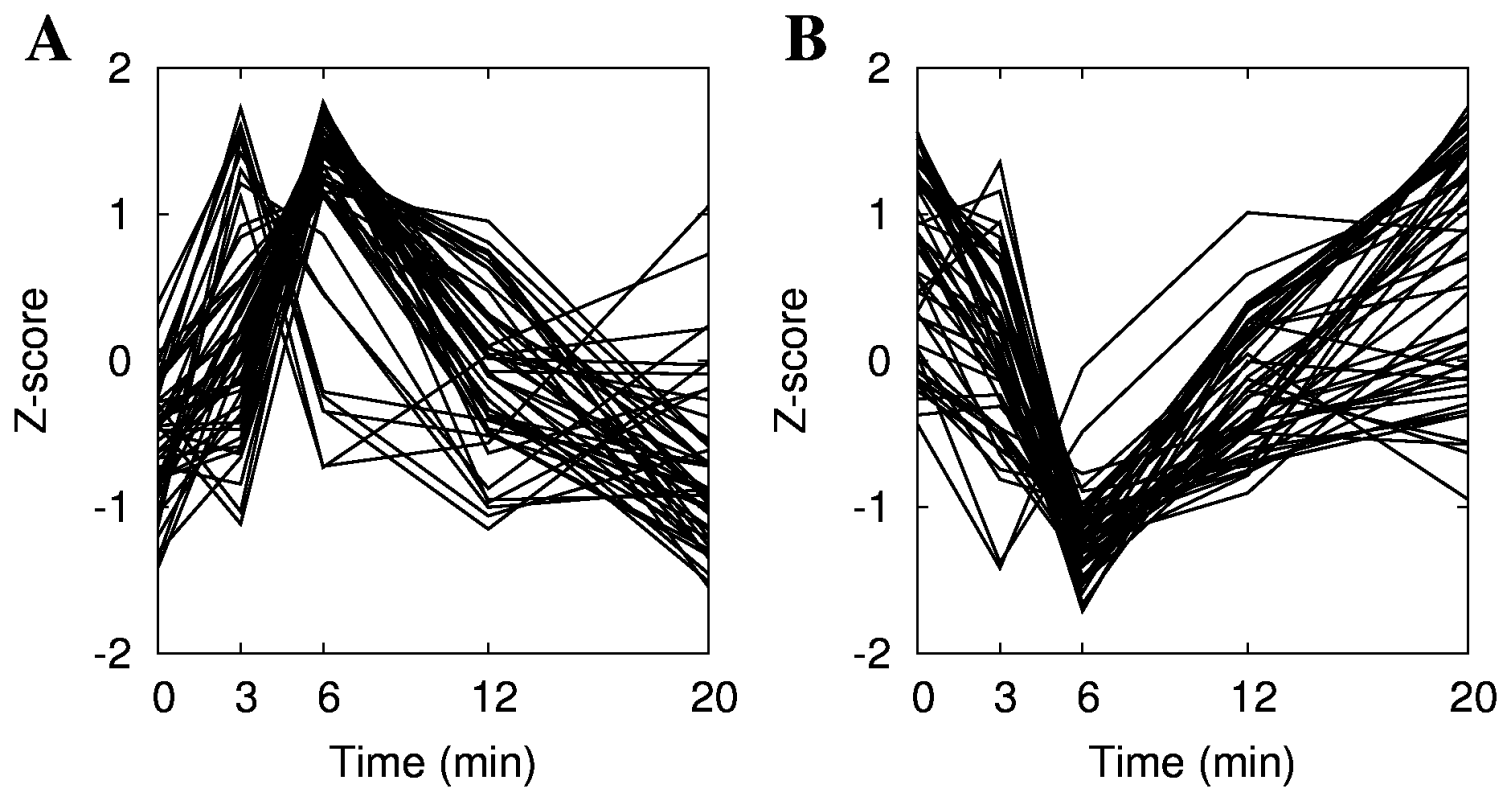
Rapid response genes. **A**: expression pattern of genes that are induced rapidly and which return to their normal (pre-perturbation) mRNA levels at 12 min post-perturbation. **B**: expression pattern of genes that are repressed rapidly, also returning to their normal mRNA levels at 12 min. A list of genes is included in Table S2. The ordinate is in a scale of unit standard deviation.

Previous microarray studies of OSR in yeast identified a set of genes responding to oxidative stress 10 min and later after treatment [19,20,21]. However, gene expression changes can be detected as early as 30 sec after the stress treatment, as shown in the study of the early gene expression induced by benomyl [25]. Our time series started at 3 min after the addition of CHP which allowed us to identify a set of the early oxidative stress responsive genes that are only transiently induced or repressed at 3 and/or 6 min after the oxidant addition (Figure 4 and Table S3) and therefore would have been undetectable in prior stress response studies.

We have identified 44 genes that were significantly induced (*p*<0.05) within 6 min of CHP exposure but which quickly returned to their original mRNA levels (Table S2-A). These genes encode transcription factors, stress response or drug resistance-related proteins, proteins involved in cell wall and actin cytoskeleton metabolism, and others, including 16 genes of unknown function. We have also identified 51 genes that were transiently repressed (*p*<0.05) in the same time frame (Table S2-B), including genes involved in DNA replication, cell growth and division, transcription, translation, mitochondrial function, and vesicle trafficking.

**Figure 5:**
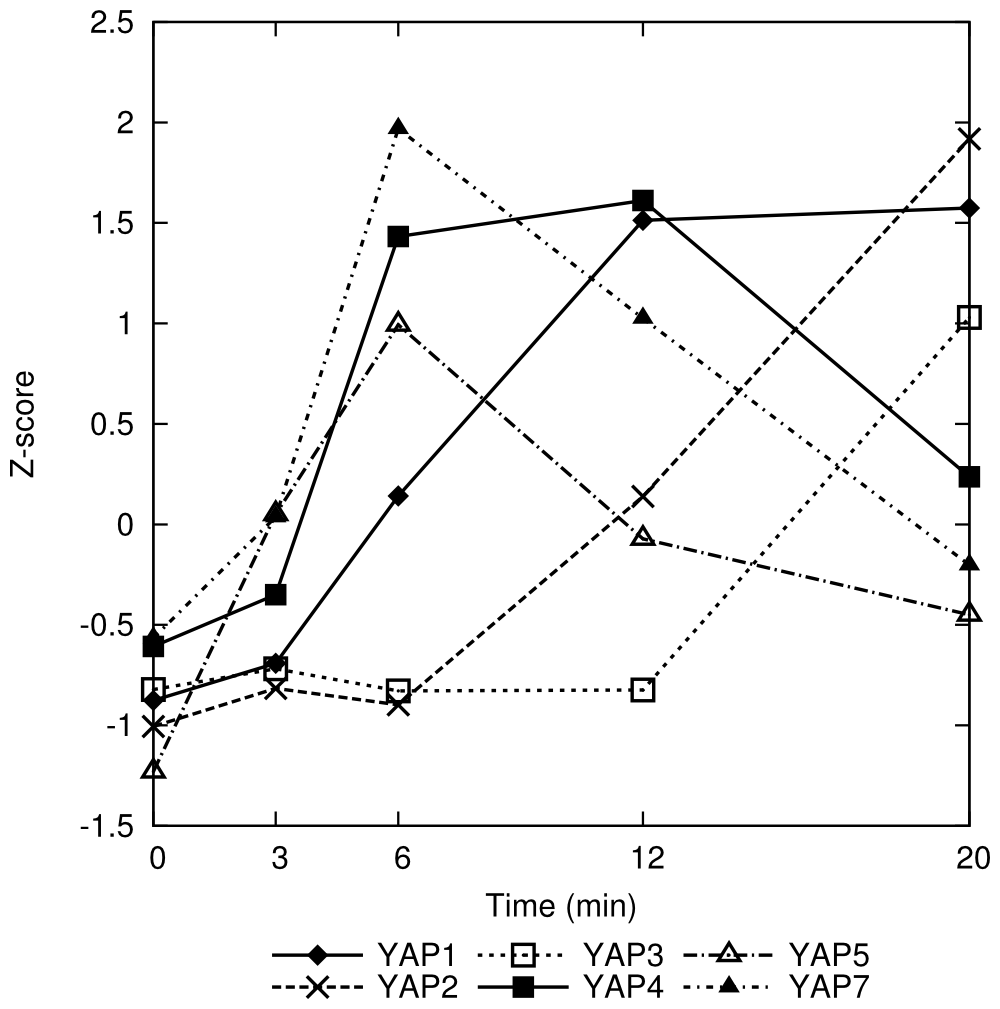
Dynamics of expression of the genes encoding proteins from the yeast YAP family in response to cumene hydroperoxide. The members of the YAP family are bZIP proteins [11] with transcription factor activity. The ordinate is in a scale of unit standard deviation. *YAP3*, *YAP5*, and *YAP7* are for the first time observed here to be induced by oxidative stress.

Many of the early up-regulated genes encode transcription factors: *HMS2*, *MET28*, *YAP5*, *NUT2*, *ROX1*, and *SUT2*. For several of these we could also observe induction of other genes that are known to be their targets. *MET28* regulates sulfur metabolism [57] and its targets *MET1*, *MET12*, *MET16*, *MET22*, *MET3*, *MET8*, *CYS3* and *STR3* were also significantly induced during the 20 min period of observation. The induction of sulfur metabolism is easily understood in the context of oxidative stress, since cysteine is component of molecules such as glutathione, glutaredoxin, thioredoxin and Yap1p, which were all induced in response to oxidative stress. *YAP5*, which had previously not been implicated in the OSR, was also transiently induced by the CHP at 6 min after the oxidant addition (Figure 5). Results from ChIP-chip experiments indicated that this gene may be regulated by Met28p [31,32], and *MET28* is an early up-regulated gene in our study. The precise role of Yap5p in the OSR remains unclear. Several genes involved in cell wall and cytoskeleton metabolism are also transiently induced by CHP. This may be related to repair processes since the primary damage caused by CHP occurs at the level of cell boundary [58].

This early fast transcriptional response to oxidative stress had not been observed before. It has been missed by many earlier experiments, particularly ChIP-chip which requires an incubation time of 15 minutes or more. The set of early transiently regulated genes identified in our study is extremely interesting as it is rich in transcription factors, pointing to a complex transcriptional regulation cascade. Fast transcriptional response indicates rapid remodeling of cellular processes to adapt to changing environment and stress conditions and it involves fast shut-down of processes related to cell growth and activation of the stress adaptation mechanisms.

### Stress response regulons

**Figure 6:**
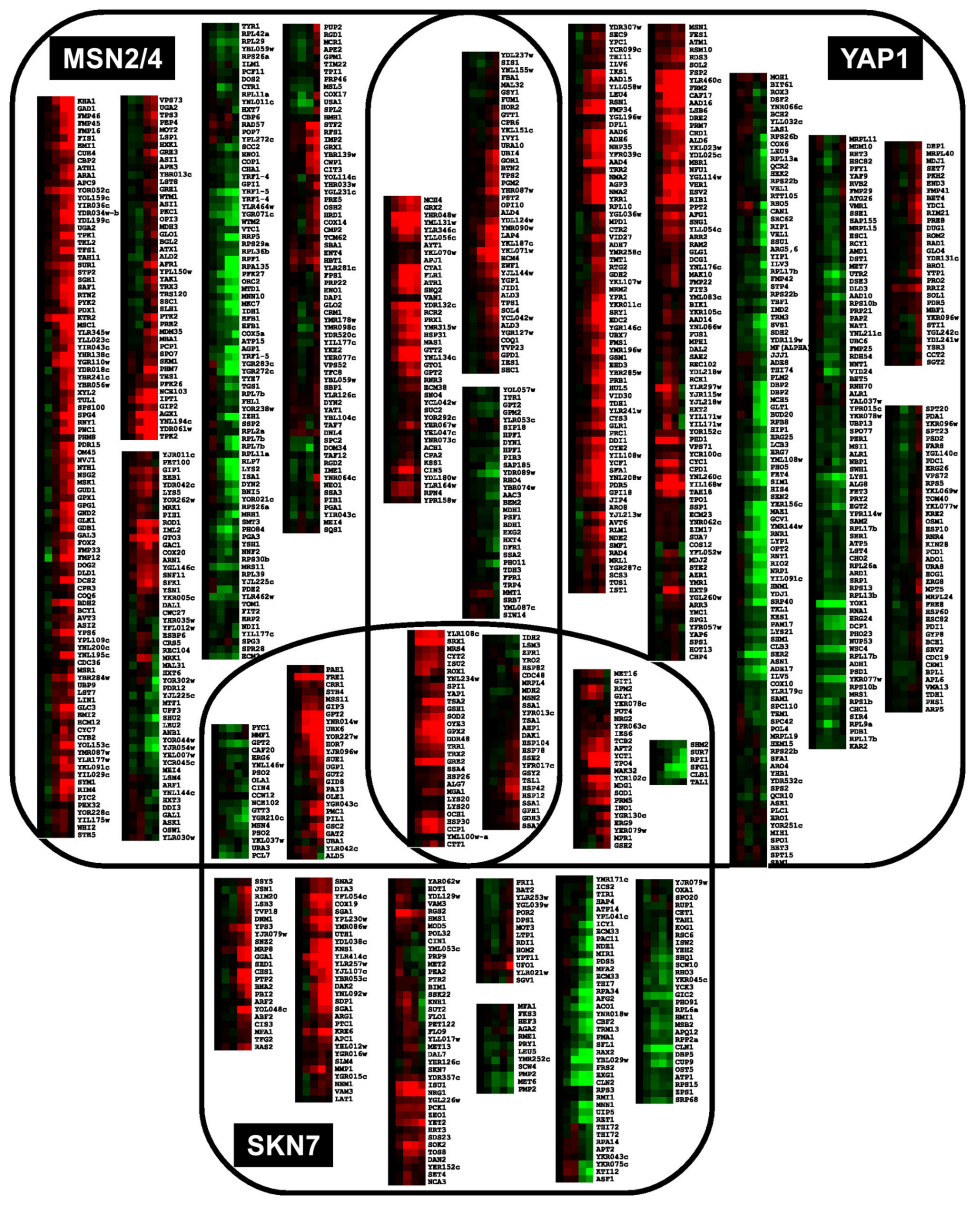
Dynamics of the transcriptional response of the *YAP1*, *SKN7*, and *MSN2/4* regulons. Expression profiles of genes known to be part of the three regulons (according to the Yeastract database [36]) associated with the oxidative stress response. The heat maps represent the five time points analyzed in their temporal sequence. Red represents expression levels above those of time zero, green represents levels below those of time zero; the intensity is proportional to the log of the ratio of median values divided by median value of time zero.

The transcriptional response to oxidative stress in yeast is known to depend on several transcription factors, including *YAP1* and *SKN7* that are thought to control independent, but also overlapping responses [33]; *MSN2* and *MSN4* mediate a transcriptional response which is common to many stresses including oxidative. Each of these regulons is composed of several hundred genes with a considerable overlap between them. In the present study a large proportion of these genes have indeed responded to CHP elicitation at different times, as depicted in Figure 6.

#### MSN2/4

Currently 601 genes are described to be controlled by Msn2p/Msn4p, and in this study 323 of them (54%) displayed statistically significant changes after addition of CHP. The actual *MSN2* and *MSN4* genes do not show any significant expression changes after CHP treatment, indicating that they are not regulated at the transcriptional level. Previous studies of the transcriptional response to H_2_O_2_-induced stress are contradictory: Causton and co-workers reported *MSN2* mRNA levels to be down-regulated at 10 min while they saw no change in *MSN4* [20]; data from Gasch et al. does not show any response of *MSN2* or *MSN4* [19]. Data from the present study with CHP agrees with the observations of Gasch et al. for H_2_O_2_ and strongly suggests that there is no response of *MSN2* and *MSN4* at the transcriptional level to oxidative stresses in general.

#### YAP1

In our experiments, *YAP1* mRNA levels were up-regulated between 6 and 20 min after addition of CHP. This result does not invalidate the hypothesis of Yap1p action by cellular localization, but suggests that its action may also involve regulation at the transcriptional level. This is supported by the finding that mutants exhibiting a constitutive nuclear localization of Yap1 do not show increased resistance to H_2_O_2_ [12]. As referred, upon oxidation, Yap1p accumulates in the nucleus leading to a rapid activation of target genes. Given that its mRNA also accumulates rapidly (6 min in our study) the total amount of Yap1p is likely to increase in a second phase. It is plausible that this transcriptional response of *YAP1* be caused by auto-induction since there are Yap1p-binding motifs (5'-TTAC/GTAA-3') upstream of the *YAP1* gene itself [11]. Of the 678 genes described to be affected by Yap1p, we report 352 (52%) that have been significantly changed by the action of CHP.

Other genes from the YAP family (*YAP2*, *YAP3*, *YAP4*, *YAP5*, and *YAP7*) were also significantly induced by CHP (Figure 5). *YAP2* and *YAP4* had been previously reported to be induced under oxidative stress [59,60] and in the case of *YAP4* also under osmotic stress [60]. *YAP5* and *YAP7* show a very early induction, while *YAP3* is slower. Interestingly, *YAP3*, *YAP5* and *YAP7* had not yet been associated with oxidative or any other stress response. It may be that their response in this case is a specific effect of the action of CHP. This is the first biological function associated to any of these genes.

#### SKN7

Similarly to the genes *MSN2*/*MSN4*, there is no significant transcriptional change of *SKN7* in response to CHP. Results from the two prior studies with H_2_O_2_ stress agree with each other and show a decrease of their mRNA levels [19,20]. Therefore it appears that the *SKN7* response and mode of action may be dependent on the oxidative agent. Further differences between the responses to H_2_O_2_ and CHP are discussed below.

Lee et al. carried out a proteomics study of the yeast response to H_2_O_2_ and classified genes in three groups: those under exclusive control of Yap1p, those under exclusive control of Skn7p, and another group that depends both on Yap1p and Skn7p [33]. These studies also revealed that Skn7p is only required for the induction of about half of the genes in the *YAP1* regulon, but it is not known if these transcription factors interact physically to cooperate in the regulation of these genes [61]. The GSH system and the pentose phosphate pathway seem to be under exclusive control of Yap1p while genes related to antioxidants and thioredoxin system are regulated by both Yap1p and Skn7p [33].

Genes that are described to be regulated by Yap1p and/or Skn7p (in the Yeast Search for Transcriptional Regulators And Consensus Tracking – YEASTRACT - database [36]) are significantly over-represented in cluster B (see Figure 3). It is impossible from these results to distinguish which ones are controlled exclusively by Yap1p, Skn7, or both. Both regulons responded with similar dynamics.

### Redox and ROS-removing enzymes

**Figure 7:**
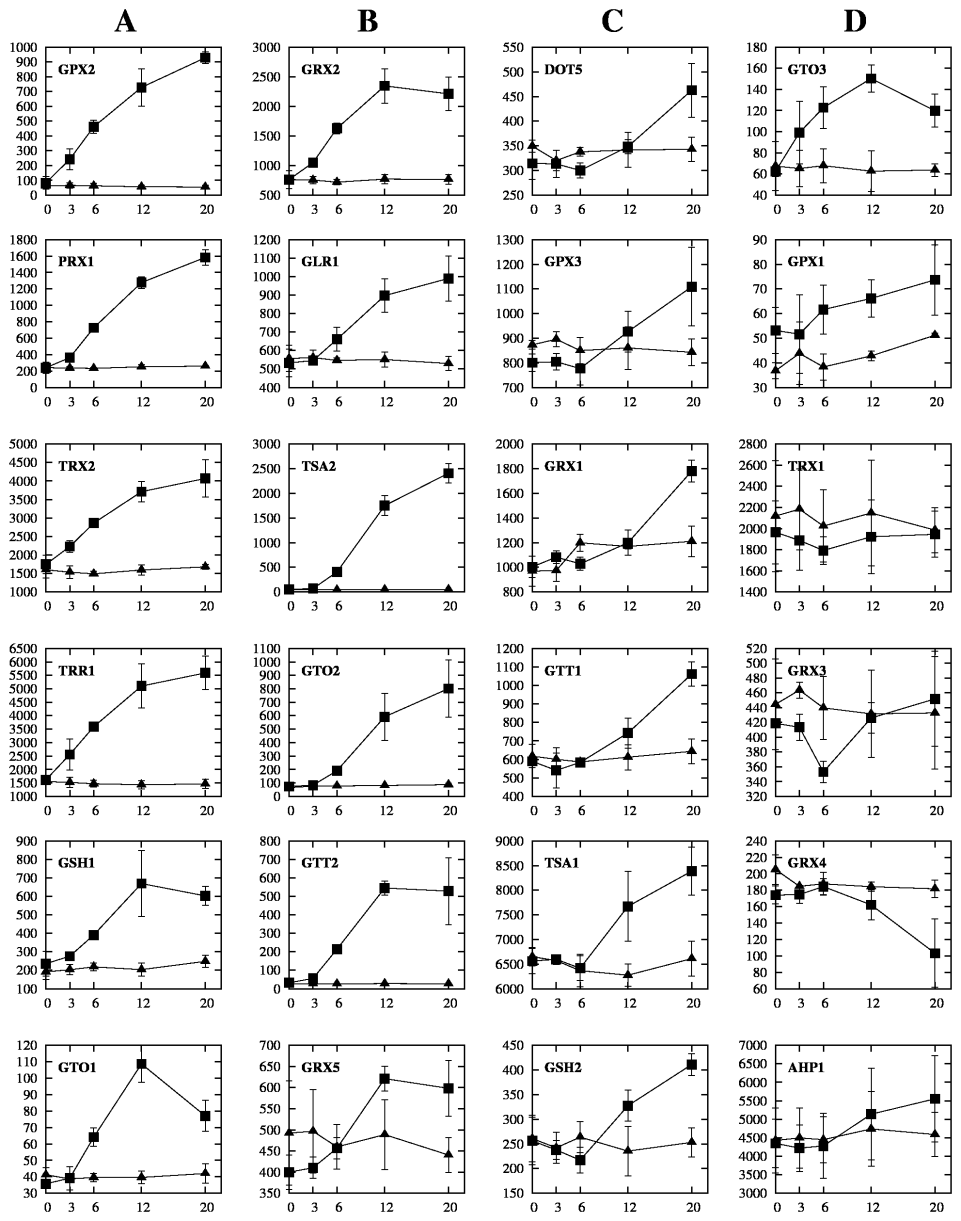
Dynamics of the transcriptional response of genes involved in the glutathione and thioredoxin systems. Columns A, B, C and D show groups of genes with different kinetics. The abscissa is in a scale of min, the ordinate in a scale of absolute value of gene expression (i.e. result of the RMA analysis transformed back to linear scale). Values plotted are medians of three biological replicates; error bars represent the standard deviations.

One of the earlier events detectable in this time course was the induction of genes encoding redox proteins, that keep the cytosol in a reduced state (glutathione, glutaredoxin, thioredoxin systems), and ROS (reactive-oxygen species)-removing enzymes (SODs and catalases). The level of *GPX2*, *PRX1, TRR1*, *TRX2, SOD2* and *CTA1* transcripts significantly increased within 3 min of exposure to CHP (Figure 7). A comparison of these results with data obtained using the chemical stressor benomyl [25] show that the response to CHP was generally faster than to benomyl. *GPX2, TRR1* and *TRX2* transcripts only display detectable changes 10 min after the addition of benomyl to the cultures, while genes encoding ROS-removing enzymes are not up-regulated in response to that drug [25]. The differences in the results are in accordance with indications that Yap1p is activated differently by peroxides than by other stressors, such as diamide [62]. Lucau-Danila et al. [25] suggest that benomyl has a similar mode of action as diamide, directly oxidizing Yap1p, whereas the peroxide action requires the involvement of Gpx3p as well [63]. The kinetics of drug entry into the cells can also account for this difference in the response speeds.

Figure 7 clearly indicates that there are four functionally distinct groups of genes in this class; genes in column A (plus *GTO3* in column D) were the fastest, showing little or no lag in response to CHP; genes in column B had a small lag, their expression level started to increase at 6 min; genes in column C displayed a longer lag, only starting to respond at 12 or 20 min. Column D shows genes that had little or no response to CHP, except *GTO3* which responded fast. We searched for patterns of regulation in these 4 groups, and found that all of the fastest genes are regulated by Yap1 and Msn2/4 and additionally by Skn7 in the cases of *GPX2, TRX2, TRR1* and *GSH1*. Only *TSA2* (in column B), *TSA1* (column C) and *AHP1* (in column D) are also regulated by these 3 transcription factors. It is likely that the concerted action of these transcription factors is needed for this fast response.

The genes *GTO1* and *DOT*5, significantly up-regulated in our work, are not responsive to H_2_O_2_-induced stress [19,20]. *GTO1* is part of a recently characterized omega-class glutathione transferase genes (EC 2.5.1.18), that also includes *GTO2* and *GTO3* [64]. All 3 genes were induced by CHP stress, with *GTO3* being the fastest. Interestingly, from the 3 proteins, only Gto3p exhibits activity against CHP [64]. The other two yeast glutathione transferase-encoding genes, *GTT1* and *GTT2*, are both up-regulated in CHP and H_2_O_2_ stress [19,20].

### Pentose phosphate pathway

The pentose phosphate pathway (PPP) has a dual role of producing reducing equivalents in the form of NADPH, and precursors for biosynthetic pathways, particularly biosynthesis of nucleic acids (from ribose), and aromatic amino acids (from erythrose 4-phosphate, a precursor of the shikimate pathway).

The oxidative branch of the PPP is the main route of production of NADPH and therefore this pathway is extremely important in the eukaryotic response to oxidative stress [65,66]. NADPH is used by glutathione reductase (Glr1p) to reduce GSSG (oxidized glutathione) that is produced when GSH reduces peroxides and other oxidants. Thioredoxin, another important antioxidant molecule, is also reduced by the NADPH-dependent thioredoxin reductase (Trr1p, Trr2p).

**Figure 8:**
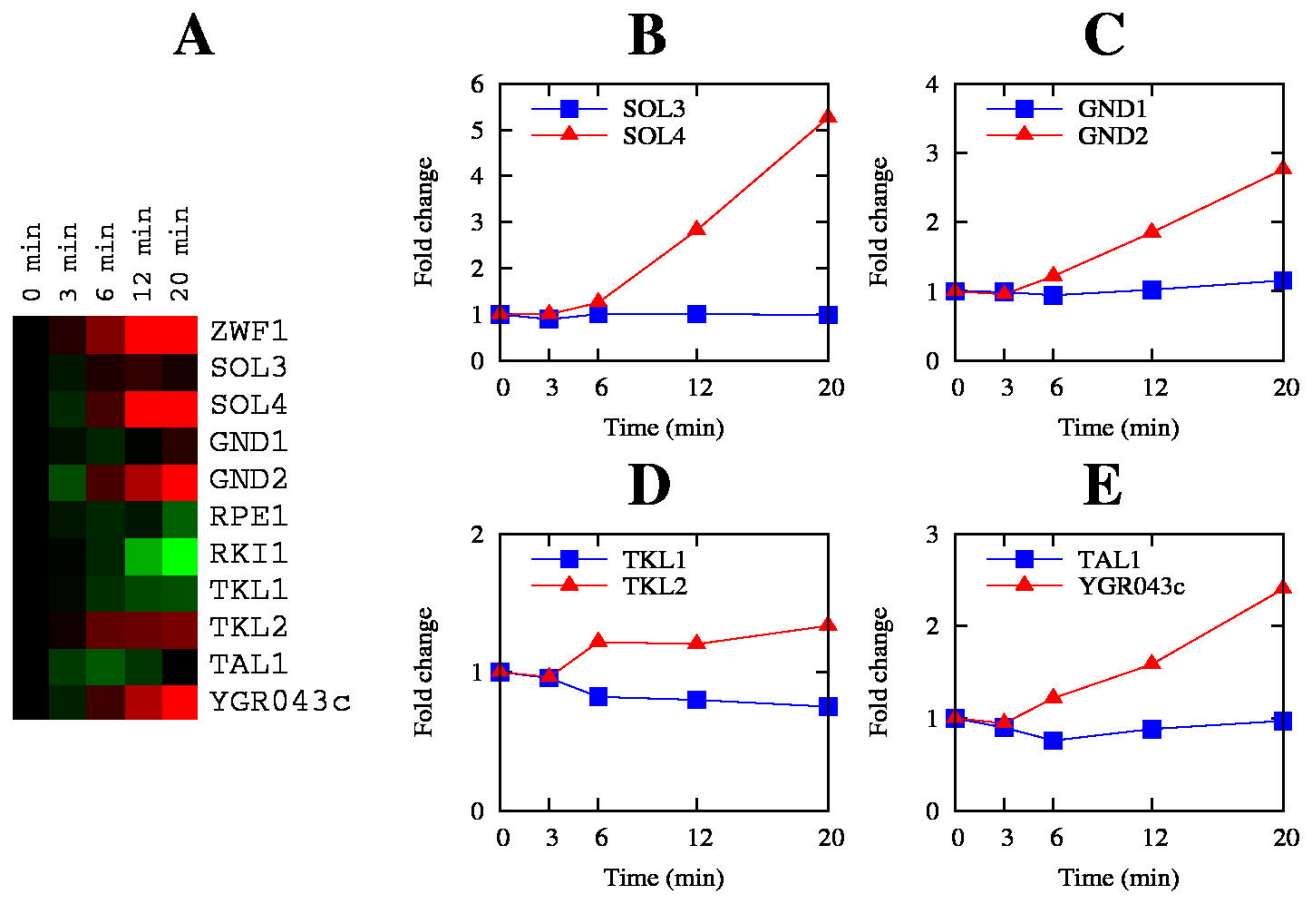
Dynamics of the transcriptional response of the pentose phosphate pathway. **A**: heat map of all genes of the pathway, details as in Figure 5; **B-D**: changes of gene expression of the four pairs of isoenzymes of the pathway; data plotted are the ratios of the median expression level divided by the median expression level of the same time series at time zero. Triangles correspond to the mRNA level in the control cultures, squares in the CHP-treated cultures.

In the present study, the physiological state of the culture changes radically, from a mode of exponential growth where the PPP is essentially producing ribose for synthesis of DNA and RNA, to a mode where growth is arrested and there is an imbalance of redox equivalents. The regulation of the PPP is tuned to these changes, as can be observed in Figure 8. Three genes encoding enzymes from the oxidative branch (*ZWF1*, *SOL4, GND2*) were quickly up-regulated, being significantly changed already at 12 min after addition of oxidant; the *RKI1* gene, encoding ribose 5-phosphate ketol-isomerase that forms the branch towards nucleic acid synthesis, was repressed.

In the PPP there are four metabolic steps that have 2 isoenzymes each: *SOL3* and *SOL4* encode 6-phosphogluconolactonases, *GND1* and *GND2* encode 6-phosphogluconate dehydrogenases, *TKL1* and *TKL2* encode transketolases; and *TAL1* and *NQM1* encode transaldolases. The expression levels of these 8 genes display a very interesting pattern: in the initial state only one of each pair is expressed to high levels (*SOL3*, *GND1*, *TKL1*, and *TAL1*) but in oxidative stress conditions, only the complementary genes (*SOL4*, *GND2*, *TKL2*, and *NQM1*) are induced (Figure 8). This suggests that each of these isoenzymes is specialized for a specific mode of operation of the PPP: *SOL4*, *GND2*, *TKL2*, and *NQM1* are needed in oxidative stress conditions, when the pathway mainly operates to maintain the NADPH/NADP^+^ ratio, while *SOL3*, *GND1*, *TKL1*, and *TAL1* are optimized for the production of precursors for growth. A similar pattern was obtained in previous studies [19,20] but this is the first time these results have been discussed. An interesting question that arises is what makes these isoenzymes specific for each condition. The process probably involves regulation by Msn2/4 since from each pair of genes, only the ones that are involved in the response to oxidative stress (*SOL4*, *GND2*, *TKL2*, *NQM1*) are documented to be regulated by these transcription factors (see Figure 6).

The important role of the PPP in the yeast response to CHP is further supported by the finding that most genes encoding glycolysis-related proteins are either down-regulated or unchanged, in accordance with a previous proteomics study [67]. However, the gene encoding glucokinase, an enzyme that catalyzes the formation of glucose-6-phosphate, the substrate for the PPP, was up-regulated. This supports the idea that, under oxidative stress, glucose-6-phosphate is diverted from energy production (glycolysis) to NADPH regeneration in the PPP. In addition to this pathway, we also see an induction of the genes of the trehalose branch. It has been suggested that trehalose quenches reactive oxygen species [68] and reduces protein aggregation, which maintains the polypeptide chains in a partially folded state, thus facilitating their refolding by chaperones [69].

### Proteasome and ubiquitin-mediated proteolysis

**Figure 9:**
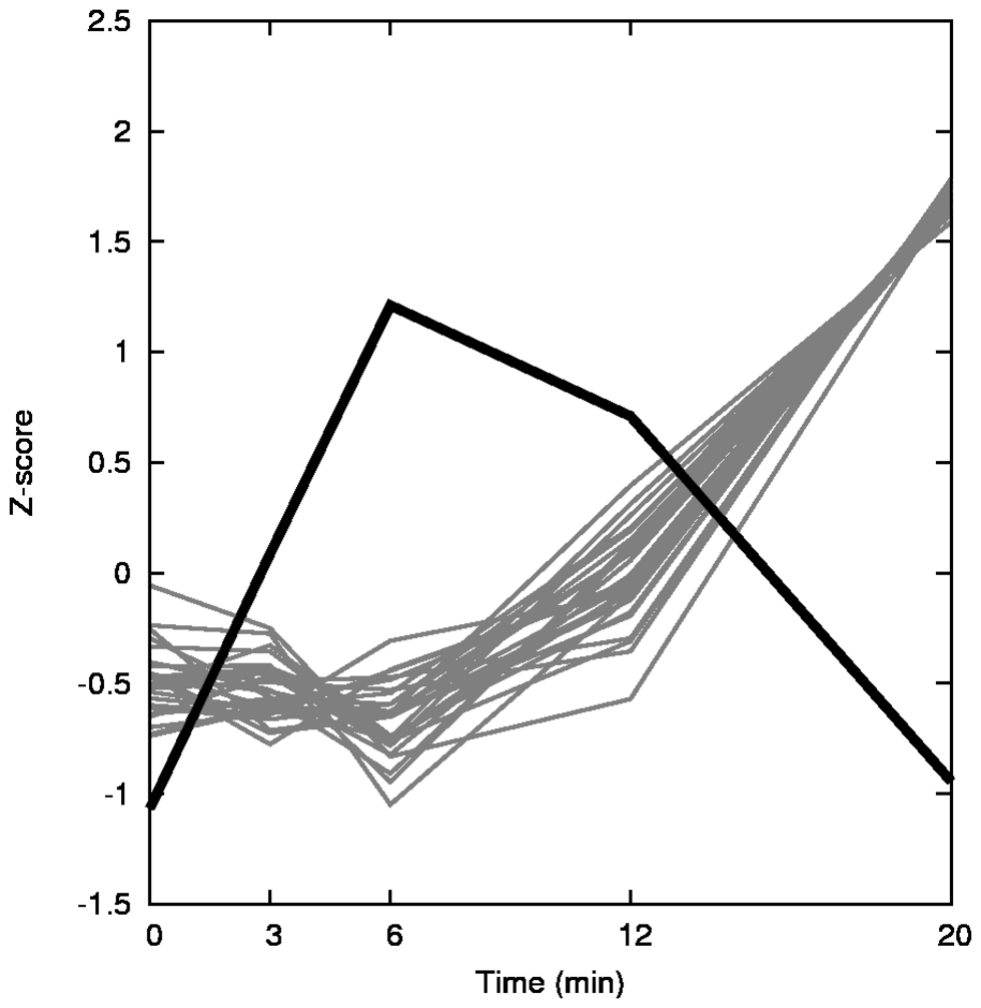
Dynamics of the transcriptional response of the proteasome genes. Gray curves correspond to the genes of the proteasome subunits, while the black thick curve corresponds to the proteasome transcription factor *RPN4*. The ordinate is in a scale of unit standard deviation.

The genes that show a late response to CHP elicitation are mainly related to proteasome and ubiquitin-mediated proteolysis (Table 1). Oxidative stress conditions lead to the accumulation of oxidant-damaged proteins with impaired function. Mildly oxidized proteins must be removed from the system before they undergo severe oxidation forming cross-linked aggregates that are poor substrates for proteases [70,71]. Substantial evidence suggests that proteolysis is responsible for degrading oxidized proteins in the cytoplasm, nucleus, and endoplasmic reticulum to avoid excessive accumulation of non-functional proteins [72]. In yeast, the ubiquitin-dependent pathway is required to withstand oxidative stress [73], since ubiquitin-mediated proteolysis serves two major functions under these conditions: removal of oxidized (damaged) proteins and rapid re-orientation of the cellular machinery towards protective OSR. Ubiquitinated proteins are processed on the eukaryotic proteasome, a highly specialized protein degradation cellular machine [74]. The transcription of genes encoding proteasome subunits is regulated by the transcription factor Rpn4p [75]. Our results show that both *RPN4* and most of the genes encoding proteasome subunits were up-regulated in response to the CHP-induced stress. Moreover, this happened in a concerted manner, with *RPN4* expression induced very early (3 min) and the genes encoding proteasome subunits induced later (20 min, see Figure 9). This provides evidence that *RPN4* is regulated at the transcriptional level (at least partially), and confirms that this Rpn4p is involved in the induction of the proteasome under oxidative stress conditions. The involvement of the proteasome in the yeast OSR was first described using a proteomics approach [67], showing the level of 12 proteasome subunits increased under H_2_O_2_-induced stress. In our experiment we observed that the transcript levels of 27 of the 31 genes encoding yeast proteasome subunits are significantly up-regulated (*p*<0.05) after CHP elicitation.

### Differential response to CHP and H_2_O_2_

Oxidative stress can be induced by a variety of agents with different modes of action, therefore cells have to maintain multiple distinct mechanisms of protection [38]. While the transcriptional response to oxidative stress induced by H_2_O_2_ [19,20], diamide [19], and menadione [19] was studied previously, the dynamics of the response to CHP was not known. Thorpe et al. [38] performed a large scale screen of viable *S. cerevisiae* deletion strains for sensitivity to 5 different oxidants, and have shown that a specific set of OSR genes is required to protect cells from CHP, and that this set is different from the set required to protect from the other oxidants tested. This study suggests that CHP has a unique mode of action, different from other peroxides, including H_2_O_2,_ and, consequently, a unique transcriptional response. We compared our results obtained using CHP with previous results where H_2_O_2_ was used as elicitor [19,20]. In our study we identified genes as differentially expressed based on proper statistical comparisons (ANOVA - ANalysis Of VAriance - with significance level at *p*<0.05 after correction for multiple testing), while previous studies with H_2_O_2_ were based on magnitude of change (larger than 2-fold changes up or down-regulated). Bearing in mind that comparing results with these different criteria has some limitations, we have identified a large group of genes that had a similar behavior in the response to the two peroxides, but several others clearly responded in different ways. Pathway analysis of these groups of genes yielded the results presented in Table S3.

Genes that were up-regulated in all three data sets are mainly involved in response to stress, glutathione metabolism and the pentose phosphate pathway (Table S3-C). Common down-regulated genes are involved in the transcription and translation processes (Table S3-D). Thus we can say with confidence that these processes are common to both transcriptional OSRs.

The set of genes up-regulated in response to CHP-induced oxidative stress but not H_2_O_2_ includes genes involved in processes related with the membrane and cell wall (Table S3-A). CHP may provoke higher damage in peripheral structures because it is larger than H_2_O_2_ and arguably much slower to penetrate the cell wall and plasma membrane, thus spending longer time outside the cell and therefore primarily damaging peripheral cell structures. The work of Thorpe et al. [38] identified vacuole and cell wall functions as needed for CHP tolerance. A different study reported that many yeast strains defective for genes involved in cell wall integrity are sensitive to bulky hydroperoxide molecules such as CHP and linoleic acid hydroperoxide, but not to the smaller H_2_O_2_ [76].

Interestingly, some genes involved in proteolysis are also specifically up-regulated in response to CHP. As discussed above, a proteome study of H_2_O_2_-induced stress had revealed earlier that only 12 genes encoding proteasome subunits were up-regulated, while our results show that 27 of the 31 genes encoding these proteins are up-regulated in response to CHP. This possibly indicates that the whole proteasome is induced and the difference in numbers between these two studies is probably due to experimental limitations of the proteomics approach.

Genes down-regulated in response to CHP but not H_2_O_2_ are mainly involved in mitochondrial processes – electron transport and oxidative phosphorylation (Table S3-B). Thorpe et al. identified the electron transport chain as vital for H_2_O_2_ tolerance but not for CHP [38]. Conflicting results about the role of mitochondria in the OSR have been reported previously. Some studies show that mitochondrial function is required for yeast resistance to oxidative stress [77] and a cluster of genes involved in oxidative phosphorylation was observed to be up-regulated as part of the environmental stress response [19]. This may be due to a higher demand of ATP in oxidative stress conditions, to provide energy for processes such as repair of damaged proteins, detoxification of lipoperoxidation products and transport of oxidized molecules [77]. Alternatively this could also be because respiring yeast cells already contain a considerable level of antioxidants to protect them from ROS derived from “normal” respiration (and so are more resistant to external sources of ROS, unlike in non-respiring states, where they would have lower levels of antioxidants). However, other studies have shown that cells lacking their entire mitochondrial genome were remarkably more resistant to oxidative stress than cells with functional mitochondria [78]. For example, unlike the response to hydrogen peroxide, rho zero petites are very resistant to linoleic acid hydroperoxide [79]. It is not clear then why CHP-induced stress appears to cause a transient down-regulation in electron transport and oxidative phosphorylation while H_2_O_2_ causes their induction but these results constitute additional evidence of a different yeast OSR to two different peroxides.

In conclusion, this study reveals for the first time the dynamics of the early yeast transcriptional response to oxidative stress induced by the aromatic peroxide CHP. Our work illustrates how a high quality transcriptomic dataset can be obtained by using a well planned experimental design that includes, i) tightly controlled culture conditions, ii) biological replicates and, iii) appropriate temporal controls. At the physiological level, yeast cultures show an efficient response to the stress, by removing most of the oxidant within 20 min (Figure 1). The dissection of the transcriptional response behind this physiological response revealed a set of very early regulated genes, many of which were not previously implicated in the OSR. This includes several transcriptional factors, indicating that there is a complex cascading regulation leading to a coordinated and extensive transcriptional reprogramming. This coordination is well illustrated by the role of the proteasome, with the early up-regulation of the gene *RPN4*, encoding the proteasome regulator, and a later up-regulation of genes that are known targets of Rpn4p and that encode the several proteasome subunits (Figure 9). Several early regulated genes are involved in the glutathione, thioredoxin and ROS-removing systems and these provide the “direct” response to oxidative stress, dealing with the oxidant and reactive species derived from its metabolism. Redox balancing also seems to involve regulation of carbohydrate metabolism, with glucose-6-phosphate being diverted from glycolysis into the PPP, which under oxidative stress operates mainly to produce reducing equivalents in the form of NADPH. From the main transcriptional regulators of the OSR, encoded by *MSN2/4, SKN7* and *YAP1*, only the last one is regulated at the transcriptional level, suggesting that its mechanism of action is more complex than just the proposed mechanism of cytoplasm/nucleus oxidant-regulated translocation [12]. Three other genes from the *YAP* family, *YAP3, YAP5* and *YAP7*, whose function was previously unknown, are also involved in the response to CHP, being up-regulated during the first 20 min after the addition of the oxidant.

The transcriptional response to CHP is different from the response to H_2_O_2_. We identified 664 genes that are specifically involved in the response to CHP, and were not responsive to H_2_O_2_ treatment [19,20]. This high number clearly shows the difference in the response to the two peroxides, as previously suggested by mutant sensitivity studies [38]. Most of these genes are up-regulated and these are involved in processes related to cell wall and proteolysis. The down-regulated ones are involved in mitochondrial processes, a category that is up-regulated in the yeast response to H_2_O_2_.

Several of the early transiently responding genes identified in our study have no assigned function yet and future studies may help to identify their precise role in the OSR in yeast.

## Supporting Information

Figure S1 **Down-regulation of genes related to cell cycle processes.**
(DOC)

Figure S2 **Down-regulation of genes encoding ribosome subunits.**
(DOC)

Figure S3 **Down-regulation of genes encoding RNA polymerase subunits.**
(DOC)

Table S1 **List of the genes included in each one of the five clusters obtained by *k*-means clustering, shown** in Figure 3.
(XLS)

Table S2 **Transiently early regulated genes.** A. Genes significantly up-regulated within 6 min. B. Genes significantly down-regulated within 6 min.
(DOC)

Table S3 **GO analysis of the differential expression of genes in CHP and H_2_O_2_.** A. Genes differentially up-regulated in CHP-induced stress (not up-regulated in H_2_O_2_). B. Genes differentially down-regulated in CHP-induced stress (not down-regulated in H_2_O_2_). C. Genes up-regulated in CHP and H_2_O_2_-induced stress. D. Genes down-regulated in CHP and H_2_O_2_-induced stress.
(DOC)
