# Supplementary material for: The Genome-Wide Early Temporal Response of Saccharomyces cerevisiae to Oxidative Stress Induced by Cumene Hydroperoxide
Source: PLoS One. 2013 Sep 20;8(9):e74939. doi: 10.1371/journal.pone.0074939 (PMC3779239; doi:10.1371/journal.pone.0074939)
Supplement: Figure S1 — Down-regulation of genes related to cell cycle processes. (DOC) [file pone.0074939.s001.doc]

**Figure S1.** Down-regulation of genes related to cell cycle processes. **A**: temporal changes in the expression of genes encoding transcription factors involved in the cell cycle *ACE2*, *FKH1*, *FKH2*, *HCM1*, *MCM1*, *NDD1*, *TOS4*, *SWI5*, *SWI6* and *YHP1*. **B**: transcriptional changes of other genes (not transcription factors) that are involved in the cell cycle process.
