# Supplementary material for: The Genome-Wide Early Temporal Response of Saccharomyces cerevisiae to Oxidative Stress Induced by Cumene Hydroperoxide
Source: PLoS One. 2013 Sep 20;8(9):e74939. doi: 10.1371/journal.pone.0074939 (PMC3779239; doi:10.1371/journal.pone.0074939)
Supplement: Figure S2 — Down-regulation of genes encoding ribosome subunits. (DOC) [file pone.0074939.s002.doc]

**Figure S2**. Down-regulation of genes encoding ribosome subunits.

**Figure S3.** Down-regulation of genes encoding RNA polymerase subunits.
