# Supplementary material for: The Genome-Wide Early Temporal Response of Saccharomyces cerevisiae to Oxidative Stress Induced by Cumene Hydroperoxide
Source: PLoS One. 2013 Sep 20;8(9):e74939. doi: 10.1371/journal.pone.0074939 (PMC3779239; doi:10.1371/journal.pone.0074939)
Supplement: Table S2 — Transiently early regulated genes. A. Genes significantly up-regulated within 6 min. B. Genes significantly down-regulated within 6 min. (DOC) [file pone.0074939.s005.doc]

**Tables S2. Transiently early regulated genes.** Genes whose expression level was significantly changed (*p* < 0.05) within 6 min after the addition of CHP but which were back to normal levels at 12 min.

**Table S2-A. Genes significantly up-regulated within 6 min**.

| ***ORF*** | ***Gene*** | ***Description (GO Annotation)*** |
| --- | --- | --- |
| **Transcription factors** | | |
| YJR147w | *HMS2* | Protein with similarity to heat shock transcription factors; overexpression suppresses the pseudohyphal filamentation defect of a diploid mep1 mep2 homozygous null mutant |
| YIR017c | *MET28* | Basic leucine zipper (bZIP) transcriptional activator in the Cbf1p-Met4p-Met28p complex, participates in the regulation of sulfur metabolism |
| YPR168w | *NUT2* | Subunit of the RNA polymerase II mediator complex; associates with core polymerase subunits to form the RNA polymerase II holoenzyme; required for transcriptional activation and has a role in basal transcription |
| YPR065w | *ROX1* | Heme-dependent repressor of hypoxic genes; contains an HMG domain that is responsible for DNA bending activity |
| YPR009w | *SUT2* | Putative transcription factor; multicopy suppressor of mutations that cause low activity of the cAMP/protein kinase A pathway; highly similar to Sut1p |
| YIR018w | *YAP5* | Basic leucine zipper (bZIP) transcription factor |
| **Stress response or drug resistance** | | |
| YNL133c | *FYV6* | Protein of unknown function, required for survival upon exposure to K1 killer toxin; proposed to regulate double-strand break repair via non-homologous end-joining |
| YDL049c | *KNH1* | Protein with similarity to Kre9p, which is involved in cell wall beta 1,6-glucan synthesis; overproduction suppresses growth defects of a kre9 null mutant; required for propionic acid resistance |
| YER038c | *KRE29* | Essential subunit of the Mms21-Smc5-Smc6 complex; required for growth and DNA repair; heterozygous mutant shows haploinsufficiency in K1 killer toxin resistance |
| YNL032w | *SIW14* | Tyrosine phosphatase that plays a role in actin filament organization and endocytosis; localized to the cytoplasm |
| **Cell wall, cytoskeleton, cell integrity** | | |
| YHR101c | *BIG1* | Integral membrane protein of the endoplasmic reticulum, required for normal content of cell wall beta-1,6-glucan |
| YCL029c | *BIK1* | Microtubule-associated protein, component of the interface between microtubules and kinetochore, involved in sister chromatid separation; essential in polyploid cells but not in haploid or diploid cells; ortholog of mammalian CLIP-170 |
| YOR284w | *HUA2* | Cytoplasmic protein of unknown function; computational analysis of large-scale protein-protein interaction data suggests a possible role in actin patch assembly |
| YNL180c | *RHO5* | Non-essential small GTPase of the Rho/Rac subfamily of Ras-like proteins, likely involved in protein kinase C (Pkc1p)-dependent signal transduction pathway that controls cell integrity |
| **Sporulation** | | |
| YDR402c | *DIT2* | N-formyltyrosine oxidase, sporulation-specific microsomal enzyme involved in the production of N,N-bisformyl dityrosine required for spore wall maturation, homologous to cytochrome P-450s |
| YNL210w | *MER1* | Protein with RNA-binding motifs required for meiosis-specific mRNA splicing; required for chromosome pairing and meiotic recombination |
| YLL005c | *SPO75* | Meiosis-specific protein of unknown function, required for spore wall formation during sporulation; dispensable for both nuclear divisions during meiosis |
| YOR338w | *---* | Putative protein of unknown function; YOR338W transcription is regulated by Azf1p and its transcript is a specific target of the G protein effector Scp160p; identified as being required for sporulation in a high-throughput mutant screen |
| **Ubiquitin** | | |
| YPR169w | *JIP5* | Nucleolar protein of unknown function, exhibits a physical interaction with Bre1p |
| YDL122w | *UBP1* | Ubiquitin-specific protease that removes ubiquitin from ubiquitinated proteins; cleaves at the C terminus of ubiquitin fusions irrespective of their size; capable of cleaving polyubiquitin chains |
| **Others** | | |
| YKL149c | DBR1 | RNA lariat debranching enzyme, involved in intron turnover; required for efficient Ty1 transposition |
| YEL046c | *GLY1* | Threonine aldolase, catalyzes the cleavage of L-allo-threonine and L-threonine to glycine; involved in glycine biosynthesis |
| YMR081c | *ISF1* | Serine-rich, hydrophilic protein with similarity to Mbr1p; overexpression suppresses growth defects of hap2, hap3, and hap4 mutants; expression is under glucose control; cotranscribed with NAM7 in a cyp1 mutant |
| YGR289c | *MAL11* | Inducible high-affinity maltose transporter (alpha-glucoside transporter); encoded in the MAL1 complex locus; member of the 12 transmembrane domain superfamily of sugar transporters; broad substrate specificity that includes maltotriose |
| YMR177w | *MMT1* | Putative metal transporter involved in mitochondrial iron accumulation; closely related to Mmt2p |
| YKL198c | *PTK1* | Putative serine/threonine protein kinase that regulates spermine uptake; involved in polyamine transport; possible mitochondrial protein |
| YOL010w | *RCL1* | Subunit of U3-containing 90S preribosome processome complex involved in 18S rRNA biogenesis and small ribosomal subunit assembly; stimulates Bms1p GTPase and U3 binding activity; similar to RNA cyclase-like proteins but no activity detected |
| YIL166c | *---* | Putative protein with similarity to the allantoate permease (Dal5p) subfamily of the major facilitator superfamily; mRNA expression is elevated by sulfur limitation; YIL166C is a non-essential gene |
| YNL234w | *--* | Similar to globins and has a functional heme-binding domain; involved in glucose signaling or metabolism; regulated by Rgt1p |
| **Biological process unknown** | | |
| YHR126c | *ANS1* | Putative protein of unknown function; transcription dependent upon Azf1p |
| YHR049w | *FSH1* | Putative serine hydrolase that localizes to both the nucleus and cytoplasm; sequence is similar to S. cerevisiae Fsh2p and Fsh3p and the human candidate tumor suppressor OVCA2 |
| YIL171w | *HXT12* | Possible pseudogene in strain S288C; YIL171W and the adjacent ORF, YIL170W/HXT12, together encode a non-functional member of the hexose transporter family |
| YPL250c | *ICY2* | Protein of unknown function; mobilized into polysomes upon a shift from a fermentable to nonfermentable carbon source; potential Cdc28p substrate |
| YOL015w | *IRC10* | Putative protein of unknown function; null mutant displays increased levels of spontaneous Rad52 foci |
| YJL162c | *JJJ2* | Protein of unknown function, contains a J-domain, which is a region with homology to the E. coli DnaJ protein |
| YIL176c | *PAU14* | Protein of unknown function, member of the seripauperin multigene family encoded mainly in subtelomeric regions; identical to Pau1p |
| YDL183c | *---* | Mitochondrial inner-membrane protein thought to be involved in the formation of an active mitochondrial K+/H+ exchanger (KHE) system; non-essential gene |
| YGL108c | *---* | Protein of unknown function, predicted to be palmitoylated; green fluorescent protein (GFP)-fusion protein localizes to the cell periphery |
| YGR031w | *---* | Putative protein of unknown function; the authentic, non-tagged protein is detected in highly purified mitochondria in high-throughput studies |
| YGR035c | *---* | Putative protein of unknown function, potential Cdc28p substrate; transcription is activated by paralogous transcription factors Yrm1p and Yrr1p along with genes involved in multidrug resistance |
| YJL218w | *---* | Putative protein of unknown function, similar to bacterial galactoside O-acetyltransferases; induced by oleate in an OAF1/PIP2-dependent manner; promoter contains an oleate response element consensus sequence; non-essential gene |
| YJR115w | *---* | Putative protein of unknown function |
| YKR075c | *---* | Protein of unknown function; similar to YOR062Cp and Reg1p; expression regulated by glucose and Rgt1p; GFP-fusion protein is induced in response to the DNA-damaging agent MMS |
| YLR287c | *---* | Putative protein of unknown function; green fluorescent protein (GFP)-fusion protein localizes to the cytoplasm; YLR287C is not an essential gene |
|  |  |  |

**Table S2-B. Genes significantly down-regulated within 6 min**.

| *ORF* | *Gene* | *Description (GO Annotation)* | |
| --- | --- | --- | --- |
| **DNA replication, cell growth and division** | | | |
| YJR065c | *ARP3* | Essential component of the Arp2/3 complex, which is a highly conserved actin nucleation center required for the motility and integrity of actin patches; involved in endocytosis and membrane growth and polarity | |
| YKL092c | *BUD2* | GTPase activating factor for Rsr1p/Bud1p required for both axial and bipolar budding patterns; mutants exhibit random budding in all cell types | |
| YOR039w | *CKB2* | Beta' regulatory subunit of casein kinase 2, a Ser/Thr protein kinase with roles in cell growth and proliferation; the holoenzyme also contains CKA1, CKA2 and CKB1, the many substrates include transcription factors and all RNA polymerases | |
| YAL040c | *CLN3* | G1 cyclin involved in cell cycle progression; activates Cdc28p kinase to promote the G1 to S phase transition; plays a role in regulating transcription of the other G1 cyclins, CLN1 and CLN2; regulated by phosphorylation and proteolysis | |
| YPL219w | *PCL8* | Cyclin, interacts with Pho85p cyclin-dependent kinase (Cdk) to phosphorylate and regulate glycogen synthase, also activates Pho85p for Glc8p phosphorylation | |
| YOL094c | *RFC4* | Subunit of heteropentameric Replication factor C (RF-C), which is a DNA binding protein and ATPase that acts as a clamp loader of the proliferating cell nuclear antigen (PCNA) processivity factor for DNA polymerases delta and epsilon | |
| YDR247w | *VHS1* | Cytoplasmic serine/threonine protein kinase; identified as a high-copy suppressor of the synthetic lethality of a sis2 sit4 double mutant, suggesting a role in G1/S phase progression; homolog of Sks1p | |
| YLR410w | *VIP1* | Inositol hexakisphosphate (IP6) and inositol heptakisphosphate (IP7) kinase; generation of IP7 by Vip1p is important for phosphate signaling; likely involved in cortical actin cytoskeleton function, by analogy with S. pombe ortholog asp1 | |
| YNL321w | *VNX1* | Low affinity vacuolar membrane localized monovalent cation/H+ antiporter; member of the calcium exchanger (CAX) family; potential Cdc28p substrate | |
| **Transcription** | | | |
| YMR033w | *ARP9* | Component of both the SWI/SNF and RSC chromatin remodeling complexes; actin-related protein involved in transcriptional regulation | |
| YDL070w | *BDF2* | Protein involved in transcription initiation at TATA-containing promoters; associates with the basal transcription factor TFIID; contains two bromodomains; corresponds to the C-terminal region of mammalian TAF1; redundant with Bdf1p | |
| YER107c | *GLE2* | Component of the Nup82 subcomplex of the nuclear pore complex; required for polyadenylated RNA export but not for protein import; homologous to S. pombe Rae1p | |
| YNR023w | *SNF12* | 73 kDa subunit of the SWI/SNF chromatin remodeling complex involved in transcriptional regulation; homolog of Rsc6p subunit of the RSC chromatin remodeling complex; deletion mutants are temperature-sensitive | |
| **Translation** | | | |
| YNL306w | *MRPS18* | Mitochondrial ribosomal protein of the small subunit; essential for viability, unlike most other mitoribosomal proteins | |
| YKL006w | *RPL14A* | N-terminally acetylated protein component of the large (60S) ribosomal subunit, nearly identical to Rpl14Bp and has similarity to rat L14 ribosomal protein; rpl14a csh5 double null mutant exhibits synthetic slow growth | |
| YMR242c | *RPL20A* | Protein component of the large (60S) ribosomal subunit, nearly identical to Rpl20Bp and has similarity to rat L18a ribosomal protein | |
| YLR344W | *RPL26A* | Protein component of the large (60S) ribosomal subunit, nearly identical to Rpl26Bp and has similarity to E. coli L24 and rat L26 ribosomal proteins; binds to 5.8S rRNA | |
| YJL138c | *TIF2* | Translation initiation factor eIF4A, identical to Tif1p; DEA(D/H)-box RNA helicase that couples ATPase activity to RNA binding and unwinding; forms a dumbbell structure of two compact domains connected by a linker; interacts with eIF4 | |
| **Mitochondria** | | | |
| YMR282c | *AEP2* | Mitochondrial protein, likely involved in translation of the mitochondrial OLI1 mRNA; exhibits genetic interaction with the OLI1 mRNA 5'-untranslated leader | |
| YJL003W | *COX16* | Mitochondrial inner membrane protein, required for assembly of cytochrome c oxidase | |
| YNR041c | *COQ2* | Para hydroxybenzoate: polyprenyl transferase, catalyzes the second step in ubiquinone (coenzyme Q) biosynthesis | |
| YKL150W | *MCR1* | Mitochondrial NADH-cytochrome b5 reductase, involved in ergosterol biosynthesis | |
| YCR028C-A | *RIM1* | Single-stranded DNA-binding protein essential for mitochondrial genome maintenance; involved in mitochondrial DNA replication | |
| **Vesicle trafficking** | | | |
| YAL042w | *ERV46* | Protein localized to COPII-coated vesicles, forms a complex with Erv41p; involved in the membrane fusion stage of transport | |
| YKL176c | *LST4* | Protein possibly involved in a post-Golgi secretory pathway; required for the transport of nitrogen-regulated amino acid permease Gap1p from the Golgi to the cell surface | |
| YDR189w | *SLY1* | Hydrophilic protein involved in vesicle trafficking between the ER and Golgi; SM (Sec1/Munc-18) family protein that binds the tSNARE Sed5p and stimulates its assembly into a trans-SNARE membrane-protein complex | |
| **Others** | | | |
| YLR028c | *ADE16* | | Enzyme of 'de novo' purine biosynthesis containing both 5-aminoimidazole-4-carboxamide ribonucleotide transformylase and inosine monophosphate cyclohydrolase activities, isozyme of Ade17p; ade16 ade17 mutants require adenine and histidine |
| YDR155c | *CPR1* | | Cytoplasmic peptidyl-prolyl cis-trans isomerase (cyclophilin), catalyzes the cis-trans isomerization of peptide bonds N-terminal to proline residues; binds the drug cyclosporin A |
| YKL152c | *GPM1* | | Tetrameric phosphoglycerate mutase, mediates the conversion of 3-phosphoglycerate to 2-phosphoglycerate during glycolysis and the reverse reaction during gluconeogenesis |
| YJR075w | *HOC1* | | Alpha-1,6-mannosyltransferase involved in cell wall mannan biosynthesis; subunit of a Golgi-localized complex that also contains Anp1p, Mnn9p, Mnn11p, and Mnn10p; identified as a suppressor of a cell lysis sensitive pkc1-371 allele |
| YBR199w | *KTR4* | | Putative mannosyltransferase involved in protein glycosylation; member of the KRE2/MNT1 mannosyltransferase family |
| YNL085w | *MKT1* | | Protein that forms a complex with Pbp1p that may mediate posttranscriptional regulation of HO endonuclease; involved in propagation of M2 dsRNA satellite of L-A virus; allelic variation affects mitochondrial genome stability |
| YLR285w | *NNT1* | | Putative nicotinamide N-methyltransferase, has a role in rDNA silencing and in lifespan determination |
| YNL091w | *NST1* | | Protein of unknown function, mediates sensitivity to salt stress; interacts physically with the splicing factor Msl1p and also displays genetic interaction with MSL1 |
| YKL057c | *NUP120* | | Subunit of the Nup84p subcomplex of the nuclear pore complex (NPC), required for even distribution of NPCs around the nuclear envelope, involved in establishment of a normal nucleocytoplasmic concentration gradient of the GTPase Gsp1p |
| YMR247c | *RKR1* | | Nuclear RING domain protein with functional connections to chromatin modification; may interact with ribosomes, based on co-purification experiments; YMR247C is not an essential gene |
| YLR254c | *TAL1* | | Transaldolase, enzyme in the non-oxidative pentose phosphate pathway; converts sedoheptulose 7-phosphate and glyceraldehyde 3-phosphate to erythrose 4-phosphate and fructose 6-phosphate |
| YKL126w | *YPK1* | | Serine/threonine protein kinase required for receptor-mediated endocytosis; involved in sphingolipid-mediated and cell integrity signaling pathways; localized to the bud neck, cytosol and plasma membrane; homolog of mammalian kinase SGK |
| YLR447c | *VMA6* | | Subunit d of the five-subunit V0 integral membrane domain of vacuolar H+-ATPase (V-ATPase), an electrogenic proton pump found in the endomembrane system; stabilizes VO subunits; required for V1 domain assembly on the vacuolar membrane |
| YJR126c | *VPS70* | | Protein of unknown function involved in vacuolar protein sorting |
| YMR099c | *---* | | Glucose-6-phosphate 1-epimerase (hexose-6-phosphate mutarotase), likely involved in carbohydrate metabolism; GFP-fusion protein localizes to both the nucleus and cytoplasm and is induced in response to the DNA-damaging agent MMS |
| **Biological process unknown** | | | |
| YDL237W | *AIM6* | Putative protein of unknown function, required for respiratory growth; YDL237W is not an essential gene | |
| YDR063w | *AIM7* | Putative protein of unknown function; green fluorescent protein (GFP)-fusion protein localizes to the cytoplasm and nucleus; null mutant is viable and displays elevated frequency of mitochondrial genome loss | |
| YLR094C | *GIS3* | Protein of unknown function | |
| YPL184C | *MRN1* | RNA binding protein proposed to be involved in translational regulation; binds specific | |
|  |  | categories of mRNAs, including those that contain upstream open reading frames (uORFs) and internal ribosome entry sites (IRES) | |
| YDR128W | *MTC5* | Protein of unknown function; mtc5 is synthetically sick with cdc13-1 | |
| YLR351c | *NIT3* | Nit protein, one of two proteins in S. cerevisiae with similarity to the Nit domain of NitFhit from fly and worm and to the mouse and human Nit protein which interacts with the Fhit tumor suppressor; nitrilase superfamily member | |
| YKL039w | *PTM1* | Protein of unknown function, copurifies with late Golgi vesicles containing the v-SNARE Tlg2p | |
| YOR086c | *TCB1* | Lipid-binding protein containing three calcium and lipid binding domains; non-tagged protein localizes to mitochondria and GFP-fusion protein localizes to the cell periphery; C-termini of Tcb1p, Tcb2p and Tcb3p interact | |
| YLR225c | *---* | Putative protein of unknown function; green fluorescent protein (GFP)-fusion protein localizes to the cytoplasm; YLR225C is not an essential gene | |
| YNR021w | *---* | Putative protein of unknown function; green fluorescent protein (GFP)-fusion protein localizes to the endoplasmic reticulum; YNR021W is not an essential gene | |
