# Supplementary material for: The Genome-Wide Early Temporal Response of Saccharomyces cerevisiae to Oxidative Stress Induced by Cumene Hydroperoxide
Source: PLoS One. 2013 Sep 20;8(9):e74939. doi: 10.1371/journal.pone.0074939 (PMC3779239; doi:10.1371/journal.pone.0074939)
Supplement: Table S3 — GO analysis of the differential expression of genes in CHP and H2O2. A. Genes differentially up-regulated in CHP-induced stress (not up-regulated in H2O2). B. Genes differentially down-regulated in CHP-induced stress (not down-regulated in H2O2). C. Genes up-regulated in CHP and H2O2-induced stress. D. Genes down-regulated in CHP and H2O2-induced stress. (DOC) [file pone.0074939.s006.doc]

**Tables S3. GO analysis of the differential expression of genes in CHP and H_2_O_2_.** Data obtained in our work (genes significantly changed, *p*<0.05) was compared with data obtained in the works of Gasch and Causton (genes changed more than 2-fold in at least one time point) [1, 2].

**Table S3-A. Genes differentially up-regulated in CHP-induced stress (not up-regulated in H_2_O_2_**).

| **Gene Ontology term (AMIGO)** | **Cluster**  **frequency** | **p-value** | **Genes annotated to the term** |
| --- | --- | --- | --- |
| Proteolysis | 37 out of 498 genes, 7.4% | 3.06e-7 | [*ADD66*](http://db.yeastgenome.org/cgi-bin/locus.pl?locus=ADD66)*,* [*AFG3*](http://db.yeastgenome.org/cgi-bin/locus.pl?locus=AFG3)*,* [*APC11*](http://db.yeastgenome.org/cgi-bin/locus.pl?locus=APC11)*,* [*ATG4*](http://db.yeastgenome.org/cgi-bin/locus.pl?locus=ATG4)*,* [*CPS1*](http://db.yeastgenome.org/cgi-bin/locus.pl?locus=CPS1)*,* [*CYM1*](http://db.yeastgenome.org/cgi-bin/locus.pl?locus=CYM1)*,* [*DOA1*](http://db.yeastgenome.org/cgi-bin/locus.pl?locus=DOA1)*,* [*DSK2*](http://db.yeastgenome.org/cgi-bin/locus.pl?locus=DSK2)*,* [*HRD3*](http://db.yeastgenome.org/cgi-bin/locus.pl?locus=HRD3)*,* [*MET30*](http://db.yeastgenome.org/cgi-bin/locus.pl?locus=MET30)*,* [*NAS2*](http://db.yeastgenome.org/cgi-bin/locus.pl?locus=NAS2)*,* [*PNG1*](http://db.yeastgenome.org/cgi-bin/locus.pl?locus=PNG1)*,* [*PRE4*](http://db.yeastgenome.org/cgi-bin/locus.pl?locus=PRE4)*,* [*PRE5*](http://db.yeastgenome.org/cgi-bin/locus.pl?locus=PRE5)*,* [*PRE7*](http://db.yeastgenome.org/cgi-bin/locus.pl?locus=PRE7)*,* [*PUP1*](http://db.yeastgenome.org/cgi-bin/locus.pl?locus=PUP1)*,* [*PUP2*](http://db.yeastgenome.org/cgi-bin/locus.pl?locus=PUP2)*,* [*PUP3*](http://db.yeastgenome.org/cgi-bin/locus.pl?locus=PUP3)*,* [*QRI8*](http://db.yeastgenome.org/cgi-bin/locus.pl?locus=QRI8)*,* [*RIM20*](http://db.yeastgenome.org/cgi-bin/locus.pl?locus=RIM20)*,* [*RPN1*](http://db.yeastgenome.org/cgi-bin/locus.pl?locus=RPN1)*,* [*RPN10*](http://db.yeastgenome.org/cgi-bin/locus.pl?locus=RPN10)*,* [*RPN13*](http://db.yeastgenome.org/cgi-bin/locus.pl?locus=RPN13)*,* [*RPN14*](http://db.yeastgenome.org/cgi-bin/locus.pl?locus=RPN14)*,* [*RPT1*](http://db.yeastgenome.org/cgi-bin/locus.pl?locus=RPT1)*,* [*RTT101*](http://db.yeastgenome.org/cgi-bin/locus.pl?locus=RTT101)*,* [*SCL1*](http://db.yeastgenome.org/cgi-bin/locus.pl?locus=SCL1)*,* [*SEM1*](http://db.yeastgenome.org/cgi-bin/locus.pl?locus=SEM1)*,* [*SHP1*](http://db.yeastgenome.org/cgi-bin/locus.pl?locus=SHP1)*,* [*SKP1*](http://db.yeastgenome.org/cgi-bin/locus.pl?locus=SKP1)*,* [*SNF7*](http://db.yeastgenome.org/cgi-bin/locus.pl?locus=SNF7)*,* [*STS1*](http://db.yeastgenome.org/cgi-bin/locus.pl?locus=STS1)*,* [*SWM1*](http://db.yeastgenome.org/cgi-bin/locus.pl?locus=SWM1)*,* [*TUL1*](http://db.yeastgenome.org/cgi-bin/locus.pl?locus=TUL1)*,* [*UFO1*](http://db.yeastgenome.org/cgi-bin/locus.pl?locus=UFO1)*,* [*VID28*](http://db.yeastgenome.org/cgi-bin/locus.pl?locus=VID28)*,* [*YDR306C*](http://db.yeastgenome.org/cgi-bin/locus.pl?locus=YDR306C) |
| Vesicle-mediated transport | 52 out of 498 genes, 10.4% | 3.19e-6 | [*AKL1*](http://db.yeastgenome.org/cgi-bin/locus.pl?locus=AKL1)*,* [*APL2*](http://db.yeastgenome.org/cgi-bin/locus.pl?locus=APL2)*,* [*APL4*](http://db.yeastgenome.org/cgi-bin/locus.pl?locus=APL4)*,* [*APM2*](http://db.yeastgenome.org/cgi-bin/locus.pl?locus=APM2)*,* [*APS2*](http://db.yeastgenome.org/cgi-bin/locus.pl?locus=APS2)*,* [*ARK1*](http://db.yeastgenome.org/cgi-bin/locus.pl?locus=ARK1)*,* [*ATG20*](http://db.yeastgenome.org/cgi-bin/locus.pl?locus=ATG20)*,* [*BCH1*](http://db.yeastgenome.org/cgi-bin/locus.pl?locus=BCH1)*,* [*BUD7*](http://db.yeastgenome.org/cgi-bin/locus.pl?locus=BUD7)*,* [*CHS5*](http://db.yeastgenome.org/cgi-bin/locus.pl?locus=CHS5)*,* [*COY1*](http://db.yeastgenome.org/cgi-bin/locus.pl?locus=COY1)*,* [*DID2*](http://db.yeastgenome.org/cgi-bin/locus.pl?locus=DID2)*,* [*EHD3*](http://db.yeastgenome.org/cgi-bin/locus.pl?locus=EHD3)*,* [*ENT2*](http://db.yeastgenome.org/cgi-bin/locus.pl?locus=ENT2)*,* [*ENT5*](http://db.yeastgenome.org/cgi-bin/locus.pl?locus=ENT5)*,* [*ERV41*](http://db.yeastgenome.org/cgi-bin/locus.pl?locus=ERV41)*,* [*GYP8*](http://db.yeastgenome.org/cgi-bin/locus.pl?locus=GYP8)*,* [*LAS17*](http://db.yeastgenome.org/cgi-bin/locus.pl?locus=LAS17)*,* [*LSB5*](http://db.yeastgenome.org/cgi-bin/locus.pl?locus=LSB5)*,* [*MON2*](http://db.yeastgenome.org/cgi-bin/locus.pl?locus=MON2)*,* [*NYV1*](http://db.yeastgenome.org/cgi-bin/locus.pl?locus=NYV1)*,* [*OSH2*](http://db.yeastgenome.org/cgi-bin/locus.pl?locus=OSH2)*,* [*PEP8*](http://db.yeastgenome.org/cgi-bin/locus.pl?locus=PEP8)*,* [*PIL1*](http://db.yeastgenome.org/cgi-bin/locus.pl?locus=PIL1)*,* [*PKH1*](http://db.yeastgenome.org/cgi-bin/locus.pl?locus=PKH1)*,* [*RCR1*](http://db.yeastgenome.org/cgi-bin/locus.pl?locus=RCR1)*,* [*RHO1*](http://db.yeastgenome.org/cgi-bin/locus.pl?locus=RHO1)*,* [*ROM2*](http://db.yeastgenome.org/cgi-bin/locus.pl?locus=ROM2)*,* [*RSN1*](http://db.yeastgenome.org/cgi-bin/locus.pl?locus=RSN1)*,* [*RVS167*](http://db.yeastgenome.org/cgi-bin/locus.pl?locus=RVS167)*,* [*SAC6*](http://db.yeastgenome.org/cgi-bin/locus.pl?locus=SAC6)*,* [*SCD5*](http://db.yeastgenome.org/cgi-bin/locus.pl?locus=SCD5)*,* [*SEC9*](http://db.yeastgenome.org/cgi-bin/locus.pl?locus=SEC9)*,* [*SED4*](http://db.yeastgenome.org/cgi-bin/locus.pl?locus=SED4)*,* [*SED5*](http://db.yeastgenome.org/cgi-bin/locus.pl?locus=SED5)*,* [*SEM1*](http://db.yeastgenome.org/cgi-bin/locus.pl?locus=SEM1)*,* [*SIW14*](http://db.yeastgenome.org/cgi-bin/locus.pl?locus=SIW14)*,* [*SLA2*](http://db.yeastgenome.org/cgi-bin/locus.pl?locus=SLA2)*,* [*SLG1*](http://db.yeastgenome.org/cgi-bin/locus.pl?locus=SLG1)*,* [*SNC1*](http://db.yeastgenome.org/cgi-bin/locus.pl?locus=SNC1)*,* [*SNF7*](http://db.yeastgenome.org/cgi-bin/locus.pl?locus=SNF7)*,* [*SPO14*](http://db.yeastgenome.org/cgi-bin/locus.pl?locus=SPO14)*,* [*SSO1*](http://db.yeastgenome.org/cgi-bin/locus.pl?locus=SSO1)*,* [*TRS31*](http://db.yeastgenome.org/cgi-bin/locus.pl?locus=TRS31)*,* [*TVP23*](http://db.yeastgenome.org/cgi-bin/locus.pl?locus=TVP23)*,* [*VPS21*](http://db.yeastgenome.org/cgi-bin/locus.pl?locus=VPS21)*,* [*VPS29*](http://db.yeastgenome.org/cgi-bin/locus.pl?locus=VPS29)*,* [*VRP1*](http://db.yeastgenome.org/cgi-bin/locus.pl?locus=VRP1)*,* [*WHI2*](http://db.yeastgenome.org/cgi-bin/locus.pl?locus=WHI2)*,* [*YIP3*](http://db.yeastgenome.org/cgi-bin/locus.pl?locus=YIP3)*,* [*YKT6*](http://db.yeastgenome.org/cgi-bin/locus.pl?locus=YKT6)*,* [*YPK1*](http://db.yeastgenome.org/cgi-bin/locus.pl?locus=YPK1) |
| Membrane invagination | 23 out of 498 genes, 4.6% | 4.62e-6 | [*AKL1*](http://db.yeastgenome.org/cgi-bin/locus.pl?locus=AKL1)*,* [*ARK1*](http://db.yeastgenome.org/cgi-bin/locus.pl?locus=ARK1)*,* [*EHD3*](http://db.yeastgenome.org/cgi-bin/locus.pl?locus=EHD3)*,* [*ENT2*](http://db.yeastgenome.org/cgi-bin/locus.pl?locus=ENT2)*,* [*LAS17*](http://db.yeastgenome.org/cgi-bin/locus.pl?locus=LAS17)*,* [*LSB5*](http://db.yeastgenome.org/cgi-bin/locus.pl?locus=LSB5)*,* [*MON2*](http://db.yeastgenome.org/cgi-bin/locus.pl?locus=MON2)*,* [*OSH2*](http://db.yeastgenome.org/cgi-bin/locus.pl?locus=OSH2)*,* [*PIL1*](http://db.yeastgenome.org/cgi-bin/locus.pl?locus=PIL1)*,* [*PKH1*](http://db.yeastgenome.org/cgi-bin/locus.pl?locus=PKH1)*,* [*RHO1*](http://db.yeastgenome.org/cgi-bin/locus.pl?locus=RHO1)*,* [*ROM2*](http://db.yeastgenome.org/cgi-bin/locus.pl?locus=ROM2)*,* [*RVS167*](http://db.yeastgenome.org/cgi-bin/locus.pl?locus=RVS167)*,* [*SAC6*](http://db.yeastgenome.org/cgi-bin/locus.pl?locus=SAC6)*,* [*SCD5*](http://db.yeastgenome.org/cgi-bin/locus.pl?locus=SCD5)*,* [*SIW14*](http://db.yeastgenome.org/cgi-bin/locus.pl?locus=SIW14)*,* [*SLA2*](http://db.yeastgenome.org/cgi-bin/locus.pl?locus=SLA2)*,* [*SLG1*](http://db.yeastgenome.org/cgi-bin/locus.pl?locus=SLG1)*,* [*SNC1*](http://db.yeastgenome.org/cgi-bin/locus.pl?locus=SNC1)*,* [*VPS21*](http://db.yeastgenome.org/cgi-bin/locus.pl?locus=VPS21)*,* [*VRP1*](http://db.yeastgenome.org/cgi-bin/locus.pl?locus=VRP1)*,* [*WHI2*](http://db.yeastgenome.org/cgi-bin/locus.pl?locus=WHI2)*,* [*YPK1*](http://db.yeastgenome.org/cgi-bin/locus.pl?locus=YPK1) |
| Endocytosis | 23 out of 498 genes, 4.6% | 4.62e-6 | [*AKL1*](http://db.yeastgenome.org/cgi-bin/locus.pl?locus=AKL1)*,* [*ARK1*](http://db.yeastgenome.org/cgi-bin/locus.pl?locus=ARK1)*,* [*EHD3*](http://db.yeastgenome.org/cgi-bin/locus.pl?locus=EHD3)*,* [*ENT2*](http://db.yeastgenome.org/cgi-bin/locus.pl?locus=ENT2)*,* [*LAS17*](http://db.yeastgenome.org/cgi-bin/locus.pl?locus=LAS17)*,* [*LSB5*](http://db.yeastgenome.org/cgi-bin/locus.pl?locus=LSB5)*,* [*MON2*](http://db.yeastgenome.org/cgi-bin/locus.pl?locus=MON2)*,* [*OSH2*](http://db.yeastgenome.org/cgi-bin/locus.pl?locus=OSH2)*,* [*PIL1*](http://db.yeastgenome.org/cgi-bin/locus.pl?locus=PIL1)*,* [*PKH1*](http://db.yeastgenome.org/cgi-bin/locus.pl?locus=PKH1)*,* [*RHO1*](http://db.yeastgenome.org/cgi-bin/locus.pl?locus=RHO1)*,* [*ROM2*](http://db.yeastgenome.org/cgi-bin/locus.pl?locus=ROM2)*,* [*RVS167*](http://db.yeastgenome.org/cgi-bin/locus.pl?locus=RVS167)*,* [*SAC6*](http://db.yeastgenome.org/cgi-bin/locus.pl?locus=SAC6)*,* [*SCD5*](http://db.yeastgenome.org/cgi-bin/locus.pl?locus=SCD5)*,* [*SIW14*](http://db.yeastgenome.org/cgi-bin/locus.pl?locus=SIW14)*,* [*SLA2*](http://db.yeastgenome.org/cgi-bin/locus.pl?locus=SLA2)*,* [*SLG1*](http://db.yeastgenome.org/cgi-bin/locus.pl?locus=SLG1)*,* [*SNC1*](http://db.yeastgenome.org/cgi-bin/locus.pl?locus=SNC1)*,* [*VPS21*](http://db.yeastgenome.org/cgi-bin/locus.pl?locus=VPS21)*,* [*VRP1*](http://db.yeastgenome.org/cgi-bin/locus.pl?locus=VRP1)*,* [*WHI2*](http://db.yeastgenome.org/cgi-bin/locus.pl?locus=WHI2)*,* [*YPK1*](http://db.yeastgenome.org/cgi-bin/locus.pl?locus=YPK1) |
| Localization | 115 out of 498 genes, 23.1% | 1.18e-5 | [*ABF2*](http://db.yeastgenome.org/cgi-bin/locus.pl?locus=ABF2)*,* [*ADY2*](http://db.yeastgenome.org/cgi-bin/locus.pl?locus=ADY2)*,* [*AFG3*](http://db.yeastgenome.org/cgi-bin/locus.pl?locus=AFG3)*,* [*AGP3*](http://db.yeastgenome.org/cgi-bin/locus.pl?locus=AGP3)*,* [*AKL1*](http://db.yeastgenome.org/cgi-bin/locus.pl?locus=AKL1)*,* [*APL2*](http://db.yeastgenome.org/cgi-bin/locus.pl?locus=APL2)*,* [*APL4*](http://db.yeastgenome.org/cgi-bin/locus.pl?locus=APL4)*,* [*APM2*](http://db.yeastgenome.org/cgi-bin/locus.pl?locus=APM2)*,* [*APS2*](http://db.yeastgenome.org/cgi-bin/locus.pl?locus=APS2)*,* [*ARC15*](http://db.yeastgenome.org/cgi-bin/locus.pl?locus=ARC15)*,* [*ARC40*](http://db.yeastgenome.org/cgi-bin/locus.pl?locus=ARC40)*,* [*ARK1*](http://db.yeastgenome.org/cgi-bin/locus.pl?locus=ARK1)*,* [*ATG18*](http://db.yeastgenome.org/cgi-bin/locus.pl?locus=ATG18)*,* [*ATG20*](http://db.yeastgenome.org/cgi-bin/locus.pl?locus=ATG20)*,* [*ATG4*](http://db.yeastgenome.org/cgi-bin/locus.pl?locus=ATG4)*,* [*ATG5*](http://db.yeastgenome.org/cgi-bin/locus.pl?locus=ATG5)*,* [*AVT3*](http://db.yeastgenome.org/cgi-bin/locus.pl?locus=AVT3)*,* [*BCH1*](http://db.yeastgenome.org/cgi-bin/locus.pl?locus=BCH1)*,* [*BPT1*](http://db.yeastgenome.org/cgi-bin/locus.pl?locus=BPT1)*,* [*BUD7*](http://db.yeastgenome.org/cgi-bin/locus.pl?locus=BUD7)*,* [*CHS5*](http://db.yeastgenome.org/cgi-bin/locus.pl?locus=CHS5)*,* [*COX19*](http://db.yeastgenome.org/cgi-bin/locus.pl?locus=COX19)*,* [*COY1*](http://db.yeastgenome.org/cgi-bin/locus.pl?locus=COY1)*,* [*DID2*](http://db.yeastgenome.org/cgi-bin/locus.pl?locus=DID2)*,* [*EHD3*](http://db.yeastgenome.org/cgi-bin/locus.pl?locus=EHD3)*,* [*ENA2*](http://db.yeastgenome.org/cgi-bin/locus.pl?locus=ENA2)*,* [*ENA5*](http://db.yeastgenome.org/cgi-bin/locus.pl?locus=ENA5)*,* [*ENT2*](http://db.yeastgenome.org/cgi-bin/locus.pl?locus=ENT2)*,* [*ENT5*](http://db.yeastgenome.org/cgi-bin/locus.pl?locus=ENT5)*,* [*ERP6*](http://db.yeastgenome.org/cgi-bin/locus.pl?locus=ERP6)*,* [*ERV1*](http://db.yeastgenome.org/cgi-bin/locus.pl?locus=ERV1)*,* [*ERV41*](http://db.yeastgenome.org/cgi-bin/locus.pl?locus=ERV41)*,* [*FLC1*](http://db.yeastgenome.org/cgi-bin/locus.pl?locus=FLC1)*,* [*FLC2*](http://db.yeastgenome.org/cgi-bin/locus.pl?locus=FLC2)*,* [*FRE4*](http://db.yeastgenome.org/cgi-bin/locus.pl?locus=FRE4)*,* [*GYP8*](http://db.yeastgenome.org/cgi-bin/locus.pl?locus=GYP8)*,* [*HXT9*](http://db.yeastgenome.org/cgi-bin/locus.pl?locus=HXT9)*,* [*IMP2*](http://db.yeastgenome.org/cgi-bin/locus.pl?locus=IMP2)*,* [*IVY1*](http://db.yeastgenome.org/cgi-bin/locus.pl?locus=IVY1)*,* [*JEN1*](http://db.yeastgenome.org/cgi-bin/locus.pl?locus=JEN1)*,* [*LAS17*](http://db.yeastgenome.org/cgi-bin/locus.pl?locus=LAS17)*,* [*LSB5*](http://db.yeastgenome.org/cgi-bin/locus.pl?locus=LSB5)*,* [*MAS2*](http://db.yeastgenome.org/cgi-bin/locus.pl?locus=MAS2)*,* [*MDM12*](http://db.yeastgenome.org/cgi-bin/locus.pl?locus=MDM12)*,* [*MDR1*](http://db.yeastgenome.org/cgi-bin/locus.pl?locus=MDR1)*,* [*MFT1*](http://db.yeastgenome.org/cgi-bin/locus.pl?locus=MFT1)*,* [*MMP1*](http://db.yeastgenome.org/cgi-bin/locus.pl?locus=MMP1)*,* [*MON2*](http://db.yeastgenome.org/cgi-bin/locus.pl?locus=MON2)*,* [*MSN5*](http://db.yeastgenome.org/cgi-bin/locus.pl?locus=MSN5)*,* [*MTM1*](http://db.yeastgenome.org/cgi-bin/locus.pl?locus=MTM1)*,* [*NAB2*](http://db.yeastgenome.org/cgi-bin/locus.pl?locus=NAB2)*,* [*NYV1*](http://db.yeastgenome.org/cgi-bin/locus.pl?locus=NYV1)*,* [*ORT1*](http://db.yeastgenome.org/cgi-bin/locus.pl?locus=ORT1)*,* [*OSH2*](http://db.yeastgenome.org/cgi-bin/locus.pl?locus=OSH2)*,* [*PAP1*](http://db.yeastgenome.org/cgi-bin/locus.pl?locus=PAP1)*,* [*PBS2*](http://db.yeastgenome.org/cgi-bin/locus.pl?locus=PBS2)*,* [*PCA1*](http://db.yeastgenome.org/cgi-bin/locus.pl?locus=PCA1)*,* [*PCP1*](http://db.yeastgenome.org/cgi-bin/locus.pl?locus=PCP1)*,* [*PDR10*](http://db.yeastgenome.org/cgi-bin/locus.pl?locus=PDR10)*,* [*PDR15*](http://db.yeastgenome.org/cgi-bin/locus.pl?locus=PDR15)*,* [*PDR16*](http://db.yeastgenome.org/cgi-bin/locus.pl?locus=PDR16)*,* [*PEP8*](http://db.yeastgenome.org/cgi-bin/locus.pl?locus=PEP8)*,* [*PET8*](http://db.yeastgenome.org/cgi-bin/locus.pl?locus=PET8)*,* [*PEX14*](http://db.yeastgenome.org/cgi-bin/locus.pl?locus=PEX14)*,* [*PHO89*](http://db.yeastgenome.org/cgi-bin/locus.pl?locus=PHO89)*,* [*PIL1*](http://db.yeastgenome.org/cgi-bin/locus.pl?locus=PIL1)*,* [*PKH1*](http://db.yeastgenome.org/cgi-bin/locus.pl?locus=PKH1)*,* [*PMR1*](http://db.yeastgenome.org/cgi-bin/locus.pl?locus=PMR1)*,* [*RCR1*](http://db.yeastgenome.org/cgi-bin/locus.pl?locus=RCR1)*,* [*RHO1*](http://db.yeastgenome.org/cgi-bin/locus.pl?locus=RHO1)*,* [*ROM2*](http://db.yeastgenome.org/cgi-bin/locus.pl?locus=ROM2)*,* [*RSN1*](http://db.yeastgenome.org/cgi-bin/locus.pl?locus=RSN1)*,* [*RTG1*](http://db.yeastgenome.org/cgi-bin/locus.pl?locus=RTG1)*,* [*RVS167*](http://db.yeastgenome.org/cgi-bin/locus.pl?locus=RVS167)*,* [*SAC6*](http://db.yeastgenome.org/cgi-bin/locus.pl?locus=SAC6)*,* [*SCD5*](http://db.yeastgenome.org/cgi-bin/locus.pl?locus=SCD5)*,* [*SEC9*](http://db.yeastgenome.org/cgi-bin/locus.pl?locus=SEC9)*,* [*SED4*](http://db.yeastgenome.org/cgi-bin/locus.pl?locus=SED4)*,* [*SED5*](http://db.yeastgenome.org/cgi-bin/locus.pl?locus=SED5)*,* [*SEM1*](http://db.yeastgenome.org/cgi-bin/locus.pl?locus=SEM1)*,* [*SFH5*](http://db.yeastgenome.org/cgi-bin/locus.pl?locus=SFH5)*,* [*SIW14*](http://db.yeastgenome.org/cgi-bin/locus.pl?locus=SIW14)*,* [*SLA2*](http://db.yeastgenome.org/cgi-bin/locus.pl?locus=SLA2)*,* [*SLG1*](http://db.yeastgenome.org/cgi-bin/locus.pl?locus=SLG1)*,* [*SMF1*](http://db.yeastgenome.org/cgi-bin/locus.pl?locus=SMF1)*,* [*SNC1*](http://db.yeastgenome.org/cgi-bin/locus.pl?locus=SNC1)*,* [*SNF7*](http://db.yeastgenome.org/cgi-bin/locus.pl?locus=SNF7)*,* [*SPO14*](http://db.yeastgenome.org/cgi-bin/locus.pl?locus=SPO14)*,* [*SSL2*](http://db.yeastgenome.org/cgi-bin/locus.pl?locus=SSL2)*,* [*SSO1*](http://db.yeastgenome.org/cgi-bin/locus.pl?locus=SSO1)*,* [*SSP120*](http://db.yeastgenome.org/cgi-bin/locus.pl?locus=SSP120)*,* [*STF1*](http://db.yeastgenome.org/cgi-bin/locus.pl?locus=STF1)*,* [*SUT2*](http://db.yeastgenome.org/cgi-bin/locus.pl?locus=SUT2)*,* [*TIM9*](http://db.yeastgenome.org/cgi-bin/locus.pl?locus=TIM9)*,* [*TPO5*](http://db.yeastgenome.org/cgi-bin/locus.pl?locus=TPO5)*,* [*TRS31*](http://db.yeastgenome.org/cgi-bin/locus.pl?locus=TRS31)*,* [*TVP23*](http://db.yeastgenome.org/cgi-bin/locus.pl?locus=TVP23)*,* [*URE2*](http://db.yeastgenome.org/cgi-bin/locus.pl?locus=URE2)*,* [*VPS21*](http://db.yeastgenome.org/cgi-bin/locus.pl?locus=VPS21)*,* [*VPS29*](http://db.yeastgenome.org/cgi-bin/locus.pl?locus=VPS29)*,* [*VPS30*](http://db.yeastgenome.org/cgi-bin/locus.pl?locus=VPS30)*,* [*VPS62*](http://db.yeastgenome.org/cgi-bin/locus.pl?locus=VPS62)*,* [*VPS70*](http://db.yeastgenome.org/cgi-bin/locus.pl?locus=VPS70)*,* [*VRP1*](http://db.yeastgenome.org/cgi-bin/locus.pl?locus=VRP1)*,* [*WHI2*](http://db.yeastgenome.org/cgi-bin/locus.pl?locus=WHI2)*,* [*YIA6*](http://db.yeastgenome.org/cgi-bin/locus.pl?locus=YIA6)*,* [*YIL166C*](http://db.yeastgenome.org/cgi-bin/locus.pl?locus=YIL166C)*,* [*YIP3*](http://db.yeastgenome.org/cgi-bin/locus.pl?locus=YIP3)*,* [*YKT6*](http://db.yeastgenome.org/cgi-bin/locus.pl?locus=YKT6)*,* [*YMD8*](http://db.yeastgenome.org/cgi-bin/locus.pl?locus=YMD8)*,* [*YMR166C*](http://db.yeastgenome.org/cgi-bin/locus.pl?locus=YMR166C)*,* [*YOL163W*](http://db.yeastgenome.org/cgi-bin/locus.pl?locus=YOL163W)*,* [*YOR1*](http://db.yeastgenome.org/cgi-bin/locus.pl?locus=YOR1)*,* [*YPK1*](http://db.yeastgenome.org/cgi-bin/locus.pl?locus=YPK1)*,* [*ZDS1*](http://db.yeastgenome.org/cgi-bin/locus.pl?locus=ZDS1) |
| Transport | 109 out of 498 genes, 21.9% | 1.31e-5 | [*ADY2*](http://db.yeastgenome.org/cgi-bin/locus.pl?locus=ADY2)*,* [*AFG3*](http://db.yeastgenome.org/cgi-bin/locus.pl?locus=AFG3)*,* [*AGP3*](http://db.yeastgenome.org/cgi-bin/locus.pl?locus=AGP3)*,* [*AKL1*](http://db.yeastgenome.org/cgi-bin/locus.pl?locus=AKL1)*,* [*APL2*](http://db.yeastgenome.org/cgi-bin/locus.pl?locus=APL2)*,* [*APL4*](http://db.yeastgenome.org/cgi-bin/locus.pl?locus=APL4)*,* [*APM2*](http://db.yeastgenome.org/cgi-bin/locus.pl?locus=APM2)*,* [*APS2*](http://db.yeastgenome.org/cgi-bin/locus.pl?locus=APS2)*,* [*ARK1*](http://db.yeastgenome.org/cgi-bin/locus.pl?locus=ARK1)*,* [*ATG18*](http://db.yeastgenome.org/cgi-bin/locus.pl?locus=ATG18)*,* [*ATG20*](http://db.yeastgenome.org/cgi-bin/locus.pl?locus=ATG20)*,* [*ATG4*](http://db.yeastgenome.org/cgi-bin/locus.pl?locus=ATG4)*,* [*ATG5*](http://db.yeastgenome.org/cgi-bin/locus.pl?locus=ATG5)*,* [*AVT3*](http://db.yeastgenome.org/cgi-bin/locus.pl?locus=AVT3)*,* [*BCH1*](http://db.yeastgenome.org/cgi-bin/locus.pl?locus=BCH1)*,* [*BPT1*](http://db.yeastgenome.org/cgi-bin/locus.pl?locus=BPT1)*,* [*BUD7*](http://db.yeastgenome.org/cgi-bin/locus.pl?locus=BUD7)*,* [*CHS5*](http://db.yeastgenome.org/cgi-bin/locus.pl?locus=CHS5)*,* [*COX19*](http://db.yeastgenome.org/cgi-bin/locus.pl?locus=COX19)*,* [*COY1*](http://db.yeastgenome.org/cgi-bin/locus.pl?locus=COY1)*,* [*DID2*](http://db.yeastgenome.org/cgi-bin/locus.pl?locus=DID2)*,* [*EHD3*](http://db.yeastgenome.org/cgi-bin/locus.pl?locus=EHD3)*,* [*ENA2*](http://db.yeastgenome.org/cgi-bin/locus.pl?locus=ENA2)*,* [*ENA5*](http://db.yeastgenome.org/cgi-bin/locus.pl?locus=ENA5)*,* [*ENT2*](http://db.yeastgenome.org/cgi-bin/locus.pl?locus=ENT2)*,* [*ENT5*](http://db.yeastgenome.org/cgi-bin/locus.pl?locus=ENT5)*,* [*ERP6*](http://db.yeastgenome.org/cgi-bin/locus.pl?locus=ERP6)*,* [*ERV1*](http://db.yeastgenome.org/cgi-bin/locus.pl?locus=ERV1)*,* [*ERV41*](http://db.yeastgenome.org/cgi-bin/locus.pl?locus=ERV41)*,* [*FLC1*](http://db.yeastgenome.org/cgi-bin/locus.pl?locus=FLC1)*,* [*FLC2*](http://db.yeastgenome.org/cgi-bin/locus.pl?locus=FLC2)*,* [*FRE4*](http://db.yeastgenome.org/cgi-bin/locus.pl?locus=FRE4)*,* [*GYP8*](http://db.yeastgenome.org/cgi-bin/locus.pl?locus=GYP8)*,* [*HXT9*](http://db.yeastgenome.org/cgi-bin/locus.pl?locus=HXT9)*,* [*IMP2*](http://db.yeastgenome.org/cgi-bin/locus.pl?locus=IMP2)*,* [*IVY1*](http://db.yeastgenome.org/cgi-bin/locus.pl?locus=IVY1)*,* [*JEN1*](http://db.yeastgenome.org/cgi-bin/locus.pl?locus=JEN1)*,* [*LAS17*](http://db.yeastgenome.org/cgi-bin/locus.pl?locus=LAS17)*,* [*LSB5*](http://db.yeastgenome.org/cgi-bin/locus.pl?locus=LSB5)*,* [*MAS2*](http://db.yeastgenome.org/cgi-bin/locus.pl?locus=MAS2)*,* [*MDR1*](http://db.yeastgenome.org/cgi-bin/locus.pl?locus=MDR1)*,* [*MFT1*](http://db.yeastgenome.org/cgi-bin/locus.pl?locus=MFT1)*,* [*MMP1*](http://db.yeastgenome.org/cgi-bin/locus.pl?locus=MMP1)*,* [*MON2*](http://db.yeastgenome.org/cgi-bin/locus.pl?locus=MON2)*,* [*MSN5*](http://db.yeastgenome.org/cgi-bin/locus.pl?locus=MSN5)*,* [*MTM1*](http://db.yeastgenome.org/cgi-bin/locus.pl?locus=MTM1)*,* [*NAB2*](http://db.yeastgenome.org/cgi-bin/locus.pl?locus=NAB2)*,* [*NYV1*](http://db.yeastgenome.org/cgi-bin/locus.pl?locus=NYV1)*,* [*ORT1*](http://db.yeastgenome.org/cgi-bin/locus.pl?locus=ORT1)*,* [*OSH2*](http://db.yeastgenome.org/cgi-bin/locus.pl?locus=OSH2)*,* [*PAP1*](http://db.yeastgenome.org/cgi-bin/locus.pl?locus=PAP1)*,* [*PBS2*](http://db.yeastgenome.org/cgi-bin/locus.pl?locus=PBS2)*,* [*PCA1*](http://db.yeastgenome.org/cgi-bin/locus.pl?locus=PCA1)*,* [*PCP1*](http://db.yeastgenome.org/cgi-bin/locus.pl?locus=PCP1)*,* [*PDR10*](http://db.yeastgenome.org/cgi-bin/locus.pl?locus=PDR10)*,* [*PDR15*](http://db.yeastgenome.org/cgi-bin/locus.pl?locus=PDR15)*,* [*PDR16*](http://db.yeastgenome.org/cgi-bin/locus.pl?locus=PDR16)*,* [*PEP8*](http://db.yeastgenome.org/cgi-bin/locus.pl?locus=PEP8)*,* [*PET8*](http://db.yeastgenome.org/cgi-bin/locus.pl?locus=PET8)*,* [*PEX14*](http://db.yeastgenome.org/cgi-bin/locus.pl?locus=PEX14)*,* [*PHO89*](http://db.yeastgenome.org/cgi-bin/locus.pl?locus=PHO89)*,* [*PIL1*](http://db.yeastgenome.org/cgi-bin/locus.pl?locus=PIL1)*,* [*PKH1*](http://db.yeastgenome.org/cgi-bin/locus.pl?locus=PKH1)*,* [*PMR1*](http://db.yeastgenome.org/cgi-bin/locus.pl?locus=PMR1)*,* [*RCR1*](http://db.yeastgenome.org/cgi-bin/locus.pl?locus=RCR1)*,* [*RHO1*](http://db.yeastgenome.org/cgi-bin/locus.pl?locus=RHO1)*,* [*ROM2*](http://db.yeastgenome.org/cgi-bin/locus.pl?locus=ROM2)*,* [*RSN1*](http://db.yeastgenome.org/cgi-bin/locus.pl?locus=RSN1)*,* [*RVS167*](http://db.yeastgenome.org/cgi-bin/locus.pl?locus=RVS167)*,* [*SAC6*](http://db.yeastgenome.org/cgi-bin/locus.pl?locus=SAC6)*,* [*SCD5*](http://db.yeastgenome.org/cgi-bin/locus.pl?locus=SCD5)*,* [*SEC9*](http://db.yeastgenome.org/cgi-bin/locus.pl?locus=SEC9)*,* [*SED4*](http://db.yeastgenome.org/cgi-bin/locus.pl?locus=SED4)*,* [*SED5*](http://db.yeastgenome.org/cgi-bin/locus.pl?locus=SED5)*,* [*SEM1*](http://db.yeastgenome.org/cgi-bin/locus.pl?locus=SEM1)*,* [*SFH5*](http://db.yeastgenome.org/cgi-bin/locus.pl?locus=SFH5)*,* [*SIW14*](http://db.yeastgenome.org/cgi-bin/locus.pl?locus=SIW14)*,* [*SLA2*](http://db.yeastgenome.org/cgi-bin/locus.pl?locus=SLA2)*,* [*SLG1*](http://db.yeastgenome.org/cgi-bin/locus.pl?locus=SLG1)*,* [*SMF1*](http://db.yeastgenome.org/cgi-bin/locus.pl?locus=SMF1)*,* [*SNC1*](http://db.yeastgenome.org/cgi-bin/locus.pl?locus=SNC1)*,* [*SNF7*](http://db.yeastgenome.org/cgi-bin/locus.pl?locus=SNF7)*,* [*SPO14*](http://db.yeastgenome.org/cgi-bin/locus.pl?locus=SPO14)*,* [*SSL2*](http://db.yeastgenome.org/cgi-bin/locus.pl?locus=SSL2)*,* [*SSO1*](http://db.yeastgenome.org/cgi-bin/locus.pl?locus=SSO1)*,* [*STF1*](http://db.yeastgenome.org/cgi-bin/locus.pl?locus=STF1)*,* [*SUT2*](http://db.yeastgenome.org/cgi-bin/locus.pl?locus=SUT2)*,* [*TIM9*](http://db.yeastgenome.org/cgi-bin/locus.pl?locus=TIM9)*,* [*TPO5*](http://db.yeastgenome.org/cgi-bin/locus.pl?locus=TPO5)*,* [*TRS31*](http://db.yeastgenome.org/cgi-bin/locus.pl?locus=TRS31)*,* [*TVP23*](http://db.yeastgenome.org/cgi-bin/locus.pl?locus=TVP23)*,* [*URE2*](http://db.yeastgenome.org/cgi-bin/locus.pl?locus=URE2)*,* [*VPS21*](http://db.yeastgenome.org/cgi-bin/locus.pl?locus=VPS21)*,* [*VPS29*](http://db.yeastgenome.org/cgi-bin/locus.pl?locus=VPS29)*,* [*VPS30*](http://db.yeastgenome.org/cgi-bin/locus.pl?locus=VPS30)*,* [*VPS62*](http://db.yeastgenome.org/cgi-bin/locus.pl?locus=VPS62)*,* [*VPS70*](http://db.yeastgenome.org/cgi-bin/locus.pl?locus=VPS70)*,* [*VRP1*](http://db.yeastgenome.org/cgi-bin/locus.pl?locus=VRP1)*,* [*WHI2*](http://db.yeastgenome.org/cgi-bin/locus.pl?locus=WHI2)*,* [*YIA6*](http://db.yeastgenome.org/cgi-bin/locus.pl?locus=YIA6)*,* [*YIL166C*](http://db.yeastgenome.org/cgi-bin/locus.pl?locus=YIL166C)*,* [*YIP3*](http://db.yeastgenome.org/cgi-bin/locus.pl?locus=YIP3)*,* [*YKT6*](http://db.yeastgenome.org/cgi-bin/locus.pl?locus=YKT6)*,* [*YMD8*](http://db.yeastgenome.org/cgi-bin/locus.pl?locus=YMD8)*,* [*YMR166C*](http://db.yeastgenome.org/cgi-bin/locus.pl?locus=YMR166C)*,* [*YOL163W*](http://db.yeastgenome.org/cgi-bin/locus.pl?locus=YOL163W)*,* [*YOR1*](http://db.yeastgenome.org/cgi-bin/locus.pl?locus=YOR1)*,* [*YPK1*](http://db.yeastgenome.org/cgi-bin/locus.pl?locus=YPK1)*,* [*ZDS1*](http://db.yeastgenome.org/cgi-bin/locus.pl?locus=ZDS1) |
| Proteolysis involved in cellular protein catabolic process | 31 out of 498 genes, 21.9% | 1.45e-5 | [*ADD66*](http://db.yeastgenome.org/cgi-bin/locus.pl?locus=ADD66)*,* [*APC11*](http://db.yeastgenome.org/cgi-bin/locus.pl?locus=APC11)*,* [*DOA1*](http://db.yeastgenome.org/cgi-bin/locus.pl?locus=DOA1)*,* [*DSK2*](http://db.yeastgenome.org/cgi-bin/locus.pl?locus=DSK2)*,* [*HRD3*](http://db.yeastgenome.org/cgi-bin/locus.pl?locus=HRD3)*,* [*MET30*](http://db.yeastgenome.org/cgi-bin/locus.pl?locus=MET30)*,* [*NAS2*](http://db.yeastgenome.org/cgi-bin/locus.pl?locus=NAS2)*,* [*PNG1*](http://db.yeastgenome.org/cgi-bin/locus.pl?locus=PNG1)*,* [*PRE4*](http://db.yeastgenome.org/cgi-bin/locus.pl?locus=PRE4)*,* [*PRE5*](http://db.yeastgenome.org/cgi-bin/locus.pl?locus=PRE5)*,* [*PRE7*](http://db.yeastgenome.org/cgi-bin/locus.pl?locus=PRE7)*,* [*PUP1*](http://db.yeastgenome.org/cgi-bin/locus.pl?locus=PUP1)*,* [*PUP2*](http://db.yeastgenome.org/cgi-bin/locus.pl?locus=PUP2)*,* [*PUP3*](http://db.yeastgenome.org/cgi-bin/locus.pl?locus=PUP3)*,* [*QRI8*](http://db.yeastgenome.org/cgi-bin/locus.pl?locus=QRI8)*,* [*RPN1*](http://db.yeastgenome.org/cgi-bin/locus.pl?locus=RPN1)*,* [*RPN10*](http://db.yeastgenome.org/cgi-bin/locus.pl?locus=RPN10)*,* [*RPN14*](http://db.yeastgenome.org/cgi-bin/locus.pl?locus=RPN14)*,* [*RPT1*](http://db.yeastgenome.org/cgi-bin/locus.pl?locus=RPT1)*,* [*RTT101*](http://db.yeastgenome.org/cgi-bin/locus.pl?locus=RTT101)*,* [*SCL1*](http://db.yeastgenome.org/cgi-bin/locus.pl?locus=SCL1)*,* [*SEM1*](http://db.yeastgenome.org/cgi-bin/locus.pl?locus=SEM1)*,* [*SHP1*](http://db.yeastgenome.org/cgi-bin/locus.pl?locus=SHP1)*,* [*SKP1*](http://db.yeastgenome.org/cgi-bin/locus.pl?locus=SKP1)*,* [*SNF7*](http://db.yeastgenome.org/cgi-bin/locus.pl?locus=SNF7)*,* [*STS1*](http://db.yeastgenome.org/cgi-bin/locus.pl?locus=STS1)*,* [*SWM1*](http://db.yeastgenome.org/cgi-bin/locus.pl?locus=SWM1)*,* [*TUL1*](http://db.yeastgenome.org/cgi-bin/locus.pl?locus=TUL1)*,* [*UFO1*](http://db.yeastgenome.org/cgi-bin/locus.pl?locus=UFO1)*,* [*VID28*](http://db.yeastgenome.org/cgi-bin/locus.pl?locus=VID28)*,* [*YDR306C*](http://db.yeastgenome.org/cgi-bin/locus.pl?locus=YDR306C) |
| Establishment of localization | 110 out of 498 genes, 22.1% | 2.00e-5 | [*ADY2*](http://db.yeastgenome.org/cgi-bin/locus.pl?locus=ADY2)*,* [*AFG3*](http://db.yeastgenome.org/cgi-bin/locus.pl?locus=AFG3)*,* [*AGP3*](http://db.yeastgenome.org/cgi-bin/locus.pl?locus=AGP3)*,* [*AKL1*](http://db.yeastgenome.org/cgi-bin/locus.pl?locus=AKL1)*,* [*APL2*](http://db.yeastgenome.org/cgi-bin/locus.pl?locus=APL2)*,* [*APL4*](http://db.yeastgenome.org/cgi-bin/locus.pl?locus=APL4)*,* [*APM2*](http://db.yeastgenome.org/cgi-bin/locus.pl?locus=APM2)*,* [*APS2*](http://db.yeastgenome.org/cgi-bin/locus.pl?locus=APS2)*,* [*ARK1*](http://db.yeastgenome.org/cgi-bin/locus.pl?locus=ARK1)*,* [*ATG18*](http://db.yeastgenome.org/cgi-bin/locus.pl?locus=ATG18)*,* [*ATG20*](http://db.yeastgenome.org/cgi-bin/locus.pl?locus=ATG20)*,* [*ATG4*](http://db.yeastgenome.org/cgi-bin/locus.pl?locus=ATG4)*,* [*ATG5*](http://db.yeastgenome.org/cgi-bin/locus.pl?locus=ATG5)*,* [*AVT3*](http://db.yeastgenome.org/cgi-bin/locus.pl?locus=AVT3)*,* [*BCH1*](http://db.yeastgenome.org/cgi-bin/locus.pl?locus=BCH1)*,* [*BPT1*](http://db.yeastgenome.org/cgi-bin/locus.pl?locus=BPT1)*,* [*BUD7*](http://db.yeastgenome.org/cgi-bin/locus.pl?locus=BUD7)*,* [*CHS5*](http://db.yeastgenome.org/cgi-bin/locus.pl?locus=CHS5)*,* [*COX19*](http://db.yeastgenome.org/cgi-bin/locus.pl?locus=COX19)*,* [*COY1*](http://db.yeastgenome.org/cgi-bin/locus.pl?locus=COY1)*,* [*DID2*](http://db.yeastgenome.org/cgi-bin/locus.pl?locus=DID2)*,* [*EHD3*](http://db.yeastgenome.org/cgi-bin/locus.pl?locus=EHD3)*,* [*ENA2*](http://db.yeastgenome.org/cgi-bin/locus.pl?locus=ENA2)*,* [*ENA5*](http://db.yeastgenome.org/cgi-bin/locus.pl?locus=ENA5)*,* [*ENT2*](http://db.yeastgenome.org/cgi-bin/locus.pl?locus=ENT2)*,* [*ENT5*](http://db.yeastgenome.org/cgi-bin/locus.pl?locus=ENT5)*,* [*ERP6*](http://db.yeastgenome.org/cgi-bin/locus.pl?locus=ERP6)*,* [*ERV1*](http://db.yeastgenome.org/cgi-bin/locus.pl?locus=ERV1)*,* [*ERV41*](http://db.yeastgenome.org/cgi-bin/locus.pl?locus=ERV41)*,* [*FLC1*](http://db.yeastgenome.org/cgi-bin/locus.pl?locus=FLC1)*,* [*FLC2*](http://db.yeastgenome.org/cgi-bin/locus.pl?locus=FLC2)*,* [*FRE4*](http://db.yeastgenome.org/cgi-bin/locus.pl?locus=FRE4)*,* [*GYP8*](http://db.yeastgenome.org/cgi-bin/locus.pl?locus=GYP8)*,* [*HXT9*](http://db.yeastgenome.org/cgi-bin/locus.pl?locus=HXT9)*,* [*IMP2*](http://db.yeastgenome.org/cgi-bin/locus.pl?locus=IMP2)*,* [*IVY1*](http://db.yeastgenome.org/cgi-bin/locus.pl?locus=IVY1)*,* [*JEN1*](http://db.yeastgenome.org/cgi-bin/locus.pl?locus=JEN1)*,* [*LAS17*](http://db.yeastgenome.org/cgi-bin/locus.pl?locus=LAS17)*,* [*LSB5*](http://db.yeastgenome.org/cgi-bin/locus.pl?locus=LSB5)*,* [*MAS2*](http://db.yeastgenome.org/cgi-bin/locus.pl?locus=MAS2)*,* [*MDR1*](http://db.yeastgenome.org/cgi-bin/locus.pl?locus=MDR1)*,* [*MFT1*](http://db.yeastgenome.org/cgi-bin/locus.pl?locus=MFT1)*,* [*MMP1*](http://db.yeastgenome.org/cgi-bin/locus.pl?locus=MMP1)*,* [*MON2*](http://db.yeastgenome.org/cgi-bin/locus.pl?locus=MON2)*,* [*MSN5*](http://db.yeastgenome.org/cgi-bin/locus.pl?locus=MSN5)*,* [*MTM1*](http://db.yeastgenome.org/cgi-bin/locus.pl?locus=MTM1)*,* [*NAB2*](http://db.yeastgenome.org/cgi-bin/locus.pl?locus=NAB2)*,* [*NYV1*](http://db.yeastgenome.org/cgi-bin/locus.pl?locus=NYV1)*,* [*ORT1*](http://db.yeastgenome.org/cgi-bin/locus.pl?locus=ORT1)*,* [*OSH2*](http://db.yeastgenome.org/cgi-bin/locus.pl?locus=OSH2)*,* [*PAP1*](http://db.yeastgenome.org/cgi-bin/locus.pl?locus=PAP1)*,* [*PBS2*](http://db.yeastgenome.org/cgi-bin/locus.pl?locus=PBS2)*,* [*PCA1*](http://db.yeastgenome.org/cgi-bin/locus.pl?locus=PCA1)*,* [*PCP1*](http://db.yeastgenome.org/cgi-bin/locus.pl?locus=PCP1)*,* [*PDR10*](http://db.yeastgenome.org/cgi-bin/locus.pl?locus=PDR10)*,* [*PDR15*](http://db.yeastgenome.org/cgi-bin/locus.pl?locus=PDR15)*,* [*PDR16*](http://db.yeastgenome.org/cgi-bin/locus.pl?locus=PDR16)*,* [*PEP8*](http://db.yeastgenome.org/cgi-bin/locus.pl?locus=PEP8)*,* [*PET8*](http://db.yeastgenome.org/cgi-bin/locus.pl?locus=PET8)*,* [*PEX14*](http://db.yeastgenome.org/cgi-bin/locus.pl?locus=PEX14)*,* [*PHO89*](http://db.yeastgenome.org/cgi-bin/locus.pl?locus=PHO89)*,* [*PIL1*](http://db.yeastgenome.org/cgi-bin/locus.pl?locus=PIL1)*,* [*PKH1*](http://db.yeastgenome.org/cgi-bin/locus.pl?locus=PKH1)*,* [*PMR1*](http://db.yeastgenome.org/cgi-bin/locus.pl?locus=PMR1)*,* [*RCR1*](http://db.yeastgenome.org/cgi-bin/locus.pl?locus=RCR1)*,* [*RHO1*](http://db.yeastgenome.org/cgi-bin/locus.pl?locus=RHO1)*,* [*ROM2*](http://db.yeastgenome.org/cgi-bin/locus.pl?locus=ROM2)*,* [*RSN1*](http://db.yeastgenome.org/cgi-bin/locus.pl?locus=RSN1)*,* [*RVS167*](http://db.yeastgenome.org/cgi-bin/locus.pl?locus=RVS167)*,* [*SAC6*](http://db.yeastgenome.org/cgi-bin/locus.pl?locus=SAC6)*,* [*SCD5*](http://db.yeastgenome.org/cgi-bin/locus.pl?locus=SCD5)*,* [*SEC9*](http://db.yeastgenome.org/cgi-bin/locus.pl?locus=SEC9)*,* [*SED4*](http://db.yeastgenome.org/cgi-bin/locus.pl?locus=SED4)*,* [*SED5*](http://db.yeastgenome.org/cgi-bin/locus.pl?locus=SED5)*,* [*SEM1*](http://db.yeastgenome.org/cgi-bin/locus.pl?locus=SEM1)*,* [*SFH5*](http://db.yeastgenome.org/cgi-bin/locus.pl?locus=SFH5)*,* [*SIW14*](http://db.yeastgenome.org/cgi-bin/locus.pl?locus=SIW14)*,* [*SLA2*](http://db.yeastgenome.org/cgi-bin/locus.pl?locus=SLA2)*,* [*SLG1*](http://db.yeastgenome.org/cgi-bin/locus.pl?locus=SLG1)*,* [*SMF1*](http://db.yeastgenome.org/cgi-bin/locus.pl?locus=SMF1)*,* [*SNC1*](http://db.yeastgenome.org/cgi-bin/locus.pl?locus=SNC1)*,* [*SNF7*](http://db.yeastgenome.org/cgi-bin/locus.pl?locus=SNF7)*,* [*SPO14*](http://db.yeastgenome.org/cgi-bin/locus.pl?locus=SPO14)*,* [*SSL2*](http://db.yeastgenome.org/cgi-bin/locus.pl?locus=SSL2)*,* [*SSO1*](http://db.yeastgenome.org/cgi-bin/locus.pl?locus=SSO1)*,* [*SSP120*](http://db.yeastgenome.org/cgi-bin/locus.pl?locus=SSP120)*,* [*STF1*](http://db.yeastgenome.org/cgi-bin/locus.pl?locus=STF1)*,* [*SUT2*](http://db.yeastgenome.org/cgi-bin/locus.pl?locus=SUT2)*,* [*TIM9*](http://db.yeastgenome.org/cgi-bin/locus.pl?locus=TIM9)*,* [*TPO5*](http://db.yeastgenome.org/cgi-bin/locus.pl?locus=TPO5)*,* [*TRS31*](http://db.yeastgenome.org/cgi-bin/locus.pl?locus=TRS31)*,* [*TVP23*](http://db.yeastgenome.org/cgi-bin/locus.pl?locus=TVP23)*,* [*URE2*](http://db.yeastgenome.org/cgi-bin/locus.pl?locus=URE2)*,* [*VPS21*](http://db.yeastgenome.org/cgi-bin/locus.pl?locus=VPS21)*,* [*VPS29*](http://db.yeastgenome.org/cgi-bin/locus.pl?locus=VPS29)*,* [*VPS30*](http://db.yeastgenome.org/cgi-bin/locus.pl?locus=VPS30)*,* [*VPS62*](http://db.yeastgenome.org/cgi-bin/locus.pl?locus=VPS62)*,* [*VPS70*](http://db.yeastgenome.org/cgi-bin/locus.pl?locus=VPS70)*,* [*VRP1*](http://db.yeastgenome.org/cgi-bin/locus.pl?locus=VRP1)*,* [*WHI2*](http://db.yeastgenome.org/cgi-bin/locus.pl?locus=WHI2)*,* [*YIA6*](http://db.yeastgenome.org/cgi-bin/locus.pl?locus=YIA6)*,* [*YIL166C*](http://db.yeastgenome.org/cgi-bin/locus.pl?locus=YIL166C)*,* [*YIP3*](http://db.yeastgenome.org/cgi-bin/locus.pl?locus=YIP3)*,* [*YKT6*](http://db.yeastgenome.org/cgi-bin/locus.pl?locus=YKT6)*,* [*YMD8*](http://db.yeastgenome.org/cgi-bin/locus.pl?locus=YMD8)*,* [*YMR166C*](http://db.yeastgenome.org/cgi-bin/locus.pl?locus=YMR166C)*,* [*YOL163W*](http://db.yeastgenome.org/cgi-bin/locus.pl?locus=YOL163W)*,* [*YOR1*](http://db.yeastgenome.org/cgi-bin/locus.pl?locus=YOR1)*,* [*YPK1*](http://db.yeastgenome.org/cgi-bin/locus.pl?locus=YPK1)*,* [*ZDS1*](http://db.yeastgenome.org/cgi-bin/locus.pl?locus=ZDS1) |
| Actin cytoskeleton organization and biogenesis | 25 out of 498 genes, 5.0% | 2.72e-5 | [*ACF2*](http://db.yeastgenome.org/cgi-bin/locus.pl?locus=ACF2)*,* [*AKL1*](http://db.yeastgenome.org/cgi-bin/locus.pl?locus=AKL1)*,* [*APP1*](http://db.yeastgenome.org/cgi-bin/locus.pl?locus=APP1)*,* [*ARC15*](http://db.yeastgenome.org/cgi-bin/locus.pl?locus=ARC15)*,* [*ARC40*](http://db.yeastgenome.org/cgi-bin/locus.pl?locus=ARC40)*,* [*ARK1*](http://db.yeastgenome.org/cgi-bin/locus.pl?locus=ARK1)*,* [*AVO2*](http://db.yeastgenome.org/cgi-bin/locus.pl?locus=AVO2)*,* [*BBC1*](http://db.yeastgenome.org/cgi-bin/locus.pl?locus=BBC1)*,* [*ENT2*](http://db.yeastgenome.org/cgi-bin/locus.pl?locus=ENT2)*,* [*HUA1*](http://db.yeastgenome.org/cgi-bin/locus.pl?locus=HUA1)*,* [*HUA2*](http://db.yeastgenome.org/cgi-bin/locus.pl?locus=HUA2)*,* [*LAS17*](http://db.yeastgenome.org/cgi-bin/locus.pl?locus=LAS17)*,* [*LSB5*](http://db.yeastgenome.org/cgi-bin/locus.pl?locus=LSB5)*,* [*PBS2*](http://db.yeastgenome.org/cgi-bin/locus.pl?locus=PBS2)*,* [*RHO1*](http://db.yeastgenome.org/cgi-bin/locus.pl?locus=RHO1)*,* [*ROM2*](http://db.yeastgenome.org/cgi-bin/locus.pl?locus=ROM2)*,* [*SAC6*](http://db.yeastgenome.org/cgi-bin/locus.pl?locus=SAC6)*,* [*SAC7*](http://db.yeastgenome.org/cgi-bin/locus.pl?locus=SAC7)*,* [*SCD5*](http://db.yeastgenome.org/cgi-bin/locus.pl?locus=SCD5)*,* [*SIW14*](http://db.yeastgenome.org/cgi-bin/locus.pl?locus=SIW14)*,* [*SLA2*](http://db.yeastgenome.org/cgi-bin/locus.pl?locus=SLA2)*,* [*SLG1*](http://db.yeastgenome.org/cgi-bin/locus.pl?locus=SLG1)*,* [*SLM1*](http://db.yeastgenome.org/cgi-bin/locus.pl?locus=SLM1)*,* [*VRP1*](http://db.yeastgenome.org/cgi-bin/locus.pl?locus=VRP1)*,* [*WHI2*](http://db.yeastgenome.org/cgi-bin/locus.pl?locus=WHI2) |
| Modification-dependent protein catabolic process | 30 out of 498 genes, 6.0% | 3.96e-5 | [*ADD66*](http://db.yeastgenome.org/cgi-bin/locus.pl?locus=ADD66)*,* [*APC11*](http://db.yeastgenome.org/cgi-bin/locus.pl?locus=APC11)*,* [*DOA1*](http://db.yeastgenome.org/cgi-bin/locus.pl?locus=DOA1)*,* [*DSK2*](http://db.yeastgenome.org/cgi-bin/locus.pl?locus=DSK2)*,* [*HRD3*](http://db.yeastgenome.org/cgi-bin/locus.pl?locus=HRD3)*,* [*MET30*](http://db.yeastgenome.org/cgi-bin/locus.pl?locus=MET30)*,* [*NAS2*](http://db.yeastgenome.org/cgi-bin/locus.pl?locus=NAS2)*,* [*PRE4*](http://db.yeastgenome.org/cgi-bin/locus.pl?locus=PRE4)*,* [*PRE5*](http://db.yeastgenome.org/cgi-bin/locus.pl?locus=PRE5)*,* [*PRE7*](http://db.yeastgenome.org/cgi-bin/locus.pl?locus=PRE7)*,* [*PUP1*](http://db.yeastgenome.org/cgi-bin/locus.pl?locus=PUP1)*,* [*PUP2*](http://db.yeastgenome.org/cgi-bin/locus.pl?locus=PUP2)*,* [*PUP3*](http://db.yeastgenome.org/cgi-bin/locus.pl?locus=PUP3)*,* [*QRI8*](http://db.yeastgenome.org/cgi-bin/locus.pl?locus=QRI8)*,* [*RPN1*](http://db.yeastgenome.org/cgi-bin/locus.pl?locus=RPN1)*,* [*RPN10*](http://db.yeastgenome.org/cgi-bin/locus.pl?locus=RPN10)*,* [*RPN14*](http://db.yeastgenome.org/cgi-bin/locus.pl?locus=RPN14)*,* [*RPT1*](http://db.yeastgenome.org/cgi-bin/locus.pl?locus=RPT1)*,* [*RTT101*](http://db.yeastgenome.org/cgi-bin/locus.pl?locus=RTT101)*,* [*SCL1*](http://db.yeastgenome.org/cgi-bin/locus.pl?locus=SCL1)*,* [*SEM1*](http://db.yeastgenome.org/cgi-bin/locus.pl?locus=SEM1)*,* [*SHP1*](http://db.yeastgenome.org/cgi-bin/locus.pl?locus=SHP1)*,* [*SKP1*](http://db.yeastgenome.org/cgi-bin/locus.pl?locus=SKP1)*,* [*SNF7*](http://db.yeastgenome.org/cgi-bin/locus.pl?locus=SNF7)*,* [*STS1*](http://db.yeastgenome.org/cgi-bin/locus.pl?locus=STS1)*,* [*SWM1*](http://db.yeastgenome.org/cgi-bin/locus.pl?locus=SWM1)*,* [*TUL1*](http://db.yeastgenome.org/cgi-bin/locus.pl?locus=TUL1)*,* [*UFO1*](http://db.yeastgenome.org/cgi-bin/locus.pl?locus=UFO1)*,* [*VID28*](http://db.yeastgenome.org/cgi-bin/locus.pl?locus=VID28)*,* [*YDR306C*](http://db.yeastgenome.org/cgi-bin/locus.pl?locus=YDR306C) |
| Ubiquitin-dependent protein catabolic process | 30 out of 498 genes, 6.0% | 3.96e-5 | [*ADD66*](http://db.yeastgenome.org/cgi-bin/locus.pl?locus=ADD66)*,* [*APC11*](http://db.yeastgenome.org/cgi-bin/locus.pl?locus=APC11)*,* [*DOA1*](http://db.yeastgenome.org/cgi-bin/locus.pl?locus=DOA1)*,* [*DSK2*](http://db.yeastgenome.org/cgi-bin/locus.pl?locus=DSK2)*,* [*HRD3*](http://db.yeastgenome.org/cgi-bin/locus.pl?locus=HRD3)*,* [*MET30*](http://db.yeastgenome.org/cgi-bin/locus.pl?locus=MET30)*,* [*NAS2*](http://db.yeastgenome.org/cgi-bin/locus.pl?locus=NAS2)*,* [*PRE4*](http://db.yeastgenome.org/cgi-bin/locus.pl?locus=PRE4)*,* [*PRE5*](http://db.yeastgenome.org/cgi-bin/locus.pl?locus=PRE5)*,* [*PRE7*](http://db.yeastgenome.org/cgi-bin/locus.pl?locus=PRE7)*,* [*PUP1*](http://db.yeastgenome.org/cgi-bin/locus.pl?locus=PUP1)*,* [*PUP2*](http://db.yeastgenome.org/cgi-bin/locus.pl?locus=PUP2)*,* [*PUP3*](http://db.yeastgenome.org/cgi-bin/locus.pl?locus=PUP3)*,* [*QRI8*](http://db.yeastgenome.org/cgi-bin/locus.pl?locus=QRI8)*,* [*RPN1*](http://db.yeastgenome.org/cgi-bin/locus.pl?locus=RPN1)*,* [*RPN10*](http://db.yeastgenome.org/cgi-bin/locus.pl?locus=RPN10)*,* [*RPN14*](http://db.yeastgenome.org/cgi-bin/locus.pl?locus=RPN14)*,* [*RPT1*](http://db.yeastgenome.org/cgi-bin/locus.pl?locus=RPT1)*,* [*RTT101*](http://db.yeastgenome.org/cgi-bin/locus.pl?locus=RTT101)*,* [*SCL1*](http://db.yeastgenome.org/cgi-bin/locus.pl?locus=SCL1)*,* [*SEM1*](http://db.yeastgenome.org/cgi-bin/locus.pl?locus=SEM1)*,* [*SHP1*](http://db.yeastgenome.org/cgi-bin/locus.pl?locus=SHP1)*,* [*SKP1*](http://db.yeastgenome.org/cgi-bin/locus.pl?locus=SKP1)*,* [*SNF7*](http://db.yeastgenome.org/cgi-bin/locus.pl?locus=SNF7)*,* [*STS1*](http://db.yeastgenome.org/cgi-bin/locus.pl?locus=STS1)*,* [*SWM1*](http://db.yeastgenome.org/cgi-bin/locus.pl?locus=SWM1)*,* [*TUL1*](http://db.yeastgenome.org/cgi-bin/locus.pl?locus=TUL1)*,* [*UFO1*](http://db.yeastgenome.org/cgi-bin/locus.pl?locus=UFO1)*,* [*VID28*](http://db.yeastgenome.org/cgi-bin/locus.pl?locus=VID28)*,* [*YDR306C*](http://db.yeastgenome.org/cgi-bin/locus.pl?locus=YDR306C) |
| Protein catabolic process | 33 out of 498 genes, 6.6% | 4.03e-5 | [*ADD66*](http://db.yeastgenome.org/cgi-bin/locus.pl?locus=ADD66)*,* [*APC11*](http://db.yeastgenome.org/cgi-bin/locus.pl?locus=APC11)*,* [*DOA1*](http://db.yeastgenome.org/cgi-bin/locus.pl?locus=DOA1)*,* [*DSK2*](http://db.yeastgenome.org/cgi-bin/locus.pl?locus=DSK2)*,* [*HRD3*](http://db.yeastgenome.org/cgi-bin/locus.pl?locus=HRD3)*,LAP2,* [*MET30*](http://db.yeastgenome.org/cgi-bin/locus.pl?locus=MET30)*,* [*NAS2*](http://db.yeastgenome.org/cgi-bin/locus.pl?locus=NAS2)*,* [*PNG1*](http://db.yeastgenome.org/cgi-bin/locus.pl?locus=PNG1)*,* [*PRE4*](http://db.yeastgenome.org/cgi-bin/locus.pl?locus=PRE4)*,* [*PRE5*](http://db.yeastgenome.org/cgi-bin/locus.pl?locus=PRE5)*,* [*PRE7*](http://db.yeastgenome.org/cgi-bin/locus.pl?locus=PRE7)*,* [*PUP1*](http://db.yeastgenome.org/cgi-bin/locus.pl?locus=PUP1)*,* [*PUP2*](http://db.yeastgenome.org/cgi-bin/locus.pl?locus=PUP2)*,* [*PUP3*](http://db.yeastgenome.org/cgi-bin/locus.pl?locus=PUP3)*,* [*QRI8*](http://db.yeastgenome.org/cgi-bin/locus.pl?locus=QRI8)*,* [*RPN1*](http://db.yeastgenome.org/cgi-bin/locus.pl?locus=RPN1)*,* [*RPN10*](http://db.yeastgenome.org/cgi-bin/locus.pl?locus=RPN10)*,* [*RPN14*](http://db.yeastgenome.org/cgi-bin/locus.pl?locus=RPN14)*,* [*RPT1*](http://db.yeastgenome.org/cgi-bin/locus.pl?locus=RPT1)*,* [*RTT101*](http://db.yeastgenome.org/cgi-bin/locus.pl?locus=RTT101)*,* [*SCL1*](http://db.yeastgenome.org/cgi-bin/locus.pl?locus=SCL1)*,* [*SEM1*](http://db.yeastgenome.org/cgi-bin/locus.pl?locus=SEM1)*,* [*SHP1*](http://db.yeastgenome.org/cgi-bin/locus.pl?locus=SHP1)*,* [*SKP1*](http://db.yeastgenome.org/cgi-bin/locus.pl?locus=SKP1)*,* [*SNF7*](http://db.yeastgenome.org/cgi-bin/locus.pl?locus=SNF7)*,* [*STS1*](http://db.yeastgenome.org/cgi-bin/locus.pl?locus=STS1)*,* [*SWM1*](http://db.yeastgenome.org/cgi-bin/locus.pl?locus=SWM1)*,* [*TUL1*](http://db.yeastgenome.org/cgi-bin/locus.pl?locus=TUL1)*,* [*UFO1*](http://db.yeastgenome.org/cgi-bin/locus.pl?locus=UFO1)*,* [*VID28*](http://db.yeastgenome.org/cgi-bin/locus.pl?locus=VID28)*,* [*YDR306C*](http://db.yeastgenome.org/cgi-bin/locus.pl?locus=YDR306C)*,* [*YTA7*](http://db.yeastgenome.org/cgi-bin/locus.pl?locus=YTA7) |
| Cellular protein catabolic process | 31 out of 498 genes, 6.2% | 6.12e-5 | [*ADD66*](http://db.yeastgenome.org/cgi-bin/locus.pl?locus=ADD66)*,* [*APC11*](http://db.yeastgenome.org/cgi-bin/locus.pl?locus=APC11)*,* [*DOA1*](http://db.yeastgenome.org/cgi-bin/locus.pl?locus=DOA1)*,* [*DSK2*](http://db.yeastgenome.org/cgi-bin/locus.pl?locus=DSK2)*,* [*HRD3*](http://db.yeastgenome.org/cgi-bin/locus.pl?locus=HRD3)*,* [*MET30*](http://db.yeastgenome.org/cgi-bin/locus.pl?locus=MET30)*,* [*NAS2*](http://db.yeastgenome.org/cgi-bin/locus.pl?locus=NAS2)*,* [*PNG1*](http://db.yeastgenome.org/cgi-bin/locus.pl?locus=PNG1)*,* [*PRE4*](http://db.yeastgenome.org/cgi-bin/locus.pl?locus=PRE4)*,* [*PRE5*](http://db.yeastgenome.org/cgi-bin/locus.pl?locus=PRE5)*,* [*PRE7*](http://db.yeastgenome.org/cgi-bin/locus.pl?locus=PRE7)*,* [*PUP1*](http://db.yeastgenome.org/cgi-bin/locus.pl?locus=PUP1)*,* [*PUP2*](http://db.yeastgenome.org/cgi-bin/locus.pl?locus=PUP2)*,* [*PUP3*](http://db.yeastgenome.org/cgi-bin/locus.pl?locus=PUP3)*,* [*QRI8*](http://db.yeastgenome.org/cgi-bin/locus.pl?locus=QRI8)*,* [*RPN1*](http://db.yeastgenome.org/cgi-bin/locus.pl?locus=RPN1)*,* [*RPN10*](http://db.yeastgenome.org/cgi-bin/locus.pl?locus=RPN10)*,* [*RPN14*](http://db.yeastgenome.org/cgi-bin/locus.pl?locus=RPN14)*,* [*RPT1*](http://db.yeastgenome.org/cgi-bin/locus.pl?locus=RPT1)*,* [*RTT101*](http://db.yeastgenome.org/cgi-bin/locus.pl?locus=RTT101)*,* [*SCL1*](http://db.yeastgenome.org/cgi-bin/locus.pl?locus=SCL1)*,* [*SEM1*](http://db.yeastgenome.org/cgi-bin/locus.pl?locus=SEM1)*,* [*SHP1*](http://db.yeastgenome.org/cgi-bin/locus.pl?locus=SHP1)*,* [*SKP1*](http://db.yeastgenome.org/cgi-bin/locus.pl?locus=SKP1)*,* [*SNF7*](http://db.yeastgenome.org/cgi-bin/locus.pl?locus=SNF7)*,* [*STS1*](http://db.yeastgenome.org/cgi-bin/locus.pl?locus=STS1)*,* [*SWM1*](http://db.yeastgenome.org/cgi-bin/locus.pl?locus=SWM1)*,* [*TUL1*](http://db.yeastgenome.org/cgi-bin/locus.pl?locus=TUL1)*,* [*UFO1*](http://db.yeastgenome.org/cgi-bin/locus.pl?locus=UFO1)*,* [*VID28*](http://db.yeastgenome.org/cgi-bin/locus.pl?locus=VID28)*,* [*YDR306C*](http://db.yeastgenome.org/cgi-bin/locus.pl?locus=YDR306C) |
| Action filament-based process | 25 out of 498 genes, 5.0% | 7.26e-5 | [*ACF2*](http://db.yeastgenome.org/cgi-bin/locus.pl?locus=ACF2)*,* [*AKL1*](http://db.yeastgenome.org/cgi-bin/locus.pl?locus=AKL1)*,* [*APP1*](http://db.yeastgenome.org/cgi-bin/locus.pl?locus=APP1)*,* [*ARC15*](http://db.yeastgenome.org/cgi-bin/locus.pl?locus=ARC15)*,* [*ARC40*](http://db.yeastgenome.org/cgi-bin/locus.pl?locus=ARC40)*,* [*ARK1*](http://db.yeastgenome.org/cgi-bin/locus.pl?locus=ARK1)*,* [*AVO2*](http://db.yeastgenome.org/cgi-bin/locus.pl?locus=AVO2)*,* [*BBC1*](http://db.yeastgenome.org/cgi-bin/locus.pl?locus=BBC1)*,* [*ENT2*](http://db.yeastgenome.org/cgi-bin/locus.pl?locus=ENT2)*,* [*HUA1*](http://db.yeastgenome.org/cgi-bin/locus.pl?locus=HUA1)*,* [*HUA2*](http://db.yeastgenome.org/cgi-bin/locus.pl?locus=HUA2)*,* [*LAS17*](http://db.yeastgenome.org/cgi-bin/locus.pl?locus=LAS17)*,* [*LSB5*](http://db.yeastgenome.org/cgi-bin/locus.pl?locus=LSB5)*,* [*PBS2*](http://db.yeastgenome.org/cgi-bin/locus.pl?locus=PBS2)*,* [*RHO1*](http://db.yeastgenome.org/cgi-bin/locus.pl?locus=RHO1)*,* [*ROM2*](http://db.yeastgenome.org/cgi-bin/locus.pl?locus=ROM2)*,* [*SAC6*](http://db.yeastgenome.org/cgi-bin/locus.pl?locus=SAC6)*,* [*SAC7*](http://db.yeastgenome.org/cgi-bin/locus.pl?locus=SAC7)*,* [*SCD5*](http://db.yeastgenome.org/cgi-bin/locus.pl?locus=SCD5)*,* [*SIW14*](http://db.yeastgenome.org/cgi-bin/locus.pl?locus=SIW14)*,* [*SLA2*](http://db.yeastgenome.org/cgi-bin/locus.pl?locus=SLA2)*,* [*SLG1*](http://db.yeastgenome.org/cgi-bin/locus.pl?locus=SLG1)*,* [*SLM1*](http://db.yeastgenome.org/cgi-bin/locus.pl?locus=SLM1)*,* [*VRP1*](http://db.yeastgenome.org/cgi-bin/locus.pl?locus=VRP1)*,* [*WHI2*](http://db.yeastgenome.org/cgi-bin/locus.pl?locus=WHI2) |
| External encapsulating structure organization and biosynthesis | 35 out of 498 genes, 7.0% | 0.00010 | [*AVO2*](http://db.yeastgenome.org/cgi-bin/locus.pl?locus=AVO2)*,* [*BCH1*](http://db.yeastgenome.org/cgi-bin/locus.pl?locus=BCH1)*,* [*BUD7*](http://db.yeastgenome.org/cgi-bin/locus.pl?locus=BUD7)*,* [*CHS5*](http://db.yeastgenome.org/cgi-bin/locus.pl?locus=CHS5)*,* [*CWH41*](http://db.yeastgenome.org/cgi-bin/locus.pl?locus=CWH41)*,* [*DFG5*](http://db.yeastgenome.org/cgi-bin/locus.pl?locus=DFG5)*,* [*ECM19*](http://db.yeastgenome.org/cgi-bin/locus.pl?locus=ECM19)*,* [*ECM21*](http://db.yeastgenome.org/cgi-bin/locus.pl?locus=ECM21)*,* [*FLC1*](http://db.yeastgenome.org/cgi-bin/locus.pl?locus=FLC1)*,* [*FLC2*](http://db.yeastgenome.org/cgi-bin/locus.pl?locus=FLC2)*,* [*HSP150*](http://db.yeastgenome.org/cgi-bin/locus.pl?locus=HSP150)*,* [*KIC1*](http://db.yeastgenome.org/cgi-bin/locus.pl?locus=KIC1)*,* [*KNH1*](http://db.yeastgenome.org/cgi-bin/locus.pl?locus=KNH1)*,* [*KRE1*](http://db.yeastgenome.org/cgi-bin/locus.pl?locus=KRE1)*,* [*KRE5*](http://db.yeastgenome.org/cgi-bin/locus.pl?locus=KRE5)*,* [*KRE6*](http://db.yeastgenome.org/cgi-bin/locus.pl?locus=KRE6)*,* [*MHP1*](http://db.yeastgenome.org/cgi-bin/locus.pl?locus=MHP1)*,* [*OSW2*](http://db.yeastgenome.org/cgi-bin/locus.pl?locus=OSW2)*,* [*PKH1*](http://db.yeastgenome.org/cgi-bin/locus.pl?locus=PKH1)*,* [*RCR1*](http://db.yeastgenome.org/cgi-bin/locus.pl?locus=RCR1)*,* [*RHO1*](http://db.yeastgenome.org/cgi-bin/locus.pl?locus=RHO1)*,* [*RIM101*](http://db.yeastgenome.org/cgi-bin/locus.pl?locus=RIM101)*,* [*RIM21*](http://db.yeastgenome.org/cgi-bin/locus.pl?locus=RIM21)*,* [*RLM1*](http://db.yeastgenome.org/cgi-bin/locus.pl?locus=RLM1)*,* [*ROM2*](http://db.yeastgenome.org/cgi-bin/locus.pl?locus=ROM2)*,* [*ROT1*](http://db.yeastgenome.org/cgi-bin/locus.pl?locus=ROT1)*,* [*SLA2*](http://db.yeastgenome.org/cgi-bin/locus.pl?locus=SLA2)*,* [*SLG1*](http://db.yeastgenome.org/cgi-bin/locus.pl?locus=SLG1)*,* [*SMK1*](http://db.yeastgenome.org/cgi-bin/locus.pl?locus=SMK1)*,* [*SPO75*](http://db.yeastgenome.org/cgi-bin/locus.pl?locus=SPO75)*,* [*SSD1*](http://db.yeastgenome.org/cgi-bin/locus.pl?locus=SSD1)*,* [*SWM1*](http://db.yeastgenome.org/cgi-bin/locus.pl?locus=SWM1)*,* [*TAX4*](http://db.yeastgenome.org/cgi-bin/locus.pl?locus=TAX4)*,* [*YLR194C*](http://db.yeastgenome.org/cgi-bin/locus.pl?locus=YLR194C)*,* [*YPS3*](http://db.yeastgenome.org/cgi-bin/locus.pl?locus=YPS3) |
| Cell wall organization and biogenesis | 35 out of 498 genes, 7.0% | 0.00010 | [*AVO2*](http://db.yeastgenome.org/cgi-bin/locus.pl?locus=AVO2)*,* [*BCH1*](http://db.yeastgenome.org/cgi-bin/locus.pl?locus=BCH1)*,* [*BUD7*](http://db.yeastgenome.org/cgi-bin/locus.pl?locus=BUD7)*,* [*CHS5*](http://db.yeastgenome.org/cgi-bin/locus.pl?locus=CHS5)*,* [*CWH41*](http://db.yeastgenome.org/cgi-bin/locus.pl?locus=CWH41)*,* [*DFG5*](http://db.yeastgenome.org/cgi-bin/locus.pl?locus=DFG5)*,* [*ECM19*](http://db.yeastgenome.org/cgi-bin/locus.pl?locus=ECM19)*,* [*ECM21*](http://db.yeastgenome.org/cgi-bin/locus.pl?locus=ECM21)*,* [*FLC1*](http://db.yeastgenome.org/cgi-bin/locus.pl?locus=FLC1)*,* [*FLC2*](http://db.yeastgenome.org/cgi-bin/locus.pl?locus=FLC2)*,* [*HSP150*](http://db.yeastgenome.org/cgi-bin/locus.pl?locus=HSP150)*,* [*KIC1*](http://db.yeastgenome.org/cgi-bin/locus.pl?locus=KIC1)*,* [*KNH1*](http://db.yeastgenome.org/cgi-bin/locus.pl?locus=KNH1)*,* [*KRE1*](http://db.yeastgenome.org/cgi-bin/locus.pl?locus=KRE1)*,* [*KRE5*](http://db.yeastgenome.org/cgi-bin/locus.pl?locus=KRE5)*,* [*KRE6*](http://db.yeastgenome.org/cgi-bin/locus.pl?locus=KRE6)*,* [*MHP1*](http://db.yeastgenome.org/cgi-bin/locus.pl?locus=MHP1)*,* [*OSW2*](http://db.yeastgenome.org/cgi-bin/locus.pl?locus=OSW2)*,* [*PKH1*](http://db.yeastgenome.org/cgi-bin/locus.pl?locus=PKH1)*,* [*RCR1*](http://db.yeastgenome.org/cgi-bin/locus.pl?locus=RCR1)*,* [*RHO1*](http://db.yeastgenome.org/cgi-bin/locus.pl?locus=RHO1)*,* [*RIM101*](http://db.yeastgenome.org/cgi-bin/locus.pl?locus=RIM101)*,* [*RIM21*](http://db.yeastgenome.org/cgi-bin/locus.pl?locus=RIM21)*,* [*RLM1*](http://db.yeastgenome.org/cgi-bin/locus.pl?locus=RLM1)*,* [*ROM2*](http://db.yeastgenome.org/cgi-bin/locus.pl?locus=ROM2)*,* [*ROT1*](http://db.yeastgenome.org/cgi-bin/locus.pl?locus=ROT1)*,* [*SLA2*](http://db.yeastgenome.org/cgi-bin/locus.pl?locus=SLA2)*,* [*SLG1*](http://db.yeastgenome.org/cgi-bin/locus.pl?locus=SLG1)*,* [*SMK1*](http://db.yeastgenome.org/cgi-bin/locus.pl?locus=SMK1)*,* [*SPO75*](http://db.yeastgenome.org/cgi-bin/locus.pl?locus=SPO75)*,* [*SSD1*](http://db.yeastgenome.org/cgi-bin/locus.pl?locus=SSD1)*,* [*SWM1*](http://db.yeastgenome.org/cgi-bin/locus.pl?locus=SWM1)*,* [*TAX4*](http://db.yeastgenome.org/cgi-bin/locus.pl?locus=TAX4)*,* [*YLR194C*](http://db.yeastgenome.org/cgi-bin/locus.pl?locus=YLR194C)*,* [*YPS3*](http://db.yeastgenome.org/cgi-bin/locus.pl?locus=YPS3) |
| Modification-dependent macromolecule catabolic process | 30 out of 498 genes, 6.0% | 0.00011 | [*ADD66*](http://db.yeastgenome.org/cgi-bin/locus.pl?locus=ADD66)*,* [*APC11*](http://db.yeastgenome.org/cgi-bin/locus.pl?locus=APC11)*,* [*DOA1*](http://db.yeastgenome.org/cgi-bin/locus.pl?locus=DOA1)*,* [*DSK2*](http://db.yeastgenome.org/cgi-bin/locus.pl?locus=DSK2)*,* [*HRD3*](http://db.yeastgenome.org/cgi-bin/locus.pl?locus=HRD3)*,* [*MET30*](http://db.yeastgenome.org/cgi-bin/locus.pl?locus=MET30)*,* [*NAS2*](http://db.yeastgenome.org/cgi-bin/locus.pl?locus=NAS2)*,* [*PRE4*](http://db.yeastgenome.org/cgi-bin/locus.pl?locus=PRE4)*,* [*PRE5*](http://db.yeastgenome.org/cgi-bin/locus.pl?locus=PRE5)*,* [*PRE7*](http://db.yeastgenome.org/cgi-bin/locus.pl?locus=PRE7)*,* [*PUP1*](http://db.yeastgenome.org/cgi-bin/locus.pl?locus=PUP1)*,* [*PUP2*](http://db.yeastgenome.org/cgi-bin/locus.pl?locus=PUP2)*,* [*PUP3*](http://db.yeastgenome.org/cgi-bin/locus.pl?locus=PUP3)*,* [*QRI8*](http://db.yeastgenome.org/cgi-bin/locus.pl?locus=QRI8)*,* [*RPN1*](http://db.yeastgenome.org/cgi-bin/locus.pl?locus=RPN1)*,* [*RPN10*](http://db.yeastgenome.org/cgi-bin/locus.pl?locus=RPN10)*,* [*RPN14*](http://db.yeastgenome.org/cgi-bin/locus.pl?locus=RPN14)*,* [*RPT1*](http://db.yeastgenome.org/cgi-bin/locus.pl?locus=RPT1)*,* [*RTT101*](http://db.yeastgenome.org/cgi-bin/locus.pl?locus=RTT101)*,* [*SCL1*](http://db.yeastgenome.org/cgi-bin/locus.pl?locus=SCL1)*,* [*SEM1*](http://db.yeastgenome.org/cgi-bin/locus.pl?locus=SEM1)*,* [*SHP1*](http://db.yeastgenome.org/cgi-bin/locus.pl?locus=SHP1)*,* [*SKP1*](http://db.yeastgenome.org/cgi-bin/locus.pl?locus=SKP1)*,* [*SNF7*](http://db.yeastgenome.org/cgi-bin/locus.pl?locus=SNF7)*,* [*STS1*](http://db.yeastgenome.org/cgi-bin/locus.pl?locus=STS1)*,* [*SWM1*](http://db.yeastgenome.org/cgi-bin/locus.pl?locus=SWM1)*,* [*TUL1*](http://db.yeastgenome.org/cgi-bin/locus.pl?locus=TUL1)*,* [*UFO1*](http://db.yeastgenome.org/cgi-bin/locus.pl?locus=UFO1)*,* [*VID28*](http://db.yeastgenome.org/cgi-bin/locus.pl?locus=VID28)*,* [*YDR306C*](http://db.yeastgenome.org/cgi-bin/locus.pl?locus=YDR306C) |
| Chitin- and beta-glucan-containing cell wall organization and biogenesis | 13 out of 498 genes, 2.6% | 0.00065 | [*AVO2*](http://db.yeastgenome.org/cgi-bin/locus.pl?locus=AVO2)*,* [*BCH1*](http://db.yeastgenome.org/cgi-bin/locus.pl?locus=BCH1)*,* [*BUD7*](http://db.yeastgenome.org/cgi-bin/locus.pl?locus=BUD7)*,* [*DFG5*](http://db.yeastgenome.org/cgi-bin/locus.pl?locus=DFG5)*,* [*FLC1*](http://db.yeastgenome.org/cgi-bin/locus.pl?locus=FLC1)*,* [*FLC2*](http://db.yeastgenome.org/cgi-bin/locus.pl?locus=FLC2)*,* [*KNH1*](http://db.yeastgenome.org/cgi-bin/locus.pl?locus=KNH1)*,* [*KRE5*](http://db.yeastgenome.org/cgi-bin/locus.pl?locus=KRE5)*,* [*KRE6*](http://db.yeastgenome.org/cgi-bin/locus.pl?locus=KRE6)*,* [*RIM101*](http://db.yeastgenome.org/cgi-bin/locus.pl?locus=RIM101)*,* [*RIM21*](http://db.yeastgenome.org/cgi-bin/locus.pl?locus=RIM21)*,* [*YLR194C*](http://db.yeastgenome.org/cgi-bin/locus.pl?locus=YLR194C)*,* [*YPS3*](http://db.yeastgenome.org/cgi-bin/locus.pl?locus=YPS3) |
| Cellular component organization and biogenesis | 197 out of 498 genes, 39.6% | 0.00162 | [*ABF2*](http://db.yeastgenome.org/cgi-bin/locus.pl?locus=ABF2)*,* [*ACF2*](http://db.yeastgenome.org/cgi-bin/locus.pl?locus=ACF2)*,* [*ACS1*](http://db.yeastgenome.org/cgi-bin/locus.pl?locus=ACS1)*,* [*ADD66*](http://db.yeastgenome.org/cgi-bin/locus.pl?locus=ADD66)*,* [*ADR1*](http://db.yeastgenome.org/cgi-bin/locus.pl?locus=ADR1)*,* [*AFG3*](http://db.yeastgenome.org/cgi-bin/locus.pl?locus=AFG3)*,* [*AKL1*](http://db.yeastgenome.org/cgi-bin/locus.pl?locus=AKL1)*,* [*APC11*](http://db.yeastgenome.org/cgi-bin/locus.pl?locus=APC11)*,* [*APL2*](http://db.yeastgenome.org/cgi-bin/locus.pl?locus=APL2)*,* [*APL4*](http://db.yeastgenome.org/cgi-bin/locus.pl?locus=APL4)*,* [*APM2*](http://db.yeastgenome.org/cgi-bin/locus.pl?locus=APM2)*,* [*APP1*](http://db.yeastgenome.org/cgi-bin/locus.pl?locus=APP1)*,* [*ARC15*](http://db.yeastgenome.org/cgi-bin/locus.pl?locus=ARC15)*,* [*ARC40*](http://db.yeastgenome.org/cgi-bin/locus.pl?locus=ARC40)*,* [*ARK1*](http://db.yeastgenome.org/cgi-bin/locus.pl?locus=ARK1)*,* [*ATG18*](http://db.yeastgenome.org/cgi-bin/locus.pl?locus=ATG18)*,* [*ATG20*](http://db.yeastgenome.org/cgi-bin/locus.pl?locus=ATG20)*,* [*ATG4*](http://db.yeastgenome.org/cgi-bin/locus.pl?locus=ATG4)*,* [*ATG5*](http://db.yeastgenome.org/cgi-bin/locus.pl?locus=ATG5)*,* [*AVO2*](http://db.yeastgenome.org/cgi-bin/locus.pl?locus=AVO2)*,* [*AVT3*](http://db.yeastgenome.org/cgi-bin/locus.pl?locus=AVT3)*,* [*BBC1*](http://db.yeastgenome.org/cgi-bin/locus.pl?locus=BBC1)*,* [*BCH1*](http://db.yeastgenome.org/cgi-bin/locus.pl?locus=BCH1)*,* [*BIK1*](http://db.yeastgenome.org/cgi-bin/locus.pl?locus=BIK1)*,* [*BOI1*](http://db.yeastgenome.org/cgi-bin/locus.pl?locus=BOI1)*,* [*BUD7*](http://db.yeastgenome.org/cgi-bin/locus.pl?locus=BUD7)*,* [*CBP3*](http://db.yeastgenome.org/cgi-bin/locus.pl?locus=CBP3)*,* [*CCT2*](http://db.yeastgenome.org/cgi-bin/locus.pl?locus=CCT2)*,* [*CHS5*](http://db.yeastgenome.org/cgi-bin/locus.pl?locus=CHS5)*,* [*COX19*](http://db.yeastgenome.org/cgi-bin/locus.pl?locus=COX19)*,* [*COY1*](http://db.yeastgenome.org/cgi-bin/locus.pl?locus=COY1)*,* [*CST6*](http://db.yeastgenome.org/cgi-bin/locus.pl?locus=CST6)*,* [*CTI6*](http://db.yeastgenome.org/cgi-bin/locus.pl?locus=CTI6)*,* [*CWH41*](http://db.yeastgenome.org/cgi-bin/locus.pl?locus=CWH41)*,* [*CYC3*](http://db.yeastgenome.org/cgi-bin/locus.pl?locus=CYC3)*,* [*DBR1*](http://db.yeastgenome.org/cgi-bin/locus.pl?locus=DBR1)*,* [*DFG5*](http://db.yeastgenome.org/cgi-bin/locus.pl?locus=DFG5)*,* [*DID2*](http://db.yeastgenome.org/cgi-bin/locus.pl?locus=DID2)*,* [*DIG2*](http://db.yeastgenome.org/cgi-bin/locus.pl?locus=DIG2)*,* [*DSK2*](http://db.yeastgenome.org/cgi-bin/locus.pl?locus=DSK2)*,* [*EAF3*](http://db.yeastgenome.org/cgi-bin/locus.pl?locus=EAF3)*,* [*EAP1*](http://db.yeastgenome.org/cgi-bin/locus.pl?locus=EAP1)*,* [*ECM19*](http://db.yeastgenome.org/cgi-bin/locus.pl?locus=ECM19)*,* [*ECM21*](http://db.yeastgenome.org/cgi-bin/locus.pl?locus=ECM21)*,* [*EHD3*](http://db.yeastgenome.org/cgi-bin/locus.pl?locus=EHD3)*,* [*ENT2*](http://db.yeastgenome.org/cgi-bin/locus.pl?locus=ENT2)*,* [*ENT5*](http://db.yeastgenome.org/cgi-bin/locus.pl?locus=ENT5)*,* [*ERP6*](http://db.yeastgenome.org/cgi-bin/locus.pl?locus=ERP6)*,* [*ERV1*](http://db.yeastgenome.org/cgi-bin/locus.pl?locus=ERV1)*,* [*ERV41*](http://db.yeastgenome.org/cgi-bin/locus.pl?locus=ERV41)*,* [*FLC1*](http://db.yeastgenome.org/cgi-bin/locus.pl?locus=FLC1)*,* [*FLC2*](http://db.yeastgenome.org/cgi-bin/locus.pl?locus=FLC2)*,* [*FMC1*](http://db.yeastgenome.org/cgi-bin/locus.pl?locus=FMC1)*,* [*FYV6*](http://db.yeastgenome.org/cgi-bin/locus.pl?locus=FYV6)*,* [*GRH1*](http://db.yeastgenome.org/cgi-bin/locus.pl?locus=GRH1)*,* [*HEX3*](http://db.yeastgenome.org/cgi-bin/locus.pl?locus=HEX3)*,* [*HMS2*](http://db.yeastgenome.org/cgi-bin/locus.pl?locus=HMS2)*,* [*HOP2*](http://db.yeastgenome.org/cgi-bin/locus.pl?locus=HOP2)*,* [*HOS4*](http://db.yeastgenome.org/cgi-bin/locus.pl?locus=HOS4)*,* [*HRR25*](http://db.yeastgenome.org/cgi-bin/locus.pl?locus=HRR25)*,* [*HSF1*](http://db.yeastgenome.org/cgi-bin/locus.pl?locus=HSF1)*,* [*HSP150*](http://db.yeastgenome.org/cgi-bin/locus.pl?locus=HSP150)*,* [*HUA1*](http://db.yeastgenome.org/cgi-bin/locus.pl?locus=HUA1)*,* [*HUA2*](http://db.yeastgenome.org/cgi-bin/locus.pl?locus=HUA2)*,* [*IML3*](http://db.yeastgenome.org/cgi-bin/locus.pl?locus=IML3)*,* [*IMP2*](http://db.yeastgenome.org/cgi-bin/locus.pl?locus=IMP2)*,* [*INO80*](http://db.yeastgenome.org/cgi-bin/locus.pl?locus=INO80)*,* [*IOC3*](http://db.yeastgenome.org/cgi-bin/locus.pl?locus=IOC3)*,* [*ITT1*](http://db.yeastgenome.org/cgi-bin/locus.pl?locus=ITT1)*,* [*IVY1*](http://db.yeastgenome.org/cgi-bin/locus.pl?locus=IVY1)*,* [*KIC1*](http://db.yeastgenome.org/cgi-bin/locus.pl?locus=KIC1)*,* [*KNH1*](http://db.yeastgenome.org/cgi-bin/locus.pl?locus=KNH1)*,* [*KRE1*](http://db.yeastgenome.org/cgi-bin/locus.pl?locus=KRE1)*,* [*KRE5*](http://db.yeastgenome.org/cgi-bin/locus.pl?locus=KRE5)*,* [*KRE6*](http://db.yeastgenome.org/cgi-bin/locus.pl?locus=KRE6)*,* [*LAS17*](http://db.yeastgenome.org/cgi-bin/locus.pl?locus=LAS17)*,* [*LSB5*](http://db.yeastgenome.org/cgi-bin/locus.pl?locus=LSB5)*,* [*MAD2*](http://db.yeastgenome.org/cgi-bin/locus.pl?locus=MAD2)*,* [*MAK10*](http://db.yeastgenome.org/cgi-bin/locus.pl?locus=MAK10)*,* [*MAS2*](http://db.yeastgenome.org/cgi-bin/locus.pl?locus=MAS2)*,* [*MDM12*](http://db.yeastgenome.org/cgi-bin/locus.pl?locus=MDM12)*,* [*MDM34*](http://db.yeastgenome.org/cgi-bin/locus.pl?locus=MDM34)*,* [*MDM35*](http://db.yeastgenome.org/cgi-bin/locus.pl?locus=MDM35)*,* [*MDR1*](http://db.yeastgenome.org/cgi-bin/locus.pl?locus=MDR1)*,* [*MDY2*](http://db.yeastgenome.org/cgi-bin/locus.pl?locus=MDY2)*,* [*MFT1*](http://db.yeastgenome.org/cgi-bin/locus.pl?locus=MFT1)*,* [*MHP1*](http://db.yeastgenome.org/cgi-bin/locus.pl?locus=MHP1)*,* [*MHR1*](http://db.yeastgenome.org/cgi-bin/locus.pl?locus=MHR1)*,* [*MON2*](http://db.yeastgenome.org/cgi-bin/locus.pl?locus=MON2)*,* [*MRPL9*](http://db.yeastgenome.org/cgi-bin/locus.pl?locus=MRPL9)*,* [*MSN5*](http://db.yeastgenome.org/cgi-bin/locus.pl?locus=MSN5)*,* [*MSS11*](http://db.yeastgenome.org/cgi-bin/locus.pl?locus=MSS11)*,* [*MST27*](http://db.yeastgenome.org/cgi-bin/locus.pl?locus=MST27)*,* [*NAB2*](http://db.yeastgenome.org/cgi-bin/locus.pl?locus=NAB2)*,* [*NAB3*](http://db.yeastgenome.org/cgi-bin/locus.pl?locus=NAB3)*,* [*NPT1*](http://db.yeastgenome.org/cgi-bin/locus.pl?locus=NPT1)*,* [*NYV1*](http://db.yeastgenome.org/cgi-bin/locus.pl?locus=NYV1)*,* [*OAF1*](http://db.yeastgenome.org/cgi-bin/locus.pl?locus=OAF1)*,* [*ORT1*](http://db.yeastgenome.org/cgi-bin/locus.pl?locus=ORT1)*,* [*OSH2*](http://db.yeastgenome.org/cgi-bin/locus.pl?locus=OSH2)*,* [*OSW2*](http://db.yeastgenome.org/cgi-bin/locus.pl?locus=OSW2)*,* [*PAM1*](http://db.yeastgenome.org/cgi-bin/locus.pl?locus=PAM1)*,* [*PAP1*](http://db.yeastgenome.org/cgi-bin/locus.pl?locus=PAP1)*,* [*PBS2*](http://db.yeastgenome.org/cgi-bin/locus.pl?locus=PBS2)*,* [*PCP1*](http://db.yeastgenome.org/cgi-bin/locus.pl?locus=PCP1)*,* [*PEP8*](http://db.yeastgenome.org/cgi-bin/locus.pl?locus=PEP8)*,* [*PEX10*](http://db.yeastgenome.org/cgi-bin/locus.pl?locus=PEX10)*,* [*PEX14*](http://db.yeastgenome.org/cgi-bin/locus.pl?locus=PEX14)*,* [*PEX2*](http://db.yeastgenome.org/cgi-bin/locus.pl?locus=PEX2)*,* [*PIL1*](http://db.yeastgenome.org/cgi-bin/locus.pl?locus=PIL1)*,* [*PKH1*](http://db.yeastgenome.org/cgi-bin/locus.pl?locus=PKH1)*,* [*PKP1*](http://db.yeastgenome.org/cgi-bin/locus.pl?locus=PKP1)*,* [*PMR1*](http://db.yeastgenome.org/cgi-bin/locus.pl?locus=PMR1)*,* [*PRM9*](http://db.yeastgenome.org/cgi-bin/locus.pl?locus=PRM9)*,* [*QRI8*](http://db.yeastgenome.org/cgi-bin/locus.pl?locus=QRI8)*,* [*RCL1*](http://db.yeastgenome.org/cgi-bin/locus.pl?locus=RCL1)*,* [*RCR1*](http://db.yeastgenome.org/cgi-bin/locus.pl?locus=RCR1)*,* [*RHO1*](http://db.yeastgenome.org/cgi-bin/locus.pl?locus=RHO1)*,* [*RIM101*](http://db.yeastgenome.org/cgi-bin/locus.pl?locus=RIM101)*,* [*RIM20*](http://db.yeastgenome.org/cgi-bin/locus.pl?locus=RIM20)*,* [*RIM21*](http://db.yeastgenome.org/cgi-bin/locus.pl?locus=RIM21)*,* [*RLM1*](http://db.yeastgenome.org/cgi-bin/locus.pl?locus=RLM1)*,* [*ROM2*](http://db.yeastgenome.org/cgi-bin/locus.pl?locus=ROM2)*,* [*ROT1*](http://db.yeastgenome.org/cgi-bin/locus.pl?locus=ROT1)*,* [*RPH1*](http://db.yeastgenome.org/cgi-bin/locus.pl?locus=RPH1)*,* [*RRG9*](http://db.yeastgenome.org/cgi-bin/locus.pl?locus=YNL213C)*,* [*RSN1*](http://db.yeastgenome.org/cgi-bin/locus.pl?locus=RSN1)*,* [*RTG1*](http://db.yeastgenome.org/cgi-bin/locus.pl?locus=RTG1)*,* [*RTG2*](http://db.yeastgenome.org/cgi-bin/locus.pl?locus=RTG2)*,* [*RTS1*](http://db.yeastgenome.org/cgi-bin/locus.pl?locus=RTS1)*,* [*RVS167*](http://db.yeastgenome.org/cgi-bin/locus.pl?locus=RVS167)*,* [*SAC6*](http://db.yeastgenome.org/cgi-bin/locus.pl?locus=SAC6)*,* [*SAC7*](http://db.yeastgenome.org/cgi-bin/locus.pl?locus=SAC7)*,* [*SCD5*](http://db.yeastgenome.org/cgi-bin/locus.pl?locus=SCD5)*,* [*SEC9*](http://db.yeastgenome.org/cgi-bin/locus.pl?locus=SEC9)*,* [*SED4*](http://db.yeastgenome.org/cgi-bin/locus.pl?locus=SED4)*,* [*SED5*](http://db.yeastgenome.org/cgi-bin/locus.pl?locus=SED5)*,* [*SEM1*](http://db.yeastgenome.org/cgi-bin/locus.pl?locus=SEM1)*,* [*SHP1*](http://db.yeastgenome.org/cgi-bin/locus.pl?locus=SHP1)*,* [*SIW14*](http://db.yeastgenome.org/cgi-bin/locus.pl?locus=SIW14)*,* [*SKP1*](http://db.yeastgenome.org/cgi-bin/locus.pl?locus=SKP1)*,* [*SLA2*](http://db.yeastgenome.org/cgi-bin/locus.pl?locus=SLA2)*,* [*SLG1*](http://db.yeastgenome.org/cgi-bin/locus.pl?locus=SLG1)*,* [*SLM1*](http://db.yeastgenome.org/cgi-bin/locus.pl?locus=SLM1)*,* [*SMK1*](http://db.yeastgenome.org/cgi-bin/locus.pl?locus=SMK1)*,* [*SNC1*](http://db.yeastgenome.org/cgi-bin/locus.pl?locus=SNC1)*,* [*SNF11*](http://db.yeastgenome.org/cgi-bin/locus.pl?locus=SNF11)*,* [*SNF7*](http://db.yeastgenome.org/cgi-bin/locus.pl?locus=SNF7)*,* [*SNU114*](http://db.yeastgenome.org/cgi-bin/locus.pl?locus=SNU114)*,* [*SOH1*](http://db.yeastgenome.org/cgi-bin/locus.pl?locus=SOH1)*,* [*SOK2*](http://db.yeastgenome.org/cgi-bin/locus.pl?locus=SOK2)*,* [*SPO14*](http://db.yeastgenome.org/cgi-bin/locus.pl?locus=SPO14)*,* [*SPO75*](http://db.yeastgenome.org/cgi-bin/locus.pl?locus=SPO75)*,* [*SPT20*](http://db.yeastgenome.org/cgi-bin/locus.pl?locus=SPT20)*,* [*SSD1*](http://db.yeastgenome.org/cgi-bin/locus.pl?locus=SSD1)*,* [*SSL2*](http://db.yeastgenome.org/cgi-bin/locus.pl?locus=SSL2)*,* [*SSO1*](http://db.yeastgenome.org/cgi-bin/locus.pl?locus=SSO1)*,* [*SSP120*](http://db.yeastgenome.org/cgi-bin/locus.pl?locus=SSP120)*,* [*STB2*](http://db.yeastgenome.org/cgi-bin/locus.pl?locus=STB2)*,* [*STE11*](http://db.yeastgenome.org/cgi-bin/locus.pl?locus=STE11)*,* [*STE4*](http://db.yeastgenome.org/cgi-bin/locus.pl?locus=STE4)*,* [*STV1*](http://db.yeastgenome.org/cgi-bin/locus.pl?locus=STV1)*,* [*SUB1*](http://db.yeastgenome.org/cgi-bin/locus.pl?locus=SUB1)*,* [*SWC5*](http://db.yeastgenome.org/cgi-bin/locus.pl?locus=SWC5)*,* [*SWM1*](http://db.yeastgenome.org/cgi-bin/locus.pl?locus=SWM1)*,* [*SWR1*](http://db.yeastgenome.org/cgi-bin/locus.pl?locus=SWR1)*,* [*TAX4*](http://db.yeastgenome.org/cgi-bin/locus.pl?locus=TAX4)*,* [*TFA2*](http://db.yeastgenome.org/cgi-bin/locus.pl?locus=TFA2)*,* [*TFG1*](http://db.yeastgenome.org/cgi-bin/locus.pl?locus=TFG1)*,* [*THI4*](http://db.yeastgenome.org/cgi-bin/locus.pl?locus=THI4)*,* [*TIF4632*](http://db.yeastgenome.org/cgi-bin/locus.pl?locus=TIF4632)*,* [*TIM9*](http://db.yeastgenome.org/cgi-bin/locus.pl?locus=TIM9)*,* [*TRS31*](http://db.yeastgenome.org/cgi-bin/locus.pl?locus=TRS31)*,* [*UME6*](http://db.yeastgenome.org/cgi-bin/locus.pl?locus=UME6)*,* [*URE2*](http://db.yeastgenome.org/cgi-bin/locus.pl?locus=URE2)*,* [*UTH1*](http://db.yeastgenome.org/cgi-bin/locus.pl?locus=UTH1)*,* [*VAM6*](http://db.yeastgenome.org/cgi-bin/locus.pl?locus=VAM6)*,* [*VID21*](http://db.yeastgenome.org/cgi-bin/locus.pl?locus=VID21)*,* [*VPS21*](http://db.yeastgenome.org/cgi-bin/locus.pl?locus=VPS21)*,* [*VPS29*](http://db.yeastgenome.org/cgi-bin/locus.pl?locus=VPS29)*,* [*VPS30*](http://db.yeastgenome.org/cgi-bin/locus.pl?locus=VPS30)*,* [*VPS62*](http://db.yeastgenome.org/cgi-bin/locus.pl?locus=VPS62)*,* [*VPS70*](http://db.yeastgenome.org/cgi-bin/locus.pl?locus=VPS70)*,* [*VRP1*](http://db.yeastgenome.org/cgi-bin/locus.pl?locus=VRP1)*,* [*WHI2*](http://db.yeastgenome.org/cgi-bin/locus.pl?locus=WHI2)*,* [*XDJ1*](http://db.yeastgenome.org/cgi-bin/locus.pl?locus=XDJ1)*,* [*YBL055C*](http://db.yeastgenome.org/cgi-bin/locus.pl?locus=YBL055C)*,* [*YCR061W*](http://db.yeastgenome.org/cgi-bin/locus.pl?locus=YCR061W)*,* [*YIA6*](http://db.yeastgenome.org/cgi-bin/locus.pl?locus=YIA6)*,* [*YIP3*](http://db.yeastgenome.org/cgi-bin/locus.pl?locus=YIP3)*,* [*YKT6*](http://db.yeastgenome.org/cgi-bin/locus.pl?locus=YKT6)*,* [*YLR194C*](http://db.yeastgenome.org/cgi-bin/locus.pl?locus=YLR194C)*,* [*YOR1*](http://db.yeastgenome.org/cgi-bin/locus.pl?locus=YOR1)*,* [*YPK1*](http://db.yeastgenome.org/cgi-bin/locus.pl?locus=YPK1)*,* [*YPS3*](http://db.yeastgenome.org/cgi-bin/locus.pl?locus=YPS3)*,* [*YTA7*](http://db.yeastgenome.org/cgi-bin/locus.pl?locus=YTA7)*,* [*ZDS1*](http://db.yeastgenome.org/cgi-bin/locus.pl?locus=ZDS1) |
| Actin filament organization | 16 out of 498 genes, 3.2% | 0.00198 | [*ARC40*](http://db.yeastgenome.org/cgi-bin/locus.pl?locus=ARC40)*,* [*ARK1*](http://db.yeastgenome.org/cgi-bin/locus.pl?locus=ARK1)*,* [*ENT2*](http://db.yeastgenome.org/cgi-bin/locus.pl?locus=ENT2)*,* [*LAS17*](http://db.yeastgenome.org/cgi-bin/locus.pl?locus=LAS17)*,* [*LSB5*](http://db.yeastgenome.org/cgi-bin/locus.pl?locus=LSB5)*,* [*PBS2*](http://db.yeastgenome.org/cgi-bin/locus.pl?locus=PBS2)*,* [*RHO1*](http://db.yeastgenome.org/cgi-bin/locus.pl?locus=RHO1)*,* [*ROM2*](http://db.yeastgenome.org/cgi-bin/locus.pl?locus=ROM2)*,* [*SAC6*](http://db.yeastgenome.org/cgi-bin/locus.pl?locus=SAC6)*,* [*SAC7*](http://db.yeastgenome.org/cgi-bin/locus.pl?locus=SAC7)*,* [*SCD5*](http://db.yeastgenome.org/cgi-bin/locus.pl?locus=SCD5)*,* [*SIW14*](http://db.yeastgenome.org/cgi-bin/locus.pl?locus=SIW14)*,* [*SLA2*](http://db.yeastgenome.org/cgi-bin/locus.pl?locus=SLA2)*,* [*SLM1*](http://db.yeastgenome.org/cgi-bin/locus.pl?locus=SLM1)*,* [*VRP1*](http://db.yeastgenome.org/cgi-bin/locus.pl?locus=VRP1)*,* [*WHI2*](http://db.yeastgenome.org/cgi-bin/locus.pl?locus=WHI2) |
| Membrane organization and biogenesis | 30 out of 498 genes, 6.0% | 0.00264 | [*AKL1*](http://db.yeastgenome.org/cgi-bin/locus.pl?locus=AKL1)*,* [*ARK1*](http://db.yeastgenome.org/cgi-bin/locus.pl?locus=ARK1)*,* [*EHD3*](http://db.yeastgenome.org/cgi-bin/locus.pl?locus=EHD3)*,* [*ENT2*](http://db.yeastgenome.org/cgi-bin/locus.pl?locus=ENT2)*,* [*LAS17*](http://db.yeastgenome.org/cgi-bin/locus.pl?locus=LAS17)*,* [*LSB5*](http://db.yeastgenome.org/cgi-bin/locus.pl?locus=LSB5)*,* [*MON2*](http://db.yeastgenome.org/cgi-bin/locus.pl?locus=MON2)*,* [*NYV1*](http://db.yeastgenome.org/cgi-bin/locus.pl?locus=NYV1)*,* [*OSH2*](http://db.yeastgenome.org/cgi-bin/locus.pl?locus=OSH2)*,* [*PIL1*](http://db.yeastgenome.org/cgi-bin/locus.pl?locus=PIL1)*,* [*PKH1*](http://db.yeastgenome.org/cgi-bin/locus.pl?locus=PKH1)*,* [*RHO1*](http://db.yeastgenome.org/cgi-bin/locus.pl?locus=RHO1)*,* [*ROM2*](http://db.yeastgenome.org/cgi-bin/locus.pl?locus=ROM2)*,* [*RVS167*](http://db.yeastgenome.org/cgi-bin/locus.pl?locus=RVS167)*,* [*SAC6*](http://db.yeastgenome.org/cgi-bin/locus.pl?locus=SAC6)*,* [*SCD5*](http://db.yeastgenome.org/cgi-bin/locus.pl?locus=SCD5)*,* [*SEC9*](http://db.yeastgenome.org/cgi-bin/locus.pl?locus=SEC9)*,* [*SED5*](http://db.yeastgenome.org/cgi-bin/locus.pl?locus=SED5)*,* [*SIW14*](http://db.yeastgenome.org/cgi-bin/locus.pl?locus=SIW14)*,* [*SLA2*](http://db.yeastgenome.org/cgi-bin/locus.pl?locus=SLA2)*,* [*SLG1*](http://db.yeastgenome.org/cgi-bin/locus.pl?locus=SLG1)*,* [*SNC1*](http://db.yeastgenome.org/cgi-bin/locus.pl?locus=SNC1)*,* [*SSO1*](http://db.yeastgenome.org/cgi-bin/locus.pl?locus=SSO1)*,* [*TIM9*](http://db.yeastgenome.org/cgi-bin/locus.pl?locus=TIM9)*,* [*VAM6*](http://db.yeastgenome.org/cgi-bin/locus.pl?locus=VAM6)*,* [*VPS21*](http://db.yeastgenome.org/cgi-bin/locus.pl?locus=VPS21)*,* [*VRP1*](http://db.yeastgenome.org/cgi-bin/locus.pl?locus=VRP1)*,* [*WHI2*](http://db.yeastgenome.org/cgi-bin/locus.pl?locus=WHI2)*,* [*YKT6*](http://db.yeastgenome.org/cgi-bin/locus.pl?locus=YKT6)*,* [*YPK1*](http://db.yeastgenome.org/cgi-bin/locus.pl?locus=YPK1) |
| Cell wall biogenesis | 10 out of 498 genes, 2.0% | 0.00579 | [*BCH1*](http://db.yeastgenome.org/cgi-bin/locus.pl?locus=BCH1)*,* [*BUD7*](http://db.yeastgenome.org/cgi-bin/locus.pl?locus=BUD7)*,* [*DFG5*](http://db.yeastgenome.org/cgi-bin/locus.pl?locus=DFG5)*,* [*FLC1*](http://db.yeastgenome.org/cgi-bin/locus.pl?locus=FLC1)*,* [*FLC2*](http://db.yeastgenome.org/cgi-bin/locus.pl?locus=FLC2)*,* [*KNH1*](http://db.yeastgenome.org/cgi-bin/locus.pl?locus=KNH1)*,* [*KRE5*](http://db.yeastgenome.org/cgi-bin/locus.pl?locus=KRE5)*,* [*KRE6*](http://db.yeastgenome.org/cgi-bin/locus.pl?locus=KRE6)*,* [*RIM101*](http://db.yeastgenome.org/cgi-bin/locus.pl?locus=RIM101)*,* [*RIM21*](http://db.yeastgenome.org/cgi-bin/locus.pl?locus=RIM21) |
| Chitin and beta-glucan-containing cell wall biogenesis | 10 out of 498 genes, 2.0% | 0.00579 | [*BCH1*](http://db.yeastgenome.org/cgi-bin/locus.pl?locus=BCH1)*,* [*BUD7*](http://db.yeastgenome.org/cgi-bin/locus.pl?locus=BUD7)*,* [*DFG5*](http://db.yeastgenome.org/cgi-bin/locus.pl?locus=DFG5)*,* [*FLC1*](http://db.yeastgenome.org/cgi-bin/locus.pl?locus=FLC1)*,* [*FLC2*](http://db.yeastgenome.org/cgi-bin/locus.pl?locus=FLC2)*,* [*KNH1*](http://db.yeastgenome.org/cgi-bin/locus.pl?locus=KNH1)*,* [*KRE5*](http://db.yeastgenome.org/cgi-bin/locus.pl?locus=KRE5)*,* [*KRE6*](http://db.yeastgenome.org/cgi-bin/locus.pl?locus=KRE6)*,* [*RIM101*](http://db.yeastgenome.org/cgi-bin/locus.pl?locus=RIM101)*,* [*RIM21*](http://db.yeastgenome.org/cgi-bin/locus.pl?locus=RIM21) |
| Biopolymer catabolic process | 39 out of 498 genes, 7.8% | 0.00585 | [*ADD66*](http://db.yeastgenome.org/cgi-bin/locus.pl?locus=ADD66)*,* [*APC11*](http://db.yeastgenome.org/cgi-bin/locus.pl?locus=APC11)*,* [*CHS5*](http://db.yeastgenome.org/cgi-bin/locus.pl?locus=CHS5)*,* [*DBR1*](http://db.yeastgenome.org/cgi-bin/locus.pl?locus=DBR1)*,* [*DHH1*](http://db.yeastgenome.org/cgi-bin/locus.pl?locus=DHH1)*,* [*DOA1*](http://db.yeastgenome.org/cgi-bin/locus.pl?locus=DOA1)*,* [*DSK2*](http://db.yeastgenome.org/cgi-bin/locus.pl?locus=DSK2)*,* [*HRD3*](http://db.yeastgenome.org/cgi-bin/locus.pl?locus=HRD3)*, LAP2,* [*MET30*](http://db.yeastgenome.org/cgi-bin/locus.pl?locus=MET30)*,* [*MSS11*](http://db.yeastgenome.org/cgi-bin/locus.pl?locus=MSS11)*,* [*NAS2*](http://db.yeastgenome.org/cgi-bin/locus.pl?locus=NAS2)*,* [*PNG1*](http://db.yeastgenome.org/cgi-bin/locus.pl?locus=PNG1)*,* [*PRE4*](http://db.yeastgenome.org/cgi-bin/locus.pl?locus=PRE4)*,* [*PRE5*](http://db.yeastgenome.org/cgi-bin/locus.pl?locus=PRE5)*,* [*PRE7*](http://db.yeastgenome.org/cgi-bin/locus.pl?locus=PRE7)*,* [*PUP1*](http://db.yeastgenome.org/cgi-bin/locus.pl?locus=PUP1)*,* [*PUP2*](http://db.yeastgenome.org/cgi-bin/locus.pl?locus=PUP2)*,* [*PUP3*](http://db.yeastgenome.org/cgi-bin/locus.pl?locus=PUP3)*,* [*QRI8*](http://db.yeastgenome.org/cgi-bin/locus.pl?locus=QRI8)*,* [*RAD1*](http://db.yeastgenome.org/cgi-bin/locus.pl?locus=RAD1)*,* [*RPN1*](http://db.yeastgenome.org/cgi-bin/locus.pl?locus=RPN1)*,* [*RPN10*](http://db.yeastgenome.org/cgi-bin/locus.pl?locus=RPN10)*,* [*RPN14*](http://db.yeastgenome.org/cgi-bin/locus.pl?locus=RPN14)*,* [*RPT1*](http://db.yeastgenome.org/cgi-bin/locus.pl?locus=RPT1)*,* [*RTT101*](http://db.yeastgenome.org/cgi-bin/locus.pl?locus=RTT101)*,* [*SCL1*](http://db.yeastgenome.org/cgi-bin/locus.pl?locus=SCL1)*,* [*SEM1*](http://db.yeastgenome.org/cgi-bin/locus.pl?locus=SEM1)*,* [*SHP1*](http://db.yeastgenome.org/cgi-bin/locus.pl?locus=SHP1)*,* [*SKP1*](http://db.yeastgenome.org/cgi-bin/locus.pl?locus=SKP1)*,* [*SNF7*](http://db.yeastgenome.org/cgi-bin/locus.pl?locus=SNF7)*,* [*STS1*](http://db.yeastgenome.org/cgi-bin/locus.pl?locus=STS1)*,* [*SWM1*](http://db.yeastgenome.org/cgi-bin/locus.pl?locus=SWM1)*,* [*TUL1*](http://db.yeastgenome.org/cgi-bin/locus.pl?locus=TUL1)*,* [*UFO1*](http://db.yeastgenome.org/cgi-bin/locus.pl?locus=UFO1)*,* [*VID28*](http://db.yeastgenome.org/cgi-bin/locus.pl?locus=VID28)*,* [*YBL055C*](http://db.yeastgenome.org/cgi-bin/locus.pl?locus=YBL055C)*,* [*YDR306C*](http://db.yeastgenome.org/cgi-bin/locus.pl?locus=YDR306C)*,* [*YTA7*](http://db.yeastgenome.org/cgi-bin/locus.pl?locus=YTA7) |

**Table S3-B. Genes differentially down-regulated in CHP-induced stress (not down-regulated in H_2_O_2_**).

| **Gene Ontology term (AMIGO)** | **Cluster**  **frequency** | **p-value** | **Genes annotated to the term** |
| --- | --- | --- | --- |
| [Oxidative phosphorylation](http://db.yeastgenome.org/cgi-bin/GO/go.pl?goid=6119) | 15 out of 146 genes, 10.3% | 3.25e-12 | [*ATP14*](http://db.yeastgenome.org/cgi-bin/locus.pl?locus=ATP14)*,* [*ATP15*](http://db.yeastgenome.org/cgi-bin/locus.pl?locus=ATP15)*,* [*ATP20*](http://db.yeastgenome.org/cgi-bin/locus.pl?locus=ATP20)*,* [*COX6*](http://db.yeastgenome.org/cgi-bin/locus.pl?locus=COX6)*,* [*COX7*](http://db.yeastgenome.org/cgi-bin/locus.pl?locus=COX7)*,* [*COX8*](http://db.yeastgenome.org/cgi-bin/locus.pl?locus=COX8)*,* [*COX9*](http://db.yeastgenome.org/cgi-bin/locus.pl?locus=COX9)*,* [*CYT1*](http://db.yeastgenome.org/cgi-bin/locus.pl?locus=CYT1)*,* [*QCR2*](http://db.yeastgenome.org/cgi-bin/locus.pl?locus=QCR2)*,* [*QCR6*](http://db.yeastgenome.org/cgi-bin/locus.pl?locus=QCR6)*,* [*QCR7*](http://db.yeastgenome.org/cgi-bin/locus.pl?locus=QCR7)*,* [*QCR9*](http://db.yeastgenome.org/cgi-bin/locus.pl?locus=QCR9)*,* [*RIP1*](http://db.yeastgenome.org/cgi-bin/locus.pl?locus=RIP1)*,* [*SDH2*](http://db.yeastgenome.org/cgi-bin/locus.pl?locus=SDH2)*,* [*SDH3*](http://db.yeastgenome.org/cgi-bin/locus.pl?locus=SDH3) |
| [ATP synthesis coupled electron transport](http://db.yeastgenome.org/cgi-bin/GO/go.pl?goid=42773) | 12 out of 146 genes, 8.2% | 7.35e-12 | [*COX6*](http://db.yeastgenome.org/cgi-bin/locus.pl?locus=COX6)*,* [*COX7*](http://db.yeastgenome.org/cgi-bin/locus.pl?locus=COX7)*,* [*COX8*](http://db.yeastgenome.org/cgi-bin/locus.pl?locus=COX8)*,* [*COX9*](http://db.yeastgenome.org/cgi-bin/locus.pl?locus=COX9)*,* [*CYT1*](http://db.yeastgenome.org/cgi-bin/locus.pl?locus=CYT1)*,* [*QCR2*](http://db.yeastgenome.org/cgi-bin/locus.pl?locus=QCR2)*,* [*QCR6*](http://db.yeastgenome.org/cgi-bin/locus.pl?locus=QCR6)*,* [*QCR7*](http://db.yeastgenome.org/cgi-bin/locus.pl?locus=QCR7)*,* [*QCR9*](http://db.yeastgenome.org/cgi-bin/locus.pl?locus=QCR9)*,* [*RIP1*](http://db.yeastgenome.org/cgi-bin/locus.pl?locus=RIP1)*,* [*SDH2*](http://db.yeastgenome.org/cgi-bin/locus.pl?locus=SDH2)*,* [*SDH3*](http://db.yeastgenome.org/cgi-bin/locus.pl?locus=SDH3) |
| [organelle ATP synthesis coupled electron transport](http://db.yeastgenome.org/cgi-bin/GO/go.pl?goid=42775) | 12 out of 146 genes, 8.2% | 7.35e-12 | [*COX6*](http://db.yeastgenome.org/cgi-bin/locus.pl?locus=COX6)*,* [*COX7*](http://db.yeastgenome.org/cgi-bin/locus.pl?locus=COX7)*,* [*COX8*](http://db.yeastgenome.org/cgi-bin/locus.pl?locus=COX8)*,* [*COX9*](http://db.yeastgenome.org/cgi-bin/locus.pl?locus=COX9)*,* [*CYT1*](http://db.yeastgenome.org/cgi-bin/locus.pl?locus=CYT1)*,* [*QCR2*](http://db.yeastgenome.org/cgi-bin/locus.pl?locus=QCR2)*,* [*QCR6*](http://db.yeastgenome.org/cgi-bin/locus.pl?locus=QCR6)*,* [*QCR7*](http://db.yeastgenome.org/cgi-bin/locus.pl?locus=QCR7)*,* [*QCR9*](http://db.yeastgenome.org/cgi-bin/locus.pl?locus=QCR9)*,* [*RIP1*](http://db.yeastgenome.org/cgi-bin/locus.pl?locus=RIP1)*,* [*SDH2*](http://db.yeastgenome.org/cgi-bin/locus.pl?locus=SDH2)*,* [*SDH3*](http://db.yeastgenome.org/cgi-bin/locus.pl?locus=SDH3) |
| Electron transport | 13 out of 146 genes, 8.9% | 1.32e-11 | [*COX6*](http://db.yeastgenome.org/cgi-bin/locus.pl?locus=COX6)*,* [*COX7*](http://db.yeastgenome.org/cgi-bin/locus.pl?locus=COX7)*,* [*COX8*](http://db.yeastgenome.org/cgi-bin/locus.pl?locus=COX8)*,* [*COX9*](http://db.yeastgenome.org/cgi-bin/locus.pl?locus=COX9)*,* [*CYT1*](http://db.yeastgenome.org/cgi-bin/locus.pl?locus=CYT1)*,* [*MCR1*](http://db.yeastgenome.org/cgi-bin/locus.pl?locus=MCR1)*,* [*QCR2*](http://db.yeastgenome.org/cgi-bin/locus.pl?locus=QCR2)*,* [*QCR6*](http://db.yeastgenome.org/cgi-bin/locus.pl?locus=QCR6)*,* [*QCR7*](http://db.yeastgenome.org/cgi-bin/locus.pl?locus=QCR7)*,* [*QCR9*](http://db.yeastgenome.org/cgi-bin/locus.pl?locus=QCR9)*,* [*RIP1*](http://db.yeastgenome.org/cgi-bin/locus.pl?locus=RIP1)*,* [*SDH2*](http://db.yeastgenome.org/cgi-bin/locus.pl?locus=SDH2)*,* [*SDH3*](http://db.yeastgenome.org/cgi-bin/locus.pl?locus=SDH3) |
| Generation of precursor metabolites and energy | 24 out of 146 genes, 16.4% | 5.48e-11 | [*ADE16*](http://db.yeastgenome.org/cgi-bin/locus.pl?locus=ADE16)*,* [*ATP14*](http://db.yeastgenome.org/cgi-bin/locus.pl?locus=ATP14)*,* [*ATP15*](http://db.yeastgenome.org/cgi-bin/locus.pl?locus=ATP15)*,* [*ATP20*](http://db.yeastgenome.org/cgi-bin/locus.pl?locus=ATP20)*,* [*COX16*](http://db.yeastgenome.org/cgi-bin/locus.pl?locus=COX16)*,* [*COX6*](http://db.yeastgenome.org/cgi-bin/locus.pl?locus=COX6)*,* [*COX7*](http://db.yeastgenome.org/cgi-bin/locus.pl?locus=COX7)*,* [*COX8*](http://db.yeastgenome.org/cgi-bin/locus.pl?locus=COX8)*,* [*COX9*](http://db.yeastgenome.org/cgi-bin/locus.pl?locus=COX9)*,* [*CYT1*](http://db.yeastgenome.org/cgi-bin/locus.pl?locus=CYT1)*,* [*IDH1*](http://db.yeastgenome.org/cgi-bin/locus.pl?locus=IDH1)*,* [*IDH2*](http://db.yeastgenome.org/cgi-bin/locus.pl?locus=IDH2)*,* [*MCR1*](http://db.yeastgenome.org/cgi-bin/locus.pl?locus=MCR1)*,* [*PCL8*](http://db.yeastgenome.org/cgi-bin/locus.pl?locus=PCL8)*,* [*PDC2*](http://db.yeastgenome.org/cgi-bin/locus.pl?locus=PDC2)*,* [*PDC5*](http://db.yeastgenome.org/cgi-bin/locus.pl?locus=PDC5)*,* [*PET309*](http://db.yeastgenome.org/cgi-bin/locus.pl?locus=PET309)*,* [*QCR2*](http://db.yeastgenome.org/cgi-bin/locus.pl?locus=QCR2)*,* [*QCR6*](http://db.yeastgenome.org/cgi-bin/locus.pl?locus=QCR6)*,* [*QCR7*](http://db.yeastgenome.org/cgi-bin/locus.pl?locus=QCR7)*,* [*QCR9*](http://db.yeastgenome.org/cgi-bin/locus.pl?locus=QCR9)*,* [*RIP1*](http://db.yeastgenome.org/cgi-bin/locus.pl?locus=RIP1)*,* [*SDH2*](http://db.yeastgenome.org/cgi-bin/locus.pl?locus=SDH2)*,* [*SDH3*](http://db.yeastgenome.org/cgi-bin/locus.pl?locus=SDH3) |
| Phosphorylation | 19 out of 146 genes, 13% | 6.63e-8 | [*ATP14*](http://db.yeastgenome.org/cgi-bin/locus.pl?locus=ATP14)*,* [*ATP15*](http://db.yeastgenome.org/cgi-bin/locus.pl?locus=ATP15)*,* [*ATP20*](http://db.yeastgenome.org/cgi-bin/locus.pl?locus=ATP20)*,* [*BUB1*](http://db.yeastgenome.org/cgi-bin/locus.pl?locus=BUB1)*,* [*CKB2*](http://db.yeastgenome.org/cgi-bin/locus.pl?locus=CKB2)*,* [*COX6*](http://db.yeastgenome.org/cgi-bin/locus.pl?locus=COX6)*,* [*COX7*](http://db.yeastgenome.org/cgi-bin/locus.pl?locus=COX7)*,* [*COX8*](http://db.yeastgenome.org/cgi-bin/locus.pl?locus=COX8)*,* [*COX9*](http://db.yeastgenome.org/cgi-bin/locus.pl?locus=COX9)*,* [*CYT1*](http://db.yeastgenome.org/cgi-bin/locus.pl?locus=CYT1)*,* [*ELM1*](http://db.yeastgenome.org/cgi-bin/locus.pl?locus=ELM1)*,* [*QCR2*](http://db.yeastgenome.org/cgi-bin/locus.pl?locus=QCR2)*,* [*QCR6*](http://db.yeastgenome.org/cgi-bin/locus.pl?locus=QCR6)*,* [*QCR7*](http://db.yeastgenome.org/cgi-bin/locus.pl?locus=QCR7)*,* [*QCR9*](http://db.yeastgenome.org/cgi-bin/locus.pl?locus=QCR9)*,* [*RIP1*](http://db.yeastgenome.org/cgi-bin/locus.pl?locus=RIP1)*,* [*SDH2*](http://db.yeastgenome.org/cgi-bin/locus.pl?locus=SDH2)*,* [*SDH3*](http://db.yeastgenome.org/cgi-bin/locus.pl?locus=SDH3)*,* [*YPK1*](http://db.yeastgenome.org/cgi-bin/locus.pl?locus=YPK1) |
| Phosphorus metabolic process | 20 out of 146 genes, 13.7% | 1.77e-6 | [*ATP14*](http://db.yeastgenome.org/cgi-bin/locus.pl?locus=ATP14)*,* [*ATP15*](http://db.yeastgenome.org/cgi-bin/locus.pl?locus=ATP15)*,* [*ATP20*](http://db.yeastgenome.org/cgi-bin/locus.pl?locus=ATP20)*,* [*BUB1*](http://db.yeastgenome.org/cgi-bin/locus.pl?locus=BUB1)*,* [*CKB2*](http://db.yeastgenome.org/cgi-bin/locus.pl?locus=CKB2)*,* [*COX6*](http://db.yeastgenome.org/cgi-bin/locus.pl?locus=COX6)*,* [*COX7*](http://db.yeastgenome.org/cgi-bin/locus.pl?locus=COX7)*,* [*COX8*](http://db.yeastgenome.org/cgi-bin/locus.pl?locus=COX8)*,* [*COX9*](http://db.yeastgenome.org/cgi-bin/locus.pl?locus=COX9)*,* [*CYT1*](http://db.yeastgenome.org/cgi-bin/locus.pl?locus=CYT1)*,* [*ELM1*](http://db.yeastgenome.org/cgi-bin/locus.pl?locus=ELM1)*,* [*PHO13*](http://db.yeastgenome.org/cgi-bin/locus.pl?locus=PHO13)*,* [*QCR2*](http://db.yeastgenome.org/cgi-bin/locus.pl?locus=QCR2)*,* [*QCR6*](http://db.yeastgenome.org/cgi-bin/locus.pl?locus=QCR6)*,* [*QCR7*](http://db.yeastgenome.org/cgi-bin/locus.pl?locus=QCR7)*,* [*QCR9*](http://db.yeastgenome.org/cgi-bin/locus.pl?locus=QCR9)*,* [*RIP1*](http://db.yeastgenome.org/cgi-bin/locus.pl?locus=RIP1)*,* [*SDH2*](http://db.yeastgenome.org/cgi-bin/locus.pl?locus=SDH2)*,* [*SDH3*](http://db.yeastgenome.org/cgi-bin/locus.pl?locus=SDH3)*,* [*YPK1*](http://db.yeastgenome.org/cgi-bin/locus.pl?locus=YPK1) |
| Phosphate metabolic process | 20 out of 146 genes, 13.7% | 1.77e-6 | [*ATP14*](http://db.yeastgenome.org/cgi-bin/locus.pl?locus=ATP14)*,* [*ATP15*](http://db.yeastgenome.org/cgi-bin/locus.pl?locus=ATP15)*,* [*ATP20*](http://db.yeastgenome.org/cgi-bin/locus.pl?locus=ATP20)*,* [*BUB1*](http://db.yeastgenome.org/cgi-bin/locus.pl?locus=BUB1)*,* [*CKB2*](http://db.yeastgenome.org/cgi-bin/locus.pl?locus=CKB2)*,* [*COX6*](http://db.yeastgenome.org/cgi-bin/locus.pl?locus=COX6)*,* [*COX7*](http://db.yeastgenome.org/cgi-bin/locus.pl?locus=COX7)*,* [*COX8*](http://db.yeastgenome.org/cgi-bin/locus.pl?locus=COX8)*,* [*COX9*](http://db.yeastgenome.org/cgi-bin/locus.pl?locus=COX9)*,* [*CYT1*](http://db.yeastgenome.org/cgi-bin/locus.pl?locus=CYT1)*,* [*ELM1*](http://db.yeastgenome.org/cgi-bin/locus.pl?locus=ELM1)*,* [*PHO13*](http://db.yeastgenome.org/cgi-bin/locus.pl?locus=PHO13)*,* [*QCR2*](http://db.yeastgenome.org/cgi-bin/locus.pl?locus=QCR2)*,* [*QCR6*](http://db.yeastgenome.org/cgi-bin/locus.pl?locus=QCR6)*,* [*QCR7*](http://db.yeastgenome.org/cgi-bin/locus.pl?locus=QCR7)*,* [*QCR9*](http://db.yeastgenome.org/cgi-bin/locus.pl?locus=QCR9)*,* [*RIP1*](http://db.yeastgenome.org/cgi-bin/locus.pl?locus=RIP1)*,* [*SDH2*](http://db.yeastgenome.org/cgi-bin/locus.pl?locus=SDH2)*,* [*SDH3*](http://db.yeastgenome.org/cgi-bin/locus.pl?locus=SDH3)*,* [*YPK1*](http://db.yeastgenome.org/cgi-bin/locus.pl?locus=YPK1) |
| Mitochondrial electron transport, ubiquinol to cytochrome c | 6 out of 146 genes, 4.1% | 3.11e-6 | [*CYT1*](http://db.yeastgenome.org/cgi-bin/locus.pl?locus=CYT1)*,* [*QCR2*](http://db.yeastgenome.org/cgi-bin/locus.pl?locus=QCR2)*,* [*QCR6*](http://db.yeastgenome.org/cgi-bin/locus.pl?locus=QCR6)*,* [*QCR7*](http://db.yeastgenome.org/cgi-bin/locus.pl?locus=QCR7)*,* [*QCR9*](http://db.yeastgenome.org/cgi-bin/locus.pl?locus=QCR9)*,* [*RIP1*](http://db.yeastgenome.org/cgi-bin/locus.pl?locus=RIP1) |
| Aerobic respiration | 12 out of 146 genes, 8.2% | 4.40e-5 | [*ADE16*](http://db.yeastgenome.org/cgi-bin/locus.pl?locus=ADE16)*,* [*COX16*](http://db.yeastgenome.org/cgi-bin/locus.pl?locus=COX16)*,* [*IDH1*](http://db.yeastgenome.org/cgi-bin/locus.pl?locus=IDH1)*,* [*IDH2*](http://db.yeastgenome.org/cgi-bin/locus.pl?locus=IDH2)*,* [*PET309*](http://db.yeastgenome.org/cgi-bin/locus.pl?locus=PET309)*,* [*QCR2*](http://db.yeastgenome.org/cgi-bin/locus.pl?locus=QCR2)*,* [*QCR6*](http://db.yeastgenome.org/cgi-bin/locus.pl?locus=QCR6)*,* [*QCR7*](http://db.yeastgenome.org/cgi-bin/locus.pl?locus=QCR7)*,* [*QCR9*](http://db.yeastgenome.org/cgi-bin/locus.pl?locus=QCR9)*,* [*RIP1*](http://db.yeastgenome.org/cgi-bin/locus.pl?locus=RIP1)*,* [*SDH2*](http://db.yeastgenome.org/cgi-bin/locus.pl?locus=SDH2)*,* [*SDH3*](http://db.yeastgenome.org/cgi-bin/locus.pl?locus=SDH3) |
| Energy derivation by oxidation of organic compounds | 15 out of 146 genes, 10.3% | 8.42e-5 | [*ADE16*](http://db.yeastgenome.org/cgi-bin/locus.pl?locus=ADE16)*,* [*COX16*](http://db.yeastgenome.org/cgi-bin/locus.pl?locus=COX16)*,* [*IDH1*](http://db.yeastgenome.org/cgi-bin/locus.pl?locus=IDH1)*,* [*IDH2*](http://db.yeastgenome.org/cgi-bin/locus.pl?locus=IDH2)*,* [*PCL8*](http://db.yeastgenome.org/cgi-bin/locus.pl?locus=PCL8)*,* [*PDC2*](http://db.yeastgenome.org/cgi-bin/locus.pl?locus=PDC2)*,* [*PDC5*](http://db.yeastgenome.org/cgi-bin/locus.pl?locus=PDC5)*,* [*PET309*](http://db.yeastgenome.org/cgi-bin/locus.pl?locus=PET309)*,* [*QCR2*](http://db.yeastgenome.org/cgi-bin/locus.pl?locus=QCR2)*,* [*QCR6*](http://db.yeastgenome.org/cgi-bin/locus.pl?locus=QCR6)*,* [*QCR7*](http://db.yeastgenome.org/cgi-bin/locus.pl?locus=QCR7)*,* [*QCR9*](http://db.yeastgenome.org/cgi-bin/locus.pl?locus=QCR9)*,* [*RIP1*](http://db.yeastgenome.org/cgi-bin/locus.pl?locus=RIP1)*,* [*SDH2*](http://db.yeastgenome.org/cgi-bin/locus.pl?locus=SDH2)*,* [*SDH3*](http://db.yeastgenome.org/cgi-bin/locus.pl?locus=SDH3) |
| Cellular respiration | 12 out of 146 genes, 8.2% | 9.85e-5 | [*ADE16*](http://db.yeastgenome.org/cgi-bin/locus.pl?locus=ADE16)*,* [*COX16*](http://db.yeastgenome.org/cgi-bin/locus.pl?locus=COX16)*,* [*IDH1*](http://db.yeastgenome.org/cgi-bin/locus.pl?locus=IDH1)*,* [*IDH2*](http://db.yeastgenome.org/cgi-bin/locus.pl?locus=IDH2)*,* [*PET309*](http://db.yeastgenome.org/cgi-bin/locus.pl?locus=PET309)*,* [*QCR2*](http://db.yeastgenome.org/cgi-bin/locus.pl?locus=QCR2)*,* [*QCR6*](http://db.yeastgenome.org/cgi-bin/locus.pl?locus=QCR6)*,* [*QCR7*](http://db.yeastgenome.org/cgi-bin/locus.pl?locus=QCR7)*,* [*QCR9*](http://db.yeastgenome.org/cgi-bin/locus.pl?locus=QCR9)*,* [*RIP1*](http://db.yeastgenome.org/cgi-bin/locus.pl?locus=RIP1)*,* [*SDH2*](http://db.yeastgenome.org/cgi-bin/locus.pl?locus=SDH2)*,* [*SDH3*](http://db.yeastgenome.org/cgi-bin/locus.pl?locus=SDH3) |
| Cellular process | 122 out of 146 genes, 83.6% | 0.00010 | [*ADE16*](http://db.yeastgenome.org/cgi-bin/locus.pl?locus=ADE16)*,* [*ADE2*](http://db.yeastgenome.org/cgi-bin/locus.pl?locus=ADE2)*,* [*APC4*](http://db.yeastgenome.org/cgi-bin/locus.pl?locus=APC4)*,* [*ARG3*](http://db.yeastgenome.org/cgi-bin/locus.pl?locus=ARG3)*,* [*ARP3*](http://db.yeastgenome.org/cgi-bin/locus.pl?locus=ARP3)*,* [*ASN1*](http://db.yeastgenome.org/cgi-bin/locus.pl?locus=ASN1)*,* [*ATP14*](http://db.yeastgenome.org/cgi-bin/locus.pl?locus=ATP14)*,* [*ATP15*](http://db.yeastgenome.org/cgi-bin/locus.pl?locus=ATP15)*,* [*ATP20*](http://db.yeastgenome.org/cgi-bin/locus.pl?locus=ATP20)*,* [*BNI5*](http://db.yeastgenome.org/cgi-bin/locus.pl?locus=BNI5)*,* [*BUB1*](http://db.yeastgenome.org/cgi-bin/locus.pl?locus=BUB1)*,* [*BUD31*](http://db.yeastgenome.org/cgi-bin/locus.pl?locus=BUD31)*,* [*CBC2*](http://db.yeastgenome.org/cgi-bin/locus.pl?locus=CBC2)*,* [*CCC1*](http://db.yeastgenome.org/cgi-bin/locus.pl?locus=CCC1)*,* [*CHL4*](http://db.yeastgenome.org/cgi-bin/locus.pl?locus=CHL4)*,* [*CIN2*](http://db.yeastgenome.org/cgi-bin/locus.pl?locus=CIN2)*,* [*CIN8*](http://db.yeastgenome.org/cgi-bin/locus.pl?locus=CIN8)*,* [*CKB2*](http://db.yeastgenome.org/cgi-bin/locus.pl?locus=CKB2)*,* [*CNA1*](http://db.yeastgenome.org/cgi-bin/locus.pl?locus=CNA1)*,* [*COX12*](http://db.yeastgenome.org/cgi-bin/locus.pl?locus=COX12)*,* [*COX16*](http://db.yeastgenome.org/cgi-bin/locus.pl?locus=COX16)*,* [*COX6*](http://db.yeastgenome.org/cgi-bin/locus.pl?locus=COX6)*,* [*COX7*](http://db.yeastgenome.org/cgi-bin/locus.pl?locus=COX7)*,* [*COX8*](http://db.yeastgenome.org/cgi-bin/locus.pl?locus=COX8)*,* [*COX9*](http://db.yeastgenome.org/cgi-bin/locus.pl?locus=COX9)*,* [*CPR1*](http://db.yeastgenome.org/cgi-bin/locus.pl?locus=CPR1)*,* [*CSE4*](http://db.yeastgenome.org/cgi-bin/locus.pl?locus=CSE4)*,* [*CSM2*](http://db.yeastgenome.org/cgi-bin/locus.pl?locus=CSM2)*,* [*CSR1*](http://db.yeastgenome.org/cgi-bin/locus.pl?locus=CSR1)*,* [*CTF4*](http://db.yeastgenome.org/cgi-bin/locus.pl?locus=CTF4)*,* [*CYT1*](http://db.yeastgenome.org/cgi-bin/locus.pl?locus=CYT1)*,* [*DCP1*](http://db.yeastgenome.org/cgi-bin/locus.pl?locus=DCP1)*,* [*DED1*](http://db.yeastgenome.org/cgi-bin/locus.pl?locus=DED1)*,* [*DOM34*](http://db.yeastgenome.org/cgi-bin/locus.pl?locus=DOM34)*,* [*ELM1*](http://db.yeastgenome.org/cgi-bin/locus.pl?locus=ELM1)*,* [*ELP6*](http://db.yeastgenome.org/cgi-bin/locus.pl?locus=ELP6)*,* [*FUS2*](http://db.yeastgenome.org/cgi-bin/locus.pl?locus=FUS2)*,* [*GBP2*](http://db.yeastgenome.org/cgi-bin/locus.pl?locus=GBP2)*,* [*GPD2*](http://db.yeastgenome.org/cgi-bin/locus.pl?locus=GPD2)*,* [*GPI15*](http://db.yeastgenome.org/cgi-bin/locus.pl?locus=GPI15)*,* [*HEM4*](http://db.yeastgenome.org/cgi-bin/locus.pl?locus=HEM4)*,* [*IDH1*](http://db.yeastgenome.org/cgi-bin/locus.pl?locus=IDH1)*,* [*IDH2*](http://db.yeastgenome.org/cgi-bin/locus.pl?locus=IDH2)*,* [*IDI1*](http://db.yeastgenome.org/cgi-bin/locus.pl?locus=IDI1)*,* [*INP2*](http://db.yeastgenome.org/cgi-bin/locus.pl?locus=INP2)*,* [*JIP5*](http://db.yeastgenome.org/cgi-bin/locus.pl?locus=JIP5)*,* [*KEL2*](http://db.yeastgenome.org/cgi-bin/locus.pl?locus=KEL2)*,* [*KIN2*](http://db.yeastgenome.org/cgi-bin/locus.pl?locus=KIN2)*,* [*KNH1*](http://db.yeastgenome.org/cgi-bin/locus.pl?locus=KNH1)*,* [*KTR4*](http://db.yeastgenome.org/cgi-bin/locus.pl?locus=KTR4)*,* [*LRG1*](http://db.yeastgenome.org/cgi-bin/locus.pl?locus=LRG1)*,* [*LTE1*](http://db.yeastgenome.org/cgi-bin/locus.pl?locus=LTE1)*,* [*LYS1*](http://db.yeastgenome.org/cgi-bin/locus.pl?locus=LYS1)*,* [*MCR1*](http://db.yeastgenome.org/cgi-bin/locus.pl?locus=MCR1)*,* [*MED1*](http://db.yeastgenome.org/cgi-bin/locus.pl?locus=MED1)*,* [*MRPS18*](http://db.yeastgenome.org/cgi-bin/locus.pl?locus=MRPS18)*,* [*MSH3*](http://db.yeastgenome.org/cgi-bin/locus.pl?locus=MSH3)*,* [*MSH5*](http://db.yeastgenome.org/cgi-bin/locus.pl?locus=MSH5)*,* [*MTQ2*](http://db.yeastgenome.org/cgi-bin/locus.pl?locus=MTQ2)*,* [*MTW1*](http://db.yeastgenome.org/cgi-bin/locus.pl?locus=MTW1)*,* [*MUQ1*](http://db.yeastgenome.org/cgi-bin/locus.pl?locus=MUQ1)*,* [*NAT4*](http://db.yeastgenome.org/cgi-bin/locus.pl?locus=NAT4)*,* [*NHX1*](http://db.yeastgenome.org/cgi-bin/locus.pl?locus=NHX1)*,* [*NMA1*](http://db.yeastgenome.org/cgi-bin/locus.pl?locus=NMA1)*,* [*NMD5*](http://db.yeastgenome.org/cgi-bin/locus.pl?locus=NMD5)*,* [*NUF2*](http://db.yeastgenome.org/cgi-bin/locus.pl?locus=NUF2)*,* [*NUP53*](http://db.yeastgenome.org/cgi-bin/locus.pl?locus=NUP53)*,* [*OST1*](http://db.yeastgenome.org/cgi-bin/locus.pl?locus=OST1)*,* [*PAC11*](http://db.yeastgenome.org/cgi-bin/locus.pl?locus=PAC11)*,* [*PCL2*](http://db.yeastgenome.org/cgi-bin/locus.pl?locus=PCL2)*,* [*PCL8*](http://db.yeastgenome.org/cgi-bin/locus.pl?locus=PCL8)*,* [*PDC2*](http://db.yeastgenome.org/cgi-bin/locus.pl?locus=PDC2)*,* [*PDC5*](http://db.yeastgenome.org/cgi-bin/locus.pl?locus=PDC5)*,* [*PDS1*](http://db.yeastgenome.org/cgi-bin/locus.pl?locus=PDS1)*,* [*PEP3*](http://db.yeastgenome.org/cgi-bin/locus.pl?locus=PEP3)*,* [*PET309*](http://db.yeastgenome.org/cgi-bin/locus.pl?locus=PET309)*,* [*PEX21*](http://db.yeastgenome.org/cgi-bin/locus.pl?locus=PEX21)*,* [*PFK27*](http://db.yeastgenome.org/cgi-bin/locus.pl?locus=PFK27)*,* [*PHO13*](http://db.yeastgenome.org/cgi-bin/locus.pl?locus=PHO13)*,* [*PIS1*](http://db.yeastgenome.org/cgi-bin/locus.pl?locus=PIS1)*,* [*PLM2*](http://db.yeastgenome.org/cgi-bin/locus.pl?locus=PLM2)*,* [*PRI2*](http://db.yeastgenome.org/cgi-bin/locus.pl?locus=PRI2)*,* [*PRP31*](http://db.yeastgenome.org/cgi-bin/locus.pl?locus=PRP31)*,* [*QCR2*](http://db.yeastgenome.org/cgi-bin/locus.pl?locus=QCR2)*,* [*QCR6*](http://db.yeastgenome.org/cgi-bin/locus.pl?locus=QCR6)*,* [*QCR7*](http://db.yeastgenome.org/cgi-bin/locus.pl?locus=QCR7)*,* [*QCR9*](http://db.yeastgenome.org/cgi-bin/locus.pl?locus=QCR9)*,* [*RCE1*](http://db.yeastgenome.org/cgi-bin/locus.pl?locus=RCE1)*,* [*RHO5*](http://db.yeastgenome.org/cgi-bin/locus.pl?locus=RHO5)*,* [*RIM1*](http://db.yeastgenome.org/cgi-bin/locus.pl?locus=RIM1)*,* [*RIP1*](http://db.yeastgenome.org/cgi-bin/locus.pl?locus=RIP1)*,* [*RNH70*](http://db.yeastgenome.org/cgi-bin/locus.pl?locus=RNH70)*,* [*RPB7*](http://db.yeastgenome.org/cgi-bin/locus.pl?locus=RPB7)*,* [*RPL22B*](http://db.yeastgenome.org/cgi-bin/locus.pl?locus=RPL22B)*,* [*RRP42*](http://db.yeastgenome.org/cgi-bin/locus.pl?locus=RRP42)*,* [*RSC2*](http://db.yeastgenome.org/cgi-bin/locus.pl?locus=RSC2)*,* [*SDH2*](http://db.yeastgenome.org/cgi-bin/locus.pl?locus=SDH2)*,* [*SDH3*](http://db.yeastgenome.org/cgi-bin/locus.pl?locus=SDH3)*,* [*SEC39*](http://db.yeastgenome.org/cgi-bin/locus.pl?locus=SEC39)*,* [*SKI6*](http://db.yeastgenome.org/cgi-bin/locus.pl?locus=SKI6)*,* [*SMC6*](http://db.yeastgenome.org/cgi-bin/locus.pl?locus=SMC6)*,* [*SNF12*](http://db.yeastgenome.org/cgi-bin/locus.pl?locus=SNF12)*,* [*SNT1*](http://db.yeastgenome.org/cgi-bin/locus.pl?locus=SNT1)*,* [*SNT309*](http://db.yeastgenome.org/cgi-bin/locus.pl?locus=SNT309)*,* [*SPC110*](http://db.yeastgenome.org/cgi-bin/locus.pl?locus=SPC110)*,* [*SPH1*](http://db.yeastgenome.org/cgi-bin/locus.pl?locus=SPH1)*,* [*SRP101*](http://db.yeastgenome.org/cgi-bin/locus.pl?locus=SRP101)*,* [*SSL1*](http://db.yeastgenome.org/cgi-bin/locus.pl?locus=SSL1)*,* [*SSP2*](http://db.yeastgenome.org/cgi-bin/locus.pl?locus=SSP2)*,* [*TAF4*](http://db.yeastgenome.org/cgi-bin/locus.pl?locus=TAF4)*,* [*TGL5*](http://db.yeastgenome.org/cgi-bin/locus.pl?locus=TGL5)*,* [*TOS2*](http://db.yeastgenome.org/cgi-bin/locus.pl?locus=TOS2)*,* [*TPT1*](http://db.yeastgenome.org/cgi-bin/locus.pl?locus=TPT1)*,* [*UBP8*](http://db.yeastgenome.org/cgi-bin/locus.pl?locus=UBP8)*,* [*UFE1*](http://db.yeastgenome.org/cgi-bin/locus.pl?locus=UFE1)*,* [*VCX1*](http://db.yeastgenome.org/cgi-bin/locus.pl?locus=VCX1)*,* [*VPS70*](http://db.yeastgenome.org/cgi-bin/locus.pl?locus=VPS70)*,* [*YLH47*](http://db.yeastgenome.org/cgi-bin/locus.pl?locus=YLH47)*,* [*YOR302W*](http://db.yeastgenome.org/cgi-bin/locus.pl?locus=YOR302W)*,* [*YPK1*](http://db.yeastgenome.org/cgi-bin/locus.pl?locus=YPK1)*,* [*YPL041C*](http://db.yeastgenome.org/cgi-bin/locus.pl?locus=YPL041C)*,* [*YPT7*](http://db.yeastgenome.org/cgi-bin/locus.pl?locus=YPT7) |

**Table S3-C. Genes up-regulated in CHP and H_2_O_2_**-induced stress.

| **Gene Ontology term (AMIGO)** | **Cluster**  **frequency** | **p-value** | **Genes annotated to the term** |
| --- | --- | --- | --- |
| Response to chemical stimulus | 63 out of 399 genes, 15.8% | 4.00e-13 | [*AAD15*](http://db.yeastgenome.org/cgi-bin/locus.pl?locus=AAD15)*,* [*AAD16*](http://db.yeastgenome.org/cgi-bin/locus.pl?locus=AAD16)*,* [*AAD4*](http://db.yeastgenome.org/cgi-bin/locus.pl?locus=AAD4)*,* [*AAD6*](http://db.yeastgenome.org/cgi-bin/locus.pl?locus=AAD6)*,* [*AFR1*](http://db.yeastgenome.org/cgi-bin/locus.pl?locus=AFR1)*,* [*AFT2*](http://db.yeastgenome.org/cgi-bin/locus.pl?locus=AFT2)*,* [*AIF1*](http://db.yeastgenome.org/cgi-bin/locus.pl?locus=AIF1)*,* [*ARR2*](http://db.yeastgenome.org/cgi-bin/locus.pl?locus=ARR2)*,* [*ATR1*](http://db.yeastgenome.org/cgi-bin/locus.pl?locus=ATR1)*,* [*CCP1*](http://db.yeastgenome.org/cgi-bin/locus.pl?locus=CCP1)*,* [*CIN5*](http://db.yeastgenome.org/cgi-bin/locus.pl?locus=CIN5)*,* [*CTT1*](http://db.yeastgenome.org/cgi-bin/locus.pl?locus=CTT1)*,* [*ECM4*](http://db.yeastgenome.org/cgi-bin/locus.pl?locus=ECM4)*,* [*FLR1*](http://db.yeastgenome.org/cgi-bin/locus.pl?locus=FLR1)*,* [*GAD1*](http://db.yeastgenome.org/cgi-bin/locus.pl?locus=GAD1)*,* [*GLO1*](http://db.yeastgenome.org/cgi-bin/locus.pl?locus=GLO1)*,* [*GLO4*](http://db.yeastgenome.org/cgi-bin/locus.pl?locus=GLO4)*,* [*GLR1*](http://db.yeastgenome.org/cgi-bin/locus.pl?locus=GLR1)*,* [*GPX1*](http://db.yeastgenome.org/cgi-bin/locus.pl?locus=GPX1)*,* [*GPX2*](http://db.yeastgenome.org/cgi-bin/locus.pl?locus=GPX2)*,* [*GRE2*](http://db.yeastgenome.org/cgi-bin/locus.pl?locus=GRE2)*,* [*GRX1*](http://db.yeastgenome.org/cgi-bin/locus.pl?locus=GRX1)*,* [*GRX2*](http://db.yeastgenome.org/cgi-bin/locus.pl?locus=GRX2)*,* [*GSH1*](http://db.yeastgenome.org/cgi-bin/locus.pl?locus=GSH1)*,* [*GTT2*](http://db.yeastgenome.org/cgi-bin/locus.pl?locus=GTT2)*,* [*HBT1*](http://db.yeastgenome.org/cgi-bin/locus.pl?locus=HBT1)*,* [*HSP12*](http://db.yeastgenome.org/cgi-bin/locus.pl?locus=HSP12)*,* [*ISA2*](http://db.yeastgenome.org/cgi-bin/locus.pl?locus=ISA2)*,* [*KIN82*](http://db.yeastgenome.org/cgi-bin/locus.pl?locus=KIN82)*,* [*LAP3*](http://db.yeastgenome.org/cgi-bin/locus.pl?locus=LAP3)*,* [*MDG1*](http://db.yeastgenome.org/cgi-bin/locus.pl?locus=MDG1)*,* [*MET16*](http://db.yeastgenome.org/cgi-bin/locus.pl?locus=MET16)*,* [*NCE103*](http://db.yeastgenome.org/cgi-bin/locus.pl?locus=NCE103)*,* [*OYE3*](http://db.yeastgenome.org/cgi-bin/locus.pl?locus=OYE3)*,* [*PRM5*](http://db.yeastgenome.org/cgi-bin/locus.pl?locus=PRM5)*,* [*PRM8*](http://db.yeastgenome.org/cgi-bin/locus.pl?locus=PRM8)*,* [*PRX1*](http://db.yeastgenome.org/cgi-bin/locus.pl?locus=PRX1)*,* [*QDR1*](http://db.yeastgenome.org/cgi-bin/locus.pl?locus=QDR1)*,* [*RDS1*](http://db.yeastgenome.org/cgi-bin/locus.pl?locus=RDS1)*,* [*RIB1*](http://db.yeastgenome.org/cgi-bin/locus.pl?locus=RIB1)*,* [*ROD1*](http://db.yeastgenome.org/cgi-bin/locus.pl?locus=ROD1)*,* [*RRI1*](http://db.yeastgenome.org/cgi-bin/locus.pl?locus=RRI1)*,* [*RRI2*](http://db.yeastgenome.org/cgi-bin/locus.pl?locus=RRI2)*,* [*RTA1*](http://db.yeastgenome.org/cgi-bin/locus.pl?locus=RTA1)*,* [*SDL1*](http://db.yeastgenome.org/cgi-bin/locus.pl?locus=SDL1)*,* [*SLT2*](http://db.yeastgenome.org/cgi-bin/locus.pl?locus=SLT2)*,* [*SOD1*](http://db.yeastgenome.org/cgi-bin/locus.pl?locus=SOD1)*,* [*SOD2*](http://db.yeastgenome.org/cgi-bin/locus.pl?locus=SOD2)*,* [*TPS1*](http://db.yeastgenome.org/cgi-bin/locus.pl?locus=TPS1)*,* [*TRR1*](http://db.yeastgenome.org/cgi-bin/locus.pl?locus=TRR1)*,* [*TRX2*](http://db.yeastgenome.org/cgi-bin/locus.pl?locus=TRX2)*,* [*TSA2*](http://db.yeastgenome.org/cgi-bin/locus.pl?locus=TSA2)*,* [*UBA4*](http://db.yeastgenome.org/cgi-bin/locus.pl?locus=UBA4)*,* [*UBC5*](http://db.yeastgenome.org/cgi-bin/locus.pl?locus=UBC5)*,* [*VPS60*](http://db.yeastgenome.org/cgi-bin/locus.pl?locus=VPS60)*,* [*YAP1*](http://db.yeastgenome.org/cgi-bin/locus.pl?locus=YAP1)*,* [*YCR102C*](http://db.yeastgenome.org/cgi-bin/locus.pl?locus=YCR102C)*,* [*YGP1*](http://db.yeastgenome.org/cgi-bin/locus.pl?locus=YGP1)*,* [*YIL029C*](http://db.yeastgenome.org/cgi-bin/locus.pl?locus=YIL029C)*,* [*YLL056C*](http://db.yeastgenome.org/cgi-bin/locus.pl?locus=YLL056C)*,* [*YLR346C*](http://db.yeastgenome.org/cgi-bin/locus.pl?locus=YLR346C)*,* [*YML131W*](http://db.yeastgenome.org/cgi-bin/locus.pl?locus=YML131W)*,* [*ZWF1*](http://db.yeastgenome.org/cgi-bin/locus.pl?locus=ZWF1) |
| Response to toxin | 16 out of 399 genes, 4.0% | 6.09e-11 | [*AAD15*](http://db.yeastgenome.org/cgi-bin/locus.pl?locus=AAD15)*,* [*AAD16*](http://db.yeastgenome.org/cgi-bin/locus.pl?locus=AAD16)*,* [*AAD4*](http://db.yeastgenome.org/cgi-bin/locus.pl?locus=AAD4)*,* [*AAD6*](http://db.yeastgenome.org/cgi-bin/locus.pl?locus=AAD6)*,* [*ECM4*](http://db.yeastgenome.org/cgi-bin/locus.pl?locus=ECM4)*,* [*FLR1*](http://db.yeastgenome.org/cgi-bin/locus.pl?locus=FLR1)*,* [*GLO1*](http://db.yeastgenome.org/cgi-bin/locus.pl?locus=GLO1)*,* [*GLO4*](http://db.yeastgenome.org/cgi-bin/locus.pl?locus=GLO4)*,* [*GRE2*](http://db.yeastgenome.org/cgi-bin/locus.pl?locus=GRE2)*,* [*GTT2*](http://db.yeastgenome.org/cgi-bin/locus.pl?locus=GTT2)*,* [*OYE3*](http://db.yeastgenome.org/cgi-bin/locus.pl?locus=OYE3)*,* [*RTA1*](http://db.yeastgenome.org/cgi-bin/locus.pl?locus=RTA1)*,* [*SDL1*](http://db.yeastgenome.org/cgi-bin/locus.pl?locus=SDL1)*,* [*YLL056C*](http://db.yeastgenome.org/cgi-bin/locus.pl?locus=YLL056C)*,* [*YLR346C*](http://db.yeastgenome.org/cgi-bin/locus.pl?locus=YLR346C)*,* [*YML131W*](http://db.yeastgenome.org/cgi-bin/locus.pl?locus=YML131W) |
| Response to oxidative stress | 22 out of 399 genes, 5.5% | 2.91e-9 | [*AFT2*](http://db.yeastgenome.org/cgi-bin/locus.pl?locus=AFT2)*,* [*AIF1*](http://db.yeastgenome.org/cgi-bin/locus.pl?locus=AIF1)*,* [*CCP1*](http://db.yeastgenome.org/cgi-bin/locus.pl?locus=CCP1)*,* [*CTT1*](http://db.yeastgenome.org/cgi-bin/locus.pl?locus=CTT1)*,* [*GAD1*](http://db.yeastgenome.org/cgi-bin/locus.pl?locus=GAD1)*,* [*GLR1*](http://db.yeastgenome.org/cgi-bin/locus.pl?locus=GLR1)*,* [*GPX1*](http://db.yeastgenome.org/cgi-bin/locus.pl?locus=GPX1)*,* [*GPX2*](http://db.yeastgenome.org/cgi-bin/locus.pl?locus=GPX2)*,* [*GRX1*](http://db.yeastgenome.org/cgi-bin/locus.pl?locus=GRX1)*,* [*GRX2*](http://db.yeastgenome.org/cgi-bin/locus.pl?locus=GRX2)*,* [*GSH1*](http://db.yeastgenome.org/cgi-bin/locus.pl?locus=GSH1)*,* [*HSP12*](http://db.yeastgenome.org/cgi-bin/locus.pl?locus=HSP12)*,* [*NCE103*](http://db.yeastgenome.org/cgi-bin/locus.pl?locus=NCE103)*,* [*PRX1*](http://db.yeastgenome.org/cgi-bin/locus.pl?locus=PRX1)*,* [*SOD1*](http://db.yeastgenome.org/cgi-bin/locus.pl?locus=SOD1)*,* [*SOD2*](http://db.yeastgenome.org/cgi-bin/locus.pl?locus=SOD2)*,* [*TRR1*](http://db.yeastgenome.org/cgi-bin/locus.pl?locus=TRR1)*,* [*TRX2*](http://db.yeastgenome.org/cgi-bin/locus.pl?locus=TRX2)*,* [*TSA2*](http://db.yeastgenome.org/cgi-bin/locus.pl?locus=TSA2)*,* [*UBA4*](http://db.yeastgenome.org/cgi-bin/locus.pl?locus=UBA4)*,* [*YAP1*](http://db.yeastgenome.org/cgi-bin/locus.pl?locus=YAP1)*,* [*ZWF1*](http://db.yeastgenome.org/cgi-bin/locus.pl?locus=ZWF1) |
| Oxygen and reactive oxygen species metabolic process | 22 out of 399 genes, 5.5% | 4.07e-9 | [*AFT2*](http://db.yeastgenome.org/cgi-bin/locus.pl?locus=AFT2)*,* [*AIF1*](http://db.yeastgenome.org/cgi-bin/locus.pl?locus=AIF1)*,* [*CCP1*](http://db.yeastgenome.org/cgi-bin/locus.pl?locus=CCP1)*,* [*CTT1*](http://db.yeastgenome.org/cgi-bin/locus.pl?locus=CTT1)*,* [*GAD1*](http://db.yeastgenome.org/cgi-bin/locus.pl?locus=GAD1)*,* [*GLR1*](http://db.yeastgenome.org/cgi-bin/locus.pl?locus=GLR1)*,* [*GPX1*](http://db.yeastgenome.org/cgi-bin/locus.pl?locus=GPX1)*,* [*GPX2*](http://db.yeastgenome.org/cgi-bin/locus.pl?locus=GPX2)*,* [*GRX1*](http://db.yeastgenome.org/cgi-bin/locus.pl?locus=GRX1)*,* [*GRX2*](http://db.yeastgenome.org/cgi-bin/locus.pl?locus=GRX2)*,* [*GSH1*](http://db.yeastgenome.org/cgi-bin/locus.pl?locus=GSH1)*,* [*HSP12*](http://db.yeastgenome.org/cgi-bin/locus.pl?locus=HSP12)*,* [*NCE103*](http://db.yeastgenome.org/cgi-bin/locus.pl?locus=NCE103)*,* [*PRX1*](http://db.yeastgenome.org/cgi-bin/locus.pl?locus=PRX1)*,* [*SOD1*](http://db.yeastgenome.org/cgi-bin/locus.pl?locus=SOD1)*,* [*SOD2*](http://db.yeastgenome.org/cgi-bin/locus.pl?locus=SOD2)*,* [*TRR1*](http://db.yeastgenome.org/cgi-bin/locus.pl?locus=TRR1)*,* [*TRX2*](http://db.yeastgenome.org/cgi-bin/locus.pl?locus=TRX2)*,* [*TSA2*](http://db.yeastgenome.org/cgi-bin/locus.pl?locus=TSA2)*,* [*UBA4*](http://db.yeastgenome.org/cgi-bin/locus.pl?locus=UBA4)*,* [*YAP1*](http://db.yeastgenome.org/cgi-bin/locus.pl?locus=YAP1)*,* [*ZWF1*](http://db.yeastgenome.org/cgi-bin/locus.pl?locus=ZWF1) |
| Response to stress | 61 out of 399 genes, 15.3% | 1.76e-7 | [*AFT2*](http://db.yeastgenome.org/cgi-bin/locus.pl?locus=AFT2)*,* [*AGP2*](http://db.yeastgenome.org/cgi-bin/locus.pl?locus=AGP2)*,* [*AIF1*](http://db.yeastgenome.org/cgi-bin/locus.pl?locus=AIF1)*,* [*ALD3*](http://db.yeastgenome.org/cgi-bin/locus.pl?locus=ALD3)*,* [*CCP1*](http://db.yeastgenome.org/cgi-bin/locus.pl?locus=CCP1)*,* [*CIN5*](http://db.yeastgenome.org/cgi-bin/locus.pl?locus=CIN5)*,* [*CTT1*](http://db.yeastgenome.org/cgi-bin/locus.pl?locus=CTT1)*,* [*DAK1*](http://db.yeastgenome.org/cgi-bin/locus.pl?locus=DAK1)*,* [*DDR48*](http://db.yeastgenome.org/cgi-bin/locus.pl?locus=DDR48)*,* [*GAD1*](http://db.yeastgenome.org/cgi-bin/locus.pl?locus=GAD1)*,* [*GCY1*](http://db.yeastgenome.org/cgi-bin/locus.pl?locus=GCY1)*,* [*GLR1*](http://db.yeastgenome.org/cgi-bin/locus.pl?locus=GLR1)*,* [*GPD1*](http://db.yeastgenome.org/cgi-bin/locus.pl?locus=GPD1)*,* [*GPX1*](http://db.yeastgenome.org/cgi-bin/locus.pl?locus=GPX1)*,* [*GPX2*](http://db.yeastgenome.org/cgi-bin/locus.pl?locus=GPX2)*,* [*GRE2*](http://db.yeastgenome.org/cgi-bin/locus.pl?locus=GRE2)*,* [*GRE3*](http://db.yeastgenome.org/cgi-bin/locus.pl?locus=GRE3)*,* [*GRX1*](http://db.yeastgenome.org/cgi-bin/locus.pl?locus=GRX1)*,* [*GRX2*](http://db.yeastgenome.org/cgi-bin/locus.pl?locus=GRX2)*,* [*GSH1*](http://db.yeastgenome.org/cgi-bin/locus.pl?locus=GSH1)*,* [*HAL1*](http://db.yeastgenome.org/cgi-bin/locus.pl?locus=HAL1)*,* [*HSP104*](http://db.yeastgenome.org/cgi-bin/locus.pl?locus=HSP104)*,* [*HSP12*](http://db.yeastgenome.org/cgi-bin/locus.pl?locus=HSP12)*,* [*HSP26*](http://db.yeastgenome.org/cgi-bin/locus.pl?locus=HSP26)*,* [*HSP30*](http://db.yeastgenome.org/cgi-bin/locus.pl?locus=HSP30)*,* [*HSP42*](http://db.yeastgenome.org/cgi-bin/locus.pl?locus=HSP42)*,* [*HSP82*](http://db.yeastgenome.org/cgi-bin/locus.pl?locus=HSP82)*,* [*LIF1*](http://db.yeastgenome.org/cgi-bin/locus.pl?locus=LIF1)*,* [*LSP1*](http://db.yeastgenome.org/cgi-bin/locus.pl?locus=LSP1)*,* [*NCE103*](http://db.yeastgenome.org/cgi-bin/locus.pl?locus=NCE103)*,* [*NTG1*](http://db.yeastgenome.org/cgi-bin/locus.pl?locus=NTG1)*,* [*NTH1*](http://db.yeastgenome.org/cgi-bin/locus.pl?locus=NTH1)*,* [*PEP4*](http://db.yeastgenome.org/cgi-bin/locus.pl?locus=PEP4)*,* [*PHR1*](http://db.yeastgenome.org/cgi-bin/locus.pl?locus=PHR1)*,* [*PRB1*](http://db.yeastgenome.org/cgi-bin/locus.pl?locus=PRB1)*,* [*PRX1*](http://db.yeastgenome.org/cgi-bin/locus.pl?locus=PRX1)*,* [*RAD16*](http://db.yeastgenome.org/cgi-bin/locus.pl?locus=RAD16)*,* [*RAD4*](http://db.yeastgenome.org/cgi-bin/locus.pl?locus=RAD4)*,* [*RAD50*](http://db.yeastgenome.org/cgi-bin/locus.pl?locus=RAD50)*,* [*RFX1*](http://db.yeastgenome.org/cgi-bin/locus.pl?locus=RFX1)*,* [*RIM11*](http://db.yeastgenome.org/cgi-bin/locus.pl?locus=RIM11)*,* [*SIP2*](http://db.yeastgenome.org/cgi-bin/locus.pl?locus=SIP2)*,* [*SLT2*](http://db.yeastgenome.org/cgi-bin/locus.pl?locus=SLT2)*,* [*SOD1*](http://db.yeastgenome.org/cgi-bin/locus.pl?locus=SOD1)*,* [*SOD2*](http://db.yeastgenome.org/cgi-bin/locus.pl?locus=SOD2)*,* [*STF2*](http://db.yeastgenome.org/cgi-bin/locus.pl?locus=STF2)*,* [*TPS1*](http://db.yeastgenome.org/cgi-bin/locus.pl?locus=TPS1)*,* [*TPS2*](http://db.yeastgenome.org/cgi-bin/locus.pl?locus=TPS2)*,* [*TPS3*](http://db.yeastgenome.org/cgi-bin/locus.pl?locus=TPS3)*,* [*TRR1*](http://db.yeastgenome.org/cgi-bin/locus.pl?locus=TRR1)*,* [*TRX2*](http://db.yeastgenome.org/cgi-bin/locus.pl?locus=TRX2)*,* [*TSA2*](http://db.yeastgenome.org/cgi-bin/locus.pl?locus=TSA2)*,* [*TSL1*](http://db.yeastgenome.org/cgi-bin/locus.pl?locus=TSL1)*,* [*UBA4*](http://db.yeastgenome.org/cgi-bin/locus.pl?locus=UBA4)*,* [*UBC5*](http://db.yeastgenome.org/cgi-bin/locus.pl?locus=UBC5)*,* [*UBI4*](http://db.yeastgenome.org/cgi-bin/locus.pl?locus=UBI4)*,* [*YAP1*](http://db.yeastgenome.org/cgi-bin/locus.pl?locus=YAP1)*,* [*YDC1*](http://db.yeastgenome.org/cgi-bin/locus.pl?locus=YDC1)*,* [*YGP1*](http://db.yeastgenome.org/cgi-bin/locus.pl?locus=YGP1)*,* [*YKU70*](http://db.yeastgenome.org/cgi-bin/locus.pl?locus=YKU70)*,* [*ZWF1*](http://db.yeastgenome.org/cgi-bin/locus.pl?locus=ZWF1) |
| Catabolic process | 51 out of 399 genes, 12.8% | 5.95e-5 | [*ALD3*](http://db.yeastgenome.org/cgi-bin/locus.pl?locus=ALD3)*,* [*CDC53*](http://db.yeastgenome.org/cgi-bin/locus.pl?locus=CDC53)*,* [*DAK1*](http://db.yeastgenome.org/cgi-bin/locus.pl?locus=DAK1)*,* [*DCS1*](http://db.yeastgenome.org/cgi-bin/locus.pl?locus=DCS1)*,* [*DCS2*](http://db.yeastgenome.org/cgi-bin/locus.pl?locus=DCS2)*,* [*DDI1*](http://db.yeastgenome.org/cgi-bin/locus.pl?locus=DDI1)*,* [*ECM29*](http://db.yeastgenome.org/cgi-bin/locus.pl?locus=ECM29)*,* [*ECM38*](http://db.yeastgenome.org/cgi-bin/locus.pl?locus=ECM38)*,* [*GAD1*](http://db.yeastgenome.org/cgi-bin/locus.pl?locus=GAD1)*,* [*GCY1*](http://db.yeastgenome.org/cgi-bin/locus.pl?locus=GCY1)*,* [*GDB1*](http://db.yeastgenome.org/cgi-bin/locus.pl?locus=GDB1)*,* [*GGA1*](http://db.yeastgenome.org/cgi-bin/locus.pl?locus=GGA1)*,* [*GLK1*](http://db.yeastgenome.org/cgi-bin/locus.pl?locus=GLK1)*,* [*GLO1*](http://db.yeastgenome.org/cgi-bin/locus.pl?locus=GLO1)*,* [*GLO4*](http://db.yeastgenome.org/cgi-bin/locus.pl?locus=GLO4)*,* [*GND2*](http://db.yeastgenome.org/cgi-bin/locus.pl?locus=GND2)*,* [*GOR1*](http://db.yeastgenome.org/cgi-bin/locus.pl?locus=GOR1)*,* [*GPH1*](http://db.yeastgenome.org/cgi-bin/locus.pl?locus=GPH1)*,* [*GRE3*](http://db.yeastgenome.org/cgi-bin/locus.pl?locus=GRE3)*,* [*HMX1*](http://db.yeastgenome.org/cgi-bin/locus.pl?locus=HMX1)*,* [*LAP3*](http://db.yeastgenome.org/cgi-bin/locus.pl?locus=LAP3)*,* [*LAP4*](http://db.yeastgenome.org/cgi-bin/locus.pl?locus=LAP4)*,* [*NPL4*](http://db.yeastgenome.org/cgi-bin/locus.pl?locus=NPL4)*,* [*NTA1*](http://db.yeastgenome.org/cgi-bin/locus.pl?locus=NTA1)*,* [*NTG1*](http://db.yeastgenome.org/cgi-bin/locus.pl?locus=NTG1)*,* [*NTH1*](http://db.yeastgenome.org/cgi-bin/locus.pl?locus=NTH1)*,* [*PBN1*](http://db.yeastgenome.org/cgi-bin/locus.pl?locus=PBN1)*,* [*PEP4*](http://db.yeastgenome.org/cgi-bin/locus.pl?locus=PEP4)*,* [*PRB1*](http://db.yeastgenome.org/cgi-bin/locus.pl?locus=PRB1)*,* [*PRE8*](http://db.yeastgenome.org/cgi-bin/locus.pl?locus=PRE8)*,* [*RAD16*](http://db.yeastgenome.org/cgi-bin/locus.pl?locus=RAD16)*,* [*RAD50*](http://db.yeastgenome.org/cgi-bin/locus.pl?locus=RAD50)*,* [*RPN12*](http://db.yeastgenome.org/cgi-bin/locus.pl?locus=RPN12)*,* [*RPN5*](http://db.yeastgenome.org/cgi-bin/locus.pl?locus=RPN5)*,* [*RPN8*](http://db.yeastgenome.org/cgi-bin/locus.pl?locus=RPN8)*,* [*RPT2*](http://db.yeastgenome.org/cgi-bin/locus.pl?locus=RPT2)*,* [*SAF1*](http://db.yeastgenome.org/cgi-bin/locus.pl?locus=SAF1)*,* [*SFA1*](http://db.yeastgenome.org/cgi-bin/locus.pl?locus=SFA1)*,* [*SOL4*](http://db.yeastgenome.org/cgi-bin/locus.pl?locus=SOL4)*,* [*SRY1*](http://db.yeastgenome.org/cgi-bin/locus.pl?locus=SRY1)*,* [*SUE1*](http://db.yeastgenome.org/cgi-bin/locus.pl?locus=SUE1)*,* [*TKL2*](http://db.yeastgenome.org/cgi-bin/locus.pl?locus=TKL2)*,* [*UBC8*](http://db.yeastgenome.org/cgi-bin/locus.pl?locus=UBC8)*,* [*UBX4*](http://db.yeastgenome.org/cgi-bin/locus.pl?locus=UBX4)*,* [*UBX5*](http://db.yeastgenome.org/cgi-bin/locus.pl?locus=UBX5)*,* [*UBX6*](http://db.yeastgenome.org/cgi-bin/locus.pl?locus=UBX6)*,* [*UFD1*](http://db.yeastgenome.org/cgi-bin/locus.pl?locus=UFD1)*,* [*YJR096W*](http://db.yeastgenome.org/cgi-bin/locus.pl?locus=YJR096W)*,* [*YLR345W*](http://db.yeastgenome.org/cgi-bin/locus.pl?locus=YLR345W)*,* [*YPR1*](http://db.yeastgenome.org/cgi-bin/locus.pl?locus=YPR1)*,* [*ZWF1*](http://db.yeastgenome.org/cgi-bin/locus.pl?locus=ZWF1) |
| Pentose metabolic process | 7 out of 399 genes, 1.8% | 0.00028 | [*GCY1*](http://db.yeastgenome.org/cgi-bin/locus.pl?locus=GCY1)*,* [*GND2*](http://db.yeastgenome.org/cgi-bin/locus.pl?locus=GND2)*,* [*GRE3*](http://db.yeastgenome.org/cgi-bin/locus.pl?locus=GRE3)*,* [*SOL4*](http://db.yeastgenome.org/cgi-bin/locus.pl?locus=SOL4)*,* [*YJR096W*](http://db.yeastgenome.org/cgi-bin/locus.pl?locus=YJR096W)*,* [*YPR1*](http://db.yeastgenome.org/cgi-bin/locus.pl?locus=YPR1)*,* [*ZWF1*](http://db.yeastgenome.org/cgi-bin/locus.pl?locus=ZWF1) |
| Cellular catabolic process | 48 out of 399 genes, 12.0% | 0.00034 | [*ALD3*](http://db.yeastgenome.org/cgi-bin/locus.pl?locus=ALD3)*,* [*CDC53*](http://db.yeastgenome.org/cgi-bin/locus.pl?locus=CDC53)*,* [*DAK1*](http://db.yeastgenome.org/cgi-bin/locus.pl?locus=DAK1)*,* [*DCS1*](http://db.yeastgenome.org/cgi-bin/locus.pl?locus=DCS1)*,* [*DCS2*](http://db.yeastgenome.org/cgi-bin/locus.pl?locus=DCS2)*,* [*DDI1*](http://db.yeastgenome.org/cgi-bin/locus.pl?locus=DDI1)*,* [*ECM38*](http://db.yeastgenome.org/cgi-bin/locus.pl?locus=ECM38)*,* [*GAD1*](http://db.yeastgenome.org/cgi-bin/locus.pl?locus=GAD1)*,* [*GCY1*](http://db.yeastgenome.org/cgi-bin/locus.pl?locus=GCY1)*,* [*GDB1*](http://db.yeastgenome.org/cgi-bin/locus.pl?locus=GDB1)*,* [*GGA1*](http://db.yeastgenome.org/cgi-bin/locus.pl?locus=GGA1)*,* [*GLK1*](http://db.yeastgenome.org/cgi-bin/locus.pl?locus=GLK1)*,* [*GLO1*](http://db.yeastgenome.org/cgi-bin/locus.pl?locus=GLO1)*,* [*GLO4*](http://db.yeastgenome.org/cgi-bin/locus.pl?locus=GLO4)*,* [*GND2*](http://db.yeastgenome.org/cgi-bin/locus.pl?locus=GND2)*,* [*GOR1*](http://db.yeastgenome.org/cgi-bin/locus.pl?locus=GOR1)*,* [*GPH1*](http://db.yeastgenome.org/cgi-bin/locus.pl?locus=GPH1)*,* [*GRE3*](http://db.yeastgenome.org/cgi-bin/locus.pl?locus=GRE3)*,* [*HMX1*](http://db.yeastgenome.org/cgi-bin/locus.pl?locus=HMX1)*,* [*LAP3*](http://db.yeastgenome.org/cgi-bin/locus.pl?locus=LAP3)*,* [*LAP4*](http://db.yeastgenome.org/cgi-bin/locus.pl?locus=LAP4)*,* [*NPL4*](http://db.yeastgenome.org/cgi-bin/locus.pl?locus=NPL4)*,* [*NTG1*](http://db.yeastgenome.org/cgi-bin/locus.pl?locus=NTG1)*,* [*NTH1*](http://db.yeastgenome.org/cgi-bin/locus.pl?locus=NTH1)*,* [*PBN1*](http://db.yeastgenome.org/cgi-bin/locus.pl?locus=PBN1)*,* [*PEP4*](http://db.yeastgenome.org/cgi-bin/locus.pl?locus=PEP4)*,* [*PRB1*](http://db.yeastgenome.org/cgi-bin/locus.pl?locus=PRB1)*,* [*PRE8*](http://db.yeastgenome.org/cgi-bin/locus.pl?locus=PRE8)*,* [*RAD16*](http://db.yeastgenome.org/cgi-bin/locus.pl?locus=RAD16)*,* [*RAD50*](http://db.yeastgenome.org/cgi-bin/locus.pl?locus=RAD50)*,* [*RPN12*](http://db.yeastgenome.org/cgi-bin/locus.pl?locus=RPN12)*,* [*RPN5*](http://db.yeastgenome.org/cgi-bin/locus.pl?locus=RPN5)*,* [*RPN8*](http://db.yeastgenome.org/cgi-bin/locus.pl?locus=RPN8)*,* [*RPT2*](http://db.yeastgenome.org/cgi-bin/locus.pl?locus=RPT2)*,* [*SAF1*](http://db.yeastgenome.org/cgi-bin/locus.pl?locus=SAF1)*,* [*SFA1*](http://db.yeastgenome.org/cgi-bin/locus.pl?locus=SFA1)*,* [*SOL4*](http://db.yeastgenome.org/cgi-bin/locus.pl?locus=SOL4)*,* [*SRY1*](http://db.yeastgenome.org/cgi-bin/locus.pl?locus=SRY1)*,* [*TKL2*](http://db.yeastgenome.org/cgi-bin/locus.pl?locus=TKL2)*,* [*UBC8*](http://db.yeastgenome.org/cgi-bin/locus.pl?locus=UBC8)*,* [*UBX4*](http://db.yeastgenome.org/cgi-bin/locus.pl?locus=UBX4)*,* [*UBX5*](http://db.yeastgenome.org/cgi-bin/locus.pl?locus=UBX5)*,* [*UBX6*](http://db.yeastgenome.org/cgi-bin/locus.pl?locus=UBX6)*,* [*UFD1*](http://db.yeastgenome.org/cgi-bin/locus.pl?locus=UFD1)*,* [*YJR096W*](http://db.yeastgenome.org/cgi-bin/locus.pl?locus=YJR096W)*,* [*YLR345W*](http://db.yeastgenome.org/cgi-bin/locus.pl?locus=YLR345W)*,* [*YPR1*](http://db.yeastgenome.org/cgi-bin/locus.pl?locus=YPR1)*,* [*ZWF1*](http://db.yeastgenome.org/cgi-bin/locus.pl?locus=ZWF1) |
| Aldehyde metabolic process | 9 out of 399 genes, 2.3% | 0.00049 | [*AAD15*](http://db.yeastgenome.org/cgi-bin/locus.pl?locus=AAD15)*,* [*AAD16*](http://db.yeastgenome.org/cgi-bin/locus.pl?locus=AAD16)*,* [*AAD4*](http://db.yeastgenome.org/cgi-bin/locus.pl?locus=AAD4)*,* [*AAD6*](http://db.yeastgenome.org/cgi-bin/locus.pl?locus=AAD6)*,* [*GLO1*](http://db.yeastgenome.org/cgi-bin/locus.pl?locus=GLO1)*,* [*GLO4*](http://db.yeastgenome.org/cgi-bin/locus.pl?locus=GLO4)*,* [*GOR1*](http://db.yeastgenome.org/cgi-bin/locus.pl?locus=GOR1)*,* [*HFD1*](http://db.yeastgenome.org/cgi-bin/locus.pl?locus=HFD1)*,* [*SFA1*](http://db.yeastgenome.org/cgi-bin/locus.pl?locus=SFA1) |
| Response to reactive oxygen species | 7 out of 399 genes, 1.8% | 0.00066 | [*AIF1*](http://db.yeastgenome.org/cgi-bin/locus.pl?locus=AIF1)*,* [*CTT1*](http://db.yeastgenome.org/cgi-bin/locus.pl?locus=CTT1)*,* [*GSH1*](http://db.yeastgenome.org/cgi-bin/locus.pl?locus=GSH1)*,* [*SOD1*](http://db.yeastgenome.org/cgi-bin/locus.pl?locus=SOD1)*,* [*SOD2*](http://db.yeastgenome.org/cgi-bin/locus.pl?locus=SOD2)*,* [*YAP1*](http://db.yeastgenome.org/cgi-bin/locus.pl?locus=YAP1)*,* [*ZWF1*](http://db.yeastgenome.org/cgi-bin/locus.pl?locus=ZWF1) |
| Carbohydrate metabolic process | 32 out of 399 genes, 8.0% | 0.00083 | [*AMS1*](http://db.yeastgenome.org/cgi-bin/locus.pl?locus=AMS1)*,* [*ARA1*](http://db.yeastgenome.org/cgi-bin/locus.pl?locus=ARA1)*,* [*FBP26*](http://db.yeastgenome.org/cgi-bin/locus.pl?locus=FBP26)*,* [*FYV10*](http://db.yeastgenome.org/cgi-bin/locus.pl?locus=FYV10)*,* [*GCY1*](http://db.yeastgenome.org/cgi-bin/locus.pl?locus=GCY1)*,* [*GDB1*](http://db.yeastgenome.org/cgi-bin/locus.pl?locus=GDB1)*,* [*GLC3*](http://db.yeastgenome.org/cgi-bin/locus.pl?locus=GLC3)*,* [*GLK1*](http://db.yeastgenome.org/cgi-bin/locus.pl?locus=GLK1)*,* [*GLO4*](http://db.yeastgenome.org/cgi-bin/locus.pl?locus=GLO4)*,* [*GND2*](http://db.yeastgenome.org/cgi-bin/locus.pl?locus=GND2)*,* [*GPH1*](http://db.yeastgenome.org/cgi-bin/locus.pl?locus=GPH1)*,* [*GRE3*](http://db.yeastgenome.org/cgi-bin/locus.pl?locus=GRE3)*,* [*GSY2*](http://db.yeastgenome.org/cgi-bin/locus.pl?locus=GSY2)*,* [*NRG1*](http://db.yeastgenome.org/cgi-bin/locus.pl?locus=NRG1)*,* [*NTH1*](http://db.yeastgenome.org/cgi-bin/locus.pl?locus=NTH1)*,* [*OPI10*](http://db.yeastgenome.org/cgi-bin/locus.pl?locus=OPI10)*,* [*PGM2*](http://db.yeastgenome.org/cgi-bin/locus.pl?locus=PGM2)*,* [*PIG2*](http://db.yeastgenome.org/cgi-bin/locus.pl?locus=PIG2)*,* [*PSK1*](http://db.yeastgenome.org/cgi-bin/locus.pl?locus=PSK1)*,* [*SOL4*](http://db.yeastgenome.org/cgi-bin/locus.pl?locus=SOL4)*,* [*TKL2*](http://db.yeastgenome.org/cgi-bin/locus.pl?locus=TKL2)*,* [*TPS1*](http://db.yeastgenome.org/cgi-bin/locus.pl?locus=TPS1)*,* [*TPS2*](http://db.yeastgenome.org/cgi-bin/locus.pl?locus=TPS2)*,* [*TPS3*](http://db.yeastgenome.org/cgi-bin/locus.pl?locus=TPS3)*,* [*TSL1*](http://db.yeastgenome.org/cgi-bin/locus.pl?locus=TSL1)*,* [*UBC8*](http://db.yeastgenome.org/cgi-bin/locus.pl?locus=UBC8)*,* [*UGP1*](http://db.yeastgenome.org/cgi-bin/locus.pl?locus=UGP1)*,* [*YJR096W*](http://db.yeastgenome.org/cgi-bin/locus.pl?locus=YJR096W)*,* [*YLR345W*](http://db.yeastgenome.org/cgi-bin/locus.pl?locus=YLR345W)*,* [*YPI1*](http://db.yeastgenome.org/cgi-bin/locus.pl?locus=YPI1)*,* [*YPR1*](http://db.yeastgenome.org/cgi-bin/locus.pl?locus=YPR1)*,* [*ZWF1*](http://db.yeastgenome.org/cgi-bin/locus.pl?locus=ZWF1) |
| Monosaccharide metabolic process | 18 out of 399 genes, 4.5% | 0.00139 | [*FBP26*](http://db.yeastgenome.org/cgi-bin/locus.pl?locus=FBP26)*,* [*FYV10*](http://db.yeastgenome.org/cgi-bin/locus.pl?locus=FYV10)*,* [*GCY1*](http://db.yeastgenome.org/cgi-bin/locus.pl?locus=GCY1)*,* [*GLK1*](http://db.yeastgenome.org/cgi-bin/locus.pl?locus=GLK1)*,* [*GND2*](http://db.yeastgenome.org/cgi-bin/locus.pl?locus=GND2)*,* [*GRE3*](http://db.yeastgenome.org/cgi-bin/locus.pl?locus=GRE3)*,* [*NRG1*](http://db.yeastgenome.org/cgi-bin/locus.pl?locus=NRG1)*,* [*OPI10*](http://db.yeastgenome.org/cgi-bin/locus.pl?locus=OPI10)*,* [*PGM2*](http://db.yeastgenome.org/cgi-bin/locus.pl?locus=PGM2)*,* [*PSK1*](http://db.yeastgenome.org/cgi-bin/locus.pl?locus=PSK1)*,* [*SOL4*](http://db.yeastgenome.org/cgi-bin/locus.pl?locus=SOL4)*,* [*TKL2*](http://db.yeastgenome.org/cgi-bin/locus.pl?locus=TKL2)*,* [*UBC8*](http://db.yeastgenome.org/cgi-bin/locus.pl?locus=UBC8)*,* [*UGP1*](http://db.yeastgenome.org/cgi-bin/locus.pl?locus=UGP1)*,* [*YJR096W*](http://db.yeastgenome.org/cgi-bin/locus.pl?locus=YJR096W)*,* [*YLR345W*](http://db.yeastgenome.org/cgi-bin/locus.pl?locus=YLR345W)*,* [*YPR1*](http://db.yeastgenome.org/cgi-bin/locus.pl?locus=YPR1)*,* [*ZWF1*](http://db.yeastgenome.org/cgi-bin/locus.pl?locus=ZWF1) |
| Energy reserve metabolic process | 11 out of 399 genes, 2.8% | 0.00151 | [*GDB1*](http://db.yeastgenome.org/cgi-bin/locus.pl?locus=GDB1)*,* [*GLC3*](http://db.yeastgenome.org/cgi-bin/locus.pl?locus=GLC3)*,* [*GPH1*](http://db.yeastgenome.org/cgi-bin/locus.pl?locus=GPH1)*,* [*GSY2*](http://db.yeastgenome.org/cgi-bin/locus.pl?locus=GSY2)*,* [*NTH1*](http://db.yeastgenome.org/cgi-bin/locus.pl?locus=NTH1)*,* [*PIG2*](http://db.yeastgenome.org/cgi-bin/locus.pl?locus=PIG2)*,* [*TPS1*](http://db.yeastgenome.org/cgi-bin/locus.pl?locus=TPS1)*,* [*TPS2*](http://db.yeastgenome.org/cgi-bin/locus.pl?locus=TPS2)*,* [*TPS3*](http://db.yeastgenome.org/cgi-bin/locus.pl?locus=TPS3)*,* [*TSL1*](http://db.yeastgenome.org/cgi-bin/locus.pl?locus=TSL1)*,* [*YPI1*](http://db.yeastgenome.org/cgi-bin/locus.pl?locus=YPI1) |
| Sulfur metabolic process | 15 out of 399 genes, 3.8% | 0.00173 | [*ECM38*](http://db.yeastgenome.org/cgi-bin/locus.pl?locus=ECM38)*,* [*ECM4*](http://db.yeastgenome.org/cgi-bin/locus.pl?locus=ECM4)*,* [*GLO1*](http://db.yeastgenome.org/cgi-bin/locus.pl?locus=GLO1)*,* [*GSH1*](http://db.yeastgenome.org/cgi-bin/locus.pl?locus=GSH1)*,* [*GTO3*](http://db.yeastgenome.org/cgi-bin/locus.pl?locus=GTO3)*,* [*GTT1*](http://db.yeastgenome.org/cgi-bin/locus.pl?locus=GTT1)*,* [*GTT2*](http://db.yeastgenome.org/cgi-bin/locus.pl?locus=GTT2)*,* [*ISA2*](http://db.yeastgenome.org/cgi-bin/locus.pl?locus=ISA2)*,* [*JLP1*](http://db.yeastgenome.org/cgi-bin/locus.pl?locus=JLP1)*,* [*LAP3*](http://db.yeastgenome.org/cgi-bin/locus.pl?locus=LAP3)*,* [*MET16*](http://db.yeastgenome.org/cgi-bin/locus.pl?locus=MET16)*,* [*STR2*](http://db.yeastgenome.org/cgi-bin/locus.pl?locus=STR2)*,* [*STR3*](http://db.yeastgenome.org/cgi-bin/locus.pl?locus=STR3)*,* [*TRX2*](http://db.yeastgenome.org/cgi-bin/locus.pl?locus=TRX2)*,* [*YLL058W*](http://db.yeastgenome.org/cgi-bin/locus.pl?locus=YLL058W) |
| Glutathione metabolic process | 7 out of 399 genes, 1.8% | 0.00259 | [*ECM38*](http://db.yeastgenome.org/cgi-bin/locus.pl?locus=ECM38)*,* [*ECM4*](http://db.yeastgenome.org/cgi-bin/locus.pl?locus=ECM4)*,* [*GLO1*](http://db.yeastgenome.org/cgi-bin/locus.pl?locus=GLO1)*,* [*GSH1*](http://db.yeastgenome.org/cgi-bin/locus.pl?locus=GSH1)*,* [*GTO3*](http://db.yeastgenome.org/cgi-bin/locus.pl?locus=GTO3)*,* [*GTT1*](http://db.yeastgenome.org/cgi-bin/locus.pl?locus=GTT1)*,* [*GTT2*](http://db.yeastgenome.org/cgi-bin/locus.pl?locus=GTT2) |
| Cellular carbohydrate metabolic process | 29 out of 399 genes, 7.3% | 0.00323 | [*FBP26*](http://db.yeastgenome.org/cgi-bin/locus.pl?locus=FBP26)*,* [*FYV10*](http://db.yeastgenome.org/cgi-bin/locus.pl?locus=FYV10)*,* [*GCY1*](http://db.yeastgenome.org/cgi-bin/locus.pl?locus=GCY1)*,* [*GDB1*](http://db.yeastgenome.org/cgi-bin/locus.pl?locus=GDB1)*,* [*GLC3*](http://db.yeastgenome.org/cgi-bin/locus.pl?locus=GLC3)*,* [*GLK1*](http://db.yeastgenome.org/cgi-bin/locus.pl?locus=GLK1)*,* [*GND2*](http://db.yeastgenome.org/cgi-bin/locus.pl?locus=GND2)*,* [*GPH1*](http://db.yeastgenome.org/cgi-bin/locus.pl?locus=GPH1)*,* [*GRE3*](http://db.yeastgenome.org/cgi-bin/locus.pl?locus=GRE3)*,* [*GSY2*](http://db.yeastgenome.org/cgi-bin/locus.pl?locus=GSY2)*,* [*NRG1*](http://db.yeastgenome.org/cgi-bin/locus.pl?locus=NRG1)*,* [*NTH1*](http://db.yeastgenome.org/cgi-bin/locus.pl?locus=NTH1)*,* [*OPI10*](http://db.yeastgenome.org/cgi-bin/locus.pl?locus=OPI10)*,* [*PGM2*](http://db.yeastgenome.org/cgi-bin/locus.pl?locus=PGM2)*,* [*PIG2*](http://db.yeastgenome.org/cgi-bin/locus.pl?locus=PIG2)*,* [*PSK1*](http://db.yeastgenome.org/cgi-bin/locus.pl?locus=PSK1)*,* [*SOL4*](http://db.yeastgenome.org/cgi-bin/locus.pl?locus=SOL4)*,* [*TKL2*](http://db.yeastgenome.org/cgi-bin/locus.pl?locus=TKL2)*,* [*TPS1*](http://db.yeastgenome.org/cgi-bin/locus.pl?locus=TPS1)*,* [*TPS2*](http://db.yeastgenome.org/cgi-bin/locus.pl?locus=TPS2)*,* [*TPS3*](http://db.yeastgenome.org/cgi-bin/locus.pl?locus=TPS3)*,* [*TSL1*](http://db.yeastgenome.org/cgi-bin/locus.pl?locus=TSL1)*,* [*UBC8*](http://db.yeastgenome.org/cgi-bin/locus.pl?locus=UBC8)*,* [*UGP1*](http://db.yeastgenome.org/cgi-bin/locus.pl?locus=UGP1)*,* [*YJR096W*](http://db.yeastgenome.org/cgi-bin/locus.pl?locus=YJR096W)*,* [*YLR345W*](http://db.yeastgenome.org/cgi-bin/locus.pl?locus=YLR345W)*,* [*YPI1*](http://db.yeastgenome.org/cgi-bin/locus.pl?locus=YPI1)*,* [*YPR1*](http://db.yeastgenome.org/cgi-bin/locus.pl?locus=YPR1)*,* [*ZWF1*](http://db.yeastgenome.org/cgi-bin/locus.pl?locus=ZWF1) |
| Cofactor metabolic process | 25 out of 399 genes, 6.3% | 0.00340 | [*ABZ1*](http://db.yeastgenome.org/cgi-bin/locus.pl?locus=ABZ1)*,* [*CFD1*](http://db.yeastgenome.org/cgi-bin/locus.pl?locus=CFD1)*,* [*COQ1*](http://db.yeastgenome.org/cgi-bin/locus.pl?locus=COQ1)*,* [*COQ4*](http://db.yeastgenome.org/cgi-bin/locus.pl?locus=COQ4)*,* [*ECM38*](http://db.yeastgenome.org/cgi-bin/locus.pl?locus=ECM38)*,* [*ECM4*](http://db.yeastgenome.org/cgi-bin/locus.pl?locus=ECM4)*,* [*FAD1*](http://db.yeastgenome.org/cgi-bin/locus.pl?locus=FAD1)*,* [*GLO1*](http://db.yeastgenome.org/cgi-bin/locus.pl?locus=GLO1)*,* [*GND2*](http://db.yeastgenome.org/cgi-bin/locus.pl?locus=GND2)*,* [*GPD1*](http://db.yeastgenome.org/cgi-bin/locus.pl?locus=GPD1)*,* [*GSH1*](http://db.yeastgenome.org/cgi-bin/locus.pl?locus=GSH1)*,* [*GTO3*](http://db.yeastgenome.org/cgi-bin/locus.pl?locus=GTO3)*,* [*GTT1*](http://db.yeastgenome.org/cgi-bin/locus.pl?locus=GTT1)*,* [*GTT2*](http://db.yeastgenome.org/cgi-bin/locus.pl?locus=GTT2)*,* [*HMX1*](http://db.yeastgenome.org/cgi-bin/locus.pl?locus=HMX1)*,* [*ISA2*](http://db.yeastgenome.org/cgi-bin/locus.pl?locus=ISA2)*,* [*ISU1*](http://db.yeastgenome.org/cgi-bin/locus.pl?locus=ISU1)*,* [*ISU2*](http://db.yeastgenome.org/cgi-bin/locus.pl?locus=ISU2)*,* [*NBP35*](http://db.yeastgenome.org/cgi-bin/locus.pl?locus=NBP35)*,* [*PNC1*](http://db.yeastgenome.org/cgi-bin/locus.pl?locus=PNC1)*,* [*SOL4*](http://db.yeastgenome.org/cgi-bin/locus.pl?locus=SOL4)*,* [*STF2*](http://db.yeastgenome.org/cgi-bin/locus.pl?locus=STF2)*,* [*TKL2*](http://db.yeastgenome.org/cgi-bin/locus.pl?locus=TKL2)*,* [*YEF1*](http://db.yeastgenome.org/cgi-bin/locus.pl?locus=YEF1)*,* [*ZWF1*](http://db.yeastgenome.org/cgi-bin/locus.pl?locus=ZWF1) |
| Macromolecule metabolic process | 39 out of 399 genes, 9.8% | 0.00441 | [*CDC53*](http://db.yeastgenome.org/cgi-bin/locus.pl?locus=CDC53)*,* [*DCS1*](http://db.yeastgenome.org/cgi-bin/locus.pl?locus=DCS1)*,* [*DCS2*](http://db.yeastgenome.org/cgi-bin/locus.pl?locus=DCS2)*,* [*DDI1*](http://db.yeastgenome.org/cgi-bin/locus.pl?locus=DDI1)*,* [*ECM29*](http://db.yeastgenome.org/cgi-bin/locus.pl?locus=ECM29)*,* [*GCY1*](http://db.yeastgenome.org/cgi-bin/locus.pl?locus=GCY1)*,* [*GDB1*](http://db.yeastgenome.org/cgi-bin/locus.pl?locus=GDB1)*,* [*GGA1*](http://db.yeastgenome.org/cgi-bin/locus.pl?locus=GGA1)*,* [*GLK1*](http://db.yeastgenome.org/cgi-bin/locus.pl?locus=GLK1)*,* [*GND2*](http://db.yeastgenome.org/cgi-bin/locus.pl?locus=GND2)*,* [*GPH1*](http://db.yeastgenome.org/cgi-bin/locus.pl?locus=GPH1)*,* [*GRE3*](http://db.yeastgenome.org/cgi-bin/locus.pl?locus=GRE3)*,* [*LAP4*](http://db.yeastgenome.org/cgi-bin/locus.pl?locus=LAP4)*,* [*NPL4*](http://db.yeastgenome.org/cgi-bin/locus.pl?locus=NPL4)*,* [*NTA1*](http://db.yeastgenome.org/cgi-bin/locus.pl?locus=NTA1)*,* [*NTH1*](http://db.yeastgenome.org/cgi-bin/locus.pl?locus=NTH1)*,* [*PBN1*](http://db.yeastgenome.org/cgi-bin/locus.pl?locus=PBN1)*,* [*PEP4*](http://db.yeastgenome.org/cgi-bin/locus.pl?locus=PEP4)*,* [*PRB1*](http://db.yeastgenome.org/cgi-bin/locus.pl?locus=PRB1)*,* [*PRE8*](http://db.yeastgenome.org/cgi-bin/locus.pl?locus=PRE8)*,* [*RAD16*](http://db.yeastgenome.org/cgi-bin/locus.pl?locus=RAD16)*,* [*RAD50*](http://db.yeastgenome.org/cgi-bin/locus.pl?locus=RAD50)*,* [*RPN12*](http://db.yeastgenome.org/cgi-bin/locus.pl?locus=RPN12)*,* [*RPN5*](http://db.yeastgenome.org/cgi-bin/locus.pl?locus=RPN5)*,* [*RPN8*](http://db.yeastgenome.org/cgi-bin/locus.pl?locus=RPN8)*,* [*RPT2*](http://db.yeastgenome.org/cgi-bin/locus.pl?locus=RPT2)*,* [*SAF1*](http://db.yeastgenome.org/cgi-bin/locus.pl?locus=SAF1)*,* [*SOL4*](http://db.yeastgenome.org/cgi-bin/locus.pl?locus=SOL4)*,* [*SUE1*](http://db.yeastgenome.org/cgi-bin/locus.pl?locus=SUE1)*,* [*TKL2*](http://db.yeastgenome.org/cgi-bin/locus.pl?locus=TKL2)*,* [*UBC8*](http://db.yeastgenome.org/cgi-bin/locus.pl?locus=UBC8)*,* [*UBX4*](http://db.yeastgenome.org/cgi-bin/locus.pl?locus=UBX4)*,* [*UBX5*](http://db.yeastgenome.org/cgi-bin/locus.pl?locus=UBX5)*,* [*UBX6*](http://db.yeastgenome.org/cgi-bin/locus.pl?locus=UBX6)*,* [*UFD1*](http://db.yeastgenome.org/cgi-bin/locus.pl?locus=UFD1)*,* [*YJR096W*](http://db.yeastgenome.org/cgi-bin/locus.pl?locus=YJR096W)*,* [*YLR345W*](http://db.yeastgenome.org/cgi-bin/locus.pl?locus=YLR345W)*,* [*YPR1*](http://db.yeastgenome.org/cgi-bin/locus.pl?locus=YPR1)*,* [*ZWF1*](http://db.yeastgenome.org/cgi-bin/locus.pl?locus=ZWF1) |
| Arabinose metabolic process | 4 out of 399 genes, 1.0% | 0.00670 | [*GCY1*](http://db.yeastgenome.org/cgi-bin/locus.pl?locus=GCY1)*,* [*GRE3*](http://db.yeastgenome.org/cgi-bin/locus.pl?locus=GRE3)*,* [*YJR096W*](http://db.yeastgenome.org/cgi-bin/locus.pl?locus=YJR096W)*,* [*YPR1*](http://db.yeastgenome.org/cgi-bin/locus.pl?locus=YPR1) |
| Arabinose catabolic process | 4 out of 399 genes, 1.0% | 0.00670 | [*GCY1*](http://db.yeastgenome.org/cgi-bin/locus.pl?locus=GCY1)*,* [*GRE3*](http://db.yeastgenome.org/cgi-bin/locus.pl?locus=GRE3)*,* [*YJR096W*](http://db.yeastgenome.org/cgi-bin/locus.pl?locus=YJR096W)*,* [*YPR1*](http://db.yeastgenome.org/cgi-bin/locus.pl?locus=YPR1) |
| D-Xylose metabolic process | 4 out of 399 genes, 1.0% | 0.00670 | [*GCY1*](http://db.yeastgenome.org/cgi-bin/locus.pl?locus=GCY1)*,* [*GRE3*](http://db.yeastgenome.org/cgi-bin/locus.pl?locus=GRE3)*,* [*YJR096W*](http://db.yeastgenome.org/cgi-bin/locus.pl?locus=YJR096W)*,* [*YPR1*](http://db.yeastgenome.org/cgi-bin/locus.pl?locus=YPR1) |
| D-Xylose catabolic process | 4 out of 399 genes, 1.0% | 0.00670 | [*GCY1*](http://db.yeastgenome.org/cgi-bin/locus.pl?locus=GCY1)*,* [*GRE3*](http://db.yeastgenome.org/cgi-bin/locus.pl?locus=GRE3)*,* [*YJR096W*](http://db.yeastgenome.org/cgi-bin/locus.pl?locus=YJR096W)*,* [*YPR1*](http://db.yeastgenome.org/cgi-bin/locus.pl?locus=YPR1) |
| Disaccharide biosynthetic process | 4 out of 399 genes, 1.0% | 0.00670 | [*TPS1*](http://db.yeastgenome.org/cgi-bin/locus.pl?locus=TPS1)*,* [*TPS2*](http://db.yeastgenome.org/cgi-bin/locus.pl?locus=TPS2)*,* [*TPS3*](http://db.yeastgenome.org/cgi-bin/locus.pl?locus=TPS3)*,* [*TSL1*](http://db.yeastgenome.org/cgi-bin/locus.pl?locus=TSL1) |
| Trehalose biosynthetic process | 4 out of 399 genes, 1.0% | 0.00670 | [*TPS1*](http://db.yeastgenome.org/cgi-bin/locus.pl?locus=TPS1)*,* [*TPS2*](http://db.yeastgenome.org/cgi-bin/locus.pl?locus=TPS2)*,* [*TPS3*](http://db.yeastgenome.org/cgi-bin/locus.pl?locus=TPS3)*,* [*TSL1*](http://db.yeastgenome.org/cgi-bin/locus.pl?locus=TSL1) |
| Trehalose metabolic process | 5 out of 399 genes, 1.3% | 0.00695 | [*NTH1*](http://db.yeastgenome.org/cgi-bin/locus.pl?locus=NTH1)*,* [*TPS1*](http://db.yeastgenome.org/cgi-bin/locus.pl?locus=TPS1)*,* [*TPS2*](http://db.yeastgenome.org/cgi-bin/locus.pl?locus=TPS2)*,* [*TPS3*](http://db.yeastgenome.org/cgi-bin/locus.pl?locus=TPS3)*,* [*TSL1*](http://db.yeastgenome.org/cgi-bin/locus.pl?locus=TSL1) |

**Table 3S-D. Genes down-regulated in CHP and H_2_O_2_**-induced stress.

| **Gene Ontology term (AMIGO)** | **Cluster**  **frequency** | **p-value** | **Genes annotated to the term** |
| --- | --- | --- | --- |
| Ribosome biogenesis and assembly | 219 out of 558 genes, 39.2% | 3.17e-173 | [*AAH1*](http://db.yeastgenome.org/cgi-bin/locus.pl?locus=AAH1)*,* [*ALB1*](http://db.yeastgenome.org/cgi-bin/locus.pl?locus=ALB1)*,* [*ARB1*](http://db.yeastgenome.org/cgi-bin/locus.pl?locus=ARB1)*,* [*ARX1*](http://db.yeastgenome.org/cgi-bin/locus.pl?locus=ARX1)*,* [*ATC1*](http://db.yeastgenome.org/cgi-bin/locus.pl?locus=ATC1)*,* [*BCP1*](http://db.yeastgenome.org/cgi-bin/locus.pl?locus=BCP1)*,* [*BMS1*](http://db.yeastgenome.org/cgi-bin/locus.pl?locus=BMS1)*,* [*BRX1*](http://db.yeastgenome.org/cgi-bin/locus.pl?locus=BRX1)*,* [*BUD21*](http://db.yeastgenome.org/cgi-bin/locus.pl?locus=BUD21)*,* [*BUD22*](http://db.yeastgenome.org/cgi-bin/locus.pl?locus=BUD22)*,* [*CBF5*](http://db.yeastgenome.org/cgi-bin/locus.pl?locus=CBF5)*,* [*CGR1*](http://db.yeastgenome.org/cgi-bin/locus.pl?locus=CGR1)*,* [*CIC1*](http://db.yeastgenome.org/cgi-bin/locus.pl?locus=CIC1)*, CMS1,* [*DBP10*](http://db.yeastgenome.org/cgi-bin/locus.pl?locus=DBP10)*,* [*DBP2*](http://db.yeastgenome.org/cgi-bin/locus.pl?locus=DBP2)*,* [*DBP3*](http://db.yeastgenome.org/cgi-bin/locus.pl?locus=DBP3)*,* [*DBP6*](http://db.yeastgenome.org/cgi-bin/locus.pl?locus=DBP6)*,* [*DBP7*](http://db.yeastgenome.org/cgi-bin/locus.pl?locus=DBP7)*,* [*DBP8*](http://db.yeastgenome.org/cgi-bin/locus.pl?locus=DBP8)*,* [*DBP9*](http://db.yeastgenome.org/cgi-bin/locus.pl?locus=DBP9)*,* [*DHR2*](http://db.yeastgenome.org/cgi-bin/locus.pl?locus=DHR2)*,* [*DIM1*](http://db.yeastgenome.org/cgi-bin/locus.pl?locus=DIM1)*,* [*DIP2*](http://db.yeastgenome.org/cgi-bin/locus.pl?locus=DIP2)*,* [*DPH2*](http://db.yeastgenome.org/cgi-bin/locus.pl?locus=DPH2)*,* [*DRS1*](http://db.yeastgenome.org/cgi-bin/locus.pl?locus=DRS1)*,* [*DUS3*](http://db.yeastgenome.org/cgi-bin/locus.pl?locus=DUS3)*,* [*EBP2*](http://db.yeastgenome.org/cgi-bin/locus.pl?locus=EBP2)*,* [*ECM16*](http://db.yeastgenome.org/cgi-bin/locus.pl?locus=ECM16)*, EFG1,* [*EMG1*](http://db.yeastgenome.org/cgi-bin/locus.pl?locus=EMG1)*,* [*ENP1*](http://db.yeastgenome.org/cgi-bin/locus.pl?locus=ENP1)*,* [*ENP2*](http://db.yeastgenome.org/cgi-bin/locus.pl?locus=ENP2)*,* [*ERB1*](http://db.yeastgenome.org/cgi-bin/locus.pl?locus=ERB1)*,* [*ESF1*](http://db.yeastgenome.org/cgi-bin/locus.pl?locus=ESF1)*,* [*ESF2*](http://db.yeastgenome.org/cgi-bin/locus.pl?locus=ESF2)*,* [*FAF1*](http://db.yeastgenome.org/cgi-bin/locus.pl?locus=FAF1)*,* [*FAL1*](http://db.yeastgenome.org/cgi-bin/locus.pl?locus=FAL1)*,* [*FAP7*](http://db.yeastgenome.org/cgi-bin/locus.pl?locus=FAP7)*,* [*FCF2*](http://db.yeastgenome.org/cgi-bin/locus.pl?locus=FCF2)*,* [*FPR4*](http://db.yeastgenome.org/cgi-bin/locus.pl?locus=FPR4)*,* [*FYV7*](http://db.yeastgenome.org/cgi-bin/locus.pl?locus=FYV7)*,* [*GAR1*](http://db.yeastgenome.org/cgi-bin/locus.pl?locus=GAR1)*,* [*GCD10*](http://db.yeastgenome.org/cgi-bin/locus.pl?locus=GCD10)*,* [*GCD14*](http://db.yeastgenome.org/cgi-bin/locus.pl?locus=GCD14)*,* [*GDT1*](http://db.yeastgenome.org/cgi-bin/locus.pl?locus=GDT1)*,* [*GRC3*](http://db.yeastgenome.org/cgi-bin/locus.pl?locus=GRC3)*,* [*HAS1*](http://db.yeastgenome.org/cgi-bin/locus.pl?locus=HAS1)*,* [*HCA4*](http://db.yeastgenome.org/cgi-bin/locus.pl?locus=HCA4)*,* [*HGH1*](http://db.yeastgenome.org/cgi-bin/locus.pl?locus=HGH1)*,* [*HMT1*](http://db.yeastgenome.org/cgi-bin/locus.pl?locus=HMT1)*,* [*IMP3*](http://db.yeastgenome.org/cgi-bin/locus.pl?locus=IMP3)*,* [*IPI1*](http://db.yeastgenome.org/cgi-bin/locus.pl?locus=IPI1)*,* [*IPI3*](http://db.yeastgenome.org/cgi-bin/locus.pl?locus=IPI3)*,* [*KRE33*](http://db.yeastgenome.org/cgi-bin/locus.pl?locus=KRE33)*,* [*KRI1*](http://db.yeastgenome.org/cgi-bin/locus.pl?locus=KRI1)*,* [*KRR1*](http://db.yeastgenome.org/cgi-bin/locus.pl?locus=KRR1)*,* [*LCP5*](http://db.yeastgenome.org/cgi-bin/locus.pl?locus=LCP5)*,* [*LIA1*](http://db.yeastgenome.org/cgi-bin/locus.pl?locus=LIA1)*,* [*LOC1*](http://db.yeastgenome.org/cgi-bin/locus.pl?locus=LOC1)*,* [*LSG1*](http://db.yeastgenome.org/cgi-bin/locus.pl?locus=LSG1)*,* [*MAK11*](http://db.yeastgenome.org/cgi-bin/locus.pl?locus=MAK11)*,* [*MAK16*](http://db.yeastgenome.org/cgi-bin/locus.pl?locus=MAK16)*,* [*MAK21*](http://db.yeastgenome.org/cgi-bin/locus.pl?locus=MAK21)*,* [*MIS1*](http://db.yeastgenome.org/cgi-bin/locus.pl?locus=MIS1)*,* [*MOT1*](http://db.yeastgenome.org/cgi-bin/locus.pl?locus=MOT1)*,* [*MPP10*](http://db.yeastgenome.org/cgi-bin/locus.pl?locus=MPP10)*,* [*MRD1*](http://db.yeastgenome.org/cgi-bin/locus.pl?locus=MRD1)*,* [*MRT4*](http://db.yeastgenome.org/cgi-bin/locus.pl?locus=MRT4)*,* [*MTR3*](http://db.yeastgenome.org/cgi-bin/locus.pl?locus=MTR3)*,* [*NAN1*](http://db.yeastgenome.org/cgi-bin/locus.pl?locus=NAN1)*,* [*NCL1*](http://db.yeastgenome.org/cgi-bin/locus.pl?locus=NCL1)*,* [*NCS2*](http://db.yeastgenome.org/cgi-bin/locus.pl?locus=NCS2)*,* [*NEW1*](http://db.yeastgenome.org/cgi-bin/locus.pl?locus=NEW1)*,* [*NHP2*](http://db.yeastgenome.org/cgi-bin/locus.pl?locus=NHP2)*,* [*NIP7*](http://db.yeastgenome.org/cgi-bin/locus.pl?locus=NIP7)*,* [*NMD3*](http://db.yeastgenome.org/cgi-bin/locus.pl?locus=NMD3)*,* [*NOB1*](http://db.yeastgenome.org/cgi-bin/locus.pl?locus=NOB1)*,* [*NOC2*](http://db.yeastgenome.org/cgi-bin/locus.pl?locus=NOC2)*,* [*NOC3*](http://db.yeastgenome.org/cgi-bin/locus.pl?locus=NOC3)*,* [*NOC4*](http://db.yeastgenome.org/cgi-bin/locus.pl?locus=NOC4)*,* [*NOG1*](http://db.yeastgenome.org/cgi-bin/locus.pl?locus=NOG1)*,* [*NOG2*](http://db.yeastgenome.org/cgi-bin/locus.pl?locus=NOG2)*,* [*NOP13*](http://db.yeastgenome.org/cgi-bin/locus.pl?locus=NOP13)*,* [*NOP14*](http://db.yeastgenome.org/cgi-bin/locus.pl?locus=NOP14)*,* [*NOP15*](http://db.yeastgenome.org/cgi-bin/locus.pl?locus=NOP15)*,* [*NOP16*](http://db.yeastgenome.org/cgi-bin/locus.pl?locus=NOP16)*,* [*NOP2*](http://db.yeastgenome.org/cgi-bin/locus.pl?locus=NOP2)*,* [*NOP4*](http://db.yeastgenome.org/cgi-bin/locus.pl?locus=NOP4)*,* [*NOP53*](http://db.yeastgenome.org/cgi-bin/locus.pl?locus=NOP53)*,* [*NOP58*](http://db.yeastgenome.org/cgi-bin/locus.pl?locus=NOP58)*,* [*NOP6*](http://db.yeastgenome.org/cgi-bin/locus.pl?locus=NOP6)*,* [*NOP7*](http://db.yeastgenome.org/cgi-bin/locus.pl?locus=NOP7)*,* [*NOP8*](http://db.yeastgenome.org/cgi-bin/locus.pl?locus=NOP8)*,* [*NOP9*](http://db.yeastgenome.org/cgi-bin/locus.pl?locus=NOP9)*,* [*NRP1*](http://db.yeastgenome.org/cgi-bin/locus.pl?locus=NRP1)*,* [*NSA1*](http://db.yeastgenome.org/cgi-bin/locus.pl?locus=NSA1)*,* [*NSR1*](http://db.yeastgenome.org/cgi-bin/locus.pl?locus=NSR1)*,* [*NUC1*](http://db.yeastgenome.org/cgi-bin/locus.pl?locus=NUC1)*,* [*NUG1*](http://db.yeastgenome.org/cgi-bin/locus.pl?locus=NUG1)*,* [*PNO1*](http://db.yeastgenome.org/cgi-bin/locus.pl?locus=PNO1)*,* [*POP3*](http://db.yeastgenome.org/cgi-bin/locus.pl?locus=POP3)*,* [*POP6*](http://db.yeastgenome.org/cgi-bin/locus.pl?locus=POP6)*,* [*POP8*](http://db.yeastgenome.org/cgi-bin/locus.pl?locus=POP8)*,* [*PPT1*](http://db.yeastgenome.org/cgi-bin/locus.pl?locus=PPT1)*,* [*PRO1*](http://db.yeastgenome.org/cgi-bin/locus.pl?locus=PRO1)*,* [*PRP43*](http://db.yeastgenome.org/cgi-bin/locus.pl?locus=PRP43)*,* [*PRS4*](http://db.yeastgenome.org/cgi-bin/locus.pl?locus=PRS4)*,* [*PUF6*](http://db.yeastgenome.org/cgi-bin/locus.pl?locus=PUF6)*,* [*PUS1*](http://db.yeastgenome.org/cgi-bin/locus.pl?locus=PUS1)*,* [*PUS7*](http://db.yeastgenome.org/cgi-bin/locus.pl?locus=PUS7)*,* [*PWP1*](http://db.yeastgenome.org/cgi-bin/locus.pl?locus=PWP1)*,* [*PWP2*](http://db.yeastgenome.org/cgi-bin/locus.pl?locus=PWP2)*,* [*PXR1*](http://db.yeastgenome.org/cgi-bin/locus.pl?locus=PXR1)*,* [*RBG1*](http://db.yeastgenome.org/cgi-bin/locus.pl?locus=RBG1)*,* [*RCL1*](http://db.yeastgenome.org/cgi-bin/locus.pl?locus=RCL1)*,* [*REI1*](http://db.yeastgenome.org/cgi-bin/locus.pl?locus=REI1)*,* [*REX4*](http://db.yeastgenome.org/cgi-bin/locus.pl?locus=REX4)*,* [*RIO1*](http://db.yeastgenome.org/cgi-bin/locus.pl?locus=RIO1)*,* [*RIX1*](http://db.yeastgenome.org/cgi-bin/locus.pl?locus=RIX1)*,* [*RIX7*](http://db.yeastgenome.org/cgi-bin/locus.pl?locus=RIX7)*,* [*RKI1*](http://db.yeastgenome.org/cgi-bin/locus.pl?locus=RKI1)*,* [*RLI1*](http://db.yeastgenome.org/cgi-bin/locus.pl?locus=RLI1)*,* [*RLP24*](http://db.yeastgenome.org/cgi-bin/locus.pl?locus=RLP24)*,* [*RLP7*](http://db.yeastgenome.org/cgi-bin/locus.pl?locus=RLP7)*,* [*RMT2*](http://db.yeastgenome.org/cgi-bin/locus.pl?locus=RMT2)*,* [*RNT1*](http://db.yeastgenome.org/cgi-bin/locus.pl?locus=RNT1)*,* [*ROK1*](http://db.yeastgenome.org/cgi-bin/locus.pl?locus=ROK1)*,* [*RPA12*](http://db.yeastgenome.org/cgi-bin/locus.pl?locus=RPA12)*,* [*RPA34*](http://db.yeastgenome.org/cgi-bin/locus.pl?locus=RPA34)*,* [*RPA43*](http://db.yeastgenome.org/cgi-bin/locus.pl?locus=RPA43)*,* [*RPA49*](http://db.yeastgenome.org/cgi-bin/locus.pl?locus=RPA49)*,* [*RPC19*](http://db.yeastgenome.org/cgi-bin/locus.pl?locus=RPC19)*,* [*RPC34*](http://db.yeastgenome.org/cgi-bin/locus.pl?locus=RPC34)*,* [*RPC40*](http://db.yeastgenome.org/cgi-bin/locus.pl?locus=RPC40)*,* [*RPC82*](http://db.yeastgenome.org/cgi-bin/locus.pl?locus=RPC82)*,* [*RPF1*](http://db.yeastgenome.org/cgi-bin/locus.pl?locus=RPF1)*,* [*RPF2*](http://db.yeastgenome.org/cgi-bin/locus.pl?locus=RPF2)*,* [*RPP1*](http://db.yeastgenome.org/cgi-bin/locus.pl?locus=RPP1)*,* [*RPS14B*](http://db.yeastgenome.org/cgi-bin/locus.pl?locus=RPS14B)*,* [*RPS19B*](http://db.yeastgenome.org/cgi-bin/locus.pl?locus=RPS19B)*,* [*RRB1*](http://db.yeastgenome.org/cgi-bin/locus.pl?locus=RRB1)*,* [*RRP1*](http://db.yeastgenome.org/cgi-bin/locus.pl?locus=RRP1)*,* [*RRP12*](http://db.yeastgenome.org/cgi-bin/locus.pl?locus=RRP12)*,* [*RRP14*](http://db.yeastgenome.org/cgi-bin/locus.pl?locus=RRP14)*,* [*RRP15*](http://db.yeastgenome.org/cgi-bin/locus.pl?locus=RRP15)*,* [*RRP17*](http://db.yeastgenome.org/cgi-bin/locus.pl?locus=RRP17)*,* [*RRP3*](http://db.yeastgenome.org/cgi-bin/locus.pl?locus=RRP3)*,RRP36,* [*RRP40*](http://db.yeastgenome.org/cgi-bin/locus.pl?locus=RRP40)*,* [*RRP43*](http://db.yeastgenome.org/cgi-bin/locus.pl?locus=RRP43)*,* [*RRP5*](http://db.yeastgenome.org/cgi-bin/locus.pl?locus=RRP5)*,* [*RRP6*](http://db.yeastgenome.org/cgi-bin/locus.pl?locus=RRP6)*,* [*RRP8*](http://db.yeastgenome.org/cgi-bin/locus.pl?locus=RRP8)*,* [*RRP9*](http://db.yeastgenome.org/cgi-bin/locus.pl?locus=RRP9)*,* [*RRS1*](http://db.yeastgenome.org/cgi-bin/locus.pl?locus=RRS1)*, RRT14,* [*RSA1*](http://db.yeastgenome.org/cgi-bin/locus.pl?locus=RSA1)*,* [*RSA3*](http://db.yeastgenome.org/cgi-bin/locus.pl?locus=RSA3)*,* [*RSA4*](http://db.yeastgenome.org/cgi-bin/locus.pl?locus=RSA4)*,* [*SAS10*](http://db.yeastgenome.org/cgi-bin/locus.pl?locus=SAS10)*,* [*SDA1*](http://db.yeastgenome.org/cgi-bin/locus.pl?locus=SDA1)*, SEE1,* [*SIK1*](http://db.yeastgenome.org/cgi-bin/locus.pl?locus=SIK1)*,* [*SLX9*](http://db.yeastgenome.org/cgi-bin/locus.pl?locus=SLX9)*,* [*SNU13*](http://db.yeastgenome.org/cgi-bin/locus.pl?locus=SNU13)*,* [*SOF1*](http://db.yeastgenome.org/cgi-bin/locus.pl?locus=SOF1)*,* [*SPB1*](http://db.yeastgenome.org/cgi-bin/locus.pl?locus=SPB1)*,* [*SPB4*](http://db.yeastgenome.org/cgi-bin/locus.pl?locus=SPB4)*,* [*SQT1*](http://db.yeastgenome.org/cgi-bin/locus.pl?locus=SQT1)*,* [*SRO9*](http://db.yeastgenome.org/cgi-bin/locus.pl?locus=SRO9)*,* [*SRP40*](http://db.yeastgenome.org/cgi-bin/locus.pl?locus=SRP40)*,* [*SSF1*](http://db.yeastgenome.org/cgi-bin/locus.pl?locus=SSF1)*,* [*SSF2*](http://db.yeastgenome.org/cgi-bin/locus.pl?locus=SSF2)*,* [*TIF4631*](http://db.yeastgenome.org/cgi-bin/locus.pl?locus=TIF4631)*,* [*TMA23*](http://db.yeastgenome.org/cgi-bin/locus.pl?locus=TMA23)*,* [*TMA46*](http://db.yeastgenome.org/cgi-bin/locus.pl?locus=TMA46)*,* [*TOB6*](http://db.yeastgenome.org/cgi-bin/locus.pl?locus=YBL054W)*,* [*TPA1*](http://db.yeastgenome.org/cgi-bin/locus.pl?locus=TPA1)*,* [*TRM1*](http://db.yeastgenome.org/cgi-bin/locus.pl?locus=TRM1)*,* [*TRM11*](http://db.yeastgenome.org/cgi-bin/locus.pl?locus=TRM11)*,* [*TRM2*](http://db.yeastgenome.org/cgi-bin/locus.pl?locus=TRM2)*,* [*TRM8*](http://db.yeastgenome.org/cgi-bin/locus.pl?locus=TRM8)*,* [*TRM82*](http://db.yeastgenome.org/cgi-bin/locus.pl?locus=TRM82)*,* [*TSR1*](http://db.yeastgenome.org/cgi-bin/locus.pl?locus=TSR1)*,* [*TSR2*](http://db.yeastgenome.org/cgi-bin/locus.pl?locus=TSR2)*,* [*URB1*](http://db.yeastgenome.org/cgi-bin/locus.pl?locus=URB1)*,* [*URB2*](http://db.yeastgenome.org/cgi-bin/locus.pl?locus=URB2)*,* [*URK1*](http://db.yeastgenome.org/cgi-bin/locus.pl?locus=URK1)*,* [*UTP10*](http://db.yeastgenome.org/cgi-bin/locus.pl?locus=UTP10)*,* [*UTP11*](http://db.yeastgenome.org/cgi-bin/locus.pl?locus=UTP11)*,* [*UTP13*](http://db.yeastgenome.org/cgi-bin/locus.pl?locus=UTP13)*,* [*UTP14*](http://db.yeastgenome.org/cgi-bin/locus.pl?locus=UTP14)*,* [*UTP15*](http://db.yeastgenome.org/cgi-bin/locus.pl?locus=UTP15)*,* [*UTP18*](http://db.yeastgenome.org/cgi-bin/locus.pl?locus=UTP18)*,* [*UTP21*](http://db.yeastgenome.org/cgi-bin/locus.pl?locus=UTP21)*,* [*UTP22*](http://db.yeastgenome.org/cgi-bin/locus.pl?locus=UTP22)*,* [*UTP23*](http://db.yeastgenome.org/cgi-bin/locus.pl?locus=UTP23)*,* [*UTP30*](http://db.yeastgenome.org/cgi-bin/locus.pl?locus=UTP30)*,* [*UTP4*](http://db.yeastgenome.org/cgi-bin/locus.pl?locus=UTP4)*,* [*UTP5*](http://db.yeastgenome.org/cgi-bin/locus.pl?locus=UTP5)*,* [*UTP7*](http://db.yeastgenome.org/cgi-bin/locus.pl?locus=UTP7)*,* [*UTP8*](http://db.yeastgenome.org/cgi-bin/locus.pl?locus=UTP8)*,* [*UTP9*](http://db.yeastgenome.org/cgi-bin/locus.pl?locus=UTP9)*,* [*YAR1*](http://db.yeastgenome.org/cgi-bin/locus.pl?locus=YAR1)*,* [*YBL028C*](http://db.yeastgenome.org/cgi-bin/locus.pl?locus=YBL028C)*,* [*YBR238C*](http://db.yeastgenome.org/cgi-bin/locus.pl?locus=YBR238C)*,* [*YCR016W*](http://db.yeastgenome.org/cgi-bin/locus.pl?locus=YCR016W)*,* [*YDL063C*](http://db.yeastgenome.org/cgi-bin/locus.pl?locus=YDL063C)*,* [*YEF3*](http://db.yeastgenome.org/cgi-bin/locus.pl?locus=YEF3)*,* [*YGR283C*](http://db.yeastgenome.org/cgi-bin/locus.pl?locus=YGR283C)*,* [*YHL039W*](http://db.yeastgenome.org/cgi-bin/locus.pl?locus=YHL039W)*,* [*YIL096C*](http://db.yeastgenome.org/cgi-bin/locus.pl?locus=YIL096C)*,* [*YMR310C*](http://db.yeastgenome.org/cgi-bin/locus.pl?locus=YMR310C)*,* [*YNL022C*](http://db.yeastgenome.org/cgi-bin/locus.pl?locus=YNL022C)*,* [*YNL247W*](http://db.yeastgenome.org/cgi-bin/locus.pl?locus=YNL247W)*,* [*YOR021C*](http://db.yeastgenome.org/cgi-bin/locus.pl?locus=YOR021C)*,* [*YTM1*](http://db.yeastgenome.org/cgi-bin/locus.pl?locus=YTM1)*,* [*YVH1*](http://db.yeastgenome.org/cgi-bin/locus.pl?locus=YVH1) |
| Ribonucleoprotein complex biogenesis and assembly | 227 out of 558 genes, 4.0% | 3.71e-158 | [*AAH1*](http://db.yeastgenome.org/cgi-bin/locus.pl?locus=AAH1)*,* [*ALB1*](http://db.yeastgenome.org/cgi-bin/locus.pl?locus=ALB1)*,* [*ARB1*](http://db.yeastgenome.org/cgi-bin/locus.pl?locus=ARB1)*,* [*ARX1*](http://db.yeastgenome.org/cgi-bin/locus.pl?locus=ARX1)*,* [*ATC1*](http://db.yeastgenome.org/cgi-bin/locus.pl?locus=ATC1)*,* [*BCP1*](http://db.yeastgenome.org/cgi-bin/locus.pl?locus=BCP1)*,* [*BMS1*](http://db.yeastgenome.org/cgi-bin/locus.pl?locus=BMS1)*,* [*BRX1*](http://db.yeastgenome.org/cgi-bin/locus.pl?locus=BRX1)*,* [*BUD21*](http://db.yeastgenome.org/cgi-bin/locus.pl?locus=BUD21)*,* [*BUD22*](http://db.yeastgenome.org/cgi-bin/locus.pl?locus=BUD22)*,* [*CBF5*](http://db.yeastgenome.org/cgi-bin/locus.pl?locus=CBF5)*,* [*CGR1*](http://db.yeastgenome.org/cgi-bin/locus.pl?locus=CGR1)*,* [*CIC1*](http://db.yeastgenome.org/cgi-bin/locus.pl?locus=CIC1)*, CMS1,* [*CUS1*](http://db.yeastgenome.org/cgi-bin/locus.pl?locus=CUS1)*,* [*DBP10*](http://db.yeastgenome.org/cgi-bin/locus.pl?locus=DBP10)*,* [*DBP2*](http://db.yeastgenome.org/cgi-bin/locus.pl?locus=DBP2)*,* [*DBP3*](http://db.yeastgenome.org/cgi-bin/locus.pl?locus=DBP3)*,* [*DBP6*](http://db.yeastgenome.org/cgi-bin/locus.pl?locus=DBP6)*,* [*DBP7*](http://db.yeastgenome.org/cgi-bin/locus.pl?locus=DBP7)*,* [*DBP8*](http://db.yeastgenome.org/cgi-bin/locus.pl?locus=DBP8)*,* [*DBP9*](http://db.yeastgenome.org/cgi-bin/locus.pl?locus=DBP9)*,* [*DHR2*](http://db.yeastgenome.org/cgi-bin/locus.pl?locus=DHR2)*,* [*DIM1*](http://db.yeastgenome.org/cgi-bin/locus.pl?locus=DIM1)*,* [*DIP2*](http://db.yeastgenome.org/cgi-bin/locus.pl?locus=DIP2)*,* [*DPH2*](http://db.yeastgenome.org/cgi-bin/locus.pl?locus=DPH2)*,* [*DRS1*](http://db.yeastgenome.org/cgi-bin/locus.pl?locus=DRS1)*,* [*DUS3*](http://db.yeastgenome.org/cgi-bin/locus.pl?locus=DUS3)*,* [*EBP2*](http://db.yeastgenome.org/cgi-bin/locus.pl?locus=EBP2)*,* [*ECM16*](http://db.yeastgenome.org/cgi-bin/locus.pl?locus=ECM16)*, EFG1,* [*EMG1*](http://db.yeastgenome.org/cgi-bin/locus.pl?locus=EMG1)*,* [*ENP1*](http://db.yeastgenome.org/cgi-bin/locus.pl?locus=ENP1)*,* [*ENP2*](http://db.yeastgenome.org/cgi-bin/locus.pl?locus=ENP2)*,* [*ERB1*](http://db.yeastgenome.org/cgi-bin/locus.pl?locus=ERB1)*,* [*ESF1*](http://db.yeastgenome.org/cgi-bin/locus.pl?locus=ESF1)*,* [*ESF2*](http://db.yeastgenome.org/cgi-bin/locus.pl?locus=ESF2)*,* [*FAF1*](http://db.yeastgenome.org/cgi-bin/locus.pl?locus=FAF1)*,* [*FAL1*](http://db.yeastgenome.org/cgi-bin/locus.pl?locus=FAL1)*,* [*FAP7*](http://db.yeastgenome.org/cgi-bin/locus.pl?locus=FAP7)*,* [*FCF2*](http://db.yeastgenome.org/cgi-bin/locus.pl?locus=FCF2)*,* [*FPR4*](http://db.yeastgenome.org/cgi-bin/locus.pl?locus=FPR4)*,* [*FYV7*](http://db.yeastgenome.org/cgi-bin/locus.pl?locus=FYV7)*,* [*GAR1*](http://db.yeastgenome.org/cgi-bin/locus.pl?locus=GAR1)*,* [*GCD10*](http://db.yeastgenome.org/cgi-bin/locus.pl?locus=GCD10)*,* [*GCD11*](http://db.yeastgenome.org/cgi-bin/locus.pl?locus=GCD11)*,* [*GCD14*](http://db.yeastgenome.org/cgi-bin/locus.pl?locus=GCD14)*,* [*GCD2*](http://db.yeastgenome.org/cgi-bin/locus.pl?locus=GCD2)*,* [*GCN3*](http://db.yeastgenome.org/cgi-bin/locus.pl?locus=GCN3)*,* [*GDT1*](http://db.yeastgenome.org/cgi-bin/locus.pl?locus=GDT1)*,* [*GRC3*](http://db.yeastgenome.org/cgi-bin/locus.pl?locus=GRC3)*,* [*HAS1*](http://db.yeastgenome.org/cgi-bin/locus.pl?locus=HAS1)*,* [*HCA4*](http://db.yeastgenome.org/cgi-bin/locus.pl?locus=HCA4)*,* [*HGH1*](http://db.yeastgenome.org/cgi-bin/locus.pl?locus=HGH1)*,* [*HMT1*](http://db.yeastgenome.org/cgi-bin/locus.pl?locus=HMT1)*,* [*IMP3*](http://db.yeastgenome.org/cgi-bin/locus.pl?locus=IMP3)*,* [*IPI1*](http://db.yeastgenome.org/cgi-bin/locus.pl?locus=IPI1)*,* [*IPI3*](http://db.yeastgenome.org/cgi-bin/locus.pl?locus=IPI3)*,* [*KRE33*](http://db.yeastgenome.org/cgi-bin/locus.pl?locus=KRE33)*,* [*KRI1*](http://db.yeastgenome.org/cgi-bin/locus.pl?locus=KRI1)*,* [*KRR1*](http://db.yeastgenome.org/cgi-bin/locus.pl?locus=KRR1)*,* [*LCP5*](http://db.yeastgenome.org/cgi-bin/locus.pl?locus=LCP5)*,* [*LIA1*](http://db.yeastgenome.org/cgi-bin/locus.pl?locus=LIA1)*,* [*LOC1*](http://db.yeastgenome.org/cgi-bin/locus.pl?locus=LOC1)*,* [*LSG1*](http://db.yeastgenome.org/cgi-bin/locus.pl?locus=LSG1)*,* [*MAK11*](http://db.yeastgenome.org/cgi-bin/locus.pl?locus=MAK11)*,* [*MAK16*](http://db.yeastgenome.org/cgi-bin/locus.pl?locus=MAK16)*,* [*MAK21*](http://db.yeastgenome.org/cgi-bin/locus.pl?locus=MAK21)*,* [*MIS1*](http://db.yeastgenome.org/cgi-bin/locus.pl?locus=MIS1)*,* [*MOT1*](http://db.yeastgenome.org/cgi-bin/locus.pl?locus=MOT1)*,* [*MPP10*](http://db.yeastgenome.org/cgi-bin/locus.pl?locus=MPP10)*,* [*MRD1*](http://db.yeastgenome.org/cgi-bin/locus.pl?locus=MRD1)*,* [*MRT4*](http://db.yeastgenome.org/cgi-bin/locus.pl?locus=MRT4)*,* [*MTR3*](http://db.yeastgenome.org/cgi-bin/locus.pl?locus=MTR3)*,* [*NAN1*](http://db.yeastgenome.org/cgi-bin/locus.pl?locus=NAN1)*,* [*NCL1*](http://db.yeastgenome.org/cgi-bin/locus.pl?locus=NCL1)*,* [*NCS2*](http://db.yeastgenome.org/cgi-bin/locus.pl?locus=NCS2)*,* [*NEW1*](http://db.yeastgenome.org/cgi-bin/locus.pl?locus=NEW1)*,* [*NHP2*](http://db.yeastgenome.org/cgi-bin/locus.pl?locus=NHP2)*,* [*NIP7*](http://db.yeastgenome.org/cgi-bin/locus.pl?locus=NIP7)*,* [*NMD3*](http://db.yeastgenome.org/cgi-bin/locus.pl?locus=NMD3)*,* [*NOB1*](http://db.yeastgenome.org/cgi-bin/locus.pl?locus=NOB1)*,* [*NOC2*](http://db.yeastgenome.org/cgi-bin/locus.pl?locus=NOC2)*,* [*NOC3*](http://db.yeastgenome.org/cgi-bin/locus.pl?locus=NOC3)*,* [*NOC4*](http://db.yeastgenome.org/cgi-bin/locus.pl?locus=NOC4)*,* [*NOG1*](http://db.yeastgenome.org/cgi-bin/locus.pl?locus=NOG1)*,* [*NOG2*](http://db.yeastgenome.org/cgi-bin/locus.pl?locus=NOG2)*,* [*NOP13*](http://db.yeastgenome.org/cgi-bin/locus.pl?locus=NOP13)*,* [*NOP14*](http://db.yeastgenome.org/cgi-bin/locus.pl?locus=NOP14)*,* [*NOP15*](http://db.yeastgenome.org/cgi-bin/locus.pl?locus=NOP15)*,* [*NOP16*](http://db.yeastgenome.org/cgi-bin/locus.pl?locus=NOP16)*,* [*NOP2*](http://db.yeastgenome.org/cgi-bin/locus.pl?locus=NOP2)*,* [*NOP4*](http://db.yeastgenome.org/cgi-bin/locus.pl?locus=NOP4)*,* [*NOP53*](http://db.yeastgenome.org/cgi-bin/locus.pl?locus=NOP53)*,* [*NOP58*](http://db.yeastgenome.org/cgi-bin/locus.pl?locus=NOP58)*,* [*NOP6*](http://db.yeastgenome.org/cgi-bin/locus.pl?locus=NOP6)*,* [*NOP7*](http://db.yeastgenome.org/cgi-bin/locus.pl?locus=NOP7)*,* [*NOP8*](http://db.yeastgenome.org/cgi-bin/locus.pl?locus=NOP8)*,* [*NOP9*](http://db.yeastgenome.org/cgi-bin/locus.pl?locus=NOP9)*,* [*NRP1*](http://db.yeastgenome.org/cgi-bin/locus.pl?locus=NRP1)*,* [*NSA1*](http://db.yeastgenome.org/cgi-bin/locus.pl?locus=NSA1)*,* [*NSR1*](http://db.yeastgenome.org/cgi-bin/locus.pl?locus=NSR1)*,* [*NUC1*](http://db.yeastgenome.org/cgi-bin/locus.pl?locus=NUC1)*,* [*NUG1*](http://db.yeastgenome.org/cgi-bin/locus.pl?locus=NUG1)*,* [*PNO1*](http://db.yeastgenome.org/cgi-bin/locus.pl?locus=PNO1)*,* [*POP3*](http://db.yeastgenome.org/cgi-bin/locus.pl?locus=POP3)*,* [*POP6*](http://db.yeastgenome.org/cgi-bin/locus.pl?locus=POP6)*,* [*POP8*](http://db.yeastgenome.org/cgi-bin/locus.pl?locus=POP8)*,* [*PPT1*](http://db.yeastgenome.org/cgi-bin/locus.pl?locus=PPT1)*,* [*PRO1*](http://db.yeastgenome.org/cgi-bin/locus.pl?locus=PRO1)*,* [*PRP24*](http://db.yeastgenome.org/cgi-bin/locus.pl?locus=PRP24)*,* [*PRP43*](http://db.yeastgenome.org/cgi-bin/locus.pl?locus=PRP43)*,* [*PRS4*](http://db.yeastgenome.org/cgi-bin/locus.pl?locus=PRS4)*,* [*PUF6*](http://db.yeastgenome.org/cgi-bin/locus.pl?locus=PUF6)*,* [*PUS1*](http://db.yeastgenome.org/cgi-bin/locus.pl?locus=PUS1)*,* [*PUS7*](http://db.yeastgenome.org/cgi-bin/locus.pl?locus=PUS7)*,* [*PWP1*](http://db.yeastgenome.org/cgi-bin/locus.pl?locus=PWP1)*,* [*PWP2*](http://db.yeastgenome.org/cgi-bin/locus.pl?locus=PWP2)*,* [*PXR1*](http://db.yeastgenome.org/cgi-bin/locus.pl?locus=PXR1)*,* [*RBG1*](http://db.yeastgenome.org/cgi-bin/locus.pl?locus=RBG1)*,* [*RCL1*](http://db.yeastgenome.org/cgi-bin/locus.pl?locus=RCL1)*,* [*REI1*](http://db.yeastgenome.org/cgi-bin/locus.pl?locus=REI1)*,* [*REX4*](http://db.yeastgenome.org/cgi-bin/locus.pl?locus=REX4)*,* [*RIO1*](http://db.yeastgenome.org/cgi-bin/locus.pl?locus=RIO1)*,* [*RIX1*](http://db.yeastgenome.org/cgi-bin/locus.pl?locus=RIX1)*,* [*RIX7*](http://db.yeastgenome.org/cgi-bin/locus.pl?locus=RIX7)*,* [*RKI1*](http://db.yeastgenome.org/cgi-bin/locus.pl?locus=RKI1)*,* [*RLI1*](http://db.yeastgenome.org/cgi-bin/locus.pl?locus=RLI1)*,* [*RLP24*](http://db.yeastgenome.org/cgi-bin/locus.pl?locus=RLP24)*,* [*RLP7*](http://db.yeastgenome.org/cgi-bin/locus.pl?locus=RLP7)*,* [*RMT2*](http://db.yeastgenome.org/cgi-bin/locus.pl?locus=RMT2)*,* [*RNT1*](http://db.yeastgenome.org/cgi-bin/locus.pl?locus=RNT1)*,* [*ROK1*](http://db.yeastgenome.org/cgi-bin/locus.pl?locus=ROK1)*,* [*RPA12*](http://db.yeastgenome.org/cgi-bin/locus.pl?locus=RPA12)*,* [*RPA34*](http://db.yeastgenome.org/cgi-bin/locus.pl?locus=RPA34)*,* [*RPA43*](http://db.yeastgenome.org/cgi-bin/locus.pl?locus=RPA43)*,* [*RPA49*](http://db.yeastgenome.org/cgi-bin/locus.pl?locus=RPA49)*,* [*RPC19*](http://db.yeastgenome.org/cgi-bin/locus.pl?locus=RPC19)*,* [*RPC34*](http://db.yeastgenome.org/cgi-bin/locus.pl?locus=RPC34)*,* [*RPC40*](http://db.yeastgenome.org/cgi-bin/locus.pl?locus=RPC40)*,* [*RPC82*](http://db.yeastgenome.org/cgi-bin/locus.pl?locus=RPC82)*,* [*RPF1*](http://db.yeastgenome.org/cgi-bin/locus.pl?locus=RPF1)*,* [*RPF2*](http://db.yeastgenome.org/cgi-bin/locus.pl?locus=RPF2)*,* [*RPP1*](http://db.yeastgenome.org/cgi-bin/locus.pl?locus=RPP1)*,* [*RPS14B*](http://db.yeastgenome.org/cgi-bin/locus.pl?locus=RPS14B)*,* [*RPS19B*](http://db.yeastgenome.org/cgi-bin/locus.pl?locus=RPS19B)*,* [*RRB1*](http://db.yeastgenome.org/cgi-bin/locus.pl?locus=RRB1)*,* [*RRP1*](http://db.yeastgenome.org/cgi-bin/locus.pl?locus=RRP1)*,* [*RRP12*](http://db.yeastgenome.org/cgi-bin/locus.pl?locus=RRP12)*,* [*RRP14*](http://db.yeastgenome.org/cgi-bin/locus.pl?locus=RRP14)*,* [*RRP15*](http://db.yeastgenome.org/cgi-bin/locus.pl?locus=RRP15)*,* [*RRP17*](http://db.yeastgenome.org/cgi-bin/locus.pl?locus=RRP17)*,* [*RRP3*](http://db.yeastgenome.org/cgi-bin/locus.pl?locus=RRP3) *RRP36,* [*RRP40*](http://db.yeastgenome.org/cgi-bin/locus.pl?locus=RRP40)*,* [*RRP43*](http://db.yeastgenome.org/cgi-bin/locus.pl?locus=RRP43)*,* [*RRP5*](http://db.yeastgenome.org/cgi-bin/locus.pl?locus=RRP5)*,* [*RRP6*](http://db.yeastgenome.org/cgi-bin/locus.pl?locus=RRP6)*,* [*RRP8*](http://db.yeastgenome.org/cgi-bin/locus.pl?locus=RRP8)*,* [*RRP9*](http://db.yeastgenome.org/cgi-bin/locus.pl?locus=RRP9)*,* [*RRS1*](http://db.yeastgenome.org/cgi-bin/locus.pl?locus=RRS1)*, RRT14,* [*RSA1*](http://db.yeastgenome.org/cgi-bin/locus.pl?locus=RSA1)*,* [*RSA3*](http://db.yeastgenome.org/cgi-bin/locus.pl?locus=RSA3)*,* [*RSA4*](http://db.yeastgenome.org/cgi-bin/locus.pl?locus=RSA4)*,* [*SAS10*](http://db.yeastgenome.org/cgi-bin/locus.pl?locus=SAS10)*,* [*SDA1*](http://db.yeastgenome.org/cgi-bin/locus.pl?locus=SDA1)*, SEE1,* [*SIK1*](http://db.yeastgenome.org/cgi-bin/locus.pl?locus=SIK1)*,* [*SLX9*](http://db.yeastgenome.org/cgi-bin/locus.pl?locus=SLX9)*,* [*SNU13*](http://db.yeastgenome.org/cgi-bin/locus.pl?locus=SNU13)*,* [*SOF1*](http://db.yeastgenome.org/cgi-bin/locus.pl?locus=SOF1)*,* [*SPB1*](http://db.yeastgenome.org/cgi-bin/locus.pl?locus=SPB1)*,* [*SPB4*](http://db.yeastgenome.org/cgi-bin/locus.pl?locus=SPB4)*,* [*SQT1*](http://db.yeastgenome.org/cgi-bin/locus.pl?locus=SQT1)*,* [*SRO9*](http://db.yeastgenome.org/cgi-bin/locus.pl?locus=SRO9)*,* [*SRP40*](http://db.yeastgenome.org/cgi-bin/locus.pl?locus=SRP40)*,* [*SSF1*](http://db.yeastgenome.org/cgi-bin/locus.pl?locus=SSF1)*,* [*SSF2*](http://db.yeastgenome.org/cgi-bin/locus.pl?locus=SSF2)*,* [*TIF2*](http://db.yeastgenome.org/cgi-bin/locus.pl?locus=TIF2)*,* [*TIF3*](http://db.yeastgenome.org/cgi-bin/locus.pl?locus=TIF3)*,* [*TIF35*](http://db.yeastgenome.org/cgi-bin/locus.pl?locus=TIF35)*,* [*TIF4631*](http://db.yeastgenome.org/cgi-bin/locus.pl?locus=TIF4631)*,* [*TMA23*](http://db.yeastgenome.org/cgi-bin/locus.pl?locus=TMA23)*,* [*TMA46*](http://db.yeastgenome.org/cgi-bin/locus.pl?locus=TMA46)*,* [*TOB6*](http://db.yeastgenome.org/cgi-bin/locus.pl?locus=YBL054W)*,* [*TPA1*](http://db.yeastgenome.org/cgi-bin/locus.pl?locus=TPA1)*,* [*TRM1*](http://db.yeastgenome.org/cgi-bin/locus.pl?locus=TRM1)*,* [*TRM11*](http://db.yeastgenome.org/cgi-bin/locus.pl?locus=TRM11)*,* [*TRM2*](http://db.yeastgenome.org/cgi-bin/locus.pl?locus=TRM2)*,* [*TRM8*](http://db.yeastgenome.org/cgi-bin/locus.pl?locus=TRM8)*,* [*TRM82*](http://db.yeastgenome.org/cgi-bin/locus.pl?locus=TRM82)*,* [*TSR1*](http://db.yeastgenome.org/cgi-bin/locus.pl?locus=TSR1)*,* [*TSR2*](http://db.yeastgenome.org/cgi-bin/locus.pl?locus=TSR2)*,* [*URB1*](http://db.yeastgenome.org/cgi-bin/locus.pl?locus=URB1)*,* [*URB2*](http://db.yeastgenome.org/cgi-bin/locus.pl?locus=URB2)*,* [*URK1*](http://db.yeastgenome.org/cgi-bin/locus.pl?locus=URK1)*,* [*UTP10*](http://db.yeastgenome.org/cgi-bin/locus.pl?locus=UTP10)*,* [*UTP11*](http://db.yeastgenome.org/cgi-bin/locus.pl?locus=UTP11)*,* [*UTP13*](http://db.yeastgenome.org/cgi-bin/locus.pl?locus=UTP13)*,* [*UTP14*](http://db.yeastgenome.org/cgi-bin/locus.pl?locus=UTP14)*,* [*UTP15*](http://db.yeastgenome.org/cgi-bin/locus.pl?locus=UTP15)*,* [*UTP18*](http://db.yeastgenome.org/cgi-bin/locus.pl?locus=UTP18)*,* [*UTP21*](http://db.yeastgenome.org/cgi-bin/locus.pl?locus=UTP21)*,* [*UTP22*](http://db.yeastgenome.org/cgi-bin/locus.pl?locus=UTP22)*,* [*UTP23*](http://db.yeastgenome.org/cgi-bin/locus.pl?locus=UTP23)*,* [*UTP30*](http://db.yeastgenome.org/cgi-bin/locus.pl?locus=UTP30)*,* [*UTP4*](http://db.yeastgenome.org/cgi-bin/locus.pl?locus=UTP4)*,* [*UTP5*](http://db.yeastgenome.org/cgi-bin/locus.pl?locus=UTP5)*,* [*UTP7*](http://db.yeastgenome.org/cgi-bin/locus.pl?locus=UTP7)*,* [*UTP8*](http://db.yeastgenome.org/cgi-bin/locus.pl?locus=UTP8)*,* [*UTP9*](http://db.yeastgenome.org/cgi-bin/locus.pl?locus=UTP9)*,* [*YAR1*](http://db.yeastgenome.org/cgi-bin/locus.pl?locus=YAR1)*,* [*YBL028C*](http://db.yeastgenome.org/cgi-bin/locus.pl?locus=YBL028C)*,* [*YBR238C*](http://db.yeastgenome.org/cgi-bin/locus.pl?locus=YBR238C)*,* [*YCR016W*](http://db.yeastgenome.org/cgi-bin/locus.pl?locus=YCR016W)*,* [*YDL063C*](http://db.yeastgenome.org/cgi-bin/locus.pl?locus=YDL063C)*,* [*YEF3*](http://db.yeastgenome.org/cgi-bin/locus.pl?locus=YEF3)*,* [*YGR283C*](http://db.yeastgenome.org/cgi-bin/locus.pl?locus=YGR283C)*,* [*YHL039W*](http://db.yeastgenome.org/cgi-bin/locus.pl?locus=YHL039W)*,* [*YIL096C*](http://db.yeastgenome.org/cgi-bin/locus.pl?locus=YIL096C)*,* [*YMR310C*](http://db.yeastgenome.org/cgi-bin/locus.pl?locus=YMR310C)*,* [*YNL022C*](http://db.yeastgenome.org/cgi-bin/locus.pl?locus=YNL022C)*,* [*YNL247W*](http://db.yeastgenome.org/cgi-bin/locus.pl?locus=YNL247W)*,* [*YOR021C*](http://db.yeastgenome.org/cgi-bin/locus.pl?locus=YOR021C)*,* [*YTM1*](http://db.yeastgenome.org/cgi-bin/locus.pl?locus=YTM1)*,* [*YVH1*](http://db.yeastgenome.org/cgi-bin/locus.pl?locus=YVH1) |
| rRNA processing | 120 out of 558 genes, 5.5% | 3.50e-92 | [*BMS1*](http://db.yeastgenome.org/cgi-bin/locus.pl?locus=BMS1)*,* [*BUD21*](http://db.yeastgenome.org/cgi-bin/locus.pl?locus=BUD21)*,* [*CBF5*](http://db.yeastgenome.org/cgi-bin/locus.pl?locus=CBF5)*,* [*CGR1*](http://db.yeastgenome.org/cgi-bin/locus.pl?locus=CGR1)*,* [*DBP10*](http://db.yeastgenome.org/cgi-bin/locus.pl?locus=DBP10)*,* [*DBP2*](http://db.yeastgenome.org/cgi-bin/locus.pl?locus=DBP2)*,* [*DBP3*](http://db.yeastgenome.org/cgi-bin/locus.pl?locus=DBP3)*,* [*DBP6*](http://db.yeastgenome.org/cgi-bin/locus.pl?locus=DBP6)*,* [*DBP7*](http://db.yeastgenome.org/cgi-bin/locus.pl?locus=DBP7)*,* [*DBP8*](http://db.yeastgenome.org/cgi-bin/locus.pl?locus=DBP8)*,* [*DBP9*](http://db.yeastgenome.org/cgi-bin/locus.pl?locus=DBP9)*,* [*DIM1*](http://db.yeastgenome.org/cgi-bin/locus.pl?locus=DIM1)*,* [*DIP2*](http://db.yeastgenome.org/cgi-bin/locus.pl?locus=DIP2)*,* [*DRS1*](http://db.yeastgenome.org/cgi-bin/locus.pl?locus=DRS1)*,* [*EBP2*](http://db.yeastgenome.org/cgi-bin/locus.pl?locus=EBP2)*,* [*ECM16*](http://db.yeastgenome.org/cgi-bin/locus.pl?locus=ECM16)*, EFG1,* [*EMG1*](http://db.yeastgenome.org/cgi-bin/locus.pl?locus=EMG1)*,* [*ENP1*](http://db.yeastgenome.org/cgi-bin/locus.pl?locus=ENP1)*,* [*ENP2*](http://db.yeastgenome.org/cgi-bin/locus.pl?locus=ENP2)*,* [*ERB1*](http://db.yeastgenome.org/cgi-bin/locus.pl?locus=ERB1)*,* [*ESF1*](http://db.yeastgenome.org/cgi-bin/locus.pl?locus=ESF1)*,* [*ESF2*](http://db.yeastgenome.org/cgi-bin/locus.pl?locus=ESF2)*,* [*FAF1*](http://db.yeastgenome.org/cgi-bin/locus.pl?locus=FAF1)*,* [*FAL1*](http://db.yeastgenome.org/cgi-bin/locus.pl?locus=FAL1)*,* [*FAP7*](http://db.yeastgenome.org/cgi-bin/locus.pl?locus=FAP7)*,* [*FCF2*](http://db.yeastgenome.org/cgi-bin/locus.pl?locus=FCF2)*,* [*FYV7*](http://db.yeastgenome.org/cgi-bin/locus.pl?locus=FYV7)*,* [*GAR1*](http://db.yeastgenome.org/cgi-bin/locus.pl?locus=GAR1)*,* [*GRC3*](http://db.yeastgenome.org/cgi-bin/locus.pl?locus=GRC3)*,* [*HAS1*](http://db.yeastgenome.org/cgi-bin/locus.pl?locus=HAS1)*,* [*HCA4*](http://db.yeastgenome.org/cgi-bin/locus.pl?locus=HCA4)*,* [*IMP3*](http://db.yeastgenome.org/cgi-bin/locus.pl?locus=IMP3)*,* [*IPI1*](http://db.yeastgenome.org/cgi-bin/locus.pl?locus=IPI1)*,* [*IPI3*](http://db.yeastgenome.org/cgi-bin/locus.pl?locus=IPI3)*,* [*KRR1*](http://db.yeastgenome.org/cgi-bin/locus.pl?locus=KRR1)*,* [*LCP5*](http://db.yeastgenome.org/cgi-bin/locus.pl?locus=LCP5)*,* [*MAK11*](http://db.yeastgenome.org/cgi-bin/locus.pl?locus=MAK11)*,* [*MAK16*](http://db.yeastgenome.org/cgi-bin/locus.pl?locus=MAK16)*,* [*MOT1*](http://db.yeastgenome.org/cgi-bin/locus.pl?locus=MOT1)*,* [*MPP10*](http://db.yeastgenome.org/cgi-bin/locus.pl?locus=MPP10)*,* [*MRD1*](http://db.yeastgenome.org/cgi-bin/locus.pl?locus=MRD1)*,* [*MRT4*](http://db.yeastgenome.org/cgi-bin/locus.pl?locus=MRT4)*,* [*MTR3*](http://db.yeastgenome.org/cgi-bin/locus.pl?locus=MTR3)*,* [*NAN1*](http://db.yeastgenome.org/cgi-bin/locus.pl?locus=NAN1)*,* [*NHP2*](http://db.yeastgenome.org/cgi-bin/locus.pl?locus=NHP2)*,* [*NIP7*](http://db.yeastgenome.org/cgi-bin/locus.pl?locus=NIP7)*,* [*NOB1*](http://db.yeastgenome.org/cgi-bin/locus.pl?locus=NOB1)*,* [*NOC3*](http://db.yeastgenome.org/cgi-bin/locus.pl?locus=NOC3)*,* [*NOC4*](http://db.yeastgenome.org/cgi-bin/locus.pl?locus=NOC4)*,* [*NOG1*](http://db.yeastgenome.org/cgi-bin/locus.pl?locus=NOG1)*,* [*NOP14*](http://db.yeastgenome.org/cgi-bin/locus.pl?locus=NOP14)*,* [*NOP2*](http://db.yeastgenome.org/cgi-bin/locus.pl?locus=NOP2)*,* [*NOP4*](http://db.yeastgenome.org/cgi-bin/locus.pl?locus=NOP4)*,* [*NOP53*](http://db.yeastgenome.org/cgi-bin/locus.pl?locus=NOP53)*,* [*NOP58*](http://db.yeastgenome.org/cgi-bin/locus.pl?locus=NOP58)*,* [*NOP6*](http://db.yeastgenome.org/cgi-bin/locus.pl?locus=NOP6)*,* [*NOP7*](http://db.yeastgenome.org/cgi-bin/locus.pl?locus=NOP7)*,* [*NOP8*](http://db.yeastgenome.org/cgi-bin/locus.pl?locus=NOP8)*,* [*NOP9*](http://db.yeastgenome.org/cgi-bin/locus.pl?locus=NOP9)*,* [*NSR1*](http://db.yeastgenome.org/cgi-bin/locus.pl?locus=NSR1)*,* [*NUG1*](http://db.yeastgenome.org/cgi-bin/locus.pl?locus=NUG1)*,* [*PNO1*](http://db.yeastgenome.org/cgi-bin/locus.pl?locus=PNO1)*,* [*POP3*](http://db.yeastgenome.org/cgi-bin/locus.pl?locus=POP3)*,* [*POP6*](http://db.yeastgenome.org/cgi-bin/locus.pl?locus=POP6)*,* [*POP8*](http://db.yeastgenome.org/cgi-bin/locus.pl?locus=POP8)*,* [*PRP43*](http://db.yeastgenome.org/cgi-bin/locus.pl?locus=PRP43)*,* [*PWP1*](http://db.yeastgenome.org/cgi-bin/locus.pl?locus=PWP1)*,* [*PWP2*](http://db.yeastgenome.org/cgi-bin/locus.pl?locus=PWP2)*,* [*PXR1*](http://db.yeastgenome.org/cgi-bin/locus.pl?locus=PXR1)*,* [*RCL1*](http://db.yeastgenome.org/cgi-bin/locus.pl?locus=RCL1)*,* [*REX4*](http://db.yeastgenome.org/cgi-bin/locus.pl?locus=REX4)*,* [*RIO1*](http://db.yeastgenome.org/cgi-bin/locus.pl?locus=RIO1)*,* [*RIX1*](http://db.yeastgenome.org/cgi-bin/locus.pl?locus=RIX1)*,* [*RLP7*](http://db.yeastgenome.org/cgi-bin/locus.pl?locus=RLP7)*,* [*RNT1*](http://db.yeastgenome.org/cgi-bin/locus.pl?locus=RNT1)*,* [*ROK1*](http://db.yeastgenome.org/cgi-bin/locus.pl?locus=ROK1)*,* [*RPF1*](http://db.yeastgenome.org/cgi-bin/locus.pl?locus=RPF1)*,* [*RPF2*](http://db.yeastgenome.org/cgi-bin/locus.pl?locus=RPF2)*,* [*RPP1*](http://db.yeastgenome.org/cgi-bin/locus.pl?locus=RPP1)*,* [*RPS14B*](http://db.yeastgenome.org/cgi-bin/locus.pl?locus=RPS14B)*,* [*RPS19B*](http://db.yeastgenome.org/cgi-bin/locus.pl?locus=RPS19B)*,* [*RRP1*](http://db.yeastgenome.org/cgi-bin/locus.pl?locus=RRP1)*,* [*RRP12*](http://db.yeastgenome.org/cgi-bin/locus.pl?locus=RRP12)*,* [*RRP15*](http://db.yeastgenome.org/cgi-bin/locus.pl?locus=RRP15)*,* [*RRP17*](http://db.yeastgenome.org/cgi-bin/locus.pl?locus=RRP17)*,* [*RRP3*](http://db.yeastgenome.org/cgi-bin/locus.pl?locus=RRP3)*, RRP36,* [*RRP40*](http://db.yeastgenome.org/cgi-bin/locus.pl?locus=RRP40)*,* [*RRP43*](http://db.yeastgenome.org/cgi-bin/locus.pl?locus=RRP43)*,* [*RRP5*](http://db.yeastgenome.org/cgi-bin/locus.pl?locus=RRP5)*,* [*RRP6*](http://db.yeastgenome.org/cgi-bin/locus.pl?locus=RRP6)*,* [*RRP8*](http://db.yeastgenome.org/cgi-bin/locus.pl?locus=RRP8)*,* [*RRP9*](http://db.yeastgenome.org/cgi-bin/locus.pl?locus=RRP9)*,* [*RRS1*](http://db.yeastgenome.org/cgi-bin/locus.pl?locus=RRS1)*,* [*SAS10*](http://db.yeastgenome.org/cgi-bin/locus.pl?locus=SAS10)*,* [*SIK1*](http://db.yeastgenome.org/cgi-bin/locus.pl?locus=SIK1)*,* [*SLX9*](http://db.yeastgenome.org/cgi-bin/locus.pl?locus=SLX9)*,* [*SNU13*](http://db.yeastgenome.org/cgi-bin/locus.pl?locus=SNU13)*,* [*SOF1*](http://db.yeastgenome.org/cgi-bin/locus.pl?locus=SOF1)*,* [*SPB1*](http://db.yeastgenome.org/cgi-bin/locus.pl?locus=SPB1)*,* [*SPB4*](http://db.yeastgenome.org/cgi-bin/locus.pl?locus=SPB4)*,* [*TSR1*](http://db.yeastgenome.org/cgi-bin/locus.pl?locus=TSR1)*,* [*TSR2*](http://db.yeastgenome.org/cgi-bin/locus.pl?locus=TSR2)*,* [*URB1*](http://db.yeastgenome.org/cgi-bin/locus.pl?locus=URB1)*,* [*UTP10*](http://db.yeastgenome.org/cgi-bin/locus.pl?locus=UTP10)*,* [*UTP11*](http://db.yeastgenome.org/cgi-bin/locus.pl?locus=UTP11)*,* [*UTP13*](http://db.yeastgenome.org/cgi-bin/locus.pl?locus=UTP13)*,* [*UTP14*](http://db.yeastgenome.org/cgi-bin/locus.pl?locus=UTP14)*,* [*UTP15*](http://db.yeastgenome.org/cgi-bin/locus.pl?locus=UTP15)*,* [*UTP18*](http://db.yeastgenome.org/cgi-bin/locus.pl?locus=UTP18)*,* [*UTP21*](http://db.yeastgenome.org/cgi-bin/locus.pl?locus=UTP21)*,* [*UTP22*](http://db.yeastgenome.org/cgi-bin/locus.pl?locus=UTP22)*,* [*UTP23*](http://db.yeastgenome.org/cgi-bin/locus.pl?locus=UTP23)*,* [*UTP30*](http://db.yeastgenome.org/cgi-bin/locus.pl?locus=UTP30)*,* [*UTP4*](http://db.yeastgenome.org/cgi-bin/locus.pl?locus=UTP4)*,* [*UTP5*](http://db.yeastgenome.org/cgi-bin/locus.pl?locus=UTP5)*,* [*UTP7*](http://db.yeastgenome.org/cgi-bin/locus.pl?locus=UTP7)*,* [*UTP8*](http://db.yeastgenome.org/cgi-bin/locus.pl?locus=UTP8)*,* [*UTP9*](http://db.yeastgenome.org/cgi-bin/locus.pl?locus=UTP9) |
| Organelle organization and biogenesis | 283 out of 558 genes, 50.7% | 1.18e-73 | [*AAH1*](http://db.yeastgenome.org/cgi-bin/locus.pl?locus=AAH1)*,* [*ALB1*](http://db.yeastgenome.org/cgi-bin/locus.pl?locus=ALB1)*,* [*ARB1*](http://db.yeastgenome.org/cgi-bin/locus.pl?locus=ARB1)*,* [*ARP1*](http://db.yeastgenome.org/cgi-bin/locus.pl?locus=ARP1)*,* [*ARX1*](http://db.yeastgenome.org/cgi-bin/locus.pl?locus=ARX1)*,* [*ASC1*](http://db.yeastgenome.org/cgi-bin/locus.pl?locus=ASC1)*,* [*ATC1*](http://db.yeastgenome.org/cgi-bin/locus.pl?locus=ATC1)*,* [*BCP1*](http://db.yeastgenome.org/cgi-bin/locus.pl?locus=BCP1)*,* [*BMS1*](http://db.yeastgenome.org/cgi-bin/locus.pl?locus=BMS1)*,* [*BRR6*](http://db.yeastgenome.org/cgi-bin/locus.pl?locus=BRR6)*,* [*BRX1*](http://db.yeastgenome.org/cgi-bin/locus.pl?locus=BRX1)*,* [*BST1*](http://db.yeastgenome.org/cgi-bin/locus.pl?locus=BST1)*,* [*BUD21*](http://db.yeastgenome.org/cgi-bin/locus.pl?locus=BUD21)*,* [*BUD22*](http://db.yeastgenome.org/cgi-bin/locus.pl?locus=BUD22)*,* [*BUD32*](http://db.yeastgenome.org/cgi-bin/locus.pl?locus=BUD32)*,* [*BUD6*](http://db.yeastgenome.org/cgi-bin/locus.pl?locus=BUD6)*,* [*CBF5*](http://db.yeastgenome.org/cgi-bin/locus.pl?locus=CBF5)*,* [*CDC20*](http://db.yeastgenome.org/cgi-bin/locus.pl?locus=CDC20)*,* [*CGR1*](http://db.yeastgenome.org/cgi-bin/locus.pl?locus=CGR1)*,* [*CIC1*](http://db.yeastgenome.org/cgi-bin/locus.pl?locus=CIC1)*,* [*CIN4*](http://db.yeastgenome.org/cgi-bin/locus.pl?locus=CIN4)*,* [*CLB1*](http://db.yeastgenome.org/cgi-bin/locus.pl?locus=CLB1)*, CMS1,* [*CSM3*](http://db.yeastgenome.org/cgi-bin/locus.pl?locus=CSM3)*,* [*DBP10*](http://db.yeastgenome.org/cgi-bin/locus.pl?locus=DBP10)*,* [*DBP2*](http://db.yeastgenome.org/cgi-bin/locus.pl?locus=DBP2)*,* [*DBP3*](http://db.yeastgenome.org/cgi-bin/locus.pl?locus=DBP3)*,* [*DBP6*](http://db.yeastgenome.org/cgi-bin/locus.pl?locus=DBP6)*,* [*DBP7*](http://db.yeastgenome.org/cgi-bin/locus.pl?locus=DBP7)*,* [*DBP8*](http://db.yeastgenome.org/cgi-bin/locus.pl?locus=DBP8)*,* [*DBP9*](http://db.yeastgenome.org/cgi-bin/locus.pl?locus=DBP9)*,* [*DHR2*](http://db.yeastgenome.org/cgi-bin/locus.pl?locus=DHR2)*,* [*DIM1*](http://db.yeastgenome.org/cgi-bin/locus.pl?locus=DIM1)*,* [*DIP2*](http://db.yeastgenome.org/cgi-bin/locus.pl?locus=DIP2)*,* [*DPH2*](http://db.yeastgenome.org/cgi-bin/locus.pl?locus=DPH2)*,* [*DRS1*](http://db.yeastgenome.org/cgi-bin/locus.pl?locus=DRS1)*,* [*DUO1*](http://db.yeastgenome.org/cgi-bin/locus.pl?locus=DUO1)*,* [*DUS3*](http://db.yeastgenome.org/cgi-bin/locus.pl?locus=DUS3)*,* [*EBP2*](http://db.yeastgenome.org/cgi-bin/locus.pl?locus=EBP2)*,* [*ECM16*](http://db.yeastgenome.org/cgi-bin/locus.pl?locus=ECM16)*, EFG1,* [*EMG1*](http://db.yeastgenome.org/cgi-bin/locus.pl?locus=EMG1)*,* [*ENP1*](http://db.yeastgenome.org/cgi-bin/locus.pl?locus=ENP1)*,* [*ENP2*](http://db.yeastgenome.org/cgi-bin/locus.pl?locus=ENP2)*,* [*ERB1*](http://db.yeastgenome.org/cgi-bin/locus.pl?locus=ERB1)*,* [*ESF1*](http://db.yeastgenome.org/cgi-bin/locus.pl?locus=ESF1)*,* [*ESF2*](http://db.yeastgenome.org/cgi-bin/locus.pl?locus=ESF2)*,* [*ESP1*](http://db.yeastgenome.org/cgi-bin/locus.pl?locus=ESP1)*,* [*FAF1*](http://db.yeastgenome.org/cgi-bin/locus.pl?locus=FAF1)*,* [*FAL1*](http://db.yeastgenome.org/cgi-bin/locus.pl?locus=FAL1)*,* [*FAP7*](http://db.yeastgenome.org/cgi-bin/locus.pl?locus=FAP7)*,* [*FCF2*](http://db.yeastgenome.org/cgi-bin/locus.pl?locus=FCF2)*,* [*FKH1*](http://db.yeastgenome.org/cgi-bin/locus.pl?locus=FKH1)*,* [*FPR4*](http://db.yeastgenome.org/cgi-bin/locus.pl?locus=FPR4)*,* [*FYV7*](http://db.yeastgenome.org/cgi-bin/locus.pl?locus=FYV7)*,* [*GAR1*](http://db.yeastgenome.org/cgi-bin/locus.pl?locus=GAR1)*,* [*GCD10*](http://db.yeastgenome.org/cgi-bin/locus.pl?locus=GCD10)*,* [*GCD14*](http://db.yeastgenome.org/cgi-bin/locus.pl?locus=GCD14)*,* [*GDT1*](http://db.yeastgenome.org/cgi-bin/locus.pl?locus=GDT1)*,* [*GEA1*](http://db.yeastgenome.org/cgi-bin/locus.pl?locus=GEA1)*,* [*GLE2*](http://db.yeastgenome.org/cgi-bin/locus.pl?locus=GLE2)*,* [*GRC3*](http://db.yeastgenome.org/cgi-bin/locus.pl?locus=GRC3)*,* [*HAS1*](http://db.yeastgenome.org/cgi-bin/locus.pl?locus=HAS1)*,* [*HCA4*](http://db.yeastgenome.org/cgi-bin/locus.pl?locus=HCA4)*,* [*HGH1*](http://db.yeastgenome.org/cgi-bin/locus.pl?locus=HGH1)*,* [*HHF2*](http://db.yeastgenome.org/cgi-bin/locus.pl?locus=HHF2)*,* [*HHT1*](http://db.yeastgenome.org/cgi-bin/locus.pl?locus=HHT1)*,* [*HIR2*](http://db.yeastgenome.org/cgi-bin/locus.pl?locus=HIR2)*,* [*HMT1*](http://db.yeastgenome.org/cgi-bin/locus.pl?locus=HMT1)*,* [*HST3*](http://db.yeastgenome.org/cgi-bin/locus.pl?locus=HST3)*,* [*HTA1*](http://db.yeastgenome.org/cgi-bin/locus.pl?locus=HTA1)*,* [*HTA2*](http://db.yeastgenome.org/cgi-bin/locus.pl?locus=HTA2)*,* [*HTB1*](http://db.yeastgenome.org/cgi-bin/locus.pl?locus=HTB1)*,* [*HTB2*](http://db.yeastgenome.org/cgi-bin/locus.pl?locus=HTB2)*,* [*HTZ1*](http://db.yeastgenome.org/cgi-bin/locus.pl?locus=HTZ1)*,* [*IFH1*](http://db.yeastgenome.org/cgi-bin/locus.pl?locus=IFH1)*,* [*ILV5*](http://db.yeastgenome.org/cgi-bin/locus.pl?locus=ILV5)*,* [*IMP3*](http://db.yeastgenome.org/cgi-bin/locus.pl?locus=IMP3)*,* [*IPI1*](http://db.yeastgenome.org/cgi-bin/locus.pl?locus=IPI1)*,* [*IPI3*](http://db.yeastgenome.org/cgi-bin/locus.pl?locus=IPI3)*,* [*KAE1*](http://db.yeastgenome.org/cgi-bin/locus.pl?locus=KAE1)*,* [*KCS1*](http://db.yeastgenome.org/cgi-bin/locus.pl?locus=KCS1)*,* [*KRE33*](http://db.yeastgenome.org/cgi-bin/locus.pl?locus=KRE33)*,* [*KRI1*](http://db.yeastgenome.org/cgi-bin/locus.pl?locus=KRI1)*,* [*KRR1*](http://db.yeastgenome.org/cgi-bin/locus.pl?locus=KRR1)*,* [*LCP5*](http://db.yeastgenome.org/cgi-bin/locus.pl?locus=LCP5)*,* [*LIA1*](http://db.yeastgenome.org/cgi-bin/locus.pl?locus=LIA1)*,* [*LOC1*](http://db.yeastgenome.org/cgi-bin/locus.pl?locus=LOC1)*,* [*LSG1*](http://db.yeastgenome.org/cgi-bin/locus.pl?locus=LSG1)*,* [*MAK11*](http://db.yeastgenome.org/cgi-bin/locus.pl?locus=MAK11)*,* [*MAK16*](http://db.yeastgenome.org/cgi-bin/locus.pl?locus=MAK16)*,* [*MAK21*](http://db.yeastgenome.org/cgi-bin/locus.pl?locus=MAK21)*,* [*MAK3*](http://db.yeastgenome.org/cgi-bin/locus.pl?locus=MAK3)*,* [*MIS1*](http://db.yeastgenome.org/cgi-bin/locus.pl?locus=MIS1)*,* [*MOT1*](http://db.yeastgenome.org/cgi-bin/locus.pl?locus=MOT1)*,* [*MPP10*](http://db.yeastgenome.org/cgi-bin/locus.pl?locus=MPP10)*,* [*MRD1*](http://db.yeastgenome.org/cgi-bin/locus.pl?locus=MRD1)*,* [*MRT4*](http://db.yeastgenome.org/cgi-bin/locus.pl?locus=MRT4)*,* [*MSH1*](http://db.yeastgenome.org/cgi-bin/locus.pl?locus=MSH1)*,* [*MTR3*](http://db.yeastgenome.org/cgi-bin/locus.pl?locus=MTR3)*,* [*NAN1*](http://db.yeastgenome.org/cgi-bin/locus.pl?locus=NAN1)*,* [*NCL1*](http://db.yeastgenome.org/cgi-bin/locus.pl?locus=NCL1)*,* [*NCS2*](http://db.yeastgenome.org/cgi-bin/locus.pl?locus=NCS2)*,* [*NEW1*](http://db.yeastgenome.org/cgi-bin/locus.pl?locus=NEW1)*,* [*NHP2*](http://db.yeastgenome.org/cgi-bin/locus.pl?locus=NHP2)*,* [*NIP7*](http://db.yeastgenome.org/cgi-bin/locus.pl?locus=NIP7)*,* [*NMD3*](http://db.yeastgenome.org/cgi-bin/locus.pl?locus=NMD3)*,* [*NOB1*](http://db.yeastgenome.org/cgi-bin/locus.pl?locus=NOB1)*,* [*NOC2*](http://db.yeastgenome.org/cgi-bin/locus.pl?locus=NOC2)*,* [*NOC3*](http://db.yeastgenome.org/cgi-bin/locus.pl?locus=NOC3)*,* [*NOC4*](http://db.yeastgenome.org/cgi-bin/locus.pl?locus=NOC4)*,* [*NOG1*](http://db.yeastgenome.org/cgi-bin/locus.pl?locus=NOG1)*,* [*NOG2*](http://db.yeastgenome.org/cgi-bin/locus.pl?locus=NOG2)*,* [*NOP13*](http://db.yeastgenome.org/cgi-bin/locus.pl?locus=NOP13)*,* [*NOP14*](http://db.yeastgenome.org/cgi-bin/locus.pl?locus=NOP14)*,* [*NOP15*](http://db.yeastgenome.org/cgi-bin/locus.pl?locus=NOP15)*,* [*NOP16*](http://db.yeastgenome.org/cgi-bin/locus.pl?locus=NOP16)*,* [*NOP2*](http://db.yeastgenome.org/cgi-bin/locus.pl?locus=NOP2)*,* [*NOP4*](http://db.yeastgenome.org/cgi-bin/locus.pl?locus=NOP4)*,* [*NOP53*](http://db.yeastgenome.org/cgi-bin/locus.pl?locus=NOP53)*,* [*NOP58*](http://db.yeastgenome.org/cgi-bin/locus.pl?locus=NOP58)*,* [*NOP6*](http://db.yeastgenome.org/cgi-bin/locus.pl?locus=NOP6)*,* [*NOP7*](http://db.yeastgenome.org/cgi-bin/locus.pl?locus=NOP7)*,* [*NOP8*](http://db.yeastgenome.org/cgi-bin/locus.pl?locus=NOP8)*,* [*NOP9*](http://db.yeastgenome.org/cgi-bin/locus.pl?locus=NOP9)*,* [*NRP1*](http://db.yeastgenome.org/cgi-bin/locus.pl?locus=NRP1)*,* [*NSA1*](http://db.yeastgenome.org/cgi-bin/locus.pl?locus=NSA1)*,* [*NSR1*](http://db.yeastgenome.org/cgi-bin/locus.pl?locus=NSR1)*,* [*NUC1*](http://db.yeastgenome.org/cgi-bin/locus.pl?locus=NUC1)*,* [*NUG1*](http://db.yeastgenome.org/cgi-bin/locus.pl?locus=NUG1)*,* [*NUP1*](http://db.yeastgenome.org/cgi-bin/locus.pl?locus=NUP1)*,* [*ORC1*](http://db.yeastgenome.org/cgi-bin/locus.pl?locus=ORC1)*,* [*PNO1*](http://db.yeastgenome.org/cgi-bin/locus.pl?locus=PNO1)*,* [*POP3*](http://db.yeastgenome.org/cgi-bin/locus.pl?locus=POP3)*,* [*POP6*](http://db.yeastgenome.org/cgi-bin/locus.pl?locus=POP6)*,* [*POP8*](http://db.yeastgenome.org/cgi-bin/locus.pl?locus=POP8)*,* [*PPT1*](http://db.yeastgenome.org/cgi-bin/locus.pl?locus=PPT1)*,* [*PRO1*](http://db.yeastgenome.org/cgi-bin/locus.pl?locus=PRO1)*,* [*PRP43*](http://db.yeastgenome.org/cgi-bin/locus.pl?locus=PRP43)*,* [*PRS4*](http://db.yeastgenome.org/cgi-bin/locus.pl?locus=PRS4)*,* [*PUF6*](http://db.yeastgenome.org/cgi-bin/locus.pl?locus=PUF6)*,* [*PUS1*](http://db.yeastgenome.org/cgi-bin/locus.pl?locus=PUS1)*,* [*PUS7*](http://db.yeastgenome.org/cgi-bin/locus.pl?locus=PUS7)*,* [*PWP1*](http://db.yeastgenome.org/cgi-bin/locus.pl?locus=PWP1)*,* [*PWP2*](http://db.yeastgenome.org/cgi-bin/locus.pl?locus=PWP2)*,* [*PXR1*](http://db.yeastgenome.org/cgi-bin/locus.pl?locus=PXR1)*,* [*RAP1*](http://db.yeastgenome.org/cgi-bin/locus.pl?locus=RAP1)*,* [*RBG1*](http://db.yeastgenome.org/cgi-bin/locus.pl?locus=RBG1)*,* [*RCL1*](http://db.yeastgenome.org/cgi-bin/locus.pl?locus=RCL1)*,* [*REI1*](http://db.yeastgenome.org/cgi-bin/locus.pl?locus=REI1)*,* [*REX4*](http://db.yeastgenome.org/cgi-bin/locus.pl?locus=REX4)*,* [*RIO1*](http://db.yeastgenome.org/cgi-bin/locus.pl?locus=RIO1)*,* [*RIX1*](http://db.yeastgenome.org/cgi-bin/locus.pl?locus=RIX1)*,* [*RIX7*](http://db.yeastgenome.org/cgi-bin/locus.pl?locus=RIX7)*,* [*RKI1*](http://db.yeastgenome.org/cgi-bin/locus.pl?locus=RKI1)*,* [*RLI1*](http://db.yeastgenome.org/cgi-bin/locus.pl?locus=RLI1)*,* [*RLP24*](http://db.yeastgenome.org/cgi-bin/locus.pl?locus=RLP24)*,* [*RLP7*](http://db.yeastgenome.org/cgi-bin/locus.pl?locus=RLP7)*,* [*RMT2*](http://db.yeastgenome.org/cgi-bin/locus.pl?locus=RMT2)*,* [*RNT1*](http://db.yeastgenome.org/cgi-bin/locus.pl?locus=RNT1)*,* [*ROK1*](http://db.yeastgenome.org/cgi-bin/locus.pl?locus=ROK1)*,* [*RPA12*](http://db.yeastgenome.org/cgi-bin/locus.pl?locus=RPA12)*,* [*RPA34*](http://db.yeastgenome.org/cgi-bin/locus.pl?locus=RPA34)*,* [*RPA43*](http://db.yeastgenome.org/cgi-bin/locus.pl?locus=RPA43)*,* [*RPA49*](http://db.yeastgenome.org/cgi-bin/locus.pl?locus=RPA49)*,* [*RPB9*](http://db.yeastgenome.org/cgi-bin/locus.pl?locus=RPB9)*,* [*RPC19*](http://db.yeastgenome.org/cgi-bin/locus.pl?locus=RPC19)*,* [*RPC34*](http://db.yeastgenome.org/cgi-bin/locus.pl?locus=RPC34)*,* [*RPC40*](http://db.yeastgenome.org/cgi-bin/locus.pl?locus=RPC40)*,* [*RPC82*](http://db.yeastgenome.org/cgi-bin/locus.pl?locus=RPC82)*,* [*RPF1*](http://db.yeastgenome.org/cgi-bin/locus.pl?locus=RPF1)*,* [*RPF2*](http://db.yeastgenome.org/cgi-bin/locus.pl?locus=RPF2)*,* [*RPP1*](http://db.yeastgenome.org/cgi-bin/locus.pl?locus=RPP1)*,* [*RPS14B*](http://db.yeastgenome.org/cgi-bin/locus.pl?locus=RPS14B)*,* [*RPS19B*](http://db.yeastgenome.org/cgi-bin/locus.pl?locus=RPS19B)*,* [*RPS21A*](http://db.yeastgenome.org/cgi-bin/locus.pl?locus=RPS21A)*,* [*RRB1*](http://db.yeastgenome.org/cgi-bin/locus.pl?locus=RRB1)*,* [*RRM3*](http://db.yeastgenome.org/cgi-bin/locus.pl?locus=RRM3)*,* [*RRP1*](http://db.yeastgenome.org/cgi-bin/locus.pl?locus=RRP1)*,* [*RRP12*](http://db.yeastgenome.org/cgi-bin/locus.pl?locus=RRP12)*,* [*RRP14*](http://db.yeastgenome.org/cgi-bin/locus.pl?locus=RRP14)*,* [*RRP15*](http://db.yeastgenome.org/cgi-bin/locus.pl?locus=RRP15)*,* [*RRP17*](http://db.yeastgenome.org/cgi-bin/locus.pl?locus=RRP17)*,* [*RRP3*](http://db.yeastgenome.org/cgi-bin/locus.pl?locus=RRP3)*, RRP36,* [*RRP40*](http://db.yeastgenome.org/cgi-bin/locus.pl?locus=RRP40)*,* [*RRP43*](http://db.yeastgenome.org/cgi-bin/locus.pl?locus=RRP43)*,* [*RRP5*](http://db.yeastgenome.org/cgi-bin/locus.pl?locus=RRP5)*,* [*RRP6*](http://db.yeastgenome.org/cgi-bin/locus.pl?locus=RRP6)*,* [*RRP8*](http://db.yeastgenome.org/cgi-bin/locus.pl?locus=RRP8)*,* [*RRP9*](http://db.yeastgenome.org/cgi-bin/locus.pl?locus=RRP9)*,* [*RRS1*](http://db.yeastgenome.org/cgi-bin/locus.pl?locus=RRS1)*, RRT14,* [*RSA1*](http://db.yeastgenome.org/cgi-bin/locus.pl?locus=RSA1)*,* [*RSA3*](http://db.yeastgenome.org/cgi-bin/locus.pl?locus=RSA3)*,* [*RSA4*](http://db.yeastgenome.org/cgi-bin/locus.pl?locus=RSA4)*,* [*RVB1*](http://db.yeastgenome.org/cgi-bin/locus.pl?locus=RVB1)*,* [*SAN1*](http://db.yeastgenome.org/cgi-bin/locus.pl?locus=SAN1)*,* [*SAS10*](http://db.yeastgenome.org/cgi-bin/locus.pl?locus=SAS10)*,* [*SCC2*](http://db.yeastgenome.org/cgi-bin/locus.pl?locus=SCC2)*,* [*SDA1*](http://db.yeastgenome.org/cgi-bin/locus.pl?locus=SDA1)*, SEE1,* [*SGO1*](http://db.yeastgenome.org/cgi-bin/locus.pl?locus=SGO1)*,* [*SIK1*](http://db.yeastgenome.org/cgi-bin/locus.pl?locus=SIK1)*,* [*SIM1*](http://db.yeastgenome.org/cgi-bin/locus.pl?locus=SIM1)*,* [*SIR2*](http://db.yeastgenome.org/cgi-bin/locus.pl?locus=SIR2)*,* [*SLI15*](http://db.yeastgenome.org/cgi-bin/locus.pl?locus=SLI15)*,* [*SLK19*](http://db.yeastgenome.org/cgi-bin/locus.pl?locus=SLK19)*,* [*SLX9*](http://db.yeastgenome.org/cgi-bin/locus.pl?locus=SLX9)*,* [*SMC2*](http://db.yeastgenome.org/cgi-bin/locus.pl?locus=SMC2)*,* [*SMI1*](http://db.yeastgenome.org/cgi-bin/locus.pl?locus=SMI1)*,* [*SMY2*](http://db.yeastgenome.org/cgi-bin/locus.pl?locus=SMY2)*,* [*SNU13*](http://db.yeastgenome.org/cgi-bin/locus.pl?locus=SNU13)*,* [*SOF1*](http://db.yeastgenome.org/cgi-bin/locus.pl?locus=SOF1)*,* [*SPA2*](http://db.yeastgenome.org/cgi-bin/locus.pl?locus=SPA2)*,* [*SPB1*](http://db.yeastgenome.org/cgi-bin/locus.pl?locus=SPB1)*,* [*SPB4*](http://db.yeastgenome.org/cgi-bin/locus.pl?locus=SPB4)*,* [*SPC98*](http://db.yeastgenome.org/cgi-bin/locus.pl?locus=SPC98)*,* [*SQT1*](http://db.yeastgenome.org/cgi-bin/locus.pl?locus=SQT1)*,* [*SRO9*](http://db.yeastgenome.org/cgi-bin/locus.pl?locus=SRO9)*,* [*SRP40*](http://db.yeastgenome.org/cgi-bin/locus.pl?locus=SRP40)*,* [*SSF1*](http://db.yeastgenome.org/cgi-bin/locus.pl?locus=SSF1)*,* [*SSF2*](http://db.yeastgenome.org/cgi-bin/locus.pl?locus=SSF2)*,* [*STU2*](http://db.yeastgenome.org/cgi-bin/locus.pl?locus=STU2)*,* [*SUN4*](http://db.yeastgenome.org/cgi-bin/locus.pl?locus=SUN4)*,* [*SUR4*](http://db.yeastgenome.org/cgi-bin/locus.pl?locus=SUR4)*,* [*TEL2*](http://db.yeastgenome.org/cgi-bin/locus.pl?locus=TEL2)*,* [*THP2*](http://db.yeastgenome.org/cgi-bin/locus.pl?locus=THP2)*,* [*TIF4631*](http://db.yeastgenome.org/cgi-bin/locus.pl?locus=TIF4631)*,* [*TMA23*](http://db.yeastgenome.org/cgi-bin/locus.pl?locus=TMA23)*,* [*TMA46*](http://db.yeastgenome.org/cgi-bin/locus.pl?locus=TMA46)*, TOB6,* [*TOP1*](http://db.yeastgenome.org/cgi-bin/locus.pl?locus=TOP1)*,* [*TPA1*](http://db.yeastgenome.org/cgi-bin/locus.pl?locus=TPA1)*,* [*TRF5*](http://db.yeastgenome.org/cgi-bin/locus.pl?locus=TRF5)*,* [*TRM1*](http://db.yeastgenome.org/cgi-bin/locus.pl?locus=TRM1)*,* [*TRM11*](http://db.yeastgenome.org/cgi-bin/locus.pl?locus=TRM11)*,* [*TRM2*](http://db.yeastgenome.org/cgi-bin/locus.pl?locus=TRM2)*,* [*TRM8*](http://db.yeastgenome.org/cgi-bin/locus.pl?locus=TRM8)*,* [*TRM82*](http://db.yeastgenome.org/cgi-bin/locus.pl?locus=TRM82)*,* [*TSR1*](http://db.yeastgenome.org/cgi-bin/locus.pl?locus=TSR1)*,* [*TSR2*](http://db.yeastgenome.org/cgi-bin/locus.pl?locus=TSR2)*,* [*UBC9*](http://db.yeastgenome.org/cgi-bin/locus.pl?locus=UBC9)*,* [*UBP10*](http://db.yeastgenome.org/cgi-bin/locus.pl?locus=UBP10)*,* [*URB1*](http://db.yeastgenome.org/cgi-bin/locus.pl?locus=URB1)*,* [*URB2*](http://db.yeastgenome.org/cgi-bin/locus.pl?locus=URB2)*,* [*URK1*](http://db.yeastgenome.org/cgi-bin/locus.pl?locus=URK1)*,* [*UTP10*](http://db.yeastgenome.org/cgi-bin/locus.pl?locus=UTP10)*,* [*UTP11*](http://db.yeastgenome.org/cgi-bin/locus.pl?locus=UTP11)*,* [*UTP13*](http://db.yeastgenome.org/cgi-bin/locus.pl?locus=UTP13)*,* [*UTP14*](http://db.yeastgenome.org/cgi-bin/locus.pl?locus=UTP14)*,* [*UTP15*](http://db.yeastgenome.org/cgi-bin/locus.pl?locus=UTP15)*,* [*UTP18*](http://db.yeastgenome.org/cgi-bin/locus.pl?locus=UTP18)*,* [*UTP21*](http://db.yeastgenome.org/cgi-bin/locus.pl?locus=UTP21)*,* [*UTP22*](http://db.yeastgenome.org/cgi-bin/locus.pl?locus=UTP22)*,* [*UTP23*](http://db.yeastgenome.org/cgi-bin/locus.pl?locus=UTP23)*,* [*UTP30*](http://db.yeastgenome.org/cgi-bin/locus.pl?locus=UTP30)*,* [*UTP4*](http://db.yeastgenome.org/cgi-bin/locus.pl?locus=UTP4)*,* [*UTP5*](http://db.yeastgenome.org/cgi-bin/locus.pl?locus=UTP5)*,* [*UTP7*](http://db.yeastgenome.org/cgi-bin/locus.pl?locus=UTP7)*,* [*UTP8*](http://db.yeastgenome.org/cgi-bin/locus.pl?locus=UTP8)*,* [*UTP9*](http://db.yeastgenome.org/cgi-bin/locus.pl?locus=UTP9)*,* [*VTC1*](http://db.yeastgenome.org/cgi-bin/locus.pl?locus=VTC1)*,* [*VTC3*](http://db.yeastgenome.org/cgi-bin/locus.pl?locus=VTC3)*,* [*YAR1*](http://db.yeastgenome.org/cgi-bin/locus.pl?locus=YAR1)*,* [*YBL028C*](http://db.yeastgenome.org/cgi-bin/locus.pl?locus=YBL028C)*,* [*YBR238C*](http://db.yeastgenome.org/cgi-bin/locus.pl?locus=YBR238C)*,* [*YCG1*](http://db.yeastgenome.org/cgi-bin/locus.pl?locus=YCG1)*,* [*YCR016W*](http://db.yeastgenome.org/cgi-bin/locus.pl?locus=YCR016W)*,* [*YDL063C*](http://db.yeastgenome.org/cgi-bin/locus.pl?locus=YDL063C)*,* [*YEF3*](http://db.yeastgenome.org/cgi-bin/locus.pl?locus=YEF3)*,* [*YGR283C*](http://db.yeastgenome.org/cgi-bin/locus.pl?locus=YGR283C)*,* [*YHL039W*](http://db.yeastgenome.org/cgi-bin/locus.pl?locus=YHL039W)*,* [*YHM2*](http://db.yeastgenome.org/cgi-bin/locus.pl?locus=YHM2)*,* [*YIL096C*](http://db.yeastgenome.org/cgi-bin/locus.pl?locus=YIL096C)*,* [*YMR310C*](http://db.yeastgenome.org/cgi-bin/locus.pl?locus=YMR310C)*,* [*YNL022C*](http://db.yeastgenome.org/cgi-bin/locus.pl?locus=YNL022C)*,* [*YNL247W*](http://db.yeastgenome.org/cgi-bin/locus.pl?locus=YNL247W)*,* [*YNL313C*](http://db.yeastgenome.org/cgi-bin/locus.pl?locus=YNL313C)*,* [*YOR021C*](http://db.yeastgenome.org/cgi-bin/locus.pl?locus=YOR021C)*,* [*YRF1-6*](http://db.yeastgenome.org/cgi-bin/locus.pl?locus=YRF1-6)*,* [*YTM1*](http://db.yeastgenome.org/cgi-bin/locus.pl?locus=YTM1)*,* [*YVH1*](http://db.yeastgenome.org/cgi-bin/locus.pl?locus=YVH1) |
| rRNA metabolic process  rRNA metabolic process (cont.) | 124 out of 558 genes, 22.2% | 1.54e-70 | [*AIR1*](http://db.yeastgenome.org/cgi-bin/locus.pl?locus=AIR1)*,* [*BMS1*](http://db.yeastgenome.org/cgi-bin/locus.pl?locus=BMS1)*,* [*BUD21*](http://db.yeastgenome.org/cgi-bin/locus.pl?locus=BUD21)*,* [*CBF5*](http://db.yeastgenome.org/cgi-bin/locus.pl?locus=CBF5)*,* [*CGR1*](http://db.yeastgenome.org/cgi-bin/locus.pl?locus=CGR1)*,* [*DBP10*](http://db.yeastgenome.org/cgi-bin/locus.pl?locus=DBP10)*,* [*DBP2*](http://db.yeastgenome.org/cgi-bin/locus.pl?locus=DBP2)*,* [*DBP3*](http://db.yeastgenome.org/cgi-bin/locus.pl?locus=DBP3)*,* [*DBP6*](http://db.yeastgenome.org/cgi-bin/locus.pl?locus=DBP6)*,* [*DBP7*](http://db.yeastgenome.org/cgi-bin/locus.pl?locus=DBP7)*,* [*DBP8*](http://db.yeastgenome.org/cgi-bin/locus.pl?locus=DBP8)*,* [*DBP9*](http://db.yeastgenome.org/cgi-bin/locus.pl?locus=DBP9)*,* [*DIM1*](http://db.yeastgenome.org/cgi-bin/locus.pl?locus=DIM1)*,* [*DIP2*](http://db.yeastgenome.org/cgi-bin/locus.pl?locus=DIP2)*,* [*DRS1*](http://db.yeastgenome.org/cgi-bin/locus.pl?locus=DRS1)*,* [*EBP2*](http://db.yeastgenome.org/cgi-bin/locus.pl?locus=EBP2)*,* [*ECM16*](http://db.yeastgenome.org/cgi-bin/locus.pl?locus=ECM16)*, EFG1,* [*EMG1*](http://db.yeastgenome.org/cgi-bin/locus.pl?locus=EMG1)*,* [*ENP1*](http://db.yeastgenome.org/cgi-bin/locus.pl?locus=ENP1)*,* [*ENP2*](http://db.yeastgenome.org/cgi-bin/locus.pl?locus=ENP2)*,* [*ERB1*](http://db.yeastgenome.org/cgi-bin/locus.pl?locus=ERB1)*,* [*ESF1*](http://db.yeastgenome.org/cgi-bin/locus.pl?locus=ESF1)*,* [*ESF2*](http://db.yeastgenome.org/cgi-bin/locus.pl?locus=ESF2)*,* [*FAF1*](http://db.yeastgenome.org/cgi-bin/locus.pl?locus=FAF1)*,* [*FAL1*](http://db.yeastgenome.org/cgi-bin/locus.pl?locus=FAL1)*,* [*FAP7*](http://db.yeastgenome.org/cgi-bin/locus.pl?locus=FAP7)*,* [*FCF2*](http://db.yeastgenome.org/cgi-bin/locus.pl?locus=FCF2)*,* [*FYV7*](http://db.yeastgenome.org/cgi-bin/locus.pl?locus=FYV7)*,* [*GAR1*](http://db.yeastgenome.org/cgi-bin/locus.pl?locus=GAR1)*,* [*GRC3*](http://db.yeastgenome.org/cgi-bin/locus.pl?locus=GRC3)*,* [*HAS1*](http://db.yeastgenome.org/cgi-bin/locus.pl?locus=HAS1)*,* [*HCA4*](http://db.yeastgenome.org/cgi-bin/locus.pl?locus=HCA4)*,* [*IMP3*](http://db.yeastgenome.org/cgi-bin/locus.pl?locus=IMP3)*,* [*IPI1*](http://db.yeastgenome.org/cgi-bin/locus.pl?locus=IPI1)*,* [*IPI3*](http://db.yeastgenome.org/cgi-bin/locus.pl?locus=IPI3)*,* [*KRR1*](http://db.yeastgenome.org/cgi-bin/locus.pl?locus=KRR1)*,* [*LCP5*](http://db.yeastgenome.org/cgi-bin/locus.pl?locus=LCP5)*,* [*MAK11*](http://db.yeastgenome.org/cgi-bin/locus.pl?locus=MAK11)*,* [*MAK16*](http://db.yeastgenome.org/cgi-bin/locus.pl?locus=MAK16)*,* [*MOT1*](http://db.yeastgenome.org/cgi-bin/locus.pl?locus=MOT1)*,* [*MPP10*](http://db.yeastgenome.org/cgi-bin/locus.pl?locus=MPP10)*,* [*MRD1*](http://db.yeastgenome.org/cgi-bin/locus.pl?locus=MRD1)*,* [*MRT4*](http://db.yeastgenome.org/cgi-bin/locus.pl?locus=MRT4)*,* [*MTR3*](http://db.yeastgenome.org/cgi-bin/locus.pl?locus=MTR3)*,* [*NAN1*](http://db.yeastgenome.org/cgi-bin/locus.pl?locus=NAN1)*,* [*NHP2*](http://db.yeastgenome.org/cgi-bin/locus.pl?locus=NHP2)*,* [*NIP7*](http://db.yeastgenome.org/cgi-bin/locus.pl?locus=NIP7)*,* [*NOB1*](http://db.yeastgenome.org/cgi-bin/locus.pl?locus=NOB1)*,* [*NOC3*](http://db.yeastgenome.org/cgi-bin/locus.pl?locus=NOC3)*,* [*NOC4*](http://db.yeastgenome.org/cgi-bin/locus.pl?locus=NOC4)*,* [*NOG1*](http://db.yeastgenome.org/cgi-bin/locus.pl?locus=NOG1)*,* [*NOP12*](http://db.yeastgenome.org/cgi-bin/locus.pl?locus=NOP12)*,* [*NOP14*](http://db.yeastgenome.org/cgi-bin/locus.pl?locus=NOP14)*,* [*NOP2*](http://db.yeastgenome.org/cgi-bin/locus.pl?locus=NOP2)*,* [*NOP4*](http://db.yeastgenome.org/cgi-bin/locus.pl?locus=NOP4)*,* [*NOP53*](http://db.yeastgenome.org/cgi-bin/locus.pl?locus=NOP53)*,* [*NOP58*](http://db.yeastgenome.org/cgi-bin/locus.pl?locus=NOP58)*,* [*NOP6*](http://db.yeastgenome.org/cgi-bin/locus.pl?locus=NOP6)*,* [*NOP7*](http://db.yeastgenome.org/cgi-bin/locus.pl?locus=NOP7)*,* [*NOP8*](http://db.yeastgenome.org/cgi-bin/locus.pl?locus=NOP8)*,* [*NOP9*](http://db.yeastgenome.org/cgi-bin/locus.pl?locus=NOP9)*,* [*NSR1*](http://db.yeastgenome.org/cgi-bin/locus.pl?locus=NSR1)*,* [*NUG1*](http://db.yeastgenome.org/cgi-bin/locus.pl?locus=NUG1)*,* [*PNO1*](http://db.yeastgenome.org/cgi-bin/locus.pl?locus=PNO1)*,* [*POP3*](http://db.yeastgenome.org/cgi-bin/locus.pl?locus=POP3)*,* [*POP6*](http://db.yeastgenome.org/cgi-bin/locus.pl?locus=POP6)*,* [*POP8*](http://db.yeastgenome.org/cgi-bin/locus.pl?locus=POP8)*,* [*PRP43*](http://db.yeastgenome.org/cgi-bin/locus.pl?locus=PRP43)*,* [*PWP1*](http://db.yeastgenome.org/cgi-bin/locus.pl?locus=PWP1)*,* [*PWP2*](http://db.yeastgenome.org/cgi-bin/locus.pl?locus=PWP2)*,* [*PXR1*](http://db.yeastgenome.org/cgi-bin/locus.pl?locus=PXR1)*,* [*RCL1*](http://db.yeastgenome.org/cgi-bin/locus.pl?locus=RCL1)*,* [*REX4*](http://db.yeastgenome.org/cgi-bin/locus.pl?locus=REX4)*,* [*RIO1*](http://db.yeastgenome.org/cgi-bin/locus.pl?locus=RIO1)*,* [*RIX1*](http://db.yeastgenome.org/cgi-bin/locus.pl?locus=RIX1)*,* [*RLP7*](http://db.yeastgenome.org/cgi-bin/locus.pl?locus=RLP7)*,* [*RNT1*](http://db.yeastgenome.org/cgi-bin/locus.pl?locus=RNT1)*,* [*ROK1*](http://db.yeastgenome.org/cgi-bin/locus.pl?locus=ROK1)*,* [*RPF1*](http://db.yeastgenome.org/cgi-bin/locus.pl?locus=RPF1)*,* [*RPF2*](http://db.yeastgenome.org/cgi-bin/locus.pl?locus=RPF2)*,* [*RPP1*](http://db.yeastgenome.org/cgi-bin/locus.pl?locus=RPP1)*,* [*RPS14B*](http://db.yeastgenome.org/cgi-bin/locus.pl?locus=RPS14B)*,* [*RPS19B*](http://db.yeastgenome.org/cgi-bin/locus.pl?locus=RPS19B)*,* [*RRP1*](http://db.yeastgenome.org/cgi-bin/locus.pl?locus=RRP1)*,* [*RRP12*](http://db.yeastgenome.org/cgi-bin/locus.pl?locus=RRP12)*,* [*RRP15*](http://db.yeastgenome.org/cgi-bin/locus.pl?locus=RRP15)*,* [*RRP17*](http://db.yeastgenome.org/cgi-bin/locus.pl?locus=RRP17)*,* [*RRP3*](http://db.yeastgenome.org/cgi-bin/locus.pl?locus=RRP3)*, RRP36,* [*RRP40*](http://db.yeastgenome.org/cgi-bin/locus.pl?locus=RRP40)*,* [*RRP43*](http://db.yeastgenome.org/cgi-bin/locus.pl?locus=RRP43)*,* [*RRP5*](http://db.yeastgenome.org/cgi-bin/locus.pl?locus=RRP5)*,* [*RRP6*](http://db.yeastgenome.org/cgi-bin/locus.pl?locus=RRP6)*,* [*RRP8*](http://db.yeastgenome.org/cgi-bin/locus.pl?locus=RRP8)*,* [*RRP9*](http://db.yeastgenome.org/cgi-bin/locus.pl?locus=RRP9)*,* [*RRS1*](http://db.yeastgenome.org/cgi-bin/locus.pl?locus=RRS1)*,* [*SAS10*](http://db.yeastgenome.org/cgi-bin/locus.pl?locus=SAS10)*,* [*SIK1*](http://db.yeastgenome.org/cgi-bin/locus.pl?locus=SIK1)*,* [*SLX9*](http://db.yeastgenome.org/cgi-bin/locus.pl?locus=SLX9)*,* [*SNU13*](http://db.yeastgenome.org/cgi-bin/locus.pl?locus=SNU13)*,* [*SOF1*](http://db.yeastgenome.org/cgi-bin/locus.pl?locus=SOF1)*,* [*SPB1*](http://db.yeastgenome.org/cgi-bin/locus.pl?locus=SPB1)*,* [*SPB4*](http://db.yeastgenome.org/cgi-bin/locus.pl?locus=SPB4)*,* [*TRF5*](http://db.yeastgenome.org/cgi-bin/locus.pl?locus=TRF5)*,* [*TSR1*](http://db.yeastgenome.org/cgi-bin/locus.pl?locus=TSR1)*,* [*TSR2*](http://db.yeastgenome.org/cgi-bin/locus.pl?locus=TSR2)*,* [*URB1*](http://db.yeastgenome.org/cgi-bin/locus.pl?locus=URB1)*,* [*URB2*](http://db.yeastgenome.org/cgi-bin/locus.pl?locus=URB2)*,* [*UTP10*](http://db.yeastgenome.org/cgi-bin/locus.pl?locus=UTP10)*,* [*UTP11*](http://db.yeastgenome.org/cgi-bin/locus.pl?locus=UTP11)*,* [*UTP13*](http://db.yeastgenome.org/cgi-bin/locus.pl?locus=UTP13)*,* [*UTP14*](http://db.yeastgenome.org/cgi-bin/locus.pl?locus=UTP14)*,* [*UTP15*](http://db.yeastgenome.org/cgi-bin/locus.pl?locus=UTP15)*,* [*UTP18*](http://db.yeastgenome.org/cgi-bin/locus.pl?locus=UTP18)*,* [*UTP21*](http://db.yeastgenome.org/cgi-bin/locus.pl?locus=UTP21)*,* [*UTP22*](http://db.yeastgenome.org/cgi-bin/locus.pl?locus=UTP22)*,* [*UTP23*](http://db.yeastgenome.org/cgi-bin/locus.pl?locus=UTP23)*,* [*UTP30*](http://db.yeastgenome.org/cgi-bin/locus.pl?locus=UTP30)*,* [*UTP4*](http://db.yeastgenome.org/cgi-bin/locus.pl?locus=UTP4)*,* [*UTP5*](http://db.yeastgenome.org/cgi-bin/locus.pl?locus=UTP5)*,* [*UTP7*](http://db.yeastgenome.org/cgi-bin/locus.pl?locus=UTP7)*,* [*UTP8*](http://db.yeastgenome.org/cgi-bin/locus.pl?locus=UTP8)*,* [*UTP9*](http://db.yeastgenome.org/cgi-bin/locus.pl?locus=UTP9) |
| RNA processing | 127 out of 558 genes, 22.8% | 1.01e-50 | [*AIR1*](http://db.yeastgenome.org/cgi-bin/locus.pl?locus=AIR1)*,* [*BMS1*](http://db.yeastgenome.org/cgi-bin/locus.pl?locus=BMS1)*,* [*BUD21*](http://db.yeastgenome.org/cgi-bin/locus.pl?locus=BUD21)*,* [*CBF5*](http://db.yeastgenome.org/cgi-bin/locus.pl?locus=CBF5)*,* [*CGR1*](http://db.yeastgenome.org/cgi-bin/locus.pl?locus=CGR1)*,* [*CUS1*](http://db.yeastgenome.org/cgi-bin/locus.pl?locus=CUS1)*,* [*CWC2*](http://db.yeastgenome.org/cgi-bin/locus.pl?locus=CWC2)*,* [*DBP10*](http://db.yeastgenome.org/cgi-bin/locus.pl?locus=DBP10)*,* [*DBP2*](http://db.yeastgenome.org/cgi-bin/locus.pl?locus=DBP2)*,* [*DBP3*](http://db.yeastgenome.org/cgi-bin/locus.pl?locus=DBP3)*,* [*DBP6*](http://db.yeastgenome.org/cgi-bin/locus.pl?locus=DBP6)*,* [*DBP7*](http://db.yeastgenome.org/cgi-bin/locus.pl?locus=DBP7)*,* [*DBP8*](http://db.yeastgenome.org/cgi-bin/locus.pl?locus=DBP8)*,* [*DBP9*](http://db.yeastgenome.org/cgi-bin/locus.pl?locus=DBP9)*,* [*DIM1*](http://db.yeastgenome.org/cgi-bin/locus.pl?locus=DIM1)*,* [*DIP2*](http://db.yeastgenome.org/cgi-bin/locus.pl?locus=DIP2)*,* [*DRS1*](http://db.yeastgenome.org/cgi-bin/locus.pl?locus=DRS1)*,* [*EBP2*](http://db.yeastgenome.org/cgi-bin/locus.pl?locus=EBP2)*,* [*ECM16*](http://db.yeastgenome.org/cgi-bin/locus.pl?locus=ECM16)*, EFG1,* [*EMG1*](http://db.yeastgenome.org/cgi-bin/locus.pl?locus=EMG1)*,* [*ENP1*](http://db.yeastgenome.org/cgi-bin/locus.pl?locus=ENP1)*,* [*ENP2*](http://db.yeastgenome.org/cgi-bin/locus.pl?locus=ENP2)*,* [*ERB1*](http://db.yeastgenome.org/cgi-bin/locus.pl?locus=ERB1)*,* [*ESF1*](http://db.yeastgenome.org/cgi-bin/locus.pl?locus=ESF1)*,* [*ESF2*](http://db.yeastgenome.org/cgi-bin/locus.pl?locus=ESF2)*,* [*FAF1*](http://db.yeastgenome.org/cgi-bin/locus.pl?locus=FAF1)*,* [*FAL1*](http://db.yeastgenome.org/cgi-bin/locus.pl?locus=FAL1)*,* [*FAP7*](http://db.yeastgenome.org/cgi-bin/locus.pl?locus=FAP7)*,* [*FCF2*](http://db.yeastgenome.org/cgi-bin/locus.pl?locus=FCF2)*,* [*FYV7*](http://db.yeastgenome.org/cgi-bin/locus.pl?locus=FYV7)*,* [*GAR1*](http://db.yeastgenome.org/cgi-bin/locus.pl?locus=GAR1)*,* [*GRC3*](http://db.yeastgenome.org/cgi-bin/locus.pl?locus=GRC3)*,* [*HAS1*](http://db.yeastgenome.org/cgi-bin/locus.pl?locus=HAS1)*,* [*HCA4*](http://db.yeastgenome.org/cgi-bin/locus.pl?locus=HCA4)*,* [*IMP3*](http://db.yeastgenome.org/cgi-bin/locus.pl?locus=IMP3)*,* [*IPI1*](http://db.yeastgenome.org/cgi-bin/locus.pl?locus=IPI1)*,* [*IPI3*](http://db.yeastgenome.org/cgi-bin/locus.pl?locus=IPI3)*,* [*KRR1*](http://db.yeastgenome.org/cgi-bin/locus.pl?locus=KRR1)*,* [*LCP5*](http://db.yeastgenome.org/cgi-bin/locus.pl?locus=LCP5)*,* [*MAK11*](http://db.yeastgenome.org/cgi-bin/locus.pl?locus=MAK11)*,* [*MAK16*](http://db.yeastgenome.org/cgi-bin/locus.pl?locus=MAK16)*,* [*MOT1*](http://db.yeastgenome.org/cgi-bin/locus.pl?locus=MOT1)*,* [*MPP10*](http://db.yeastgenome.org/cgi-bin/locus.pl?locus=MPP10)*,* [*MRD1*](http://db.yeastgenome.org/cgi-bin/locus.pl?locus=MRD1)*,* [*MRT4*](http://db.yeastgenome.org/cgi-bin/locus.pl?locus=MRT4)*,* [*MTR3*](http://db.yeastgenome.org/cgi-bin/locus.pl?locus=MTR3)*,* [*NAN1*](http://db.yeastgenome.org/cgi-bin/locus.pl?locus=NAN1)*,* [*NHP2*](http://db.yeastgenome.org/cgi-bin/locus.pl?locus=NHP2)*,* [*NIP7*](http://db.yeastgenome.org/cgi-bin/locus.pl?locus=NIP7)*,* [*NOB1*](http://db.yeastgenome.org/cgi-bin/locus.pl?locus=NOB1)*,* [*NOC3*](http://db.yeastgenome.org/cgi-bin/locus.pl?locus=NOC3)*,* [*NOC4*](http://db.yeastgenome.org/cgi-bin/locus.pl?locus=NOC4)*,* [*NOG1*](http://db.yeastgenome.org/cgi-bin/locus.pl?locus=NOG1)*,* [*NOP14*](http://db.yeastgenome.org/cgi-bin/locus.pl?locus=NOP14)*,* [*NOP2*](http://db.yeastgenome.org/cgi-bin/locus.pl?locus=NOP2)*,* [*NOP4*](http://db.yeastgenome.org/cgi-bin/locus.pl?locus=NOP4)*,* [*NOP53*](http://db.yeastgenome.org/cgi-bin/locus.pl?locus=NOP53)*,* [*NOP58*](http://db.yeastgenome.org/cgi-bin/locus.pl?locus=NOP58)*,* [*NOP6*](http://db.yeastgenome.org/cgi-bin/locus.pl?locus=NOP6)*,* [*NOP7*](http://db.yeastgenome.org/cgi-bin/locus.pl?locus=NOP7)*,* [*NOP8*](http://db.yeastgenome.org/cgi-bin/locus.pl?locus=NOP8)*,* [*NOP9*](http://db.yeastgenome.org/cgi-bin/locus.pl?locus=NOP9)*,* [*NSR1*](http://db.yeastgenome.org/cgi-bin/locus.pl?locus=NSR1)*,* [*NUG1*](http://db.yeastgenome.org/cgi-bin/locus.pl?locus=NUG1)*,* [*PNO1*](http://db.yeastgenome.org/cgi-bin/locus.pl?locus=PNO1)*,* [*POP3*](http://db.yeastgenome.org/cgi-bin/locus.pl?locus=POP3)*,* [*POP6*](http://db.yeastgenome.org/cgi-bin/locus.pl?locus=POP6)*,* [*POP8*](http://db.yeastgenome.org/cgi-bin/locus.pl?locus=POP8)*,* [*PRP19*](http://db.yeastgenome.org/cgi-bin/locus.pl?locus=PRP19)*,* [*PRP24*](http://db.yeastgenome.org/cgi-bin/locus.pl?locus=PRP24)*,* [*PRP43*](http://db.yeastgenome.org/cgi-bin/locus.pl?locus=PRP43)*,* [*PWP1*](http://db.yeastgenome.org/cgi-bin/locus.pl?locus=PWP1)*,* [*PWP2*](http://db.yeastgenome.org/cgi-bin/locus.pl?locus=PWP2)*,* [*PXR1*](http://db.yeastgenome.org/cgi-bin/locus.pl?locus=PXR1)*,* [*RCL1*](http://db.yeastgenome.org/cgi-bin/locus.pl?locus=RCL1)*,* [*REX4*](http://db.yeastgenome.org/cgi-bin/locus.pl?locus=REX4)*,* [*RIO1*](http://db.yeastgenome.org/cgi-bin/locus.pl?locus=RIO1)*,* [*RIX1*](http://db.yeastgenome.org/cgi-bin/locus.pl?locus=RIX1)*,* [*RLP7*](http://db.yeastgenome.org/cgi-bin/locus.pl?locus=RLP7)*,* [*RNT1*](http://db.yeastgenome.org/cgi-bin/locus.pl?locus=RNT1)*,* [*ROK1*](http://db.yeastgenome.org/cgi-bin/locus.pl?locus=ROK1)*,* [*RPF1*](http://db.yeastgenome.org/cgi-bin/locus.pl?locus=RPF1)*,* [*RPF2*](http://db.yeastgenome.org/cgi-bin/locus.pl?locus=RPF2)*,* [*RPP1*](http://db.yeastgenome.org/cgi-bin/locus.pl?locus=RPP1)*,* [*RPS14B*](http://db.yeastgenome.org/cgi-bin/locus.pl?locus=RPS14B)*,* [*RPS19B*](http://db.yeastgenome.org/cgi-bin/locus.pl?locus=RPS19B)*,* [*RRP1*](http://db.yeastgenome.org/cgi-bin/locus.pl?locus=RRP1)*,* [*RRP12*](http://db.yeastgenome.org/cgi-bin/locus.pl?locus=RRP12)*,* [*RRP15*](http://db.yeastgenome.org/cgi-bin/locus.pl?locus=RRP15)*,* [*RRP17*](http://db.yeastgenome.org/cgi-bin/locus.pl?locus=RRP17)*,* [*RRP3*](http://db.yeastgenome.org/cgi-bin/locus.pl?locus=RRP3)*, RRP36,* [*RRP40*](http://db.yeastgenome.org/cgi-bin/locus.pl?locus=RRP40)*,* [*RRP43*](http://db.yeastgenome.org/cgi-bin/locus.pl?locus=RRP43)*,* [*RRP5*](http://db.yeastgenome.org/cgi-bin/locus.pl?locus=RRP5)*,* [*RRP6*](http://db.yeastgenome.org/cgi-bin/locus.pl?locus=RRP6)*,* [*RRP8*](http://db.yeastgenome.org/cgi-bin/locus.pl?locus=RRP8)*,* [*RRP9*](http://db.yeastgenome.org/cgi-bin/locus.pl?locus=RRP9)*,* [*RRS1*](http://db.yeastgenome.org/cgi-bin/locus.pl?locus=RRS1)*,* [*SAS10*](http://db.yeastgenome.org/cgi-bin/locus.pl?locus=SAS10)*,* [*SEN34*](http://db.yeastgenome.org/cgi-bin/locus.pl?locus=SEN34)*,* [*SIK1*](http://db.yeastgenome.org/cgi-bin/locus.pl?locus=SIK1)*,* [*SLX9*](http://db.yeastgenome.org/cgi-bin/locus.pl?locus=SLX9)*,* [*SNU13*](http://db.yeastgenome.org/cgi-bin/locus.pl?locus=SNU13)*,* [*SOF1*](http://db.yeastgenome.org/cgi-bin/locus.pl?locus=SOF1)*,* [*SPB1*](http://db.yeastgenome.org/cgi-bin/locus.pl?locus=SPB1)*,* [*SPB4*](http://db.yeastgenome.org/cgi-bin/locus.pl?locus=SPB4)*,* [*TRF5*](http://db.yeastgenome.org/cgi-bin/locus.pl?locus=TRF5)*,* [*TSR1*](http://db.yeastgenome.org/cgi-bin/locus.pl?locus=TSR1)*,* [*TSR2*](http://db.yeastgenome.org/cgi-bin/locus.pl?locus=TSR2)*,* [*URB1*](http://db.yeastgenome.org/cgi-bin/locus.pl?locus=URB1)*,* [*UTP10*](http://db.yeastgenome.org/cgi-bin/locus.pl?locus=UTP10)*,* [*UTP11*](http://db.yeastgenome.org/cgi-bin/locus.pl?locus=UTP11)*,* [*UTP13*](http://db.yeastgenome.org/cgi-bin/locus.pl?locus=UTP13)*,* [*UTP14*](http://db.yeastgenome.org/cgi-bin/locus.pl?locus=UTP14)*,* [*UTP15*](http://db.yeastgenome.org/cgi-bin/locus.pl?locus=UTP15)*,* [*UTP18*](http://db.yeastgenome.org/cgi-bin/locus.pl?locus=UTP18)*,* [*UTP21*](http://db.yeastgenome.org/cgi-bin/locus.pl?locus=UTP21)*,* [*UTP22*](http://db.yeastgenome.org/cgi-bin/locus.pl?locus=UTP22)*,* [*UTP23*](http://db.yeastgenome.org/cgi-bin/locus.pl?locus=UTP23)*,* [*UTP30*](http://db.yeastgenome.org/cgi-bin/locus.pl?locus=UTP30)*,* [*UTP4*](http://db.yeastgenome.org/cgi-bin/locus.pl?locus=UTP4)*,* [*UTP5*](http://db.yeastgenome.org/cgi-bin/locus.pl?locus=UTP5)*,* [*UTP7*](http://db.yeastgenome.org/cgi-bin/locus.pl?locus=UTP7)*,* [*UTP8*](http://db.yeastgenome.org/cgi-bin/locus.pl?locus=UTP8)*,* [*UTP9*](http://db.yeastgenome.org/cgi-bin/locus.pl?locus=UTP9) |
| RNA metabolic process | 217 out of 558 genes, 38.9% | 8.61e-49 | [*AIR1*](http://db.yeastgenome.org/cgi-bin/locus.pl?locus=AIR1)*,* [*ASH1*](http://db.yeastgenome.org/cgi-bin/locus.pl?locus=ASH1)*,* [*BCD1*](http://db.yeastgenome.org/cgi-bin/locus.pl?locus=BCD1)*,* [*BMS1*](http://db.yeastgenome.org/cgi-bin/locus.pl?locus=BMS1)*,* [*BUD21*](http://db.yeastgenome.org/cgi-bin/locus.pl?locus=BUD21)*,* [*BUD32*](http://db.yeastgenome.org/cgi-bin/locus.pl?locus=BUD32)*,* [*CBF5*](http://db.yeastgenome.org/cgi-bin/locus.pl?locus=CBF5)*,* [*CGR1*](http://db.yeastgenome.org/cgi-bin/locus.pl?locus=CGR1)*,* [*CUS1*](http://db.yeastgenome.org/cgi-bin/locus.pl?locus=CUS1)*,* [*CWC2*](http://db.yeastgenome.org/cgi-bin/locus.pl?locus=CWC2)*,* [*DBP10*](http://db.yeastgenome.org/cgi-bin/locus.pl?locus=DBP10)*,* [*DBP2*](http://db.yeastgenome.org/cgi-bin/locus.pl?locus=DBP2)*,* [*DBP3*](http://db.yeastgenome.org/cgi-bin/locus.pl?locus=DBP3)*,* [*DBP6*](http://db.yeastgenome.org/cgi-bin/locus.pl?locus=DBP6)*,* [*DBP7*](http://db.yeastgenome.org/cgi-bin/locus.pl?locus=DBP7)*,* [*DBP8*](http://db.yeastgenome.org/cgi-bin/locus.pl?locus=DBP8)*,* [*DBP9*](http://db.yeastgenome.org/cgi-bin/locus.pl?locus=DBP9)*,* [*DIM1*](http://db.yeastgenome.org/cgi-bin/locus.pl?locus=DIM1)*,* [*DIP2*](http://db.yeastgenome.org/cgi-bin/locus.pl?locus=DIP2)*,* [*DRS1*](http://db.yeastgenome.org/cgi-bin/locus.pl?locus=DRS1)*,* [*DUS1*](http://db.yeastgenome.org/cgi-bin/locus.pl?locus=DUS1)*,* [*DUS3*](http://db.yeastgenome.org/cgi-bin/locus.pl?locus=DUS3)*,* [*DUS4*](http://db.yeastgenome.org/cgi-bin/locus.pl?locus=DUS4)*,* [*EBP2*](http://db.yeastgenome.org/cgi-bin/locus.pl?locus=EBP2)*,* [*ECM16*](http://db.yeastgenome.org/cgi-bin/locus.pl?locus=ECM16)*, EFG1,* [*ELP2*](http://db.yeastgenome.org/cgi-bin/locus.pl?locus=ELP2)*,* [*ELP3*](http://db.yeastgenome.org/cgi-bin/locus.pl?locus=ELP3)*,* [*EMG1*](http://db.yeastgenome.org/cgi-bin/locus.pl?locus=EMG1)*,* [*ENP1*](http://db.yeastgenome.org/cgi-bin/locus.pl?locus=ENP1)*,* [*ENP2*](http://db.yeastgenome.org/cgi-bin/locus.pl?locus=ENP2)*,* [*ERB1*](http://db.yeastgenome.org/cgi-bin/locus.pl?locus=ERB1)*,* [*ESF1*](http://db.yeastgenome.org/cgi-bin/locus.pl?locus=ESF1)*,* [*ESF2*](http://db.yeastgenome.org/cgi-bin/locus.pl?locus=ESF2)*,* [*FAF1*](http://db.yeastgenome.org/cgi-bin/locus.pl?locus=FAF1)*,* [*FAL1*](http://db.yeastgenome.org/cgi-bin/locus.pl?locus=FAL1)*,* [*FAP7*](http://db.yeastgenome.org/cgi-bin/locus.pl?locus=FAP7)*,* [*FCF2*](http://db.yeastgenome.org/cgi-bin/locus.pl?locus=FCF2)*,* [*FKH1*](http://db.yeastgenome.org/cgi-bin/locus.pl?locus=FKH1)*,* [*FPR4*](http://db.yeastgenome.org/cgi-bin/locus.pl?locus=FPR4)*,* [*FYV7*](http://db.yeastgenome.org/cgi-bin/locus.pl?locus=FYV7)*,* [*GAR1*](http://db.yeastgenome.org/cgi-bin/locus.pl?locus=GAR1)*,* [*GCD10*](http://db.yeastgenome.org/cgi-bin/locus.pl?locus=GCD10)*,* [*GCD14*](http://db.yeastgenome.org/cgi-bin/locus.pl?locus=GCD14)*,* [*GCR1*](http://db.yeastgenome.org/cgi-bin/locus.pl?locus=GCR1)*,* [*GCR2*](http://db.yeastgenome.org/cgi-bin/locus.pl?locus=GCR2)*,* [*GLN4*](http://db.yeastgenome.org/cgi-bin/locus.pl?locus=GLN4)*,* [*GRC3*](http://db.yeastgenome.org/cgi-bin/locus.pl?locus=GRC3)*,* [*HAS1*](http://db.yeastgenome.org/cgi-bin/locus.pl?locus=HAS1)*,* [*HCA4*](http://db.yeastgenome.org/cgi-bin/locus.pl?locus=HCA4)*,* [*HIR2*](http://db.yeastgenome.org/cgi-bin/locus.pl?locus=HIR2)*,* [*HST3*](http://db.yeastgenome.org/cgi-bin/locus.pl?locus=HST3)*,* [*HTZ1*](http://db.yeastgenome.org/cgi-bin/locus.pl?locus=HTZ1)*,* [*IFH1*](http://db.yeastgenome.org/cgi-bin/locus.pl?locus=IFH1)*,* [*IMP3*](http://db.yeastgenome.org/cgi-bin/locus.pl?locus=IMP3)*,* [*INO2*](http://db.yeastgenome.org/cgi-bin/locus.pl?locus=INO2)*,* [*IPI1*](http://db.yeastgenome.org/cgi-bin/locus.pl?locus=IPI1)*,* [*IPI3*](http://db.yeastgenome.org/cgi-bin/locus.pl?locus=IPI3)*,* [*KAE1*](http://db.yeastgenome.org/cgi-bin/locus.pl?locus=KAE1)*,* [*KRR1*](http://db.yeastgenome.org/cgi-bin/locus.pl?locus=KRR1)*,* [*KTI12*](http://db.yeastgenome.org/cgi-bin/locus.pl?locus=KTI12)*,* [*LCP5*](http://db.yeastgenome.org/cgi-bin/locus.pl?locus=LCP5)*,* [*MAK11*](http://db.yeastgenome.org/cgi-bin/locus.pl?locus=MAK11)*,* [*MAK16*](http://db.yeastgenome.org/cgi-bin/locus.pl?locus=MAK16)*,* [*MES1*](http://db.yeastgenome.org/cgi-bin/locus.pl?locus=MES1)*,* [*MIS1*](http://db.yeastgenome.org/cgi-bin/locus.pl?locus=MIS1)*,* [*MOT1*](http://db.yeastgenome.org/cgi-bin/locus.pl?locus=MOT1)*,* [*MPP10*](http://db.yeastgenome.org/cgi-bin/locus.pl?locus=MPP10)*,* [*MRD1*](http://db.yeastgenome.org/cgi-bin/locus.pl?locus=MRD1)*,* [*MRT4*](http://db.yeastgenome.org/cgi-bin/locus.pl?locus=MRT4)*,* [*MTR3*](http://db.yeastgenome.org/cgi-bin/locus.pl?locus=MTR3)*,* [*NAF1*](http://db.yeastgenome.org/cgi-bin/locus.pl?locus=NAF1)*,* [*NAN1*](http://db.yeastgenome.org/cgi-bin/locus.pl?locus=NAN1)*,* [*NCL1*](http://db.yeastgenome.org/cgi-bin/locus.pl?locus=NCL1)*,* [*NHP2*](http://db.yeastgenome.org/cgi-bin/locus.pl?locus=NHP2)*,* [*NIP7*](http://db.yeastgenome.org/cgi-bin/locus.pl?locus=NIP7)*,* [*NOB1*](http://db.yeastgenome.org/cgi-bin/locus.pl?locus=NOB1)*,* [*NOC3*](http://db.yeastgenome.org/cgi-bin/locus.pl?locus=NOC3)*,* [*NOC4*](http://db.yeastgenome.org/cgi-bin/locus.pl?locus=NOC4)*,* [*NOG1*](http://db.yeastgenome.org/cgi-bin/locus.pl?locus=NOG1)*,* [*NOP12*](http://db.yeastgenome.org/cgi-bin/locus.pl?locus=NOP12)*,* [*NOP14*](http://db.yeastgenome.org/cgi-bin/locus.pl?locus=NOP14)*,* [*NOP2*](http://db.yeastgenome.org/cgi-bin/locus.pl?locus=NOP2)*,* [*NOP4*](http://db.yeastgenome.org/cgi-bin/locus.pl?locus=NOP4)*,* [*NOP53*](http://db.yeastgenome.org/cgi-bin/locus.pl?locus=NOP53)*,* [*NOP58*](http://db.yeastgenome.org/cgi-bin/locus.pl?locus=NOP58)*,* [*NOP6*](http://db.yeastgenome.org/cgi-bin/locus.pl?locus=NOP6)*,* [*NOP7*](http://db.yeastgenome.org/cgi-bin/locus.pl?locus=NOP7)*,* [*NOP8*](http://db.yeastgenome.org/cgi-bin/locus.pl?locus=NOP8)*,* [*NOP9*](http://db.yeastgenome.org/cgi-bin/locus.pl?locus=NOP9)*,* [*NSR1*](http://db.yeastgenome.org/cgi-bin/locus.pl?locus=NSR1)*,* [*NUC1*](http://db.yeastgenome.org/cgi-bin/locus.pl?locus=NUC1)*,* [*NUG1*](http://db.yeastgenome.org/cgi-bin/locus.pl?locus=NUG1)*,* [*ORC1*](http://db.yeastgenome.org/cgi-bin/locus.pl?locus=ORC1)*,* [*PNO1*](http://db.yeastgenome.org/cgi-bin/locus.pl?locus=PNO1)*,* [*POL5*](http://db.yeastgenome.org/cgi-bin/locus.pl?locus=POL5)*,* [*POP3*](http://db.yeastgenome.org/cgi-bin/locus.pl?locus=POP3)*,* [*POP6*](http://db.yeastgenome.org/cgi-bin/locus.pl?locus=POP6)*,* [*POP8*](http://db.yeastgenome.org/cgi-bin/locus.pl?locus=POP8)*,* [*PRP19*](http://db.yeastgenome.org/cgi-bin/locus.pl?locus=PRP19)*,* [*PRP24*](http://db.yeastgenome.org/cgi-bin/locus.pl?locus=PRP24)*,* [*PRP43*](http://db.yeastgenome.org/cgi-bin/locus.pl?locus=PRP43)*,* [*PUF6*](http://db.yeastgenome.org/cgi-bin/locus.pl?locus=PUF6)*,* [*PUS1*](http://db.yeastgenome.org/cgi-bin/locus.pl?locus=PUS1)*,* [*PUS4*](http://db.yeastgenome.org/cgi-bin/locus.pl?locus=PUS4)*,* [*PUS7*](http://db.yeastgenome.org/cgi-bin/locus.pl?locus=PUS7)*,* [*PWP1*](http://db.yeastgenome.org/cgi-bin/locus.pl?locus=PWP1)*,* [*PWP2*](http://db.yeastgenome.org/cgi-bin/locus.pl?locus=PWP2)*,* [*PXR1*](http://db.yeastgenome.org/cgi-bin/locus.pl?locus=PXR1)*,* [*RAP1*](http://db.yeastgenome.org/cgi-bin/locus.pl?locus=RAP1)*,* [*RBA50*](http://db.yeastgenome.org/cgi-bin/locus.pl?locus=RBA50)*,* [*RCL1*](http://db.yeastgenome.org/cgi-bin/locus.pl?locus=RCL1)*,* [*RET1*](http://db.yeastgenome.org/cgi-bin/locus.pl?locus=RET1)*,* [*REX4*](http://db.yeastgenome.org/cgi-bin/locus.pl?locus=REX4)*,* [*RIO1*](http://db.yeastgenome.org/cgi-bin/locus.pl?locus=RIO1)*,* [*RIX1*](http://db.yeastgenome.org/cgi-bin/locus.pl?locus=RIX1)*,* [*RLP7*](http://db.yeastgenome.org/cgi-bin/locus.pl?locus=RLP7)*,* [*RNT1*](http://db.yeastgenome.org/cgi-bin/locus.pl?locus=RNT1)*,* [*ROK1*](http://db.yeastgenome.org/cgi-bin/locus.pl?locus=ROK1)*,* [*ROX3*](http://db.yeastgenome.org/cgi-bin/locus.pl?locus=ROX3)*,* [*RPA12*](http://db.yeastgenome.org/cgi-bin/locus.pl?locus=RPA12)*,* [*RPA190*](http://db.yeastgenome.org/cgi-bin/locus.pl?locus=RPA190)*,* [*RPA34*](http://db.yeastgenome.org/cgi-bin/locus.pl?locus=RPA34)*,* [*RPA43*](http://db.yeastgenome.org/cgi-bin/locus.pl?locus=RPA43)*,* [*RPA49*](http://db.yeastgenome.org/cgi-bin/locus.pl?locus=RPA49)*,* [*RPB10*](http://db.yeastgenome.org/cgi-bin/locus.pl?locus=RPB10)*,* [*RPB5*](http://db.yeastgenome.org/cgi-bin/locus.pl?locus=RPB5)*,* [*RPB8*](http://db.yeastgenome.org/cgi-bin/locus.pl?locus=RPB8)*,* [*RPB9*](http://db.yeastgenome.org/cgi-bin/locus.pl?locus=RPB9)*,* [*RPC11*](http://db.yeastgenome.org/cgi-bin/locus.pl?locus=RPC11)*,* [*RPC19*](http://db.yeastgenome.org/cgi-bin/locus.pl?locus=RPC19)*,* [*RPC31*](http://db.yeastgenome.org/cgi-bin/locus.pl?locus=RPC31)*,* [*RPC34*](http://db.yeastgenome.org/cgi-bin/locus.pl?locus=RPC34)*,* [*RPC37*](http://db.yeastgenome.org/cgi-bin/locus.pl?locus=RPC37)*,* [*RPC40*](http://db.yeastgenome.org/cgi-bin/locus.pl?locus=RPC40)*,* [*RPC53*](http://db.yeastgenome.org/cgi-bin/locus.pl?locus=RPC53)*,* [*RPC82*](http://db.yeastgenome.org/cgi-bin/locus.pl?locus=RPC82)*,* [*RPF1*](http://db.yeastgenome.org/cgi-bin/locus.pl?locus=RPF1)*,* [*RPF2*](http://db.yeastgenome.org/cgi-bin/locus.pl?locus=RPF2)*,* [*RPO26*](http://db.yeastgenome.org/cgi-bin/locus.pl?locus=RPO26)*,* [*RPP1*](http://db.yeastgenome.org/cgi-bin/locus.pl?locus=RPP1)*,* [*RPS14B*](http://db.yeastgenome.org/cgi-bin/locus.pl?locus=RPS14B)*,* [*RPS19B*](http://db.yeastgenome.org/cgi-bin/locus.pl?locus=RPS19B)*,* [*RRN11*](http://db.yeastgenome.org/cgi-bin/locus.pl?locus=RRN11)*,* [*RRN7*](http://db.yeastgenome.org/cgi-bin/locus.pl?locus=RRN7)*,* [*RRP1*](http://db.yeastgenome.org/cgi-bin/locus.pl?locus=RRP1)*,* [*RRP12*](http://db.yeastgenome.org/cgi-bin/locus.pl?locus=RRP12)*,* [*RRP15*](http://db.yeastgenome.org/cgi-bin/locus.pl?locus=RRP15)*,* [*RRP17*](http://db.yeastgenome.org/cgi-bin/locus.pl?locus=RRP17)*,* [*RRP3*](http://db.yeastgenome.org/cgi-bin/locus.pl?locus=RRP3)*, RRP36,* [*RRP40*](http://db.yeastgenome.org/cgi-bin/locus.pl?locus=RRP40)*,* [*RRP43*](http://db.yeastgenome.org/cgi-bin/locus.pl?locus=RRP43)*,* [*RRP5*](http://db.yeastgenome.org/cgi-bin/locus.pl?locus=RRP5)*,* [*RRP6*](http://db.yeastgenome.org/cgi-bin/locus.pl?locus=RRP6)*,* [*RRP8*](http://db.yeastgenome.org/cgi-bin/locus.pl?locus=RRP8)*,* [*RRP9*](http://db.yeastgenome.org/cgi-bin/locus.pl?locus=RRP9)*,* [*RRS1*](http://db.yeastgenome.org/cgi-bin/locus.pl?locus=RRS1)*,* [*RVB1*](http://db.yeastgenome.org/cgi-bin/locus.pl?locus=RVB1)*,* [*SAS10*](http://db.yeastgenome.org/cgi-bin/locus.pl?locus=SAS10)*,* [*SEN34*](http://db.yeastgenome.org/cgi-bin/locus.pl?locus=SEN34)*,* [*SFG1*](http://db.yeastgenome.org/cgi-bin/locus.pl?locus=SFG1)*,* [*SHQ1*](http://db.yeastgenome.org/cgi-bin/locus.pl?locus=SHQ1)*,* [*SIK1*](http://db.yeastgenome.org/cgi-bin/locus.pl?locus=SIK1)*,* [*SIR2*](http://db.yeastgenome.org/cgi-bin/locus.pl?locus=SIR2)*,* [*SLX9*](http://db.yeastgenome.org/cgi-bin/locus.pl?locus=SLX9)*,* [*SNU13*](http://db.yeastgenome.org/cgi-bin/locus.pl?locus=SNU13)*,* [*SOF1*](http://db.yeastgenome.org/cgi-bin/locus.pl?locus=SOF1)*,* [*SPB1*](http://db.yeastgenome.org/cgi-bin/locus.pl?locus=SPB1)*,* [*SPB4*](http://db.yeastgenome.org/cgi-bin/locus.pl?locus=SPB4)*,* [*STE12*](http://db.yeastgenome.org/cgi-bin/locus.pl?locus=STE12)*,* [*SUT1*](http://db.yeastgenome.org/cgi-bin/locus.pl?locus=SUT1)*,* [*SUT2*](http://db.yeastgenome.org/cgi-bin/locus.pl?locus=SUT2)*,* [*SUV3*](http://db.yeastgenome.org/cgi-bin/locus.pl?locus=SUV3)*,* [*SWI5*](http://db.yeastgenome.org/cgi-bin/locus.pl?locus=SWI5)*,* [*TAD3*](http://db.yeastgenome.org/cgi-bin/locus.pl?locus=TAD3)*,* [*THP2*](http://db.yeastgenome.org/cgi-bin/locus.pl?locus=THP2)*,* [*TOP1*](http://db.yeastgenome.org/cgi-bin/locus.pl?locus=TOP1)*,* [*TOS4*](http://db.yeastgenome.org/cgi-bin/locus.pl?locus=TOS4)*,* [*TPA1*](http://db.yeastgenome.org/cgi-bin/locus.pl?locus=TPA1)*,* [*TRF5*](http://db.yeastgenome.org/cgi-bin/locus.pl?locus=TRF5)*,* [*TRM1*](http://db.yeastgenome.org/cgi-bin/locus.pl?locus=TRM1)*,* [*TRM10*](http://db.yeastgenome.org/cgi-bin/locus.pl?locus=TRM10)*,* [*TRM11*](http://db.yeastgenome.org/cgi-bin/locus.pl?locus=TRM11)*,* [*TRM112*](http://db.yeastgenome.org/cgi-bin/locus.pl?locus=TRM112)*,* [*TRM13*](http://db.yeastgenome.org/cgi-bin/locus.pl?locus=TRM13)*,* [*TRM2*](http://db.yeastgenome.org/cgi-bin/locus.pl?locus=TRM2)*,* [*TRM3*](http://db.yeastgenome.org/cgi-bin/locus.pl?locus=TRM3)*,* [*TRM7*](http://db.yeastgenome.org/cgi-bin/locus.pl?locus=TRM7)*,* [*TRM8*](http://db.yeastgenome.org/cgi-bin/locus.pl?locus=TRM8)*,* [*TRM82*](http://db.yeastgenome.org/cgi-bin/locus.pl?locus=TRM82)*,* [*TRM9*](http://db.yeastgenome.org/cgi-bin/locus.pl?locus=TRM9)*,* [*TSR1*](http://db.yeastgenome.org/cgi-bin/locus.pl?locus=TSR1)*,* [*TSR2*](http://db.yeastgenome.org/cgi-bin/locus.pl?locus=TSR2)*,* [*TYW1*](http://db.yeastgenome.org/cgi-bin/locus.pl?locus=TYW1)*,* [*UBP10*](http://db.yeastgenome.org/cgi-bin/locus.pl?locus=UBP10)*,* [*URB1*](http://db.yeastgenome.org/cgi-bin/locus.pl?locus=URB1)*,* [*URB2*](http://db.yeastgenome.org/cgi-bin/locus.pl?locus=URB2)*,* [*UTP10*](http://db.yeastgenome.org/cgi-bin/locus.pl?locus=UTP10)*,* [*UTP11*](http://db.yeastgenome.org/cgi-bin/locus.pl?locus=UTP11)*,* [*UTP13*](http://db.yeastgenome.org/cgi-bin/locus.pl?locus=UTP13)*,* [*UTP14*](http://db.yeastgenome.org/cgi-bin/locus.pl?locus=UTP14)*,* [*UTP15*](http://db.yeastgenome.org/cgi-bin/locus.pl?locus=UTP15)*,* [*UTP18*](http://db.yeastgenome.org/cgi-bin/locus.pl?locus=UTP18)*,* [*UTP21*](http://db.yeastgenome.org/cgi-bin/locus.pl?locus=UTP21)*,* [*UTP22*](http://db.yeastgenome.org/cgi-bin/locus.pl?locus=UTP22)*,* [*UTP23*](http://db.yeastgenome.org/cgi-bin/locus.pl?locus=UTP23)*,* [*UTP30*](http://db.yeastgenome.org/cgi-bin/locus.pl?locus=UTP30)*,* [*UTP4*](http://db.yeastgenome.org/cgi-bin/locus.pl?locus=UTP4)*,* [*UTP5*](http://db.yeastgenome.org/cgi-bin/locus.pl?locus=UTP5)*,* [*UTP7*](http://db.yeastgenome.org/cgi-bin/locus.pl?locus=UTP7)*,* [*UTP8*](http://db.yeastgenome.org/cgi-bin/locus.pl?locus=UTP8)*,* [*UTP9*](http://db.yeastgenome.org/cgi-bin/locus.pl?locus=UTP9)*,* [*WRS1*](http://db.yeastgenome.org/cgi-bin/locus.pl?locus=WRS1)*,* [*YHR020W*](http://db.yeastgenome.org/cgi-bin/locus.pl?locus=YHR020W)*,* [*YNL247W*](http://db.yeastgenome.org/cgi-bin/locus.pl?locus=YNL247W)*,* [*YOX1*](http://db.yeastgenome.org/cgi-bin/locus.pl?locus=YOX1) |
| Cellular component organization and biogenesis  Cellular component organization and biogenesis (cont.) | 326 out of 558 genes, 58.4% | 9.20e-45 | [*AAH1*](http://db.yeastgenome.org/cgi-bin/locus.pl?locus=AAH1)*,* [*AIR1*](http://db.yeastgenome.org/cgi-bin/locus.pl?locus=AIR1)*,* [*ALB1*](http://db.yeastgenome.org/cgi-bin/locus.pl?locus=ALB1)*,* [*ARB1*](http://db.yeastgenome.org/cgi-bin/locus.pl?locus=ARB1)*,* [*ARP1*](http://db.yeastgenome.org/cgi-bin/locus.pl?locus=ARP1)*,* [*ARX1*](http://db.yeastgenome.org/cgi-bin/locus.pl?locus=ARX1)*,* [*ASC1*](http://db.yeastgenome.org/cgi-bin/locus.pl?locus=ASC1)*,* [*ASH1*](http://db.yeastgenome.org/cgi-bin/locus.pl?locus=ASH1)*,* [*AST1*](http://db.yeastgenome.org/cgi-bin/locus.pl?locus=AST1)*,* [*ATC1*](http://db.yeastgenome.org/cgi-bin/locus.pl?locus=ATC1)*,* [*AVL9*](http://db.yeastgenome.org/cgi-bin/locus.pl?locus=AVL9)*,* [*BCP1*](http://db.yeastgenome.org/cgi-bin/locus.pl?locus=BCP1)*,* [*BFR2*](http://db.yeastgenome.org/cgi-bin/locus.pl?locus=BFR2)*,* [*BMS1*](http://db.yeastgenome.org/cgi-bin/locus.pl?locus=BMS1)*,* [*BRR6*](http://db.yeastgenome.org/cgi-bin/locus.pl?locus=BRR6)*,* [*BRX1*](http://db.yeastgenome.org/cgi-bin/locus.pl?locus=BRX1)*,* [*BST1*](http://db.yeastgenome.org/cgi-bin/locus.pl?locus=BST1)*,* [*BUD17*](http://db.yeastgenome.org/cgi-bin/locus.pl?locus=BUD17)*,* [*BUD21*](http://db.yeastgenome.org/cgi-bin/locus.pl?locus=BUD21)*,* [*BUD22*](http://db.yeastgenome.org/cgi-bin/locus.pl?locus=BUD22)*,* [*BUD23*](http://db.yeastgenome.org/cgi-bin/locus.pl?locus=BUD23)*,* [*BUD27*](http://db.yeastgenome.org/cgi-bin/locus.pl?locus=BUD27)*,* [*BUD32*](http://db.yeastgenome.org/cgi-bin/locus.pl?locus=BUD32)*,* [*BUD6*](http://db.yeastgenome.org/cgi-bin/locus.pl?locus=BUD6)*,* [*BUD8*](http://db.yeastgenome.org/cgi-bin/locus.pl?locus=BUD8)*,* [*CBF5*](http://db.yeastgenome.org/cgi-bin/locus.pl?locus=CBF5)*,* [*CDC20*](http://db.yeastgenome.org/cgi-bin/locus.pl?locus=CDC20)*,* [*CDC47*](http://db.yeastgenome.org/cgi-bin/locus.pl?locus=CDC47)*,* [*CGR1*](http://db.yeastgenome.org/cgi-bin/locus.pl?locus=CGR1)*,* [*CIC1*](http://db.yeastgenome.org/cgi-bin/locus.pl?locus=CIC1)*,* [*CIN4*](http://db.yeastgenome.org/cgi-bin/locus.pl?locus=CIN4)*,* [*CLA4*](http://db.yeastgenome.org/cgi-bin/locus.pl?locus=CLA4)*,* [*CLB1*](http://db.yeastgenome.org/cgi-bin/locus.pl?locus=CLB1)*, CMS1,* [*CSM3*](http://db.yeastgenome.org/cgi-bin/locus.pl?locus=CSM3)*,* [*CUS1*](http://db.yeastgenome.org/cgi-bin/locus.pl?locus=CUS1)*,* [*DBP10*](http://db.yeastgenome.org/cgi-bin/locus.pl?locus=DBP10)*,* [*DBP2*](http://db.yeastgenome.org/cgi-bin/locus.pl?locus=DBP2)*,* [*DBP3*](http://db.yeastgenome.org/cgi-bin/locus.pl?locus=DBP3)*,* [*DBP6*](http://db.yeastgenome.org/cgi-bin/locus.pl?locus=DBP6)*,* [*DBP7*](http://db.yeastgenome.org/cgi-bin/locus.pl?locus=DBP7)*,* [*DBP8*](http://db.yeastgenome.org/cgi-bin/locus.pl?locus=DBP8)*,* [*DBP9*](http://db.yeastgenome.org/cgi-bin/locus.pl?locus=DBP9)*,* [*DHR2*](http://db.yeastgenome.org/cgi-bin/locus.pl?locus=DHR2)*,* [*DIM1*](http://db.yeastgenome.org/cgi-bin/locus.pl?locus=DIM1)*,* [*DIP2*](http://db.yeastgenome.org/cgi-bin/locus.pl?locus=DIP2)*,* [*DPH2*](http://db.yeastgenome.org/cgi-bin/locus.pl?locus=DPH2)*,* [*DRS1*](http://db.yeastgenome.org/cgi-bin/locus.pl?locus=DRS1)*,* [*DSE1*](http://db.yeastgenome.org/cgi-bin/locus.pl?locus=DSE1)*,* [*DSE2*](http://db.yeastgenome.org/cgi-bin/locus.pl?locus=DSE2)*,* [*DUO1*](http://db.yeastgenome.org/cgi-bin/locus.pl?locus=DUO1)*,* [*DUS3*](http://db.yeastgenome.org/cgi-bin/locus.pl?locus=DUS3)*,* [*EBP2*](http://db.yeastgenome.org/cgi-bin/locus.pl?locus=EBP2)*,* [*ECM16*](http://db.yeastgenome.org/cgi-bin/locus.pl?locus=ECM16)*, EFG1,* [*EMG1*](http://db.yeastgenome.org/cgi-bin/locus.pl?locus=EMG1)*,* [*ENP1*](http://db.yeastgenome.org/cgi-bin/locus.pl?locus=ENP1)*,* [*ENP2*](http://db.yeastgenome.org/cgi-bin/locus.pl?locus=ENP2)*,* [*ERB1*](http://db.yeastgenome.org/cgi-bin/locus.pl?locus=ERB1)*,* [*ERG3*](http://db.yeastgenome.org/cgi-bin/locus.pl?locus=ERG3)*,* [*ESF1*](http://db.yeastgenome.org/cgi-bin/locus.pl?locus=ESF1)*,* [*ESF2*](http://db.yeastgenome.org/cgi-bin/locus.pl?locus=ESF2)*,* [*ESP1*](http://db.yeastgenome.org/cgi-bin/locus.pl?locus=ESP1)*,* [*EXG1*](http://db.yeastgenome.org/cgi-bin/locus.pl?locus=EXG1)*,* [*FAF1*](http://db.yeastgenome.org/cgi-bin/locus.pl?locus=FAF1)*,* [*FAL1*](http://db.yeastgenome.org/cgi-bin/locus.pl?locus=FAL1)*,* [*FAP7*](http://db.yeastgenome.org/cgi-bin/locus.pl?locus=FAP7)*,* [*FCF2*](http://db.yeastgenome.org/cgi-bin/locus.pl?locus=FCF2)*,* [*FET4*](http://db.yeastgenome.org/cgi-bin/locus.pl?locus=FET4)*,* [*FIG2*](http://db.yeastgenome.org/cgi-bin/locus.pl?locus=FIG2)*,* [*FKH1*](http://db.yeastgenome.org/cgi-bin/locus.pl?locus=FKH1)*,* [*FPR4*](http://db.yeastgenome.org/cgi-bin/locus.pl?locus=FPR4)*,* [*FYV7*](http://db.yeastgenome.org/cgi-bin/locus.pl?locus=FYV7)*,* [*GAR1*](http://db.yeastgenome.org/cgi-bin/locus.pl?locus=GAR1)*,* [*GCD10*](http://db.yeastgenome.org/cgi-bin/locus.pl?locus=GCD10)*,* [*GCD11*](http://db.yeastgenome.org/cgi-bin/locus.pl?locus=GCD11)*,* [*GCD14*](http://db.yeastgenome.org/cgi-bin/locus.pl?locus=GCD14)*,* [*GCD2*](http://db.yeastgenome.org/cgi-bin/locus.pl?locus=GCD2)*,* [*GCN3*](http://db.yeastgenome.org/cgi-bin/locus.pl?locus=GCN3)*,* [*GDT1*](http://db.yeastgenome.org/cgi-bin/locus.pl?locus=GDT1)*,* [*GEA1*](http://db.yeastgenome.org/cgi-bin/locus.pl?locus=GEA1)*,* [*GLE2*](http://db.yeastgenome.org/cgi-bin/locus.pl?locus=GLE2)*,* [*GRC3*](http://db.yeastgenome.org/cgi-bin/locus.pl?locus=GRC3)*,* [*HAS1*](http://db.yeastgenome.org/cgi-bin/locus.pl?locus=HAS1)*,* [*HCA4*](http://db.yeastgenome.org/cgi-bin/locus.pl?locus=HCA4)*,* [*HGH1*](http://db.yeastgenome.org/cgi-bin/locus.pl?locus=HGH1)*,* [*HHF2*](http://db.yeastgenome.org/cgi-bin/locus.pl?locus=HHF2)*,* [*HHT1*](http://db.yeastgenome.org/cgi-bin/locus.pl?locus=HHT1)*,* [*HIR2*](http://db.yeastgenome.org/cgi-bin/locus.pl?locus=HIR2)*,* [*HLR1*](http://db.yeastgenome.org/cgi-bin/locus.pl?locus=HLR1)*,* [*HMS2*](http://db.yeastgenome.org/cgi-bin/locus.pl?locus=HMS2)*,* [*HMT1*](http://db.yeastgenome.org/cgi-bin/locus.pl?locus=HMT1)*,* [*HST3*](http://db.yeastgenome.org/cgi-bin/locus.pl?locus=HST3)*,* [*HTA1*](http://db.yeastgenome.org/cgi-bin/locus.pl?locus=HTA1)*,* [*HTA2*](http://db.yeastgenome.org/cgi-bin/locus.pl?locus=HTA2)*,* [*HTB1*](http://db.yeastgenome.org/cgi-bin/locus.pl?locus=HTB1)*,* [*HTB2*](http://db.yeastgenome.org/cgi-bin/locus.pl?locus=HTB2)*,* [*HTZ1*](http://db.yeastgenome.org/cgi-bin/locus.pl?locus=HTZ1)*,* [*IFH1*](http://db.yeastgenome.org/cgi-bin/locus.pl?locus=IFH1)*,* [*ILV5*](http://db.yeastgenome.org/cgi-bin/locus.pl?locus=ILV5)*,* [*IMP3*](http://db.yeastgenome.org/cgi-bin/locus.pl?locus=IMP3)*,* [*IPI1*](http://db.yeastgenome.org/cgi-bin/locus.pl?locus=IPI1)*,* [*IPI3*](http://db.yeastgenome.org/cgi-bin/locus.pl?locus=IPI3)*,* [*KAE1*](http://db.yeastgenome.org/cgi-bin/locus.pl?locus=KAE1)*,* [*KAP123*](http://db.yeastgenome.org/cgi-bin/locus.pl?locus=KAP123)*,* [*KCS1*](http://db.yeastgenome.org/cgi-bin/locus.pl?locus=KCS1)*,* [*KRE33*](http://db.yeastgenome.org/cgi-bin/locus.pl?locus=KRE33)*,* [*KRI1*](http://db.yeastgenome.org/cgi-bin/locus.pl?locus=KRI1)*,* [*KRR1*](http://db.yeastgenome.org/cgi-bin/locus.pl?locus=KRR1)*,* [*KTI12*](http://db.yeastgenome.org/cgi-bin/locus.pl?locus=KTI12)*,* [*KTR5*](http://db.yeastgenome.org/cgi-bin/locus.pl?locus=KTR5)*,* [*LCP5*](http://db.yeastgenome.org/cgi-bin/locus.pl?locus=LCP5)*,* [*LIA1*](http://db.yeastgenome.org/cgi-bin/locus.pl?locus=LIA1)*,* [*LOC1*](http://db.yeastgenome.org/cgi-bin/locus.pl?locus=LOC1)*,* [*LSG1*](http://db.yeastgenome.org/cgi-bin/locus.pl?locus=LSG1)*,* [*MAK11*](http://db.yeastgenome.org/cgi-bin/locus.pl?locus=MAK11)*,* [*MAK16*](http://db.yeastgenome.org/cgi-bin/locus.pl?locus=MAK16)*,* [*MAK21*](http://db.yeastgenome.org/cgi-bin/locus.pl?locus=MAK21)*,* [*MAK3*](http://db.yeastgenome.org/cgi-bin/locus.pl?locus=MAK3)*,* [*MIS1*](http://db.yeastgenome.org/cgi-bin/locus.pl?locus=MIS1)*,* [*MOT1*](http://db.yeastgenome.org/cgi-bin/locus.pl?locus=MOT1)*,* [*MPP10*](http://db.yeastgenome.org/cgi-bin/locus.pl?locus=MPP10)*,* [*MRD1*](http://db.yeastgenome.org/cgi-bin/locus.pl?locus=MRD1)*,* [*MRT4*](http://db.yeastgenome.org/cgi-bin/locus.pl?locus=MRT4)*,* [*MSB1*](http://db.yeastgenome.org/cgi-bin/locus.pl?locus=MSB1)*,* [*MSH1*](http://db.yeastgenome.org/cgi-bin/locus.pl?locus=MSH1)*,* [*MTR3*](http://db.yeastgenome.org/cgi-bin/locus.pl?locus=MTR3)*,* [*NAF1*](http://db.yeastgenome.org/cgi-bin/locus.pl?locus=NAF1)*,* [*NAN1*](http://db.yeastgenome.org/cgi-bin/locus.pl?locus=NAN1)*,* [*NCL1*](http://db.yeastgenome.org/cgi-bin/locus.pl?locus=NCL1)*,* [*NCS2*](http://db.yeastgenome.org/cgi-bin/locus.pl?locus=NCS2)*,* [*NEW1*](http://db.yeastgenome.org/cgi-bin/locus.pl?locus=NEW1)*,* [*NHP2*](http://db.yeastgenome.org/cgi-bin/locus.pl?locus=NHP2)*,* [*NIP7*](http://db.yeastgenome.org/cgi-bin/locus.pl?locus=NIP7)*,* [*NMD3*](http://db.yeastgenome.org/cgi-bin/locus.pl?locus=NMD3)*,* [*NOB1*](http://db.yeastgenome.org/cgi-bin/locus.pl?locus=NOB1)*,* [*NOC2*](http://db.yeastgenome.org/cgi-bin/locus.pl?locus=NOC2)*,* [*NOC3*](http://db.yeastgenome.org/cgi-bin/locus.pl?locus=NOC3)*,* [*NOC4*](http://db.yeastgenome.org/cgi-bin/locus.pl?locus=NOC4)*,* [*NOG1*](http://db.yeastgenome.org/cgi-bin/locus.pl?locus=NOG1)*,* [*NOG2*](http://db.yeastgenome.org/cgi-bin/locus.pl?locus=NOG2)*,* [*NOP13*](http://db.yeastgenome.org/cgi-bin/locus.pl?locus=NOP13)*,* [*NOP14*](http://db.yeastgenome.org/cgi-bin/locus.pl?locus=NOP14)*,* [*NOP15*](http://db.yeastgenome.org/cgi-bin/locus.pl?locus=NOP15)*,* [*NOP16*](http://db.yeastgenome.org/cgi-bin/locus.pl?locus=NOP16)*,* [*NOP2*](http://db.yeastgenome.org/cgi-bin/locus.pl?locus=NOP2)*,* [*NOP4*](http://db.yeastgenome.org/cgi-bin/locus.pl?locus=NOP4)*,* [*NOP53*](http://db.yeastgenome.org/cgi-bin/locus.pl?locus=NOP53)*,* [*NOP58*](http://db.yeastgenome.org/cgi-bin/locus.pl?locus=NOP58)*,* [*NOP6*](http://db.yeastgenome.org/cgi-bin/locus.pl?locus=NOP6)*,* [*NOP7*](http://db.yeastgenome.org/cgi-bin/locus.pl?locus=NOP7)*,* [*NOP8*](http://db.yeastgenome.org/cgi-bin/locus.pl?locus=NOP8)*,* [*NOP9*](http://db.yeastgenome.org/cgi-bin/locus.pl?locus=NOP9)*,* [*NRP1*](http://db.yeastgenome.org/cgi-bin/locus.pl?locus=NRP1)*,* [*NSA1*](http://db.yeastgenome.org/cgi-bin/locus.pl?locus=NSA1)*,* [*NSR1*](http://db.yeastgenome.org/cgi-bin/locus.pl?locus=NSR1)*,* [*NUC1*](http://db.yeastgenome.org/cgi-bin/locus.pl?locus=NUC1)*,* [*NUG1*](http://db.yeastgenome.org/cgi-bin/locus.pl?locus=NUG1)*,* [*NUP1*](http://db.yeastgenome.org/cgi-bin/locus.pl?locus=NUP1)*,* [*ORC1*](http://db.yeastgenome.org/cgi-bin/locus.pl?locus=ORC1)*,* [*PHO5*](http://db.yeastgenome.org/cgi-bin/locus.pl?locus=PHO5)*,* [*PNO1*](http://db.yeastgenome.org/cgi-bin/locus.pl?locus=PNO1)*,* [*POP3*](http://db.yeastgenome.org/cgi-bin/locus.pl?locus=POP3)*,* [*POP6*](http://db.yeastgenome.org/cgi-bin/locus.pl?locus=POP6)*,* [*POP8*](http://db.yeastgenome.org/cgi-bin/locus.pl?locus=POP8)*,* [*PPT1*](http://db.yeastgenome.org/cgi-bin/locus.pl?locus=PPT1)*,* [*PRO1*](http://db.yeastgenome.org/cgi-bin/locus.pl?locus=PRO1)*,* [*PRP24*](http://db.yeastgenome.org/cgi-bin/locus.pl?locus=PRP24)*,* [*PRP43*](http://db.yeastgenome.org/cgi-bin/locus.pl?locus=PRP43)*,* [*PRS4*](http://db.yeastgenome.org/cgi-bin/locus.pl?locus=PRS4)*,* [*PUF6*](http://db.yeastgenome.org/cgi-bin/locus.pl?locus=PUF6)*,* [*PUS1*](http://db.yeastgenome.org/cgi-bin/locus.pl?locus=PUS1)*,* [*PUS7*](http://db.yeastgenome.org/cgi-bin/locus.pl?locus=PUS7)*,* [*PWP1*](http://db.yeastgenome.org/cgi-bin/locus.pl?locus=PWP1)*,* [*PWP2*](http://db.yeastgenome.org/cgi-bin/locus.pl?locus=PWP2)*,* [*PXR1*](http://db.yeastgenome.org/cgi-bin/locus.pl?locus=PXR1)*,* [*RAP1*](http://db.yeastgenome.org/cgi-bin/locus.pl?locus=RAP1)*,* [*RBG1*](http://db.yeastgenome.org/cgi-bin/locus.pl?locus=RBG1)*,* [*RCL1*](http://db.yeastgenome.org/cgi-bin/locus.pl?locus=RCL1)*,* [*REI1*](http://db.yeastgenome.org/cgi-bin/locus.pl?locus=REI1)*,* [*REX4*](http://db.yeastgenome.org/cgi-bin/locus.pl?locus=REX4)*,* [*RIO1*](http://db.yeastgenome.org/cgi-bin/locus.pl?locus=RIO1)*,* [*RIX1*](http://db.yeastgenome.org/cgi-bin/locus.pl?locus=RIX1)*,* [*RIX7*](http://db.yeastgenome.org/cgi-bin/locus.pl?locus=RIX7)*,* [*RKI1*](http://db.yeastgenome.org/cgi-bin/locus.pl?locus=RKI1)*,* [*RLI1*](http://db.yeastgenome.org/cgi-bin/locus.pl?locus=RLI1)*,* [*RLP24*](http://db.yeastgenome.org/cgi-bin/locus.pl?locus=RLP24)*,* [*RLP7*](http://db.yeastgenome.org/cgi-bin/locus.pl?locus=RLP7)*,* [*RMT2*](http://db.yeastgenome.org/cgi-bin/locus.pl?locus=RMT2)*,* [*RNT1*](http://db.yeastgenome.org/cgi-bin/locus.pl?locus=RNT1)*,* [*ROK1*](http://db.yeastgenome.org/cgi-bin/locus.pl?locus=ROK1)*,* [*RPA12*](http://db.yeastgenome.org/cgi-bin/locus.pl?locus=RPA12)*,* [*RPA34*](http://db.yeastgenome.org/cgi-bin/locus.pl?locus=RPA34)*,* [*RPA43*](http://db.yeastgenome.org/cgi-bin/locus.pl?locus=RPA43)*,* [*RPA49*](http://db.yeastgenome.org/cgi-bin/locus.pl?locus=RPA49)*,* [*RPB9*](http://db.yeastgenome.org/cgi-bin/locus.pl?locus=RPB9)*,* [*RPC19*](http://db.yeastgenome.org/cgi-bin/locus.pl?locus=RPC19)*,* [*RPC34*](http://db.yeastgenome.org/cgi-bin/locus.pl?locus=RPC34)*,* [*RPC40*](http://db.yeastgenome.org/cgi-bin/locus.pl?locus=RPC40)*,* [*RPC82*](http://db.yeastgenome.org/cgi-bin/locus.pl?locus=RPC82)*,* [*RPF1*](http://db.yeastgenome.org/cgi-bin/locus.pl?locus=RPF1)*,* [*RPF2*](http://db.yeastgenome.org/cgi-bin/locus.pl?locus=RPF2)*,* [*RPP1*](http://db.yeastgenome.org/cgi-bin/locus.pl?locus=RPP1)*,* [*RPS14B*](http://db.yeastgenome.org/cgi-bin/locus.pl?locus=RPS14B)*,* [*RPS19B*](http://db.yeastgenome.org/cgi-bin/locus.pl?locus=RPS19B)*,* [*RPS21A*](http://db.yeastgenome.org/cgi-bin/locus.pl?locus=RPS21A)*,* [*RRB1*](http://db.yeastgenome.org/cgi-bin/locus.pl?locus=RRB1)*,* [*RRM3*](http://db.yeastgenome.org/cgi-bin/locus.pl?locus=RRM3)*,* [*RRP1*](http://db.yeastgenome.org/cgi-bin/locus.pl?locus=RRP1)*,* [*RRP12*](http://db.yeastgenome.org/cgi-bin/locus.pl?locus=RRP12)*,* [*RRP14*](http://db.yeastgenome.org/cgi-bin/locus.pl?locus=RRP14)*,* [*RRP15*](http://db.yeastgenome.org/cgi-bin/locus.pl?locus=RRP15)*,* [*RRP17*](http://db.yeastgenome.org/cgi-bin/locus.pl?locus=RRP17)*,* [*RRP3*](http://db.yeastgenome.org/cgi-bin/locus.pl?locus=RRP3)*, RRP36,* [*RRP40*](http://db.yeastgenome.org/cgi-bin/locus.pl?locus=RRP40)*,* [*RRP43*](http://db.yeastgenome.org/cgi-bin/locus.pl?locus=RRP43)*,* [*RRP5*](http://db.yeastgenome.org/cgi-bin/locus.pl?locus=RRP5)*,* [*RRP6*](http://db.yeastgenome.org/cgi-bin/locus.pl?locus=RRP6)*,* [*RRP8*](http://db.yeastgenome.org/cgi-bin/locus.pl?locus=RRP8)*,* [*RRP9*](http://db.yeastgenome.org/cgi-bin/locus.pl?locus=RRP9)*,* [*RRS1*](http://db.yeastgenome.org/cgi-bin/locus.pl?locus=RRS1) *,RRT14,* [*RSA1*](http://db.yeastgenome.org/cgi-bin/locus.pl?locus=RSA1)*,* [*RSA3*](http://db.yeastgenome.org/cgi-bin/locus.pl?locus=RSA3)*,* [*RSA4*](http://db.yeastgenome.org/cgi-bin/locus.pl?locus=RSA4)*,* [*RVB1*](http://db.yeastgenome.org/cgi-bin/locus.pl?locus=RVB1)*,* [*SAN1*](http://db.yeastgenome.org/cgi-bin/locus.pl?locus=SAN1)*,* [*SAS10*](http://db.yeastgenome.org/cgi-bin/locus.pl?locus=SAS10)*,* [*SCC2*](http://db.yeastgenome.org/cgi-bin/locus.pl?locus=SCC2)*,* [*SCH9*](http://db.yeastgenome.org/cgi-bin/locus.pl?locus=SCH9)*,* [*SDA1*](http://db.yeastgenome.org/cgi-bin/locus.pl?locus=SDA1)*,* [*SEC53*](http://db.yeastgenome.org/cgi-bin/locus.pl?locus=SEC53)*, SEE1,* [*SFG1*](http://db.yeastgenome.org/cgi-bin/locus.pl?locus=SFG1)*,* [*SGO1*](http://db.yeastgenome.org/cgi-bin/locus.pl?locus=SGO1)*,* [*SHO1*](http://db.yeastgenome.org/cgi-bin/locus.pl?locus=SHO1)*,* [*SIK1*](http://db.yeastgenome.org/cgi-bin/locus.pl?locus=SIK1)*,* [*SIM1*](http://db.yeastgenome.org/cgi-bin/locus.pl?locus=SIM1)*,* [*SIR2*](http://db.yeastgenome.org/cgi-bin/locus.pl?locus=SIR2)*,* [*SLI15*](http://db.yeastgenome.org/cgi-bin/locus.pl?locus=SLI15)*,* [*SLK19*](http://db.yeastgenome.org/cgi-bin/locus.pl?locus=SLK19)*,* [*SLX9*](http://db.yeastgenome.org/cgi-bin/locus.pl?locus=SLX9)*,* [*SLY41*](http://db.yeastgenome.org/cgi-bin/locus.pl?locus=SLY41)*,* [*SMC2*](http://db.yeastgenome.org/cgi-bin/locus.pl?locus=SMC2)*,* [*SMI1*](http://db.yeastgenome.org/cgi-bin/locus.pl?locus=SMI1)*,* [*SMY2*](http://db.yeastgenome.org/cgi-bin/locus.pl?locus=SMY2)*,* [*SNU13*](http://db.yeastgenome.org/cgi-bin/locus.pl?locus=SNU13)*,* [*SOF1*](http://db.yeastgenome.org/cgi-bin/locus.pl?locus=SOF1)*,* [*SPA2*](http://db.yeastgenome.org/cgi-bin/locus.pl?locus=SPA2)*,* [*SPB1*](http://db.yeastgenome.org/cgi-bin/locus.pl?locus=SPB1)*,* [*SPB4*](http://db.yeastgenome.org/cgi-bin/locus.pl?locus=SPB4)*,* [*SPC98*](http://db.yeastgenome.org/cgi-bin/locus.pl?locus=SPC98)*,* [*SQT1*](http://db.yeastgenome.org/cgi-bin/locus.pl?locus=SQT1)*,* [*SRO9*](http://db.yeastgenome.org/cgi-bin/locus.pl?locus=SRO9)*,* [*SRP40*](http://db.yeastgenome.org/cgi-bin/locus.pl?locus=SRP40)*,* [*SSF1*](http://db.yeastgenome.org/cgi-bin/locus.pl?locus=SSF1)*,* [*SSF2*](http://db.yeastgenome.org/cgi-bin/locus.pl?locus=SSF2)*,* [*STE12*](http://db.yeastgenome.org/cgi-bin/locus.pl?locus=STE12)*,* [*STU2*](http://db.yeastgenome.org/cgi-bin/locus.pl?locus=STU2)*,* [*SUN4*](http://db.yeastgenome.org/cgi-bin/locus.pl?locus=SUN4)*,* [*SUR4*](http://db.yeastgenome.org/cgi-bin/locus.pl?locus=SUR4)*,* [*SUR7*](http://db.yeastgenome.org/cgi-bin/locus.pl?locus=SUR7)*,* [*SXM1*](http://db.yeastgenome.org/cgi-bin/locus.pl?locus=SXM1)*,* [*TEL2*](http://db.yeastgenome.org/cgi-bin/locus.pl?locus=TEL2)*,* [*THP2*](http://db.yeastgenome.org/cgi-bin/locus.pl?locus=THP2)*,* [*TIF2*](http://db.yeastgenome.org/cgi-bin/locus.pl?locus=TIF2)*,* [*TIF3*](http://db.yeastgenome.org/cgi-bin/locus.pl?locus=TIF3)*,* [*TIF35*](http://db.yeastgenome.org/cgi-bin/locus.pl?locus=TIF35)*,* [*TIF4631*](http://db.yeastgenome.org/cgi-bin/locus.pl?locus=TIF4631)*,* [*TIP20*](http://db.yeastgenome.org/cgi-bin/locus.pl?locus=TIP20)*,* [*TMA23*](http://db.yeastgenome.org/cgi-bin/locus.pl?locus=TMA23)*,* [*TMA46*](http://db.yeastgenome.org/cgi-bin/locus.pl?locus=TMA46)*, TOB6,* [*TOP1*](http://db.yeastgenome.org/cgi-bin/locus.pl?locus=TOP1)*,* [*TPA1*](http://db.yeastgenome.org/cgi-bin/locus.pl?locus=TPA1)*,* [*TRF5*](http://db.yeastgenome.org/cgi-bin/locus.pl?locus=TRF5)*,* [*TRM1*](http://db.yeastgenome.org/cgi-bin/locus.pl?locus=TRM1)*,* [*TRM11*](http://db.yeastgenome.org/cgi-bin/locus.pl?locus=TRM11)*,* [*TRM2*](http://db.yeastgenome.org/cgi-bin/locus.pl?locus=TRM2)*,* [*TRM8*](http://db.yeastgenome.org/cgi-bin/locus.pl?locus=TRM8)*,* [*TRM82*](http://db.yeastgenome.org/cgi-bin/locus.pl?locus=TRM82)*,* [*TSR1*](http://db.yeastgenome.org/cgi-bin/locus.pl?locus=TSR1)*,* [*TSR2*](http://db.yeastgenome.org/cgi-bin/locus.pl?locus=TSR2)*,* [*UBC9*](http://db.yeastgenome.org/cgi-bin/locus.pl?locus=UBC9)*,* [*UBP10*](http://db.yeastgenome.org/cgi-bin/locus.pl?locus=UBP10)*,* [*URB1*](http://db.yeastgenome.org/cgi-bin/locus.pl?locus=URB1)*,* [*URB2*](http://db.yeastgenome.org/cgi-bin/locus.pl?locus=URB2)*,* [*URK1*](http://db.yeastgenome.org/cgi-bin/locus.pl?locus=URK1)*,* [*UTP10*](http://db.yeastgenome.org/cgi-bin/locus.pl?locus=UTP10)*,* [*UTP11*](http://db.yeastgenome.org/cgi-bin/locus.pl?locus=UTP11)*,* [*UTP13*](http://db.yeastgenome.org/cgi-bin/locus.pl?locus=UTP13)*,* [*UTP14*](http://db.yeastgenome.org/cgi-bin/locus.pl?locus=UTP14)*,* [*UTP15*](http://db.yeastgenome.org/cgi-bin/locus.pl?locus=UTP15)*,* [*UTP18*](http://db.yeastgenome.org/cgi-bin/locus.pl?locus=UTP18)*,* [*UTP21*](http://db.yeastgenome.org/cgi-bin/locus.pl?locus=UTP21)*,* [*UTP22*](http://db.yeastgenome.org/cgi-bin/locus.pl?locus=UTP22)*,* [*UTP23*](http://db.yeastgenome.org/cgi-bin/locus.pl?locus=UTP23)*,* [*UTP30*](http://db.yeastgenome.org/cgi-bin/locus.pl?locus=UTP30)*,* [*UTP4*](http://db.yeastgenome.org/cgi-bin/locus.pl?locus=UTP4)*,* [*UTP5*](http://db.yeastgenome.org/cgi-bin/locus.pl?locus=UTP5)*,* [*UTP7*](http://db.yeastgenome.org/cgi-bin/locus.pl?locus=UTP7)*,* [*UTP8*](http://db.yeastgenome.org/cgi-bin/locus.pl?locus=UTP8)*,* [*UTP9*](http://db.yeastgenome.org/cgi-bin/locus.pl?locus=UTP9)*,* [*UTR2*](http://db.yeastgenome.org/cgi-bin/locus.pl?locus=UTR2)*,* [*VTC1*](http://db.yeastgenome.org/cgi-bin/locus.pl?locus=VTC1)*,* [*VTC3*](http://db.yeastgenome.org/cgi-bin/locus.pl?locus=VTC3)*,* [*YAR1*](http://db.yeastgenome.org/cgi-bin/locus.pl?locus=YAR1)*,* [*YBL028C*](http://db.yeastgenome.org/cgi-bin/locus.pl?locus=YBL028C)*,* [*YBR238C*](http://db.yeastgenome.org/cgi-bin/locus.pl?locus=YBR238C)*,* [*YCG1*](http://db.yeastgenome.org/cgi-bin/locus.pl?locus=YCG1)*,* [*YCR016W*](http://db.yeastgenome.org/cgi-bin/locus.pl?locus=YCR016W)*,* [*YDL063C*](http://db.yeastgenome.org/cgi-bin/locus.pl?locus=YDL063C)*,* [*YEF3*](http://db.yeastgenome.org/cgi-bin/locus.pl?locus=YEF3)*,* [*YGR283C*](http://db.yeastgenome.org/cgi-bin/locus.pl?locus=YGR283C)*,* [*YHL039W*](http://db.yeastgenome.org/cgi-bin/locus.pl?locus=YHL039W)*,* [*YHM2*](http://db.yeastgenome.org/cgi-bin/locus.pl?locus=YHM2)*,* [*YIL096C*](http://db.yeastgenome.org/cgi-bin/locus.pl?locus=YIL096C)*,* [*YMR310C*](http://db.yeastgenome.org/cgi-bin/locus.pl?locus=YMR310C)*,* [*YNL022C*](http://db.yeastgenome.org/cgi-bin/locus.pl?locus=YNL022C)*,* [*YNL247W*](http://db.yeastgenome.org/cgi-bin/locus.pl?locus=YNL247W)*,* [*YNL313C*](http://db.yeastgenome.org/cgi-bin/locus.pl?locus=YNL313C)*,* [*YOR021C*](http://db.yeastgenome.org/cgi-bin/locus.pl?locus=YOR021C)*,* [*YRF1-6*](http://db.yeastgenome.org/cgi-bin/locus.pl?locus=YRF1-6)*,* [*YTM1*](http://db.yeastgenome.org/cgi-bin/locus.pl?locus=YTM1)*,* [*YVH1*](http://db.yeastgenome.org/cgi-bin/locus.pl?locus=YVH1) |
| Nucleobase, nucleoside, nucleotide and nucleic acid metabolic process | 257 out of 558 genes, 46.1% | 1.54e-41 | [*AAH1*](http://db.yeastgenome.org/cgi-bin/locus.pl?locus=AAH1)*,* [*ADE17*](http://db.yeastgenome.org/cgi-bin/locus.pl?locus=ADE17)*,* [*ADE5,7*](http://db.yeastgenome.org/cgi-bin/locus.pl?locus=ADE5,7)*,* [*ADE6*](http://db.yeastgenome.org/cgi-bin/locus.pl?locus=ADE6)*,* [*ADE8*](http://db.yeastgenome.org/cgi-bin/locus.pl?locus=ADE8)*,* [*AIR1*](http://db.yeastgenome.org/cgi-bin/locus.pl?locus=AIR1)*,* [*APT1*](http://db.yeastgenome.org/cgi-bin/locus.pl?locus=APT1)*,* [*ASH1*](http://db.yeastgenome.org/cgi-bin/locus.pl?locus=ASH1)*,* [*BCD1*](http://db.yeastgenome.org/cgi-bin/locus.pl?locus=BCD1)*,* [*BMS1*](http://db.yeastgenome.org/cgi-bin/locus.pl?locus=BMS1)*,* [*BUD21*](http://db.yeastgenome.org/cgi-bin/locus.pl?locus=BUD21)*,* [*BUD32*](http://db.yeastgenome.org/cgi-bin/locus.pl?locus=BUD32)*,* [*CBF5*](http://db.yeastgenome.org/cgi-bin/locus.pl?locus=CBF5)*,* [*CDC47*](http://db.yeastgenome.org/cgi-bin/locus.pl?locus=CDC47)*,* [*CGR1*](http://db.yeastgenome.org/cgi-bin/locus.pl?locus=CGR1)*, CMS1,* [*CSM3*](http://db.yeastgenome.org/cgi-bin/locus.pl?locus=CSM3)*,* [*CUS1*](http://db.yeastgenome.org/cgi-bin/locus.pl?locus=CUS1)*,* [*CWC2*](http://db.yeastgenome.org/cgi-bin/locus.pl?locus=CWC2)*,* [*DBP10*](http://db.yeastgenome.org/cgi-bin/locus.pl?locus=DBP10)*,* [*DBP2*](http://db.yeastgenome.org/cgi-bin/locus.pl?locus=DBP2)*,* [*DBP3*](http://db.yeastgenome.org/cgi-bin/locus.pl?locus=DBP3)*,* [*DBP6*](http://db.yeastgenome.org/cgi-bin/locus.pl?locus=DBP6)*,* [*DBP7*](http://db.yeastgenome.org/cgi-bin/locus.pl?locus=DBP7)*,* [*DBP8*](http://db.yeastgenome.org/cgi-bin/locus.pl?locus=DBP8)*,* [*DBP9*](http://db.yeastgenome.org/cgi-bin/locus.pl?locus=DBP9)*,* [*DIM1*](http://db.yeastgenome.org/cgi-bin/locus.pl?locus=DIM1)*,* [*DIP2*](http://db.yeastgenome.org/cgi-bin/locus.pl?locus=DIP2)*,* [*DPB2*](http://db.yeastgenome.org/cgi-bin/locus.pl?locus=DPB2)*,* [*DRS1*](http://db.yeastgenome.org/cgi-bin/locus.pl?locus=DRS1)*,* [*DUS1*](http://db.yeastgenome.org/cgi-bin/locus.pl?locus=DUS1)*,* [*DUS3*](http://db.yeastgenome.org/cgi-bin/locus.pl?locus=DUS3)*,* [*DUS4*](http://db.yeastgenome.org/cgi-bin/locus.pl?locus=DUS4)*,* [*DUT1*](http://db.yeastgenome.org/cgi-bin/locus.pl?locus=DUT1)*,* [*EBP2*](http://db.yeastgenome.org/cgi-bin/locus.pl?locus=EBP2)*,* [*ECM16*](http://db.yeastgenome.org/cgi-bin/locus.pl?locus=ECM16)*, EFG1,* [*ELP2*](http://db.yeastgenome.org/cgi-bin/locus.pl?locus=ELP2)*,* [*ELP3*](http://db.yeastgenome.org/cgi-bin/locus.pl?locus=ELP3)*,* [*EMG1*](http://db.yeastgenome.org/cgi-bin/locus.pl?locus=EMG1)*,* [*ENP1*](http://db.yeastgenome.org/cgi-bin/locus.pl?locus=ENP1)*,* [*ENP2*](http://db.yeastgenome.org/cgi-bin/locus.pl?locus=ENP2)*,* [*ERB1*](http://db.yeastgenome.org/cgi-bin/locus.pl?locus=ERB1)*,* [*ESF1*](http://db.yeastgenome.org/cgi-bin/locus.pl?locus=ESF1)*,* [*ESF2*](http://db.yeastgenome.org/cgi-bin/locus.pl?locus=ESF2)*,* [*FAF1*](http://db.yeastgenome.org/cgi-bin/locus.pl?locus=FAF1)*,* [*FAL1*](http://db.yeastgenome.org/cgi-bin/locus.pl?locus=FAL1)*,* [*FAP7*](http://db.yeastgenome.org/cgi-bin/locus.pl?locus=FAP7)*,* [*FCF2*](http://db.yeastgenome.org/cgi-bin/locus.pl?locus=FCF2)*,* [*FKH1*](http://db.yeastgenome.org/cgi-bin/locus.pl?locus=FKH1)*,* [*FPR4*](http://db.yeastgenome.org/cgi-bin/locus.pl?locus=FPR4)*,* [*FUR1*](http://db.yeastgenome.org/cgi-bin/locus.pl?locus=FUR1)*,* [*FYV7*](http://db.yeastgenome.org/cgi-bin/locus.pl?locus=FYV7)*,* [*GAR1*](http://db.yeastgenome.org/cgi-bin/locus.pl?locus=GAR1)*,* [*GCD10*](http://db.yeastgenome.org/cgi-bin/locus.pl?locus=GCD10)*,* [*GCD14*](http://db.yeastgenome.org/cgi-bin/locus.pl?locus=GCD14)*,* [*GCR1*](http://db.yeastgenome.org/cgi-bin/locus.pl?locus=GCR1)*,* [*GCR2*](http://db.yeastgenome.org/cgi-bin/locus.pl?locus=GCR2)*,* [*GLN4*](http://db.yeastgenome.org/cgi-bin/locus.pl?locus=GLN4)*,* [*GRC3*](http://db.yeastgenome.org/cgi-bin/locus.pl?locus=GRC3)*,* [*GUA1*](http://db.yeastgenome.org/cgi-bin/locus.pl?locus=GUA1)*,* [*HAS1*](http://db.yeastgenome.org/cgi-bin/locus.pl?locus=HAS1)*,* [*HCA4*](http://db.yeastgenome.org/cgi-bin/locus.pl?locus=HCA4)*,* [*HEM1*](http://db.yeastgenome.org/cgi-bin/locus.pl?locus=HEM1)*,* [*HHF2*](http://db.yeastgenome.org/cgi-bin/locus.pl?locus=HHF2)*,* [*HHT1*](http://db.yeastgenome.org/cgi-bin/locus.pl?locus=HHT1)*,* [*HIR2*](http://db.yeastgenome.org/cgi-bin/locus.pl?locus=HIR2)*,* [*HPT1*](http://db.yeastgenome.org/cgi-bin/locus.pl?locus=HPT1)*,* [*HST3*](http://db.yeastgenome.org/cgi-bin/locus.pl?locus=HST3)*,* [*HTA1*](http://db.yeastgenome.org/cgi-bin/locus.pl?locus=HTA1)*,* [*HTA2*](http://db.yeastgenome.org/cgi-bin/locus.pl?locus=HTA2)*,* [*HTB1*](http://db.yeastgenome.org/cgi-bin/locus.pl?locus=HTB1)*,* [*HTB2*](http://db.yeastgenome.org/cgi-bin/locus.pl?locus=HTB2)*,* [*HTZ1*](http://db.yeastgenome.org/cgi-bin/locus.pl?locus=HTZ1)*,* [*IFH1*](http://db.yeastgenome.org/cgi-bin/locus.pl?locus=IFH1)*,* [*IMD3*](http://db.yeastgenome.org/cgi-bin/locus.pl?locus=IMD3)*,* [*IMP3*](http://db.yeastgenome.org/cgi-bin/locus.pl?locus=IMP3)*,* [*INO2*](http://db.yeastgenome.org/cgi-bin/locus.pl?locus=INO2)*,* [*IPI1*](http://db.yeastgenome.org/cgi-bin/locus.pl?locus=IPI1)*,* [*IPI3*](http://db.yeastgenome.org/cgi-bin/locus.pl?locus=IPI3)*,* [*KAE1*](http://db.yeastgenome.org/cgi-bin/locus.pl?locus=KAE1)*,* [*KRR1*](http://db.yeastgenome.org/cgi-bin/locus.pl?locus=KRR1)*,* [*KTI12*](http://db.yeastgenome.org/cgi-bin/locus.pl?locus=KTI12)*,* [*LCP5*](http://db.yeastgenome.org/cgi-bin/locus.pl?locus=LCP5)*,* [*MAK11*](http://db.yeastgenome.org/cgi-bin/locus.pl?locus=MAK11)*,* [*MAK16*](http://db.yeastgenome.org/cgi-bin/locus.pl?locus=MAK16)*,* [*MES1*](http://db.yeastgenome.org/cgi-bin/locus.pl?locus=MES1)*,* [*MIS1*](http://db.yeastgenome.org/cgi-bin/locus.pl?locus=MIS1)*,* [*MOT1*](http://db.yeastgenome.org/cgi-bin/locus.pl?locus=MOT1)*,* [*MPP10*](http://db.yeastgenome.org/cgi-bin/locus.pl?locus=MPP10)*,* [*MRD1*](http://db.yeastgenome.org/cgi-bin/locus.pl?locus=MRD1)*,* [*MRT4*](http://db.yeastgenome.org/cgi-bin/locus.pl?locus=MRT4)*,* [*MSH1*](http://db.yeastgenome.org/cgi-bin/locus.pl?locus=MSH1)*,* [*MSH6*](http://db.yeastgenome.org/cgi-bin/locus.pl?locus=MSH6)*,* [*MTD1*](http://db.yeastgenome.org/cgi-bin/locus.pl?locus=MTD1)*,* [*MTR3*](http://db.yeastgenome.org/cgi-bin/locus.pl?locus=MTR3)*,* [*NAF1*](http://db.yeastgenome.org/cgi-bin/locus.pl?locus=NAF1)*,* [*NAN1*](http://db.yeastgenome.org/cgi-bin/locus.pl?locus=NAN1)*,* [*NCL1*](http://db.yeastgenome.org/cgi-bin/locus.pl?locus=NCL1)*,* [*NHP2*](http://db.yeastgenome.org/cgi-bin/locus.pl?locus=NHP2)*,* [*NIP7*](http://db.yeastgenome.org/cgi-bin/locus.pl?locus=NIP7)*,* [*NOB1*](http://db.yeastgenome.org/cgi-bin/locus.pl?locus=NOB1)*,* [*NOC3*](http://db.yeastgenome.org/cgi-bin/locus.pl?locus=NOC3)*,* [*NOC4*](http://db.yeastgenome.org/cgi-bin/locus.pl?locus=NOC4)*,* [*NOG1*](http://db.yeastgenome.org/cgi-bin/locus.pl?locus=NOG1)*,* [*NOP12*](http://db.yeastgenome.org/cgi-bin/locus.pl?locus=NOP12)*,* [*NOP14*](http://db.yeastgenome.org/cgi-bin/locus.pl?locus=NOP14)*,* [*NOP2*](http://db.yeastgenome.org/cgi-bin/locus.pl?locus=NOP2)*,* [*NOP4*](http://db.yeastgenome.org/cgi-bin/locus.pl?locus=NOP4)*,* [*NOP53*](http://db.yeastgenome.org/cgi-bin/locus.pl?locus=NOP53)*,* [*NOP58*](http://db.yeastgenome.org/cgi-bin/locus.pl?locus=NOP58)*,* [*NOP6*](http://db.yeastgenome.org/cgi-bin/locus.pl?locus=NOP6)*,* [*NOP7*](http://db.yeastgenome.org/cgi-bin/locus.pl?locus=NOP7)*,* [*NOP8*](http://db.yeastgenome.org/cgi-bin/locus.pl?locus=NOP8)*,* [*NOP9*](http://db.yeastgenome.org/cgi-bin/locus.pl?locus=NOP9)*,* [*NSE4*](http://db.yeastgenome.org/cgi-bin/locus.pl?locus=NSE4)*,* [*NSR1*](http://db.yeastgenome.org/cgi-bin/locus.pl?locus=NSR1)*,* [*NUC1*](http://db.yeastgenome.org/cgi-bin/locus.pl?locus=NUC1)*,* [*NUG1*](http://db.yeastgenome.org/cgi-bin/locus.pl?locus=NUG1)*,* [*ORC1*](http://db.yeastgenome.org/cgi-bin/locus.pl?locus=ORC1)*,* [*PNO1*](http://db.yeastgenome.org/cgi-bin/locus.pl?locus=PNO1)*,* [*POL5*](http://db.yeastgenome.org/cgi-bin/locus.pl?locus=POL5)*,* [*POP3*](http://db.yeastgenome.org/cgi-bin/locus.pl?locus=POP3)*,* [*POP6*](http://db.yeastgenome.org/cgi-bin/locus.pl?locus=POP6)*,* [*POP8*](http://db.yeastgenome.org/cgi-bin/locus.pl?locus=POP8)*,* [*PRP19*](http://db.yeastgenome.org/cgi-bin/locus.pl?locus=PRP19)*,* [*PRP24*](http://db.yeastgenome.org/cgi-bin/locus.pl?locus=PRP24)*,* [*PRP43*](http://db.yeastgenome.org/cgi-bin/locus.pl?locus=PRP43)*,* [*PRS1*](http://db.yeastgenome.org/cgi-bin/locus.pl?locus=PRS1)*,* [*PRS2*](http://db.yeastgenome.org/cgi-bin/locus.pl?locus=PRS2)*,* [*PRS4*](http://db.yeastgenome.org/cgi-bin/locus.pl?locus=PRS4)*,* [*PUF6*](http://db.yeastgenome.org/cgi-bin/locus.pl?locus=PUF6)*,* [*PUS1*](http://db.yeastgenome.org/cgi-bin/locus.pl?locus=PUS1)*,* [*PUS4*](http://db.yeastgenome.org/cgi-bin/locus.pl?locus=PUS4)*,* [*PUS7*](http://db.yeastgenome.org/cgi-bin/locus.pl?locus=PUS7)*,* [*PWP1*](http://db.yeastgenome.org/cgi-bin/locus.pl?locus=PWP1)*,* [*PWP2*](http://db.yeastgenome.org/cgi-bin/locus.pl?locus=PWP2)*,* [*PXR1*](http://db.yeastgenome.org/cgi-bin/locus.pl?locus=PXR1)*,* [*RAP1*](http://db.yeastgenome.org/cgi-bin/locus.pl?locus=RAP1)*,* [*RBA50*](http://db.yeastgenome.org/cgi-bin/locus.pl?locus=RBA50)*,* [*RCL1*](http://db.yeastgenome.org/cgi-bin/locus.pl?locus=RCL1)*,* [*RET1*](http://db.yeastgenome.org/cgi-bin/locus.pl?locus=RET1)*,* [*REX4*](http://db.yeastgenome.org/cgi-bin/locus.pl?locus=REX4)*,* [*RIO1*](http://db.yeastgenome.org/cgi-bin/locus.pl?locus=RIO1)*,* [*RIX1*](http://db.yeastgenome.org/cgi-bin/locus.pl?locus=RIX1)*,* [*RKI1*](http://db.yeastgenome.org/cgi-bin/locus.pl?locus=RKI1)*,* [*RLP7*](http://db.yeastgenome.org/cgi-bin/locus.pl?locus=RLP7)*,* [*RNH201*](http://db.yeastgenome.org/cgi-bin/locus.pl?locus=RNH201)*,* [*RNT1*](http://db.yeastgenome.org/cgi-bin/locus.pl?locus=RNT1)*,* [*ROK1*](http://db.yeastgenome.org/cgi-bin/locus.pl?locus=ROK1)*,* [*ROX3*](http://db.yeastgenome.org/cgi-bin/locus.pl?locus=ROX3)*,* [*RPA12*](http://db.yeastgenome.org/cgi-bin/locus.pl?locus=RPA12)*,* [*RPA190*](http://db.yeastgenome.org/cgi-bin/locus.pl?locus=RPA190)*,* [*RPA34*](http://db.yeastgenome.org/cgi-bin/locus.pl?locus=RPA34)*,* [*RPA43*](http://db.yeastgenome.org/cgi-bin/locus.pl?locus=RPA43)*,* [*RPA49*](http://db.yeastgenome.org/cgi-bin/locus.pl?locus=RPA49)*,* [*RPB10*](http://db.yeastgenome.org/cgi-bin/locus.pl?locus=RPB10)*,* [*RPB5*](http://db.yeastgenome.org/cgi-bin/locus.pl?locus=RPB5)*,* [*RPB8*](http://db.yeastgenome.org/cgi-bin/locus.pl?locus=RPB8)*,* [*RPB9*](http://db.yeastgenome.org/cgi-bin/locus.pl?locus=RPB9)*,* [*RPC11*](http://db.yeastgenome.org/cgi-bin/locus.pl?locus=RPC11)*,* [*RPC19*](http://db.yeastgenome.org/cgi-bin/locus.pl?locus=RPC19)*,* [*RPC31*](http://db.yeastgenome.org/cgi-bin/locus.pl?locus=RPC31)*,* [*RPC34*](http://db.yeastgenome.org/cgi-bin/locus.pl?locus=RPC34)*,* [*RPC37*](http://db.yeastgenome.org/cgi-bin/locus.pl?locus=RPC37)*,* [*RPC40*](http://db.yeastgenome.org/cgi-bin/locus.pl?locus=RPC40)*,* [*RPC53*](http://db.yeastgenome.org/cgi-bin/locus.pl?locus=RPC53)*,* [*RPC82*](http://db.yeastgenome.org/cgi-bin/locus.pl?locus=RPC82)*,* [*RPF1*](http://db.yeastgenome.org/cgi-bin/locus.pl?locus=RPF1)*,* [*RPF2*](http://db.yeastgenome.org/cgi-bin/locus.pl?locus=RPF2)*,* [*RPO26*](http://db.yeastgenome.org/cgi-bin/locus.pl?locus=RPO26)*,* [*RPP1*](http://db.yeastgenome.org/cgi-bin/locus.pl?locus=RPP1)*,* [*RPS14B*](http://db.yeastgenome.org/cgi-bin/locus.pl?locus=RPS14B)*,* [*RPS19B*](http://db.yeastgenome.org/cgi-bin/locus.pl?locus=RPS19B)*,* [*RRM3*](http://db.yeastgenome.org/cgi-bin/locus.pl?locus=RRM3)*,* [*RRN11*](http://db.yeastgenome.org/cgi-bin/locus.pl?locus=RRN11)*,* [*RRN7*](http://db.yeastgenome.org/cgi-bin/locus.pl?locus=RRN7)*,* [*RRP1*](http://db.yeastgenome.org/cgi-bin/locus.pl?locus=RRP1)*,* [*RRP12*](http://db.yeastgenome.org/cgi-bin/locus.pl?locus=RRP12)*,* [*RRP15*](http://db.yeastgenome.org/cgi-bin/locus.pl?locus=RRP15)*,* [*RRP17*](http://db.yeastgenome.org/cgi-bin/locus.pl?locus=RRP17)*,* [*RRP3*](http://db.yeastgenome.org/cgi-bin/locus.pl?locus=RRP3)*, RRP36,* [*RRP40*](http://db.yeastgenome.org/cgi-bin/locus.pl?locus=RRP40)*,* [*RRP43*](http://db.yeastgenome.org/cgi-bin/locus.pl?locus=RRP43)*,* [*RRP5*](http://db.yeastgenome.org/cgi-bin/locus.pl?locus=RRP5)*,* [*RRP6*](http://db.yeastgenome.org/cgi-bin/locus.pl?locus=RRP6)*,* [*RRP8*](http://db.yeastgenome.org/cgi-bin/locus.pl?locus=RRP8)*,* [*RRP9*](http://db.yeastgenome.org/cgi-bin/locus.pl?locus=RRP9)*,* [*RRS1*](http://db.yeastgenome.org/cgi-bin/locus.pl?locus=RRS1)*,* [*RVB1*](http://db.yeastgenome.org/cgi-bin/locus.pl?locus=RVB1)*,* [*SAN1*](http://db.yeastgenome.org/cgi-bin/locus.pl?locus=SAN1)*,* [*SAS10*](http://db.yeastgenome.org/cgi-bin/locus.pl?locus=SAS10)*,* [*SCC2*](http://db.yeastgenome.org/cgi-bin/locus.pl?locus=SCC2)*,* [*SEN34*](http://db.yeastgenome.org/cgi-bin/locus.pl?locus=SEN34)*,* [*SFG1*](http://db.yeastgenome.org/cgi-bin/locus.pl?locus=SFG1)*,* [*SHQ1*](http://db.yeastgenome.org/cgi-bin/locus.pl?locus=SHQ1)*,* [*SIK1*](http://db.yeastgenome.org/cgi-bin/locus.pl?locus=SIK1)*,* [*SIR2*](http://db.yeastgenome.org/cgi-bin/locus.pl?locus=SIR2)*,* [*SLX9*](http://db.yeastgenome.org/cgi-bin/locus.pl?locus=SLX9)*,* [*SNU13*](http://db.yeastgenome.org/cgi-bin/locus.pl?locus=SNU13)*,* [*SOF1*](http://db.yeastgenome.org/cgi-bin/locus.pl?locus=SOF1)*,* [*SPB1*](http://db.yeastgenome.org/cgi-bin/locus.pl?locus=SPB1)*,* [*SPB4*](http://db.yeastgenome.org/cgi-bin/locus.pl?locus=SPB4)*,* [*STE12*](http://db.yeastgenome.org/cgi-bin/locus.pl?locus=STE12)*,* [*SUT1*](http://db.yeastgenome.org/cgi-bin/locus.pl?locus=SUT1)*,* [*SUT2*](http://db.yeastgenome.org/cgi-bin/locus.pl?locus=SUT2)*,* [*SUV3*](http://db.yeastgenome.org/cgi-bin/locus.pl?locus=SUV3)*,* [*SWI5*](http://db.yeastgenome.org/cgi-bin/locus.pl?locus=SWI5)*,* [*TAD3*](http://db.yeastgenome.org/cgi-bin/locus.pl?locus=TAD3)*,* [*THP2*](http://db.yeastgenome.org/cgi-bin/locus.pl?locus=THP2)*,* [*TOP1*](http://db.yeastgenome.org/cgi-bin/locus.pl?locus=TOP1)*,* [*TOS4*](http://db.yeastgenome.org/cgi-bin/locus.pl?locus=TOS4)*,* [*TPA1*](http://db.yeastgenome.org/cgi-bin/locus.pl?locus=TPA1)*,* [*TRF5*](http://db.yeastgenome.org/cgi-bin/locus.pl?locus=TRF5)*,* [*TRM1*](http://db.yeastgenome.org/cgi-bin/locus.pl?locus=TRM1)*,* [*TRM10*](http://db.yeastgenome.org/cgi-bin/locus.pl?locus=TRM10)*,* [*TRM11*](http://db.yeastgenome.org/cgi-bin/locus.pl?locus=TRM11)*,* [*TRM112*](http://db.yeastgenome.org/cgi-bin/locus.pl?locus=TRM112)*,* [*TRM13*](http://db.yeastgenome.org/cgi-bin/locus.pl?locus=TRM13)*,* [*TRM2*](http://db.yeastgenome.org/cgi-bin/locus.pl?locus=TRM2)*,* [*TRM3*](http://db.yeastgenome.org/cgi-bin/locus.pl?locus=TRM3)*,* [*TRM7*](http://db.yeastgenome.org/cgi-bin/locus.pl?locus=TRM7)*,* [*TRM8*](http://db.yeastgenome.org/cgi-bin/locus.pl?locus=TRM8)*,* [*TRM82*](http://db.yeastgenome.org/cgi-bin/locus.pl?locus=TRM82)*,* [*TRM9*](http://db.yeastgenome.org/cgi-bin/locus.pl?locus=TRM9)*,* [*TSR1*](http://db.yeastgenome.org/cgi-bin/locus.pl?locus=TSR1)*,* [*TSR2*](http://db.yeastgenome.org/cgi-bin/locus.pl?locus=TSR2)*,* [*TYW1*](http://db.yeastgenome.org/cgi-bin/locus.pl?locus=TYW1)*,* [*UBP10*](http://db.yeastgenome.org/cgi-bin/locus.pl?locus=UBP10)*,* [*UNG1*](http://db.yeastgenome.org/cgi-bin/locus.pl?locus=UNG1)*,* [*URA4*](http://db.yeastgenome.org/cgi-bin/locus.pl?locus=URA4)*,* [*URA5*](http://db.yeastgenome.org/cgi-bin/locus.pl?locus=URA5)*,* [*URA7*](http://db.yeastgenome.org/cgi-bin/locus.pl?locus=URA7)*,* [*URB1*](http://db.yeastgenome.org/cgi-bin/locus.pl?locus=URB1)*,* [*URB2*](http://db.yeastgenome.org/cgi-bin/locus.pl?locus=URB2)*,* [*URK1*](http://db.yeastgenome.org/cgi-bin/locus.pl?locus=URK1)*,* [*UTP10*](http://db.yeastgenome.org/cgi-bin/locus.pl?locus=UTP10)*,* [*UTP11*](http://db.yeastgenome.org/cgi-bin/locus.pl?locus=UTP11)*,* [*UTP13*](http://db.yeastgenome.org/cgi-bin/locus.pl?locus=UTP13)*,* [*UTP14*](http://db.yeastgenome.org/cgi-bin/locus.pl?locus=UTP14)*,* [*UTP15*](http://db.yeastgenome.org/cgi-bin/locus.pl?locus=UTP15)*,* [*UTP18*](http://db.yeastgenome.org/cgi-bin/locus.pl?locus=UTP18)*,* [*UTP21*](http://db.yeastgenome.org/cgi-bin/locus.pl?locus=UTP21)*,* [*UTP22*](http://db.yeastgenome.org/cgi-bin/locus.pl?locus=UTP22)*,* [*UTP23*](http://db.yeastgenome.org/cgi-bin/locus.pl?locus=UTP23)*,* [*UTP30*](http://db.yeastgenome.org/cgi-bin/locus.pl?locus=UTP30)*,* [*UTP4*](http://db.yeastgenome.org/cgi-bin/locus.pl?locus=UTP4)*,* [*UTP5*](http://db.yeastgenome.org/cgi-bin/locus.pl?locus=UTP5)*,* [*UTP7*](http://db.yeastgenome.org/cgi-bin/locus.pl?locus=UTP7)*,* [*UTP8*](http://db.yeastgenome.org/cgi-bin/locus.pl?locus=UTP8)*,* [*UTP9*](http://db.yeastgenome.org/cgi-bin/locus.pl?locus=UTP9)*,* [*WRS1*](http://db.yeastgenome.org/cgi-bin/locus.pl?locus=WRS1)*,* [*YHR020W*](http://db.yeastgenome.org/cgi-bin/locus.pl?locus=YHR020W)*,* [*YNL247W*](http://db.yeastgenome.org/cgi-bin/locus.pl?locus=YNL247W)*,* [*YOX1*](http://db.yeastgenome.org/cgi-bin/locus.pl?locus=YOX1)*,* [*YRF1-6*](http://db.yeastgenome.org/cgi-bin/locus.pl?locus=YRF1-6) |
| 35S primary transcript processing  35S primary transcript processing (cont.) | 55 out of 558 genes, 9.9% | 1.47e-39 | [*BMS1*](http://db.yeastgenome.org/cgi-bin/locus.pl?locus=BMS1)*,* [*CBF5*](http://db.yeastgenome.org/cgi-bin/locus.pl?locus=CBF5)*,* [*DBP10*](http://db.yeastgenome.org/cgi-bin/locus.pl?locus=DBP10)*,* [*DBP2*](http://db.yeastgenome.org/cgi-bin/locus.pl?locus=DBP2)*,* [*DBP3*](http://db.yeastgenome.org/cgi-bin/locus.pl?locus=DBP3)*,* [*DBP6*](http://db.yeastgenome.org/cgi-bin/locus.pl?locus=DBP6)*,* [*DBP7*](http://db.yeastgenome.org/cgi-bin/locus.pl?locus=DBP7)*,* [*DBP8*](http://db.yeastgenome.org/cgi-bin/locus.pl?locus=DBP8)*,* [*DBP9*](http://db.yeastgenome.org/cgi-bin/locus.pl?locus=DBP9)*,* [*DIM1*](http://db.yeastgenome.org/cgi-bin/locus.pl?locus=DIM1)*,* [*DRS1*](http://db.yeastgenome.org/cgi-bin/locus.pl?locus=DRS1)*, EFG1,* [*EMG1*](http://db.yeastgenome.org/cgi-bin/locus.pl?locus=EMG1)*,* [*ENP1*](http://db.yeastgenome.org/cgi-bin/locus.pl?locus=ENP1)*,* [*ESF2*](http://db.yeastgenome.org/cgi-bin/locus.pl?locus=ESF2)*,* [*FAL1*](http://db.yeastgenome.org/cgi-bin/locus.pl?locus=FAL1)*,* [*FCF2*](http://db.yeastgenome.org/cgi-bin/locus.pl?locus=FCF2)*,* [*GAR1*](http://db.yeastgenome.org/cgi-bin/locus.pl?locus=GAR1)*,* [*HCA4*](http://db.yeastgenome.org/cgi-bin/locus.pl?locus=HCA4)*,* [*IMP3*](http://db.yeastgenome.org/cgi-bin/locus.pl?locus=IMP3)*,* [*IPI1*](http://db.yeastgenome.org/cgi-bin/locus.pl?locus=IPI1)*,* [*IPI3*](http://db.yeastgenome.org/cgi-bin/locus.pl?locus=IPI3)*,* [*KRR1*](http://db.yeastgenome.org/cgi-bin/locus.pl?locus=KRR1)*,* [*LCP5*](http://db.yeastgenome.org/cgi-bin/locus.pl?locus=LCP5)*,* [*MOT1*](http://db.yeastgenome.org/cgi-bin/locus.pl?locus=MOT1)*,* [*MPP10*](http://db.yeastgenome.org/cgi-bin/locus.pl?locus=MPP10)*,* [*MRD1*](http://db.yeastgenome.org/cgi-bin/locus.pl?locus=MRD1)*,* [*MTR3*](http://db.yeastgenome.org/cgi-bin/locus.pl?locus=MTR3)*,* [*NHP2*](http://db.yeastgenome.org/cgi-bin/locus.pl?locus=NHP2)*,* [*NOC4*](http://db.yeastgenome.org/cgi-bin/locus.pl?locus=NOC4)*,* [*NOG1*](http://db.yeastgenome.org/cgi-bin/locus.pl?locus=NOG1)*,* [*NOP14*](http://db.yeastgenome.org/cgi-bin/locus.pl?locus=NOP14)*,* [*NOP58*](http://db.yeastgenome.org/cgi-bin/locus.pl?locus=NOP58)*,* [*PNO1*](http://db.yeastgenome.org/cgi-bin/locus.pl?locus=PNO1)*,* [*PRP43*](http://db.yeastgenome.org/cgi-bin/locus.pl?locus=PRP43)*,* [*PWP2*](http://db.yeastgenome.org/cgi-bin/locus.pl?locus=PWP2)*,* [*PXR1*](http://db.yeastgenome.org/cgi-bin/locus.pl?locus=PXR1)*,* [*RCL1*](http://db.yeastgenome.org/cgi-bin/locus.pl?locus=RCL1)*,* [*RIX1*](http://db.yeastgenome.org/cgi-bin/locus.pl?locus=RIX1)*,* [*RNT1*](http://db.yeastgenome.org/cgi-bin/locus.pl?locus=RNT1)*,* [*ROK1*](http://db.yeastgenome.org/cgi-bin/locus.pl?locus=ROK1)*,* [*RRP3*](http://db.yeastgenome.org/cgi-bin/locus.pl?locus=RRP3)*,* [*RRP40*](http://db.yeastgenome.org/cgi-bin/locus.pl?locus=RRP40)*,* [*RRP43*](http://db.yeastgenome.org/cgi-bin/locus.pl?locus=RRP43)*,* [*RRP5*](http://db.yeastgenome.org/cgi-bin/locus.pl?locus=RRP5)*,* [*RRP6*](http://db.yeastgenome.org/cgi-bin/locus.pl?locus=RRP6)*,* [*RRP9*](http://db.yeastgenome.org/cgi-bin/locus.pl?locus=RRP9)*,* [*SIK1*](http://db.yeastgenome.org/cgi-bin/locus.pl?locus=SIK1)*,* [*SPB4*](http://db.yeastgenome.org/cgi-bin/locus.pl?locus=SPB4)*,* [*UTP18*](http://db.yeastgenome.org/cgi-bin/locus.pl?locus=UTP18)*,* [*UTP21*](http://db.yeastgenome.org/cgi-bin/locus.pl?locus=UTP21)*,* [*UTP22*](http://db.yeastgenome.org/cgi-bin/locus.pl?locus=UTP22)*,* [*UTP23*](http://db.yeastgenome.org/cgi-bin/locus.pl?locus=UTP23)*,* [*UTP30*](http://db.yeastgenome.org/cgi-bin/locus.pl?locus=UTP30)*,* [*UTP7*](http://db.yeastgenome.org/cgi-bin/locus.pl?locus=UTP7) |
| Processing of 20S pre-rRNA | 36 out of 558 genes, 6.5% | 2.02e-30 | [*BUD21*](http://db.yeastgenome.org/cgi-bin/locus.pl?locus=BUD21)*,* [*DIP2*](http://db.yeastgenome.org/cgi-bin/locus.pl?locus=DIP2)*,* [*ECM16*](http://db.yeastgenome.org/cgi-bin/locus.pl?locus=ECM16)*,* [*FAF1*](http://db.yeastgenome.org/cgi-bin/locus.pl?locus=FAF1)*,* [*FAP7*](http://db.yeastgenome.org/cgi-bin/locus.pl?locus=FAP7)*,* [*FYV7*](http://db.yeastgenome.org/cgi-bin/locus.pl?locus=FYV7)*,* [*IMP3*](http://db.yeastgenome.org/cgi-bin/locus.pl?locus=IMP3)*,* [*MPP10*](http://db.yeastgenome.org/cgi-bin/locus.pl?locus=MPP10)*,* [*NAN1*](http://db.yeastgenome.org/cgi-bin/locus.pl?locus=NAN1)*,* [*NOB1*](http://db.yeastgenome.org/cgi-bin/locus.pl?locus=NOB1)*,* [*NOP14*](http://db.yeastgenome.org/cgi-bin/locus.pl?locus=NOP14)*,* [*NOP58*](http://db.yeastgenome.org/cgi-bin/locus.pl?locus=NOP58)*,* [*NOP7*](http://db.yeastgenome.org/cgi-bin/locus.pl?locus=NOP7)*,* [*PRP43*](http://db.yeastgenome.org/cgi-bin/locus.pl?locus=PRP43)*,* [*PWP2*](http://db.yeastgenome.org/cgi-bin/locus.pl?locus=PWP2)*,* [*RIO1*](http://db.yeastgenome.org/cgi-bin/locus.pl?locus=RIO1)*,* [*RPS14B*](http://db.yeastgenome.org/cgi-bin/locus.pl?locus=RPS14B)*,* [*RRP12*](http://db.yeastgenome.org/cgi-bin/locus.pl?locus=RRP12)*, RRP36,* [*RRP5*](http://db.yeastgenome.org/cgi-bin/locus.pl?locus=RRP5)*,* [*RRP9*](http://db.yeastgenome.org/cgi-bin/locus.pl?locus=RRP9)*,* [*SAS10*](http://db.yeastgenome.org/cgi-bin/locus.pl?locus=SAS10)*,* [*SIK1*](http://db.yeastgenome.org/cgi-bin/locus.pl?locus=SIK1)*,* [*SNU13*](http://db.yeastgenome.org/cgi-bin/locus.pl?locus=SNU13)*,* [*SOF1*](http://db.yeastgenome.org/cgi-bin/locus.pl?locus=SOF1)*,* [*TSR2*](http://db.yeastgenome.org/cgi-bin/locus.pl?locus=TSR2)*,* [*UTP10*](http://db.yeastgenome.org/cgi-bin/locus.pl?locus=UTP10)*,* [*UTP11*](http://db.yeastgenome.org/cgi-bin/locus.pl?locus=UTP11)*,* [*UTP13*](http://db.yeastgenome.org/cgi-bin/locus.pl?locus=UTP13)*,* [*UTP14*](http://db.yeastgenome.org/cgi-bin/locus.pl?locus=UTP14)*,* [*UTP15*](http://db.yeastgenome.org/cgi-bin/locus.pl?locus=UTP15)*,* [*UTP23*](http://db.yeastgenome.org/cgi-bin/locus.pl?locus=UTP23)*,* [*UTP4*](http://db.yeastgenome.org/cgi-bin/locus.pl?locus=UTP4)*,* [*UTP5*](http://db.yeastgenome.org/cgi-bin/locus.pl?locus=UTP5)*,* [*UTP8*](http://db.yeastgenome.org/cgi-bin/locus.pl?locus=UTP8)*,* [*UTP9*](http://db.yeastgenome.org/cgi-bin/locus.pl?locus=UTP9) |
| Ribosomal large subunit biogenesis and assembly | 42 out of 558 genes, 7.5% | 4.53e-29 | [*ALB1*](http://db.yeastgenome.org/cgi-bin/locus.pl?locus=ALB1)*,* [*ARX1*](http://db.yeastgenome.org/cgi-bin/locus.pl?locus=ARX1)*,* [*BRX1*](http://db.yeastgenome.org/cgi-bin/locus.pl?locus=BRX1)*,* [*CIC1*](http://db.yeastgenome.org/cgi-bin/locus.pl?locus=CIC1)*,* [*DBP10*](http://db.yeastgenome.org/cgi-bin/locus.pl?locus=DBP10)*,* [*DBP3*](http://db.yeastgenome.org/cgi-bin/locus.pl?locus=DBP3)*,* [*DBP6*](http://db.yeastgenome.org/cgi-bin/locus.pl?locus=DBP6)*,* [*DBP7*](http://db.yeastgenome.org/cgi-bin/locus.pl?locus=DBP7)*,* [*DBP9*](http://db.yeastgenome.org/cgi-bin/locus.pl?locus=DBP9)*,* [*DRS1*](http://db.yeastgenome.org/cgi-bin/locus.pl?locus=DRS1)*,* [*IPI1*](http://db.yeastgenome.org/cgi-bin/locus.pl?locus=IPI1)*,* [*IPI3*](http://db.yeastgenome.org/cgi-bin/locus.pl?locus=IPI3)*,* [*LOC1*](http://db.yeastgenome.org/cgi-bin/locus.pl?locus=LOC1)*,* [*MAK11*](http://db.yeastgenome.org/cgi-bin/locus.pl?locus=MAK11)*,* [*MAK16*](http://db.yeastgenome.org/cgi-bin/locus.pl?locus=MAK16)*,* [*MAK21*](http://db.yeastgenome.org/cgi-bin/locus.pl?locus=MAK21)*,* [*MRT4*](http://db.yeastgenome.org/cgi-bin/locus.pl?locus=MRT4)*,* [*NIP7*](http://db.yeastgenome.org/cgi-bin/locus.pl?locus=NIP7)*,* [*NMD3*](http://db.yeastgenome.org/cgi-bin/locus.pl?locus=NMD3)*,* [*NOG1*](http://db.yeastgenome.org/cgi-bin/locus.pl?locus=NOG1)*,* [*NOP15*](http://db.yeastgenome.org/cgi-bin/locus.pl?locus=NOP15)*,* [*NOP16*](http://db.yeastgenome.org/cgi-bin/locus.pl?locus=NOP16)*,* [*NOP7*](http://db.yeastgenome.org/cgi-bin/locus.pl?locus=NOP7)*,* [*NOP8*](http://db.yeastgenome.org/cgi-bin/locus.pl?locus=NOP8)*,* [*NSA1*](http://db.yeastgenome.org/cgi-bin/locus.pl?locus=NSA1)*,* [*PRP43*](http://db.yeastgenome.org/cgi-bin/locus.pl?locus=PRP43)*,* [*REI1*](http://db.yeastgenome.org/cgi-bin/locus.pl?locus=REI1)*,* [*RIX1*](http://db.yeastgenome.org/cgi-bin/locus.pl?locus=RIX1)*,* [*RLI1*](http://db.yeastgenome.org/cgi-bin/locus.pl?locus=RLI1)*,* [*RLP24*](http://db.yeastgenome.org/cgi-bin/locus.pl?locus=RLP24)*,* [*RLP7*](http://db.yeastgenome.org/cgi-bin/locus.pl?locus=RLP7)*,* [*RPF1*](http://db.yeastgenome.org/cgi-bin/locus.pl?locus=RPF1)*,* [*RPF2*](http://db.yeastgenome.org/cgi-bin/locus.pl?locus=RPF2)*,* [*RRP14*](http://db.yeastgenome.org/cgi-bin/locus.pl?locus=RRP14)*,* [*RSA1*](http://db.yeastgenome.org/cgi-bin/locus.pl?locus=RSA1)*,* [*RSA3*](http://db.yeastgenome.org/cgi-bin/locus.pl?locus=RSA3)*,* [*RSA4*](http://db.yeastgenome.org/cgi-bin/locus.pl?locus=RSA4)*,* [*SPB4*](http://db.yeastgenome.org/cgi-bin/locus.pl?locus=SPB4)*,* [*SQT1*](http://db.yeastgenome.org/cgi-bin/locus.pl?locus=SQT1)*,* [*SSF1*](http://db.yeastgenome.org/cgi-bin/locus.pl?locus=SSF1)*,* [*SSF2*](http://db.yeastgenome.org/cgi-bin/locus.pl?locus=SSF2)*,* [*YTM1*](http://db.yeastgenome.org/cgi-bin/locus.pl?locus=YTM1) |
| Cellular process  Cellular process (cont.) | 477 out of 558 genes, 85.5% | 2.76e-28 | [*AAH1*](http://db.yeastgenome.org/cgi-bin/locus.pl?locus=AAH1)*,* [*ADE17*](http://db.yeastgenome.org/cgi-bin/locus.pl?locus=ADE17)*,* [*ADE5,7*](http://db.yeastgenome.org/cgi-bin/locus.pl?locus=ADE5,7)*,* [*ADE6*](http://db.yeastgenome.org/cgi-bin/locus.pl?locus=ADE6)*,* [*ADE8*](http://db.yeastgenome.org/cgi-bin/locus.pl?locus=ADE8)*,* [*AIR1*](http://db.yeastgenome.org/cgi-bin/locus.pl?locus=AIR1)*,* [*ALB1*](http://db.yeastgenome.org/cgi-bin/locus.pl?locus=ALB1)*,* [*ALK1*](http://db.yeastgenome.org/cgi-bin/locus.pl?locus=ALK1)*,* [*APT1*](http://db.yeastgenome.org/cgi-bin/locus.pl?locus=APT1)*,* [*ARB1*](http://db.yeastgenome.org/cgi-bin/locus.pl?locus=ARB1)*,* [*ARP1*](http://db.yeastgenome.org/cgi-bin/locus.pl?locus=ARP1)*,* [*ARX1*](http://db.yeastgenome.org/cgi-bin/locus.pl?locus=ARX1)*,* [*ASC1*](http://db.yeastgenome.org/cgi-bin/locus.pl?locus=ASC1)*,* [*ASH1*](http://db.yeastgenome.org/cgi-bin/locus.pl?locus=ASH1)*,* [*ASP1*](http://db.yeastgenome.org/cgi-bin/locus.pl?locus=ASP1)*,* [*AST1*](http://db.yeastgenome.org/cgi-bin/locus.pl?locus=AST1)*,* [*ATC1*](http://db.yeastgenome.org/cgi-bin/locus.pl?locus=ATC1)*,* [*ATF2*](http://db.yeastgenome.org/cgi-bin/locus.pl?locus=ATF2)*,* [*AUR1*](http://db.yeastgenome.org/cgi-bin/locus.pl?locus=AUR1)*,* [*AVL9*](http://db.yeastgenome.org/cgi-bin/locus.pl?locus=AVL9)*,* [*BCD1*](http://db.yeastgenome.org/cgi-bin/locus.pl?locus=BCD1)*,* [*BCP1*](http://db.yeastgenome.org/cgi-bin/locus.pl?locus=BCP1)*,* [*BFR2*](http://db.yeastgenome.org/cgi-bin/locus.pl?locus=BFR2)*,* [*BIO2*](http://db.yeastgenome.org/cgi-bin/locus.pl?locus=BIO2)*,* [*BMS1*](http://db.yeastgenome.org/cgi-bin/locus.pl?locus=BMS1)*,* [*BRR6*](http://db.yeastgenome.org/cgi-bin/locus.pl?locus=BRR6)*,* [*BRX1*](http://db.yeastgenome.org/cgi-bin/locus.pl?locus=BRX1)*,* [*BST1*](http://db.yeastgenome.org/cgi-bin/locus.pl?locus=BST1)*,* [*BUD17*](http://db.yeastgenome.org/cgi-bin/locus.pl?locus=BUD17)*,* [*BUD21*](http://db.yeastgenome.org/cgi-bin/locus.pl?locus=BUD21)*,* [*BUD22*](http://db.yeastgenome.org/cgi-bin/locus.pl?locus=BUD22)*,* [*BUD23*](http://db.yeastgenome.org/cgi-bin/locus.pl?locus=BUD23)*,* [*BUD27*](http://db.yeastgenome.org/cgi-bin/locus.pl?locus=BUD27)*,* [*BUD32*](http://db.yeastgenome.org/cgi-bin/locus.pl?locus=BUD32)*,* [*BUD6*](http://db.yeastgenome.org/cgi-bin/locus.pl?locus=BUD6)*,* [*BUD8*](http://db.yeastgenome.org/cgi-bin/locus.pl?locus=BUD8)*,* [*CBF5*](http://db.yeastgenome.org/cgi-bin/locus.pl?locus=CBF5)*,* [*CDC14*](http://db.yeastgenome.org/cgi-bin/locus.pl?locus=CDC14)*,* [*CDC20*](http://db.yeastgenome.org/cgi-bin/locus.pl?locus=CDC20)*,* [*CDC47*](http://db.yeastgenome.org/cgi-bin/locus.pl?locus=CDC47)*,* [*CGR1*](http://db.yeastgenome.org/cgi-bin/locus.pl?locus=CGR1)*,* [*CIC1*](http://db.yeastgenome.org/cgi-bin/locus.pl?locus=CIC1)*,* [*CIN4*](http://db.yeastgenome.org/cgi-bin/locus.pl?locus=CIN4)*,* [*CLA4*](http://db.yeastgenome.org/cgi-bin/locus.pl?locus=CLA4)*,* [*CLB1*](http://db.yeastgenome.org/cgi-bin/locus.pl?locus=CLB1)*,* [*CLB3*](http://db.yeastgenome.org/cgi-bin/locus.pl?locus=CLB3)*,* [*CLN1*](http://db.yeastgenome.org/cgi-bin/locus.pl?locus=CLN1)*,* [*CLN2*](http://db.yeastgenome.org/cgi-bin/locus.pl?locus=CLN2)*, CMS1,* [*CSM3*](http://db.yeastgenome.org/cgi-bin/locus.pl?locus=CSM3)*,* [*CUS1*](http://db.yeastgenome.org/cgi-bin/locus.pl?locus=CUS1)*,* [*CWC2*](http://db.yeastgenome.org/cgi-bin/locus.pl?locus=CWC2)*,* [*CYB5*](http://db.yeastgenome.org/cgi-bin/locus.pl?locus=CYB5)*,* [*DBP10*](http://db.yeastgenome.org/cgi-bin/locus.pl?locus=DBP10)*,* [*DBP2*](http://db.yeastgenome.org/cgi-bin/locus.pl?locus=DBP2)*,* [*DBP3*](http://db.yeastgenome.org/cgi-bin/locus.pl?locus=DBP3)*,* [*DBP6*](http://db.yeastgenome.org/cgi-bin/locus.pl?locus=DBP6)*,* [*DBP7*](http://db.yeastgenome.org/cgi-bin/locus.pl?locus=DBP7)*,* [*DBP8*](http://db.yeastgenome.org/cgi-bin/locus.pl?locus=DBP8)*,* [*DBP9*](http://db.yeastgenome.org/cgi-bin/locus.pl?locus=DBP9)*,* [*DFR1*](http://db.yeastgenome.org/cgi-bin/locus.pl?locus=DFR1)*,* [*DHR2*](http://db.yeastgenome.org/cgi-bin/locus.pl?locus=DHR2)*,* [*DIM1*](http://db.yeastgenome.org/cgi-bin/locus.pl?locus=DIM1)*,* [*DIP2*](http://db.yeastgenome.org/cgi-bin/locus.pl?locus=DIP2)*,* [*DPB2*](http://db.yeastgenome.org/cgi-bin/locus.pl?locus=DPB2)*,* [*DPH2*](http://db.yeastgenome.org/cgi-bin/locus.pl?locus=DPH2)*,* [*DRS1*](http://db.yeastgenome.org/cgi-bin/locus.pl?locus=DRS1)*,* [*DSE1*](http://db.yeastgenome.org/cgi-bin/locus.pl?locus=DSE1)*,* [*DSE2*](http://db.yeastgenome.org/cgi-bin/locus.pl?locus=DSE2)*,* [*DSE4*](http://db.yeastgenome.org/cgi-bin/locus.pl?locus=DSE4)*,* [*DUO1*](http://db.yeastgenome.org/cgi-bin/locus.pl?locus=DUO1)*,* [*DUS1*](http://db.yeastgenome.org/cgi-bin/locus.pl?locus=DUS1)*,* [*DUS3*](http://db.yeastgenome.org/cgi-bin/locus.pl?locus=DUS3)*,* [*DUS4*](http://db.yeastgenome.org/cgi-bin/locus.pl?locus=DUS4)*,* [*DUT1*](http://db.yeastgenome.org/cgi-bin/locus.pl?locus=DUT1)*,* [*DYS1*](http://db.yeastgenome.org/cgi-bin/locus.pl?locus=DYS1)*,* [*EBP2*](http://db.yeastgenome.org/cgi-bin/locus.pl?locus=EBP2)*,* [*ECM16*](http://db.yeastgenome.org/cgi-bin/locus.pl?locus=ECM16)*,* [*EFB1*](http://db.yeastgenome.org/cgi-bin/locus.pl?locus=EFB1)*, EFG1,* [*EGT2*](http://db.yeastgenome.org/cgi-bin/locus.pl?locus=EGT2)*,* [*EKI1*](http://db.yeastgenome.org/cgi-bin/locus.pl?locus=EKI1)*,* [*ELP2*](http://db.yeastgenome.org/cgi-bin/locus.pl?locus=ELP2)*,* [*ELP3*](http://db.yeastgenome.org/cgi-bin/locus.pl?locus=ELP3)*,* [*EMG1*](http://db.yeastgenome.org/cgi-bin/locus.pl?locus=EMG1)*,* [*ENP1*](http://db.yeastgenome.org/cgi-bin/locus.pl?locus=ENP1)*,* [*ENP2*](http://db.yeastgenome.org/cgi-bin/locus.pl?locus=ENP2)*,* [*ERB1*](http://db.yeastgenome.org/cgi-bin/locus.pl?locus=ERB1)*,* [*ERG1*](http://db.yeastgenome.org/cgi-bin/locus.pl?locus=ERG1)*,* [*ERG11*](http://db.yeastgenome.org/cgi-bin/locus.pl?locus=ERG11)*,* [*ERG3*](http://db.yeastgenome.org/cgi-bin/locus.pl?locus=ERG3)*,* [*ERG5*](http://db.yeastgenome.org/cgi-bin/locus.pl?locus=ERG5)*,* [*ESF1*](http://db.yeastgenome.org/cgi-bin/locus.pl?locus=ESF1)*,* [*ESF2*](http://db.yeastgenome.org/cgi-bin/locus.pl?locus=ESF2)*,* [*ESP1*](http://db.yeastgenome.org/cgi-bin/locus.pl?locus=ESP1)*,* [*EXG1*](http://db.yeastgenome.org/cgi-bin/locus.pl?locus=EXG1)*,* [*FAA4*](http://db.yeastgenome.org/cgi-bin/locus.pl?locus=FAA4)*,* [*FAF1*](http://db.yeastgenome.org/cgi-bin/locus.pl?locus=FAF1)*,* [*FAL1*](http://db.yeastgenome.org/cgi-bin/locus.pl?locus=FAL1)*,* [*FAP7*](http://db.yeastgenome.org/cgi-bin/locus.pl?locus=FAP7)*,* [*FCF2*](http://db.yeastgenome.org/cgi-bin/locus.pl?locus=FCF2)*,* [*FEN1*](http://db.yeastgenome.org/cgi-bin/locus.pl?locus=FEN1)*,* [*FET4*](http://db.yeastgenome.org/cgi-bin/locus.pl?locus=FET4)*,* [*FIG2*](http://db.yeastgenome.org/cgi-bin/locus.pl?locus=FIG2)*,* [*FKH1*](http://db.yeastgenome.org/cgi-bin/locus.pl?locus=FKH1)*,* [*FOL1*](http://db.yeastgenome.org/cgi-bin/locus.pl?locus=FOL1)*,* [*FPR4*](http://db.yeastgenome.org/cgi-bin/locus.pl?locus=FPR4)*,* [*FUR1*](http://db.yeastgenome.org/cgi-bin/locus.pl?locus=FUR1)*,* [*FYV7*](http://db.yeastgenome.org/cgi-bin/locus.pl?locus=FYV7)*,* [*GAR1*](http://db.yeastgenome.org/cgi-bin/locus.pl?locus=GAR1)*,* [*GCD10*](http://db.yeastgenome.org/cgi-bin/locus.pl?locus=GCD10)*,* [*GCD11*](http://db.yeastgenome.org/cgi-bin/locus.pl?locus=GCD11)*,* [*GCD14*](http://db.yeastgenome.org/cgi-bin/locus.pl?locus=GCD14)*,* [*GCD2*](http://db.yeastgenome.org/cgi-bin/locus.pl?locus=GCD2)*,* [*GCN3*](http://db.yeastgenome.org/cgi-bin/locus.pl?locus=GCN3)*,* [*GCR1*](http://db.yeastgenome.org/cgi-bin/locus.pl?locus=GCR1)*,* [*GCR2*](http://db.yeastgenome.org/cgi-bin/locus.pl?locus=GCR2)*,* [*GDT1*](http://db.yeastgenome.org/cgi-bin/locus.pl?locus=GDT1)*,* [*GEA1*](http://db.yeastgenome.org/cgi-bin/locus.pl?locus=GEA1)*,* [*GLE2*](http://db.yeastgenome.org/cgi-bin/locus.pl?locus=GLE2)*,* [*GLN1*](http://db.yeastgenome.org/cgi-bin/locus.pl?locus=GLN1)*,* [*GLN4*](http://db.yeastgenome.org/cgi-bin/locus.pl?locus=GLN4)*,* [*GPI13*](http://db.yeastgenome.org/cgi-bin/locus.pl?locus=GPI13)*,* [*GRC3*](http://db.yeastgenome.org/cgi-bin/locus.pl?locus=GRC3)*,* [*GUA1*](http://db.yeastgenome.org/cgi-bin/locus.pl?locus=GUA1)*,* [*HAS1*](http://db.yeastgenome.org/cgi-bin/locus.pl?locus=HAS1)*,* [*HCA4*](http://db.yeastgenome.org/cgi-bin/locus.pl?locus=HCA4)*,* [*HEM1*](http://db.yeastgenome.org/cgi-bin/locus.pl?locus=HEM1)*,* [*HGH1*](http://db.yeastgenome.org/cgi-bin/locus.pl?locus=HGH1)*,* [*HHF2*](http://db.yeastgenome.org/cgi-bin/locus.pl?locus=HHF2)*,* [*HHT1*](http://db.yeastgenome.org/cgi-bin/locus.pl?locus=HHT1)*,* [*HIR2*](http://db.yeastgenome.org/cgi-bin/locus.pl?locus=HIR2)*,* [*HLR1*](http://db.yeastgenome.org/cgi-bin/locus.pl?locus=HLR1)*,* [*HMG1*](http://db.yeastgenome.org/cgi-bin/locus.pl?locus=HMG1)*,* [*HMS2*](http://db.yeastgenome.org/cgi-bin/locus.pl?locus=HMS2)*,* [*HMT1*](http://db.yeastgenome.org/cgi-bin/locus.pl?locus=HMT1)*,* [*HOF1*](http://db.yeastgenome.org/cgi-bin/locus.pl?locus=HOF1)*,* [*HPT1*](http://db.yeastgenome.org/cgi-bin/locus.pl?locus=HPT1)*,* [*HST3*](http://db.yeastgenome.org/cgi-bin/locus.pl?locus=HST3)*,* [*HTA1*](http://db.yeastgenome.org/cgi-bin/locus.pl?locus=HTA1)*,* [*HTA2*](http://db.yeastgenome.org/cgi-bin/locus.pl?locus=HTA2)*,* [*HTB1*](http://db.yeastgenome.org/cgi-bin/locus.pl?locus=HTB1)*,* [*HTB2*](http://db.yeastgenome.org/cgi-bin/locus.pl?locus=HTB2)*,* [*HTZ1*](http://db.yeastgenome.org/cgi-bin/locus.pl?locus=HTZ1)*,* [*IFH1*](http://db.yeastgenome.org/cgi-bin/locus.pl?locus=IFH1)*,* [*ILV3*](http://db.yeastgenome.org/cgi-bin/locus.pl?locus=ILV3)*,* [*ILV5*](http://db.yeastgenome.org/cgi-bin/locus.pl?locus=ILV5)*,* [*IMD3*](http://db.yeastgenome.org/cgi-bin/locus.pl?locus=IMD3)*,* [*IMP3*](http://db.yeastgenome.org/cgi-bin/locus.pl?locus=IMP3)*,* [*INO2*](http://db.yeastgenome.org/cgi-bin/locus.pl?locus=INO2)*,* [*IPI1*](http://db.yeastgenome.org/cgi-bin/locus.pl?locus=IPI1)*,* [*IPI3*](http://db.yeastgenome.org/cgi-bin/locus.pl?locus=IPI3)*,* [*IZH1*](http://db.yeastgenome.org/cgi-bin/locus.pl?locus=IZH1)*,* [*JJJ3*](http://db.yeastgenome.org/cgi-bin/locus.pl?locus=JJJ3)*,* [*KAE1*](http://db.yeastgenome.org/cgi-bin/locus.pl?locus=KAE1)*,* [*KAP123*](http://db.yeastgenome.org/cgi-bin/locus.pl?locus=KAP123)*,* [*KCS1*](http://db.yeastgenome.org/cgi-bin/locus.pl?locus=KCS1)*,* [*KIN4*](http://db.yeastgenome.org/cgi-bin/locus.pl?locus=KIN4)*,* [*KRE33*](http://db.yeastgenome.org/cgi-bin/locus.pl?locus=KRE33)*,* [*KRI1*](http://db.yeastgenome.org/cgi-bin/locus.pl?locus=KRI1)*,* [*KRR1*](http://db.yeastgenome.org/cgi-bin/locus.pl?locus=KRR1)*,* [*KTI12*](http://db.yeastgenome.org/cgi-bin/locus.pl?locus=KTI12)*,* [*KTR5*](http://db.yeastgenome.org/cgi-bin/locus.pl?locus=KTR5)*,* [*LCP5*](http://db.yeastgenome.org/cgi-bin/locus.pl?locus=LCP5)*,* [*LEU9*](http://db.yeastgenome.org/cgi-bin/locus.pl?locus=LEU9)*,* [*LIA1*](http://db.yeastgenome.org/cgi-bin/locus.pl?locus=LIA1)*,* [*LOC1*](http://db.yeastgenome.org/cgi-bin/locus.pl?locus=LOC1)*,* [*LSG1*](http://db.yeastgenome.org/cgi-bin/locus.pl?locus=LSG1)*,* [*LYS12*](http://db.yeastgenome.org/cgi-bin/locus.pl?locus=LYS12)*,* [*LYS4*](http://db.yeastgenome.org/cgi-bin/locus.pl?locus=LYS4)*,* [*MAE1*](http://db.yeastgenome.org/cgi-bin/locus.pl?locus=MAE1)*,* [*MAK11*](http://db.yeastgenome.org/cgi-bin/locus.pl?locus=MAK11)*,* [*MAK16*](http://db.yeastgenome.org/cgi-bin/locus.pl?locus=MAK16)*,* [*MAK21*](http://db.yeastgenome.org/cgi-bin/locus.pl?locus=MAK21)*,* [*MAK3*](http://db.yeastgenome.org/cgi-bin/locus.pl?locus=MAK3)*,* [*MAP1*](http://db.yeastgenome.org/cgi-bin/locus.pl?locus=MAP1)*,* [*MES1*](http://db.yeastgenome.org/cgi-bin/locus.pl?locus=MES1)*,* [*MEU1*](http://db.yeastgenome.org/cgi-bin/locus.pl?locus=MEU1)*,* [*MIS1*](http://db.yeastgenome.org/cgi-bin/locus.pl?locus=MIS1)*,* [*MNN2*](http://db.yeastgenome.org/cgi-bin/locus.pl?locus=MNN2)*,* [*MOT1*](http://db.yeastgenome.org/cgi-bin/locus.pl?locus=MOT1)*,* [*MPP10*](http://db.yeastgenome.org/cgi-bin/locus.pl?locus=MPP10)*,* [*MRD1*](http://db.yeastgenome.org/cgi-bin/locus.pl?locus=MRD1)*,* [*MRT4*](http://db.yeastgenome.org/cgi-bin/locus.pl?locus=MRT4)*,* [*MSB1*](http://db.yeastgenome.org/cgi-bin/locus.pl?locus=MSB1)*,* [*MSH1*](http://db.yeastgenome.org/cgi-bin/locus.pl?locus=MSH1)*,* [*MSH6*](http://db.yeastgenome.org/cgi-bin/locus.pl?locus=MSH6)*,* [*MTD1*](http://db.yeastgenome.org/cgi-bin/locus.pl?locus=MTD1)*,* [*MTR3*](http://db.yeastgenome.org/cgi-bin/locus.pl?locus=MTR3)*,* [*NAF1*](http://db.yeastgenome.org/cgi-bin/locus.pl?locus=NAF1)*,* [*NAN1*](http://db.yeastgenome.org/cgi-bin/locus.pl?locus=NAN1)*,* [*NCL1*](http://db.yeastgenome.org/cgi-bin/locus.pl?locus=NCL1)*,* [*NCS2*](http://db.yeastgenome.org/cgi-bin/locus.pl?locus=NCS2)*,* [*NEW1*](http://db.yeastgenome.org/cgi-bin/locus.pl?locus=NEW1)*,* [*NHP2*](http://db.yeastgenome.org/cgi-bin/locus.pl?locus=NHP2)*,* [*NIP7*](http://db.yeastgenome.org/cgi-bin/locus.pl?locus=NIP7)*,* [*NMD3*](http://db.yeastgenome.org/cgi-bin/locus.pl?locus=NMD3)*,* [*NNF1*](http://db.yeastgenome.org/cgi-bin/locus.pl?locus=NNF1)*,* [*NOB1*](http://db.yeastgenome.org/cgi-bin/locus.pl?locus=NOB1)*,* [*NOC2*](http://db.yeastgenome.org/cgi-bin/locus.pl?locus=NOC2)*,* [*NOC3*](http://db.yeastgenome.org/cgi-bin/locus.pl?locus=NOC3)*,* [*NOC4*](http://db.yeastgenome.org/cgi-bin/locus.pl?locus=NOC4)*,* [*NOG1*](http://db.yeastgenome.org/cgi-bin/locus.pl?locus=NOG1)*,* [*NOG2*](http://db.yeastgenome.org/cgi-bin/locus.pl?locus=NOG2)*,* [*NOP12*](http://db.yeastgenome.org/cgi-bin/locus.pl?locus=NOP12)*,* [*NOP13*](http://db.yeastgenome.org/cgi-bin/locus.pl?locus=NOP13)*,* [*NOP14*](http://db.yeastgenome.org/cgi-bin/locus.pl?locus=NOP14)*,* [*NOP15*](http://db.yeastgenome.org/cgi-bin/locus.pl?locus=NOP15)*,* [*NOP16*](http://db.yeastgenome.org/cgi-bin/locus.pl?locus=NOP16)*,* [*NOP2*](http://db.yeastgenome.org/cgi-bin/locus.pl?locus=NOP2)*,* [*NOP4*](http://db.yeastgenome.org/cgi-bin/locus.pl?locus=NOP4)*,* [*NOP53*](http://db.yeastgenome.org/cgi-bin/locus.pl?locus=NOP53)*,* [*NOP58*](http://db.yeastgenome.org/cgi-bin/locus.pl?locus=NOP58)*,* [*NOP6*](http://db.yeastgenome.org/cgi-bin/locus.pl?locus=NOP6)*,* [*NOP7*](http://db.yeastgenome.org/cgi-bin/locus.pl?locus=NOP7)*,* [*NOP8*](http://db.yeastgenome.org/cgi-bin/locus.pl?locus=NOP8)*,* [*NOP9*](http://db.yeastgenome.org/cgi-bin/locus.pl?locus=NOP9)*,* [*NRP1*](http://db.yeastgenome.org/cgi-bin/locus.pl?locus=NRP1)*,* [*NSA1*](http://db.yeastgenome.org/cgi-bin/locus.pl?locus=NSA1)*,* [*NSE4*](http://db.yeastgenome.org/cgi-bin/locus.pl?locus=NSE4)*,* [*NSR1*](http://db.yeastgenome.org/cgi-bin/locus.pl?locus=NSR1)*,* [*NUC1*](http://db.yeastgenome.org/cgi-bin/locus.pl?locus=NUC1)*,* [*NUG1*](http://db.yeastgenome.org/cgi-bin/locus.pl?locus=NUG1)*,* [*NUP1*](http://db.yeastgenome.org/cgi-bin/locus.pl?locus=NUP1)*,* [*ORC1*](http://db.yeastgenome.org/cgi-bin/locus.pl?locus=ORC1)*,* [*PHO11*](http://db.yeastgenome.org/cgi-bin/locus.pl?locus=PHO11)*,* [*PHO3*](http://db.yeastgenome.org/cgi-bin/locus.pl?locus=PHO3)*,* [*PHO5*](http://db.yeastgenome.org/cgi-bin/locus.pl?locus=PHO5)*,* [*PLB2*](http://db.yeastgenome.org/cgi-bin/locus.pl?locus=PLB2)*,* [*PMT2*](http://db.yeastgenome.org/cgi-bin/locus.pl?locus=PMT2)*,* [*PNO1*](http://db.yeastgenome.org/cgi-bin/locus.pl?locus=PNO1)*,* [*POL5*](http://db.yeastgenome.org/cgi-bin/locus.pl?locus=POL5)*,* [*POP3*](http://db.yeastgenome.org/cgi-bin/locus.pl?locus=POP3)*,* [*POP6*](http://db.yeastgenome.org/cgi-bin/locus.pl?locus=POP6)*,* [*POP8*](http://db.yeastgenome.org/cgi-bin/locus.pl?locus=POP8)*,* [*PPT1*](http://db.yeastgenome.org/cgi-bin/locus.pl?locus=PPT1)*,* [*PRO1*](http://db.yeastgenome.org/cgi-bin/locus.pl?locus=PRO1)*,* [*PRP19*](http://db.yeastgenome.org/cgi-bin/locus.pl?locus=PRP19)*,* [*PRP24*](http://db.yeastgenome.org/cgi-bin/locus.pl?locus=PRP24)*,* [*PRP43*](http://db.yeastgenome.org/cgi-bin/locus.pl?locus=PRP43)*,* [*PRS1*](http://db.yeastgenome.org/cgi-bin/locus.pl?locus=PRS1)*,* [*PRS2*](http://db.yeastgenome.org/cgi-bin/locus.pl?locus=PRS2)*,* [*PRS4*](http://db.yeastgenome.org/cgi-bin/locus.pl?locus=PRS4)*,* [*PSY4*](http://db.yeastgenome.org/cgi-bin/locus.pl?locus=PSY4)*,* [*PUF6*](http://db.yeastgenome.org/cgi-bin/locus.pl?locus=PUF6)*,* [*PUS1*](http://db.yeastgenome.org/cgi-bin/locus.pl?locus=PUS1)*,* [*PUS4*](http://db.yeastgenome.org/cgi-bin/locus.pl?locus=PUS4)*,* [*PUS7*](http://db.yeastgenome.org/cgi-bin/locus.pl?locus=PUS7)*,* [*PWP1*](http://db.yeastgenome.org/cgi-bin/locus.pl?locus=PWP1)*,* [*PWP2*](http://db.yeastgenome.org/cgi-bin/locus.pl?locus=PWP2)*,* [*PXR1*](http://db.yeastgenome.org/cgi-bin/locus.pl?locus=PXR1)*,* [*RAP1*](http://db.yeastgenome.org/cgi-bin/locus.pl?locus=RAP1)*,* [*RAS1*](http://db.yeastgenome.org/cgi-bin/locus.pl?locus=RAS1)*,* [*RBA50*](http://db.yeastgenome.org/cgi-bin/locus.pl?locus=RBA50)*,* [*RBG1*](http://db.yeastgenome.org/cgi-bin/locus.pl?locus=RBG1)*,* [*RCL1*](http://db.yeastgenome.org/cgi-bin/locus.pl?locus=RCL1)*,* [*REI1*](http://db.yeastgenome.org/cgi-bin/locus.pl?locus=REI1)*,* [*RET1*](http://db.yeastgenome.org/cgi-bin/locus.pl?locus=RET1)*,* [*REX4*](http://db.yeastgenome.org/cgi-bin/locus.pl?locus=REX4)*,* [*RHR2*](http://db.yeastgenome.org/cgi-bin/locus.pl?locus=RHR2)*,* [*RIO1*](http://db.yeastgenome.org/cgi-bin/locus.pl?locus=RIO1)*,* [*RIX1*](http://db.yeastgenome.org/cgi-bin/locus.pl?locus=RIX1)*,* [*RIX7*](http://db.yeastgenome.org/cgi-bin/locus.pl?locus=RIX7)*,* [*RKI1*](http://db.yeastgenome.org/cgi-bin/locus.pl?locus=RKI1)*,* [*RLI1*](http://db.yeastgenome.org/cgi-bin/locus.pl?locus=RLI1)*,* [*RLP24*](http://db.yeastgenome.org/cgi-bin/locus.pl?locus=RLP24)*,* [*RLP7*](http://db.yeastgenome.org/cgi-bin/locus.pl?locus=RLP7)*,* [*RMT2*](http://db.yeastgenome.org/cgi-bin/locus.pl?locus=RMT2)*,* [*RNH201*](http://db.yeastgenome.org/cgi-bin/locus.pl?locus=RNH201)*,* [*RNT1*](http://db.yeastgenome.org/cgi-bin/locus.pl?locus=RNT1)*,* [*ROK1*](http://db.yeastgenome.org/cgi-bin/locus.pl?locus=ROK1)*,* [*ROX3*](http://db.yeastgenome.org/cgi-bin/locus.pl?locus=ROX3)*,* [*RPA12*](http://db.yeastgenome.org/cgi-bin/locus.pl?locus=RPA12)*,* [*RPA190*](http://db.yeastgenome.org/cgi-bin/locus.pl?locus=RPA190)*,* [*RPA34*](http://db.yeastgenome.org/cgi-bin/locus.pl?locus=RPA34)*,* [*RPA43*](http://db.yeastgenome.org/cgi-bin/locus.pl?locus=RPA43)*,* [*RPA49*](http://db.yeastgenome.org/cgi-bin/locus.pl?locus=RPA49)*,* [*RPB10*](http://db.yeastgenome.org/cgi-bin/locus.pl?locus=RPB10)*,* [*RPB5*](http://db.yeastgenome.org/cgi-bin/locus.pl?locus=RPB5)*,* [*RPB8*](http://db.yeastgenome.org/cgi-bin/locus.pl?locus=RPB8)*,* [*RPB9*](http://db.yeastgenome.org/cgi-bin/locus.pl?locus=RPB9)*,* [*RPC11*](http://db.yeastgenome.org/cgi-bin/locus.pl?locus=RPC11)*,* [*RPC19*](http://db.yeastgenome.org/cgi-bin/locus.pl?locus=RPC19)*,* [*RPC31*](http://db.yeastgenome.org/cgi-bin/locus.pl?locus=RPC31)*,* [*RPC34*](http://db.yeastgenome.org/cgi-bin/locus.pl?locus=RPC34)*,* [*RPC37*](http://db.yeastgenome.org/cgi-bin/locus.pl?locus=RPC37)*,* [*RPC40*](http://db.yeastgenome.org/cgi-bin/locus.pl?locus=RPC40)*,* [*RPC53*](http://db.yeastgenome.org/cgi-bin/locus.pl?locus=RPC53)*,* [*RPC82*](http://db.yeastgenome.org/cgi-bin/locus.pl?locus=RPC82)*,* [*RPF1*](http://db.yeastgenome.org/cgi-bin/locus.pl?locus=RPF1)*,* [*RPF2*](http://db.yeastgenome.org/cgi-bin/locus.pl?locus=RPF2)*,* [*RPL13A*](http://db.yeastgenome.org/cgi-bin/locus.pl?locus=RPL13A)*,* [*RPL14A*](http://db.yeastgenome.org/cgi-bin/locus.pl?locus=RPL14A)*,* [*RPL14B*](http://db.yeastgenome.org/cgi-bin/locus.pl?locus=RPL14B)*,* [*RPL18B*](http://db.yeastgenome.org/cgi-bin/locus.pl?locus=RPL18B)*,* [*RPL20A*](http://db.yeastgenome.org/cgi-bin/locus.pl?locus=RPL20A)*,* [*RPL21B*](http://db.yeastgenome.org/cgi-bin/locus.pl?locus=RPL21B)*,* [*RPL26A*](http://db.yeastgenome.org/cgi-bin/locus.pl?locus=RPL26A)*,* [*RPL31B*](http://db.yeastgenome.org/cgi-bin/locus.pl?locus=RPL31B)*,* [*RPL33B*](http://db.yeastgenome.org/cgi-bin/locus.pl?locus=RPL33B)*,* [*RPL36A*](http://db.yeastgenome.org/cgi-bin/locus.pl?locus=RPL36A)*,* [*RPL43B*](http://db.yeastgenome.org/cgi-bin/locus.pl?locus=RPL43B)*,* [*RPL7B*](http://db.yeastgenome.org/cgi-bin/locus.pl?locus=RPL7B)*,* [*RPO26*](http://db.yeastgenome.org/cgi-bin/locus.pl?locus=RPO26)*,* [*RPP1*](http://db.yeastgenome.org/cgi-bin/locus.pl?locus=RPP1)*,* [*RPS10B*](http://db.yeastgenome.org/cgi-bin/locus.pl?locus=RPS10B)*,* [*RPS14B*](http://db.yeastgenome.org/cgi-bin/locus.pl?locus=RPS14B)*,* [*RPS19B*](http://db.yeastgenome.org/cgi-bin/locus.pl?locus=RPS19B)*,* [*RPS1A*](http://db.yeastgenome.org/cgi-bin/locus.pl?locus=RPS1A)*,* [*RPS21A*](http://db.yeastgenome.org/cgi-bin/locus.pl?locus=RPS21A)*,* [*RPS21B*](http://db.yeastgenome.org/cgi-bin/locus.pl?locus=RPS21B)*,* [*RPS26B*](http://db.yeastgenome.org/cgi-bin/locus.pl?locus=RPS26B)*,* [*RPS27A*](http://db.yeastgenome.org/cgi-bin/locus.pl?locus=RPS27A)*,* [*RPS29A*](http://db.yeastgenome.org/cgi-bin/locus.pl?locus=RPS29A)*,* [*RPS7A*](http://db.yeastgenome.org/cgi-bin/locus.pl?locus=RPS7A)*,* [*RPS7B*](http://db.yeastgenome.org/cgi-bin/locus.pl?locus=RPS7B)*,* [*RRB1*](http://db.yeastgenome.org/cgi-bin/locus.pl?locus=RRB1)*,* [*RRM3*](http://db.yeastgenome.org/cgi-bin/locus.pl?locus=RRM3)*,* [*RRN11*](http://db.yeastgenome.org/cgi-bin/locus.pl?locus=RRN11)*,* [*RRN7*](http://db.yeastgenome.org/cgi-bin/locus.pl?locus=RRN7)*,* [*RRP1*](http://db.yeastgenome.org/cgi-bin/locus.pl?locus=RRP1)*,* [*RRP12*](http://db.yeastgenome.org/cgi-bin/locus.pl?locus=RRP12)*,* [*RRP14*](http://db.yeastgenome.org/cgi-bin/locus.pl?locus=RRP14)*,* [*RRP15*](http://db.yeastgenome.org/cgi-bin/locus.pl?locus=RRP15)*,* [*RRP17*](http://db.yeastgenome.org/cgi-bin/locus.pl?locus=RRP17)*,* [*RRP3*](http://db.yeastgenome.org/cgi-bin/locus.pl?locus=RRP3)*, RRP36,* [*RRP40*](http://db.yeastgenome.org/cgi-bin/locus.pl?locus=RRP40)*,* [*RRP43*](http://db.yeastgenome.org/cgi-bin/locus.pl?locus=RRP43)*,* [*RRP5*](http://db.yeastgenome.org/cgi-bin/locus.pl?locus=RRP5)*,* [*RRP6*](http://db.yeastgenome.org/cgi-bin/locus.pl?locus=RRP6)*,* [*RRP8*](http://db.yeastgenome.org/cgi-bin/locus.pl?locus=RRP8)*,* [*RRP9*](http://db.yeastgenome.org/cgi-bin/locus.pl?locus=RRP9)*,* [*RRS1*](http://db.yeastgenome.org/cgi-bin/locus.pl?locus=RRS1)*, RRT14,* [*RSA1*](http://db.yeastgenome.org/cgi-bin/locus.pl?locus=RSA1)*,* [*RSA3*](http://db.yeastgenome.org/cgi-bin/locus.pl?locus=RSA3)*,* [*RSA4*](http://db.yeastgenome.org/cgi-bin/locus.pl?locus=RSA4)*,* [*RVB1*](http://db.yeastgenome.org/cgi-bin/locus.pl?locus=RVB1)*,* [*SAN1*](http://db.yeastgenome.org/cgi-bin/locus.pl?locus=SAN1)*,* [*SAP185*](http://db.yeastgenome.org/cgi-bin/locus.pl?locus=SAP185)*,* [*SAP190*](http://db.yeastgenome.org/cgi-bin/locus.pl?locus=SAP190)*,* [*SAS10*](http://db.yeastgenome.org/cgi-bin/locus.pl?locus=SAS10)*,* [*SCC2*](http://db.yeastgenome.org/cgi-bin/locus.pl?locus=SCC2)*,* [*SCH9*](http://db.yeastgenome.org/cgi-bin/locus.pl?locus=SCH9)*,* [*SCM4*](http://db.yeastgenome.org/cgi-bin/locus.pl?locus=SCM4)*,* [*SCS7*](http://db.yeastgenome.org/cgi-bin/locus.pl?locus=SCS7)*,* [*SCW11*](http://db.yeastgenome.org/cgi-bin/locus.pl?locus=SCW11)*,* [*SDA1*](http://db.yeastgenome.org/cgi-bin/locus.pl?locus=SDA1)*,* [*SEC53*](http://db.yeastgenome.org/cgi-bin/locus.pl?locus=SEC53)*, SEE1,* [*SEN34*](http://db.yeastgenome.org/cgi-bin/locus.pl?locus=SEN34)*,* [*SFG1*](http://db.yeastgenome.org/cgi-bin/locus.pl?locus=SFG1)*,* [*SGO1*](http://db.yeastgenome.org/cgi-bin/locus.pl?locus=SGO1)*,* [*SHO1*](http://db.yeastgenome.org/cgi-bin/locus.pl?locus=SHO1)*,* [*SHQ1*](http://db.yeastgenome.org/cgi-bin/locus.pl?locus=SHQ1)*,* [*SIK1*](http://db.yeastgenome.org/cgi-bin/locus.pl?locus=SIK1)*,* [*SIM1*](http://db.yeastgenome.org/cgi-bin/locus.pl?locus=SIM1)*,* [*SIR2*](http://db.yeastgenome.org/cgi-bin/locus.pl?locus=SIR2)*,* [*SLI15*](http://db.yeastgenome.org/cgi-bin/locus.pl?locus=SLI15)*,* [*SLK19*](http://db.yeastgenome.org/cgi-bin/locus.pl?locus=SLK19)*,* [*SLN1*](http://db.yeastgenome.org/cgi-bin/locus.pl?locus=SLN1)*,* [*SLX9*](http://db.yeastgenome.org/cgi-bin/locus.pl?locus=SLX9)*,* [*SLY41*](http://db.yeastgenome.org/cgi-bin/locus.pl?locus=SLY41)*,* [*SMC2*](http://db.yeastgenome.org/cgi-bin/locus.pl?locus=SMC2)*,* [*SMI1*](http://db.yeastgenome.org/cgi-bin/locus.pl?locus=SMI1)*,* [*SMY2*](http://db.yeastgenome.org/cgi-bin/locus.pl?locus=SMY2)*,* [*SNU13*](http://db.yeastgenome.org/cgi-bin/locus.pl?locus=SNU13)*,* [*SOF1*](http://db.yeastgenome.org/cgi-bin/locus.pl?locus=SOF1)*,* [*SPA2*](http://db.yeastgenome.org/cgi-bin/locus.pl?locus=SPA2)*,* [*SPB1*](http://db.yeastgenome.org/cgi-bin/locus.pl?locus=SPB1)*,* [*SPB4*](http://db.yeastgenome.org/cgi-bin/locus.pl?locus=SPB4)*,* [*SPC98*](http://db.yeastgenome.org/cgi-bin/locus.pl?locus=SPC98)*,* [*SPE4*](http://db.yeastgenome.org/cgi-bin/locus.pl?locus=SPE4)*,* [*SQT1*](http://db.yeastgenome.org/cgi-bin/locus.pl?locus=SQT1)*,* [*SRO9*](http://db.yeastgenome.org/cgi-bin/locus.pl?locus=SRO9)*,* [*SRP40*](http://db.yeastgenome.org/cgi-bin/locus.pl?locus=SRP40)*,* [*SSF1*](http://db.yeastgenome.org/cgi-bin/locus.pl?locus=SSF1)*,* [*SSF2*](http://db.yeastgenome.org/cgi-bin/locus.pl?locus=SSF2)*,* [*STE12*](http://db.yeastgenome.org/cgi-bin/locus.pl?locus=STE12)*,* [*STU2*](http://db.yeastgenome.org/cgi-bin/locus.pl?locus=STU2)*,* [*SUN4*](http://db.yeastgenome.org/cgi-bin/locus.pl?locus=SUN4)*,* [*SUR2*](http://db.yeastgenome.org/cgi-bin/locus.pl?locus=SUR2)*,* [*SUR4*](http://db.yeastgenome.org/cgi-bin/locus.pl?locus=SUR4)*,* [*SUR7*](http://db.yeastgenome.org/cgi-bin/locus.pl?locus=SUR7)*,* [*SUT1*](http://db.yeastgenome.org/cgi-bin/locus.pl?locus=SUT1)*,* [*SUT2*](http://db.yeastgenome.org/cgi-bin/locus.pl?locus=SUT2)*,* [*SUV3*](http://db.yeastgenome.org/cgi-bin/locus.pl?locus=SUV3)*,* [*SVF1*](http://db.yeastgenome.org/cgi-bin/locus.pl?locus=SVF1)*,* [*SWI5*](http://db.yeastgenome.org/cgi-bin/locus.pl?locus=SWI5)*,* [*SXM1*](http://db.yeastgenome.org/cgi-bin/locus.pl?locus=SXM1)*,* [*SYG1*](http://db.yeastgenome.org/cgi-bin/locus.pl?locus=SYG1)*,* [*TAD3*](http://db.yeastgenome.org/cgi-bin/locus.pl?locus=TAD3)*,* [*TEL2*](http://db.yeastgenome.org/cgi-bin/locus.pl?locus=TEL2)*,* [*TEM1*](http://db.yeastgenome.org/cgi-bin/locus.pl?locus=TEM1)*,* [*THI21*](http://db.yeastgenome.org/cgi-bin/locus.pl?locus=THI21)*,* [*THP2*](http://db.yeastgenome.org/cgi-bin/locus.pl?locus=THP2)*,* [*TIF2*](http://db.yeastgenome.org/cgi-bin/locus.pl?locus=TIF2)*,* [*TIF3*](http://db.yeastgenome.org/cgi-bin/locus.pl?locus=TIF3)*,* [*TIF35*](http://db.yeastgenome.org/cgi-bin/locus.pl?locus=TIF35)*,* [*TIF4631*](http://db.yeastgenome.org/cgi-bin/locus.pl?locus=TIF4631)*,* [*TIP20*](http://db.yeastgenome.org/cgi-bin/locus.pl?locus=TIP20)*,* [*TMA23*](http://db.yeastgenome.org/cgi-bin/locus.pl?locus=TMA23)*,* [*TMA46*](http://db.yeastgenome.org/cgi-bin/locus.pl?locus=TMA46)*, TOB6,* [*TOP1*](http://db.yeastgenome.org/cgi-bin/locus.pl?locus=TOP1)*,* [*TOS4*](http://db.yeastgenome.org/cgi-bin/locus.pl?locus=TOS4)*,* [*TPA1*](http://db.yeastgenome.org/cgi-bin/locus.pl?locus=TPA1)*,* [*TRF5*](http://db.yeastgenome.org/cgi-bin/locus.pl?locus=TRF5)*,* [*TRM1*](http://db.yeastgenome.org/cgi-bin/locus.pl?locus=TRM1)*,* [*TRM10*](http://db.yeastgenome.org/cgi-bin/locus.pl?locus=TRM10)*,* [*TRM11*](http://db.yeastgenome.org/cgi-bin/locus.pl?locus=TRM11)*,* [*TRM112*](http://db.yeastgenome.org/cgi-bin/locus.pl?locus=TRM112)*,* [*TRM13*](http://db.yeastgenome.org/cgi-bin/locus.pl?locus=TRM13)*,* [*TRM2*](http://db.yeastgenome.org/cgi-bin/locus.pl?locus=TRM2)*,* [*TRM3*](http://db.yeastgenome.org/cgi-bin/locus.pl?locus=TRM3)*,* [*TRM7*](http://db.yeastgenome.org/cgi-bin/locus.pl?locus=TRM7)*,* [*TRM8*](http://db.yeastgenome.org/cgi-bin/locus.pl?locus=TRM8)*,* [*TRM82*](http://db.yeastgenome.org/cgi-bin/locus.pl?locus=TRM82)*,* [*TRM9*](http://db.yeastgenome.org/cgi-bin/locus.pl?locus=TRM9)*,* [*TSC10*](http://db.yeastgenome.org/cgi-bin/locus.pl?locus=TSC10)*,* [*TSR1*](http://db.yeastgenome.org/cgi-bin/locus.pl?locus=TSR1)*,* [*TSR2*](http://db.yeastgenome.org/cgi-bin/locus.pl?locus=TSR2)*,* [*TYW1*](http://db.yeastgenome.org/cgi-bin/locus.pl?locus=TYW1)*,* [*UBC9*](http://db.yeastgenome.org/cgi-bin/locus.pl?locus=UBC9)*,* [*UBP10*](http://db.yeastgenome.org/cgi-bin/locus.pl?locus=UBP10)*,* [*UNG1*](http://db.yeastgenome.org/cgi-bin/locus.pl?locus=UNG1)*,* [*URA4*](http://db.yeastgenome.org/cgi-bin/locus.pl?locus=URA4)*, URA5,* [*URA7*](http://db.yeastgenome.org/cgi-bin/locus.pl?locus=URA7)*,* [*URB1*](http://db.yeastgenome.org/cgi-bin/locus.pl?locus=URB1)*,* [*URB2*](http://db.yeastgenome.org/cgi-bin/locus.pl?locus=URB2)*,* [*URK1*](http://db.yeastgenome.org/cgi-bin/locus.pl?locus=URK1)*,* [*UTP10*](http://db.yeastgenome.org/cgi-bin/locus.pl?locus=UTP10)*,* [*UTP11*](http://db.yeastgenome.org/cgi-bin/locus.pl?locus=UTP11)*,* [*UTP13*](http://db.yeastgenome.org/cgi-bin/locus.pl?locus=UTP13)*,* [*UTP14*](http://db.yeastgenome.org/cgi-bin/locus.pl?locus=UTP14)*,* [*UTP15*](http://db.yeastgenome.org/cgi-bin/locus.pl?locus=UTP15)*,* [*UTP18*](http://db.yeastgenome.org/cgi-bin/locus.pl?locus=UTP18)*,* [*UTP21*](http://db.yeastgenome.org/cgi-bin/locus.pl?locus=UTP21)*,* [*UTP22*](http://db.yeastgenome.org/cgi-bin/locus.pl?locus=UTP22)*,* [*UTP23*](http://db.yeastgenome.org/cgi-bin/locus.pl?locus=UTP23)*,* [*UTP30*](http://db.yeastgenome.org/cgi-bin/locus.pl?locus=UTP30)*,* [*UTP4*](http://db.yeastgenome.org/cgi-bin/locus.pl?locus=UTP4)*,* [*UTP5*](http://db.yeastgenome.org/cgi-bin/locus.pl?locus=UTP5)*,* [*UTP7*](http://db.yeastgenome.org/cgi-bin/locus.pl?locus=UTP7)*,* [*UTP8*](http://db.yeastgenome.org/cgi-bin/locus.pl?locus=UTP8)*,* [*UTP9*](http://db.yeastgenome.org/cgi-bin/locus.pl?locus=UTP9)*,* [*UTR2*](http://db.yeastgenome.org/cgi-bin/locus.pl?locus=UTR2)*,* [*VRG4*](http://db.yeastgenome.org/cgi-bin/locus.pl?locus=VRG4)*,* [*VTC1*](http://db.yeastgenome.org/cgi-bin/locus.pl?locus=VTC1)*,* [*VTC3*](http://db.yeastgenome.org/cgi-bin/locus.pl?locus=VTC3)*,* [*WRS1*](http://db.yeastgenome.org/cgi-bin/locus.pl?locus=WRS1)*,* [*WTM2*](http://db.yeastgenome.org/cgi-bin/locus.pl?locus=WTM2)*,* [*YAR1*](http://db.yeastgenome.org/cgi-bin/locus.pl?locus=YAR1)*,* [*YBL028C*](http://db.yeastgenome.org/cgi-bin/locus.pl?locus=YBL028C)*,* [*YBR238C*](http://db.yeastgenome.org/cgi-bin/locus.pl?locus=YBR238C)*,* [*YCG1*](http://db.yeastgenome.org/cgi-bin/locus.pl?locus=YCG1)*,* [*YCR016W*](http://db.yeastgenome.org/cgi-bin/locus.pl?locus=YCR016W)*,* [*YDL063C*](http://db.yeastgenome.org/cgi-bin/locus.pl?locus=YDL063C)*,* [*YEF3*](http://db.yeastgenome.org/cgi-bin/locus.pl?locus=YEF3)*,* [*YGR283C*](http://db.yeastgenome.org/cgi-bin/locus.pl?locus=YGR283C)*,* [*YHL039W*](http://db.yeastgenome.org/cgi-bin/locus.pl?locus=YHL039W)*,* [*YHM2*](http://db.yeastgenome.org/cgi-bin/locus.pl?locus=YHM2)*,* [*YHR020W*](http://db.yeastgenome.org/cgi-bin/locus.pl?locus=YHR020W)*,* [*YIL096C*](http://db.yeastgenome.org/cgi-bin/locus.pl?locus=YIL096C)*,* [*YML082W*](http://db.yeastgenome.org/cgi-bin/locus.pl?locus=YML082W)*,* [*YMR310C*](http://db.yeastgenome.org/cgi-bin/locus.pl?locus=YMR310C)*,* [*YNL022C*](http://db.yeastgenome.org/cgi-bin/locus.pl?locus=YNL022C)*,* [*YNL247W*](http://db.yeastgenome.org/cgi-bin/locus.pl?locus=YNL247W)*,* [*YNL313C*](http://db.yeastgenome.org/cgi-bin/locus.pl?locus=YNL313C)*,* [*YOR021C*](http://db.yeastgenome.org/cgi-bin/locus.pl?locus=YOR021C)*,* [*YOX1*](http://db.yeastgenome.org/cgi-bin/locus.pl?locus=YOX1)*,* [*YRF1-6*](http://db.yeastgenome.org/cgi-bin/locus.pl?locus=YRF1-6)*, YTM1, YVH1,* [*ZRC1*](http://db.yeastgenome.org/cgi-bin/locus.pl?locus=ZRC1) |
| Metabolic process | 375 out of 558 genes, 67.2% | 4.89e-18 | [*AAH1*](http://db.yeastgenome.org/cgi-bin/locus.pl?locus=AAH1)*,* [*ADE17*](http://db.yeastgenome.org/cgi-bin/locus.pl?locus=ADE17)*,* [*ADE5,7*](http://db.yeastgenome.org/cgi-bin/locus.pl?locus=ADE5,7)*,* [*ADE6*](http://db.yeastgenome.org/cgi-bin/locus.pl?locus=ADE6)*,* [*ADE8*](http://db.yeastgenome.org/cgi-bin/locus.pl?locus=ADE8)*,* [*AIR1*](http://db.yeastgenome.org/cgi-bin/locus.pl?locus=AIR1)*,* [*ALK1*](http://db.yeastgenome.org/cgi-bin/locus.pl?locus=ALK1)*,* [*APT1*](http://db.yeastgenome.org/cgi-bin/locus.pl?locus=APT1)*,* [*ASC1*](http://db.yeastgenome.org/cgi-bin/locus.pl?locus=ASC1)*,* [*ASH1*](http://db.yeastgenome.org/cgi-bin/locus.pl?locus=ASH1)*,* [*ASP1*](http://db.yeastgenome.org/cgi-bin/locus.pl?locus=ASP1)*,* [*ATF2*](http://db.yeastgenome.org/cgi-bin/locus.pl?locus=ATF2)*,* [*ATO3*](http://db.yeastgenome.org/cgi-bin/locus.pl?locus=ATO3)*,* [*AUR1*](http://db.yeastgenome.org/cgi-bin/locus.pl?locus=AUR1)*,* [*BCD1*](http://db.yeastgenome.org/cgi-bin/locus.pl?locus=BCD1)*,* [*BIO2*](http://db.yeastgenome.org/cgi-bin/locus.pl?locus=BIO2)*,* [*BMS1*](http://db.yeastgenome.org/cgi-bin/locus.pl?locus=BMS1)*,* [*BRX1*](http://db.yeastgenome.org/cgi-bin/locus.pl?locus=BRX1)*,* [*BST1*](http://db.yeastgenome.org/cgi-bin/locus.pl?locus=BST1)*,* [*BUD21*](http://db.yeastgenome.org/cgi-bin/locus.pl?locus=BUD21)*,* [*BUD32*](http://db.yeastgenome.org/cgi-bin/locus.pl?locus=BUD32)*,* [*CBF5*](http://db.yeastgenome.org/cgi-bin/locus.pl?locus=CBF5)*,* [*CDC14*](http://db.yeastgenome.org/cgi-bin/locus.pl?locus=CDC14)*,* [*CDC20*](http://db.yeastgenome.org/cgi-bin/locus.pl?locus=CDC20)*,* [*CDC47*](http://db.yeastgenome.org/cgi-bin/locus.pl?locus=CDC47)*,* [*CGR1*](http://db.yeastgenome.org/cgi-bin/locus.pl?locus=CGR1)*,* [*CIC1*](http://db.yeastgenome.org/cgi-bin/locus.pl?locus=CIC1)*,* [*CLA4*](http://db.yeastgenome.org/cgi-bin/locus.pl?locus=CLA4)*, CMS1,* [*CSM3*](http://db.yeastgenome.org/cgi-bin/locus.pl?locus=CSM3)*,* [*CUS1*](http://db.yeastgenome.org/cgi-bin/locus.pl?locus=CUS1)*,* [*CWC2*](http://db.yeastgenome.org/cgi-bin/locus.pl?locus=CWC2)*,* [*CYB5*](http://db.yeastgenome.org/cgi-bin/locus.pl?locus=CYB5)*,* [*DBP10*](http://db.yeastgenome.org/cgi-bin/locus.pl?locus=DBP10)*,* [*DBP2*](http://db.yeastgenome.org/cgi-bin/locus.pl?locus=DBP2)*,* [*DBP3*](http://db.yeastgenome.org/cgi-bin/locus.pl?locus=DBP3)*,* [*DBP6*](http://db.yeastgenome.org/cgi-bin/locus.pl?locus=DBP6)*,* [*DBP7*](http://db.yeastgenome.org/cgi-bin/locus.pl?locus=DBP7)*,* [*DBP8*](http://db.yeastgenome.org/cgi-bin/locus.pl?locus=DBP8)*,* [*DBP9*](http://db.yeastgenome.org/cgi-bin/locus.pl?locus=DBP9)*,* [*DFR1*](http://db.yeastgenome.org/cgi-bin/locus.pl?locus=DFR1)*,* [*DIM1*](http://db.yeastgenome.org/cgi-bin/locus.pl?locus=DIM1)*,* [*DIP2*](http://db.yeastgenome.org/cgi-bin/locus.pl?locus=DIP2)*,* [*DPB2*](http://db.yeastgenome.org/cgi-bin/locus.pl?locus=DPB2)*,* [*DPH2*](http://db.yeastgenome.org/cgi-bin/locus.pl?locus=DPH2)*,* [*DRS1*](http://db.yeastgenome.org/cgi-bin/locus.pl?locus=DRS1)*,* [*DUS1*](http://db.yeastgenome.org/cgi-bin/locus.pl?locus=DUS1)*,* [*DUS3*](http://db.yeastgenome.org/cgi-bin/locus.pl?locus=DUS3)*,* [*DUS4*](http://db.yeastgenome.org/cgi-bin/locus.pl?locus=DUS4)*,* [*DUT1*](http://db.yeastgenome.org/cgi-bin/locus.pl?locus=DUT1)*,* [*DYS1*](http://db.yeastgenome.org/cgi-bin/locus.pl?locus=DYS1)*,* [*EBP2*](http://db.yeastgenome.org/cgi-bin/locus.pl?locus=EBP2)*,* [*ECM16*](http://db.yeastgenome.org/cgi-bin/locus.pl?locus=ECM16)*,* [*EFB1*](http://db.yeastgenome.org/cgi-bin/locus.pl?locus=EFB1)*, EFG1,* [*EKI1*](http://db.yeastgenome.org/cgi-bin/locus.pl?locus=EKI1)*,* [*ELP2*](http://db.yeastgenome.org/cgi-bin/locus.pl?locus=ELP2)*,* [*ELP3*](http://db.yeastgenome.org/cgi-bin/locus.pl?locus=ELP3)*,* [*EMG1*](http://db.yeastgenome.org/cgi-bin/locus.pl?locus=EMG1)*,* [*ENP1*](http://db.yeastgenome.org/cgi-bin/locus.pl?locus=ENP1)*,* [*ENP2*](http://db.yeastgenome.org/cgi-bin/locus.pl?locus=ENP2)*,* [*ERB1*](http://db.yeastgenome.org/cgi-bin/locus.pl?locus=ERB1)*,* [*ERG1*](http://db.yeastgenome.org/cgi-bin/locus.pl?locus=ERG1)*,* [*ERG11*](http://db.yeastgenome.org/cgi-bin/locus.pl?locus=ERG11)*,* [*ERG3*](http://db.yeastgenome.org/cgi-bin/locus.pl?locus=ERG3)*,* [*ERG5*](http://db.yeastgenome.org/cgi-bin/locus.pl?locus=ERG5)*,* [*ESF1*](http://db.yeastgenome.org/cgi-bin/locus.pl?locus=ESF1)*,* [*ESF2*](http://db.yeastgenome.org/cgi-bin/locus.pl?locus=ESF2)*,* [*EXG1*](http://db.yeastgenome.org/cgi-bin/locus.pl?locus=EXG1)*,* [*FAA4*](http://db.yeastgenome.org/cgi-bin/locus.pl?locus=FAA4)*,* [*FAF1*](http://db.yeastgenome.org/cgi-bin/locus.pl?locus=FAF1)*,* [*FAL1*](http://db.yeastgenome.org/cgi-bin/locus.pl?locus=FAL1)*,* [*FAP7*](http://db.yeastgenome.org/cgi-bin/locus.pl?locus=FAP7)*,* [*FCF2*](http://db.yeastgenome.org/cgi-bin/locus.pl?locus=FCF2)*,* [*FEN1*](http://db.yeastgenome.org/cgi-bin/locus.pl?locus=FEN1)*,* [*FKH1*](http://db.yeastgenome.org/cgi-bin/locus.pl?locus=FKH1)*,* [*FOL1*](http://db.yeastgenome.org/cgi-bin/locus.pl?locus=FOL1)*,* [*FPR4*](http://db.yeastgenome.org/cgi-bin/locus.pl?locus=FPR4)*,* [*FUR1*](http://db.yeastgenome.org/cgi-bin/locus.pl?locus=FUR1)*,* [*FYV7*](http://db.yeastgenome.org/cgi-bin/locus.pl?locus=FYV7)*,* [*GAR1*](http://db.yeastgenome.org/cgi-bin/locus.pl?locus=GAR1)*,* [*GCD10*](http://db.yeastgenome.org/cgi-bin/locus.pl?locus=GCD10)*,* [*GCD11*](http://db.yeastgenome.org/cgi-bin/locus.pl?locus=GCD11)*,* [*GCD14*](http://db.yeastgenome.org/cgi-bin/locus.pl?locus=GCD14)*,* [*GCD2*](http://db.yeastgenome.org/cgi-bin/locus.pl?locus=GCD2)*,* [*GCN3*](http://db.yeastgenome.org/cgi-bin/locus.pl?locus=GCN3)*,* [*GCR1*](http://db.yeastgenome.org/cgi-bin/locus.pl?locus=GCR1)*,* [*GCR2*](http://db.yeastgenome.org/cgi-bin/locus.pl?locus=GCR2)*,* [*GLN1*](http://db.yeastgenome.org/cgi-bin/locus.pl?locus=GLN1)*,* [*GLN4*](http://db.yeastgenome.org/cgi-bin/locus.pl?locus=GLN4)*,* [*GPI13*](http://db.yeastgenome.org/cgi-bin/locus.pl?locus=GPI13)*,* [*GRC3*](http://db.yeastgenome.org/cgi-bin/locus.pl?locus=GRC3)*,* [*GUA1*](http://db.yeastgenome.org/cgi-bin/locus.pl?locus=GUA1)*,* [*HAS1*](http://db.yeastgenome.org/cgi-bin/locus.pl?locus=HAS1)*,* [*HCA4*](http://db.yeastgenome.org/cgi-bin/locus.pl?locus=HCA4)*,* [*HEM1*](http://db.yeastgenome.org/cgi-bin/locus.pl?locus=HEM1)*,* [*HHF2*](http://db.yeastgenome.org/cgi-bin/locus.pl?locus=HHF2)*,* [*HHT1*](http://db.yeastgenome.org/cgi-bin/locus.pl?locus=HHT1)*,* [*HIR2*](http://db.yeastgenome.org/cgi-bin/locus.pl?locus=HIR2)*,* [*HMG1*](http://db.yeastgenome.org/cgi-bin/locus.pl?locus=HMG1)*,* [*HMT1*](http://db.yeastgenome.org/cgi-bin/locus.pl?locus=HMT1)*,* [*HPT1*](http://db.yeastgenome.org/cgi-bin/locus.pl?locus=HPT1)*,* [*HST3*](http://db.yeastgenome.org/cgi-bin/locus.pl?locus=HST3)*,* [*HTA1*](http://db.yeastgenome.org/cgi-bin/locus.pl?locus=HTA1)*,* [*HTA2*](http://db.yeastgenome.org/cgi-bin/locus.pl?locus=HTA2)*,* [*HTB1*](http://db.yeastgenome.org/cgi-bin/locus.pl?locus=HTB1)*,* [*HTB2*](http://db.yeastgenome.org/cgi-bin/locus.pl?locus=HTB2)*,* [*HTZ1*](http://db.yeastgenome.org/cgi-bin/locus.pl?locus=HTZ1)*,* [*IFH1*](http://db.yeastgenome.org/cgi-bin/locus.pl?locus=IFH1)*,* [*ILV3*](http://db.yeastgenome.org/cgi-bin/locus.pl?locus=ILV3)*,* [*ILV5*](http://db.yeastgenome.org/cgi-bin/locus.pl?locus=ILV5)*,* [*IMD3*](http://db.yeastgenome.org/cgi-bin/locus.pl?locus=IMD3)*,* [*IMP3*](http://db.yeastgenome.org/cgi-bin/locus.pl?locus=IMP3)*,* [*INO2*](http://db.yeastgenome.org/cgi-bin/locus.pl?locus=INO2)*,* [*IPI1*](http://db.yeastgenome.org/cgi-bin/locus.pl?locus=IPI1)*,* [*IPI3*](http://db.yeastgenome.org/cgi-bin/locus.pl?locus=IPI3)*,* [*IZH1*](http://db.yeastgenome.org/cgi-bin/locus.pl?locus=IZH1)*,* [*JJJ3*](http://db.yeastgenome.org/cgi-bin/locus.pl?locus=JJJ3)*,* [*KAE1*](http://db.yeastgenome.org/cgi-bin/locus.pl?locus=KAE1)*,* [*KCS1*](http://db.yeastgenome.org/cgi-bin/locus.pl?locus=KCS1)*,* [*KRR1*](http://db.yeastgenome.org/cgi-bin/locus.pl?locus=KRR1)*,* [*KTI12*](http://db.yeastgenome.org/cgi-bin/locus.pl?locus=KTI12)*,* [*KTR5*](http://db.yeastgenome.org/cgi-bin/locus.pl?locus=KTR5)*,* [*LCP5*](http://db.yeastgenome.org/cgi-bin/locus.pl?locus=LCP5)*,* [*LEU9*](http://db.yeastgenome.org/cgi-bin/locus.pl?locus=LEU9)*,* [*LIA1*](http://db.yeastgenome.org/cgi-bin/locus.pl?locus=LIA1)*,* [*LYS12*](http://db.yeastgenome.org/cgi-bin/locus.pl?locus=LYS12)*,* [*LYS4*](http://db.yeastgenome.org/cgi-bin/locus.pl?locus=LYS4)*,* [*MAE1*](http://db.yeastgenome.org/cgi-bin/locus.pl?locus=MAE1)*,* [*MAK11*](http://db.yeastgenome.org/cgi-bin/locus.pl?locus=MAK11)*,* [*MAK16*](http://db.yeastgenome.org/cgi-bin/locus.pl?locus=MAK16)*,* [*MAK21*](http://db.yeastgenome.org/cgi-bin/locus.pl?locus=MAK21)*,* [*MAK3*](http://db.yeastgenome.org/cgi-bin/locus.pl?locus=MAK3)*,* [*MAP1*](http://db.yeastgenome.org/cgi-bin/locus.pl?locus=MAP1)*,* [*MEP3*](http://db.yeastgenome.org/cgi-bin/locus.pl?locus=MEP3)*,* [*MES1*](http://db.yeastgenome.org/cgi-bin/locus.pl?locus=MES1)*,* [*MEU1*](http://db.yeastgenome.org/cgi-bin/locus.pl?locus=MEU1)*,* [*MIS1*](http://db.yeastgenome.org/cgi-bin/locus.pl?locus=MIS1)*,* [*MNN2*](http://db.yeastgenome.org/cgi-bin/locus.pl?locus=MNN2)*,* [*MOT1*](http://db.yeastgenome.org/cgi-bin/locus.pl?locus=MOT1)*,* [*MPP10*](http://db.yeastgenome.org/cgi-bin/locus.pl?locus=MPP10)*,* [*MRD1*](http://db.yeastgenome.org/cgi-bin/locus.pl?locus=MRD1)*,* [*MRT4*](http://db.yeastgenome.org/cgi-bin/locus.pl?locus=MRT4)*,* [*MSH1*](http://db.yeastgenome.org/cgi-bin/locus.pl?locus=MSH1)*,* [*MSH6*](http://db.yeastgenome.org/cgi-bin/locus.pl?locus=MSH6)*,* [*MTD1*](http://db.yeastgenome.org/cgi-bin/locus.pl?locus=MTD1)*,* [*MTR3*](http://db.yeastgenome.org/cgi-bin/locus.pl?locus=MTR3)*,* [*NAF1*](http://db.yeastgenome.org/cgi-bin/locus.pl?locus=NAF1)*,* [*NAN1*](http://db.yeastgenome.org/cgi-bin/locus.pl?locus=NAN1)*,* [*NCL1*](http://db.yeastgenome.org/cgi-bin/locus.pl?locus=NCL1)*,* [*NCS2*](http://db.yeastgenome.org/cgi-bin/locus.pl?locus=NCS2)*,* [*NHP2*](http://db.yeastgenome.org/cgi-bin/locus.pl?locus=NHP2)*,* [*NIP7*](http://db.yeastgenome.org/cgi-bin/locus.pl?locus=NIP7)*,* [*NMD3*](http://db.yeastgenome.org/cgi-bin/locus.pl?locus=NMD3)*,* [*NOB1*](http://db.yeastgenome.org/cgi-bin/locus.pl?locus=NOB1)*,* [*NOC2*](http://db.yeastgenome.org/cgi-bin/locus.pl?locus=NOC2)*,* [*NOC3*](http://db.yeastgenome.org/cgi-bin/locus.pl?locus=NOC3)*,* [*NOC4*](http://db.yeastgenome.org/cgi-bin/locus.pl?locus=NOC4)*,* [*NOG1*](http://db.yeastgenome.org/cgi-bin/locus.pl?locus=NOG1)*,* [*NOG2*](http://db.yeastgenome.org/cgi-bin/locus.pl?locus=NOG2)*,* [*NOP12*](http://db.yeastgenome.org/cgi-bin/locus.pl?locus=NOP12)*,* [*NOP14*](http://db.yeastgenome.org/cgi-bin/locus.pl?locus=NOP14)*,* [*NOP15*](http://db.yeastgenome.org/cgi-bin/locus.pl?locus=NOP15)*,* [*NOP2*](http://db.yeastgenome.org/cgi-bin/locus.pl?locus=NOP2)*,* [*NOP4*](http://db.yeastgenome.org/cgi-bin/locus.pl?locus=NOP4)*,* [*NOP53*](http://db.yeastgenome.org/cgi-bin/locus.pl?locus=NOP53)*,* [*NOP58*](http://db.yeastgenome.org/cgi-bin/locus.pl?locus=NOP58)*,* [*NOP6*](http://db.yeastgenome.org/cgi-bin/locus.pl?locus=NOP6)*,* [*NOP7*](http://db.yeastgenome.org/cgi-bin/locus.pl?locus=NOP7)*,* [*NOP8*](http://db.yeastgenome.org/cgi-bin/locus.pl?locus=NOP8)*,* [*NOP9*](http://db.yeastgenome.org/cgi-bin/locus.pl?locus=NOP9)*,* [*NSE4*](http://db.yeastgenome.org/cgi-bin/locus.pl?locus=NSE4)*,* [*NSR1*](http://db.yeastgenome.org/cgi-bin/locus.pl?locus=NSR1)*,* [*NUC1*](http://db.yeastgenome.org/cgi-bin/locus.pl?locus=NUC1)*,* [*NUG1*](http://db.yeastgenome.org/cgi-bin/locus.pl?locus=NUG1)*,* [*ORC1*](http://db.yeastgenome.org/cgi-bin/locus.pl?locus=ORC1)*,* [*PHO11*](http://db.yeastgenome.org/cgi-bin/locus.pl?locus=PHO11)*,* [*PHO3*](http://db.yeastgenome.org/cgi-bin/locus.pl?locus=PHO3)*,* [*PHO5*](http://db.yeastgenome.org/cgi-bin/locus.pl?locus=PHO5)*,* [*PLB2*](http://db.yeastgenome.org/cgi-bin/locus.pl?locus=PLB2)*,* [*PMT2*](http://db.yeastgenome.org/cgi-bin/locus.pl?locus=PMT2)*,* [*PNO1*](http://db.yeastgenome.org/cgi-bin/locus.pl?locus=PNO1)*,* [*POL5*](http://db.yeastgenome.org/cgi-bin/locus.pl?locus=POL5)*,* [*POP3*](http://db.yeastgenome.org/cgi-bin/locus.pl?locus=POP3)*,* [*POP6*](http://db.yeastgenome.org/cgi-bin/locus.pl?locus=POP6)*,* [*POP8*](http://db.yeastgenome.org/cgi-bin/locus.pl?locus=POP8)*,* [*PPT1*](http://db.yeastgenome.org/cgi-bin/locus.pl?locus=PPT1)*,* [*PRO1*](http://db.yeastgenome.org/cgi-bin/locus.pl?locus=PRO1)*,* [*PRP19*](http://db.yeastgenome.org/cgi-bin/locus.pl?locus=PRP19)*,* [*PRP24*](http://db.yeastgenome.org/cgi-bin/locus.pl?locus=PRP24)*,* [*PRP43*](http://db.yeastgenome.org/cgi-bin/locus.pl?locus=PRP43)*,* [*PRS1*](http://db.yeastgenome.org/cgi-bin/locus.pl?locus=PRS1)*,* [*PRS2*](http://db.yeastgenome.org/cgi-bin/locus.pl?locus=PRS2)*,* [*PRS4*](http://db.yeastgenome.org/cgi-bin/locus.pl?locus=PRS4)*,* [*PSY4*](http://db.yeastgenome.org/cgi-bin/locus.pl?locus=PSY4)*,* [*PUF6*](http://db.yeastgenome.org/cgi-bin/locus.pl?locus=PUF6)*,* [*PUS1*](http://db.yeastgenome.org/cgi-bin/locus.pl?locus=PUS1)*,* [*PUS4*](http://db.yeastgenome.org/cgi-bin/locus.pl?locus=PUS4)*,* [*PUS7*](http://db.yeastgenome.org/cgi-bin/locus.pl?locus=PUS7)*,* [*PWP1*](http://db.yeastgenome.org/cgi-bin/locus.pl?locus=PWP1)*,* [*PWP2*](http://db.yeastgenome.org/cgi-bin/locus.pl?locus=PWP2)*,* [*PXR1*](http://db.yeastgenome.org/cgi-bin/locus.pl?locus=PXR1)*,* [*RAP1*](http://db.yeastgenome.org/cgi-bin/locus.pl?locus=RAP1)*,* [*RBA50*](http://db.yeastgenome.org/cgi-bin/locus.pl?locus=RBA50)*,* [*RCL1*](http://db.yeastgenome.org/cgi-bin/locus.pl?locus=RCL1)*,* [*RET1*](http://db.yeastgenome.org/cgi-bin/locus.pl?locus=RET1)*,* [*REX4*](http://db.yeastgenome.org/cgi-bin/locus.pl?locus=REX4)*,* [*RHR2*](http://db.yeastgenome.org/cgi-bin/locus.pl?locus=RHR2)*,* [*RIO1*](http://db.yeastgenome.org/cgi-bin/locus.pl?locus=RIO1)*,* [*RIX1*](http://db.yeastgenome.org/cgi-bin/locus.pl?locus=RIX1)*,* [*RKI1*](http://db.yeastgenome.org/cgi-bin/locus.pl?locus=RKI1)*,* [*RLI1*](http://db.yeastgenome.org/cgi-bin/locus.pl?locus=RLI1)*,* [*RLP7*](http://db.yeastgenome.org/cgi-bin/locus.pl?locus=RLP7)*,* [*RMT2*](http://db.yeastgenome.org/cgi-bin/locus.pl?locus=RMT2)*,* [*RNH201*](http://db.yeastgenome.org/cgi-bin/locus.pl?locus=RNH201)*,* [*RNT1*](http://db.yeastgenome.org/cgi-bin/locus.pl?locus=RNT1)*,* [*ROK1*](http://db.yeastgenome.org/cgi-bin/locus.pl?locus=ROK1)*,* [*ROX3*](http://db.yeastgenome.org/cgi-bin/locus.pl?locus=ROX3)*,* [*RPA12*](http://db.yeastgenome.org/cgi-bin/locus.pl?locus=RPA12)*,* [*RPA190*](http://db.yeastgenome.org/cgi-bin/locus.pl?locus=RPA190)*,* [*RPA34*](http://db.yeastgenome.org/cgi-bin/locus.pl?locus=RPA34)*,* [*RPA43*](http://db.yeastgenome.org/cgi-bin/locus.pl?locus=RPA43)*,* [*RPA49*](http://db.yeastgenome.org/cgi-bin/locus.pl?locus=RPA49)*,* [*RPB10*](http://db.yeastgenome.org/cgi-bin/locus.pl?locus=RPB10)*,* [*RPB5*](http://db.yeastgenome.org/cgi-bin/locus.pl?locus=RPB5)*,* [*RPB8*](http://db.yeastgenome.org/cgi-bin/locus.pl?locus=RPB8)*,* [*RPB9*](http://db.yeastgenome.org/cgi-bin/locus.pl?locus=RPB9)*,* [*RPC11*](http://db.yeastgenome.org/cgi-bin/locus.pl?locus=RPC11)*,* [*RPC19*](http://db.yeastgenome.org/cgi-bin/locus.pl?locus=RPC19)*,* [*RPC31*](http://db.yeastgenome.org/cgi-bin/locus.pl?locus=RPC31)*,* [*RPC34*](http://db.yeastgenome.org/cgi-bin/locus.pl?locus=RPC34)*,* [*RPC37*](http://db.yeastgenome.org/cgi-bin/locus.pl?locus=RPC37)*,* [*RPC40*](http://db.yeastgenome.org/cgi-bin/locus.pl?locus=RPC40)*,* [*RPC53*](http://db.yeastgenome.org/cgi-bin/locus.pl?locus=RPC53)*,* [*RPC82*](http://db.yeastgenome.org/cgi-bin/locus.pl?locus=RPC82)*,* [*RPF1*](http://db.yeastgenome.org/cgi-bin/locus.pl?locus=RPF1)*,* [*RPF2*](http://db.yeastgenome.org/cgi-bin/locus.pl?locus=RPF2)*,* [*RPL13A*](http://db.yeastgenome.org/cgi-bin/locus.pl?locus=RPL13A)*,* [*RPL14A*](http://db.yeastgenome.org/cgi-bin/locus.pl?locus=RPL14A)*,* [*RPL14B*](http://db.yeastgenome.org/cgi-bin/locus.pl?locus=RPL14B)*,* [*RPL18B*](http://db.yeastgenome.org/cgi-bin/locus.pl?locus=RPL18B)*,* [*RPL20A*](http://db.yeastgenome.org/cgi-bin/locus.pl?locus=RPL20A)*,* [*RPL21B*](http://db.yeastgenome.org/cgi-bin/locus.pl?locus=RPL21B)*,* [*RPL26A*](http://db.yeastgenome.org/cgi-bin/locus.pl?locus=RPL26A)*,* [*RPL31B*](http://db.yeastgenome.org/cgi-bin/locus.pl?locus=RPL31B)*,* [*RPL33B*](http://db.yeastgenome.org/cgi-bin/locus.pl?locus=RPL33B)*,* [*RPL36A*](http://db.yeastgenome.org/cgi-bin/locus.pl?locus=RPL36A)*,* [*RPL43B*](http://db.yeastgenome.org/cgi-bin/locus.pl?locus=RPL43B)*,* [*RPL7B*](http://db.yeastgenome.org/cgi-bin/locus.pl?locus=RPL7B)*,* [*RPO26*](http://db.yeastgenome.org/cgi-bin/locus.pl?locus=RPO26)*,* [*RPP1*](http://db.yeastgenome.org/cgi-bin/locus.pl?locus=RPP1)*,* [*RPS10B*](http://db.yeastgenome.org/cgi-bin/locus.pl?locus=RPS10B)*,* [*RPS14B*](http://db.yeastgenome.org/cgi-bin/locus.pl?locus=RPS14B)*,* [*RPS19B*](http://db.yeastgenome.org/cgi-bin/locus.pl?locus=RPS19B)*,* [*RPS1A*](http://db.yeastgenome.org/cgi-bin/locus.pl?locus=RPS1A)*,* [*RPS21A*](http://db.yeastgenome.org/cgi-bin/locus.pl?locus=RPS21A)*,* [*RPS21B*](http://db.yeastgenome.org/cgi-bin/locus.pl?locus=RPS21B)*,* [*RPS26B*](http://db.yeastgenome.org/cgi-bin/locus.pl?locus=RPS26B)*,* [*RPS27A*](http://db.yeastgenome.org/cgi-bin/locus.pl?locus=RPS27A)*,* [*RPS29A*](http://db.yeastgenome.org/cgi-bin/locus.pl?locus=RPS29A)*,* [*RPS7A*](http://db.yeastgenome.org/cgi-bin/locus.pl?locus=RPS7A)*,* [*RPS7B*](http://db.yeastgenome.org/cgi-bin/locus.pl?locus=RPS7B)*,* [*RRM3*](http://db.yeastgenome.org/cgi-bin/locus.pl?locus=RRM3)*,* [*RRN11*](http://db.yeastgenome.org/cgi-bin/locus.pl?locus=RRN11)*,* [*RRN7*](http://db.yeastgenome.org/cgi-bin/locus.pl?locus=RRN7)*,* [*RRP1*](http://db.yeastgenome.org/cgi-bin/locus.pl?locus=RRP1)*,* [*RRP12*](http://db.yeastgenome.org/cgi-bin/locus.pl?locus=RRP12)*,* [*RRP15*](http://db.yeastgenome.org/cgi-bin/locus.pl?locus=RRP15)*,* [*RRP17*](http://db.yeastgenome.org/cgi-bin/locus.pl?locus=RRP17)*,* [*RRP3*](http://db.yeastgenome.org/cgi-bin/locus.pl?locus=RRP3)*, RRP36,* [*RRP40*](http://db.yeastgenome.org/cgi-bin/locus.pl?locus=RRP40)*,* [*RRP43*](http://db.yeastgenome.org/cgi-bin/locus.pl?locus=RRP43)*,* [*RRP5*](http://db.yeastgenome.org/cgi-bin/locus.pl?locus=RRP5)*,* [*RRP6*](http://db.yeastgenome.org/cgi-bin/locus.pl?locus=RRP6)*,* [*RRP8*](http://db.yeastgenome.org/cgi-bin/locus.pl?locus=RRP8)*,* [*RRP9*](http://db.yeastgenome.org/cgi-bin/locus.pl?locus=RRP9)*,* [*RRS1*](http://db.yeastgenome.org/cgi-bin/locus.pl?locus=RRS1)*,* [*RSA1*](http://db.yeastgenome.org/cgi-bin/locus.pl?locus=RSA1)*,* [*RSA3*](http://db.yeastgenome.org/cgi-bin/locus.pl?locus=RSA3)*,* [*RSA4*](http://db.yeastgenome.org/cgi-bin/locus.pl?locus=RSA4)*,* [*RVB1*](http://db.yeastgenome.org/cgi-bin/locus.pl?locus=RVB1)*,* [*SAN1*](http://db.yeastgenome.org/cgi-bin/locus.pl?locus=SAN1)*,* [*SAS10*](http://db.yeastgenome.org/cgi-bin/locus.pl?locus=SAS10)*,* [*SCC2*](http://db.yeastgenome.org/cgi-bin/locus.pl?locus=SCC2)*,* [*SCH9*](http://db.yeastgenome.org/cgi-bin/locus.pl?locus=SCH9)*,* [*SCS7*](http://db.yeastgenome.org/cgi-bin/locus.pl?locus=SCS7)*,* [*SDA1*](http://db.yeastgenome.org/cgi-bin/locus.pl?locus=SDA1)*,* [*SEN34*](http://db.yeastgenome.org/cgi-bin/locus.pl?locus=SEN34)*,* [*SFG1*](http://db.yeastgenome.org/cgi-bin/locus.pl?locus=SFG1)*,* [*SHQ1*](http://db.yeastgenome.org/cgi-bin/locus.pl?locus=SHQ1)*,* [*SIK1*](http://db.yeastgenome.org/cgi-bin/locus.pl?locus=SIK1)*,* [*SIR2*](http://db.yeastgenome.org/cgi-bin/locus.pl?locus=SIR2)*,* [*SLI15*](http://db.yeastgenome.org/cgi-bin/locus.pl?locus=SLI15)*,* [*SLN1*](http://db.yeastgenome.org/cgi-bin/locus.pl?locus=SLN1)*,* [*SLX9*](http://db.yeastgenome.org/cgi-bin/locus.pl?locus=SLX9)*,* [*SMI1*](http://db.yeastgenome.org/cgi-bin/locus.pl?locus=SMI1)*,* [*SNU13*](http://db.yeastgenome.org/cgi-bin/locus.pl?locus=SNU13)*,* [*SOF1*](http://db.yeastgenome.org/cgi-bin/locus.pl?locus=SOF1)*,* [*SPB1*](http://db.yeastgenome.org/cgi-bin/locus.pl?locus=SPB1)*,* [*SPB4*](http://db.yeastgenome.org/cgi-bin/locus.pl?locus=SPB4)*,* [*SPE4*](http://db.yeastgenome.org/cgi-bin/locus.pl?locus=SPE4)*,* [*SQT1*](http://db.yeastgenome.org/cgi-bin/locus.pl?locus=SQT1)*,* [*SRO9*](http://db.yeastgenome.org/cgi-bin/locus.pl?locus=SRO9)*,* [*SSF1*](http://db.yeastgenome.org/cgi-bin/locus.pl?locus=SSF1)*,* [*SSF2*](http://db.yeastgenome.org/cgi-bin/locus.pl?locus=SSF2)*,* [*STE12*](http://db.yeastgenome.org/cgi-bin/locus.pl?locus=STE12)*,* [*SUR2*](http://db.yeastgenome.org/cgi-bin/locus.pl?locus=SUR2)*,* [*SUR4*](http://db.yeastgenome.org/cgi-bin/locus.pl?locus=SUR4)*,* [*SUT1*](http://db.yeastgenome.org/cgi-bin/locus.pl?locus=SUT1)*,* [*SUT2*](http://db.yeastgenome.org/cgi-bin/locus.pl?locus=SUT2)*,* [*SUV3*](http://db.yeastgenome.org/cgi-bin/locus.pl?locus=SUV3)*,* [*SVF1*](http://db.yeastgenome.org/cgi-bin/locus.pl?locus=SVF1)*,* [*SWI5*](http://db.yeastgenome.org/cgi-bin/locus.pl?locus=SWI5)*,* [*TAD3*](http://db.yeastgenome.org/cgi-bin/locus.pl?locus=TAD3)*,* [*THI21*](http://db.yeastgenome.org/cgi-bin/locus.pl?locus=THI21)*,* [*THP2*](http://db.yeastgenome.org/cgi-bin/locus.pl?locus=THP2)*,* [*TIF2*](http://db.yeastgenome.org/cgi-bin/locus.pl?locus=TIF2)*,* [*TIF3*](http://db.yeastgenome.org/cgi-bin/locus.pl?locus=TIF3)*,* [*TIF35*](http://db.yeastgenome.org/cgi-bin/locus.pl?locus=TIF35)*,* [*TIF4631*](http://db.yeastgenome.org/cgi-bin/locus.pl?locus=TIF4631)*,* [*TOP1*](http://db.yeastgenome.org/cgi-bin/locus.pl?locus=TOP1)*,* [*TOS4*](http://db.yeastgenome.org/cgi-bin/locus.pl?locus=TOS4)*,* [*TPA1*](http://db.yeastgenome.org/cgi-bin/locus.pl?locus=TPA1)*,* [*TRF5*](http://db.yeastgenome.org/cgi-bin/locus.pl?locus=TRF5)*,* [*TRM1*](http://db.yeastgenome.org/cgi-bin/locus.pl?locus=TRM1)*,* [*TRM10*](http://db.yeastgenome.org/cgi-bin/locus.pl?locus=TRM10)*,* [*TRM11*](http://db.yeastgenome.org/cgi-bin/locus.pl?locus=TRM11)*,* [*TRM112*](http://db.yeastgenome.org/cgi-bin/locus.pl?locus=TRM112)*,* [*TRM13*](http://db.yeastgenome.org/cgi-bin/locus.pl?locus=TRM13)*,* [*TRM2*](http://db.yeastgenome.org/cgi-bin/locus.pl?locus=TRM2)*,* [*TRM3*](http://db.yeastgenome.org/cgi-bin/locus.pl?locus=TRM3)*,* [*TRM7*](http://db.yeastgenome.org/cgi-bin/locus.pl?locus=TRM7)*,* [*TRM8*](http://db.yeastgenome.org/cgi-bin/locus.pl?locus=TRM8)*,* [*TRM82*](http://db.yeastgenome.org/cgi-bin/locus.pl?locus=TRM82)*,* [*TRM9*](http://db.yeastgenome.org/cgi-bin/locus.pl?locus=TRM9)*,* [*TSC10*](http://db.yeastgenome.org/cgi-bin/locus.pl?locus=TSC10)*,* [*TSR1*](http://db.yeastgenome.org/cgi-bin/locus.pl?locus=TSR1)*,* [*TSR2*](http://db.yeastgenome.org/cgi-bin/locus.pl?locus=TSR2)*,* [*TYW1*](http://db.yeastgenome.org/cgi-bin/locus.pl?locus=TYW1)*,* [*UBC9*](http://db.yeastgenome.org/cgi-bin/locus.pl?locus=UBC9)*,* [*UBP10*](http://db.yeastgenome.org/cgi-bin/locus.pl?locus=UBP10)*,* [*UNG1*](http://db.yeastgenome.org/cgi-bin/locus.pl?locus=UNG1)*,* [*URA4*](http://db.yeastgenome.org/cgi-bin/locus.pl?locus=URA4)*,* [*URA5*](http://db.yeastgenome.org/cgi-bin/locus.pl?locus=URA5)*,* [*URA7*](http://db.yeastgenome.org/cgi-bin/locus.pl?locus=URA7)*,* [*URB1*](http://db.yeastgenome.org/cgi-bin/locus.pl?locus=URB1)*,* [*URB2*](http://db.yeastgenome.org/cgi-bin/locus.pl?locus=URB2)*,* [*URK1*](http://db.yeastgenome.org/cgi-bin/locus.pl?locus=URK1)*,* [*UTP10*](http://db.yeastgenome.org/cgi-bin/locus.pl?locus=UTP10)*,* [*UTP11*](http://db.yeastgenome.org/cgi-bin/locus.pl?locus=UTP11)*,* [*UTP13*](http://db.yeastgenome.org/cgi-bin/locus.pl?locus=UTP13)*,* [*UTP14*](http://db.yeastgenome.org/cgi-bin/locus.pl?locus=UTP14)*,* [*UTP15*](http://db.yeastgenome.org/cgi-bin/locus.pl?locus=UTP15)*,* [*UTP18*](http://db.yeastgenome.org/cgi-bin/locus.pl?locus=UTP18)*,* [*UTP21*](http://db.yeastgenome.org/cgi-bin/locus.pl?locus=UTP21)*,* [*UTP22*](http://db.yeastgenome.org/cgi-bin/locus.pl?locus=UTP22)*,* [*UTP23*](http://db.yeastgenome.org/cgi-bin/locus.pl?locus=UTP23)*,* [*UTP30*](http://db.yeastgenome.org/cgi-bin/locus.pl?locus=UTP30)*,* [*UTP4*](http://db.yeastgenome.org/cgi-bin/locus.pl?locus=UTP4)*,* [*UTP5*](http://db.yeastgenome.org/cgi-bin/locus.pl?locus=UTP5)*,* [*UTP7*](http://db.yeastgenome.org/cgi-bin/locus.pl?locus=UTP7)*,* [*UTP8*](http://db.yeastgenome.org/cgi-bin/locus.pl?locus=UTP8)*,* [*UTP9*](http://db.yeastgenome.org/cgi-bin/locus.pl?locus=UTP9)*,* [*VRG4*](http://db.yeastgenome.org/cgi-bin/locus.pl?locus=VRG4)*,* [*WRS1*](http://db.yeastgenome.org/cgi-bin/locus.pl?locus=WRS1)*,* [*YAR1*](http://db.yeastgenome.org/cgi-bin/locus.pl?locus=YAR1)*,* [*YEF3*](http://db.yeastgenome.org/cgi-bin/locus.pl?locus=YEF3)*,* [*YHR020W*](http://db.yeastgenome.org/cgi-bin/locus.pl?locus=YHR020W)*,* [*YML082W*](http://db.yeastgenome.org/cgi-bin/locus.pl?locus=YML082W)*,* [*YNL247W*](http://db.yeastgenome.org/cgi-bin/locus.pl?locus=YNL247W)*,* [*YOX1*](http://db.yeastgenome.org/cgi-bin/locus.pl?locus=YOX1)*,* [*YRF1-6*](http://db.yeastgenome.org/cgi-bin/locus.pl?locus=YRF1-6)*,* [*YVH1*](http://db.yeastgenome.org/cgi-bin/locus.pl?locus=YVH1)*,* [*ZRC1*](http://db.yeastgenome.org/cgi-bin/locus.pl?locus=ZRC1) |
| Ribosome assembly | 33 out of 558 genes, 5.9% | 3.53e-17 | [*BMS1*](http://db.yeastgenome.org/cgi-bin/locus.pl?locus=BMS1)*,* [*BRX1*](http://db.yeastgenome.org/cgi-bin/locus.pl?locus=BRX1)*,* [*DBP10*](http://db.yeastgenome.org/cgi-bin/locus.pl?locus=DBP10)*,* [*DBP3*](http://db.yeastgenome.org/cgi-bin/locus.pl?locus=DBP3)*,* [*DBP6*](http://db.yeastgenome.org/cgi-bin/locus.pl?locus=DBP6)*,* [*DBP7*](http://db.yeastgenome.org/cgi-bin/locus.pl?locus=DBP7)*,* [*DBP9*](http://db.yeastgenome.org/cgi-bin/locus.pl?locus=DBP9)*,* [*DRS1*](http://db.yeastgenome.org/cgi-bin/locus.pl?locus=DRS1)*,* [*IPI1*](http://db.yeastgenome.org/cgi-bin/locus.pl?locus=IPI1)*,* [*IPI3*](http://db.yeastgenome.org/cgi-bin/locus.pl?locus=IPI3)*,* [*MAK11*](http://db.yeastgenome.org/cgi-bin/locus.pl?locus=MAK11)*,* [*MAK21*](http://db.yeastgenome.org/cgi-bin/locus.pl?locus=MAK21)*,* [*NIP7*](http://db.yeastgenome.org/cgi-bin/locus.pl?locus=NIP7)*,* [*NMD3*](http://db.yeastgenome.org/cgi-bin/locus.pl?locus=NMD3)*,* [*NOC2*](http://db.yeastgenome.org/cgi-bin/locus.pl?locus=NOC2)*,* [*NOG2*](http://db.yeastgenome.org/cgi-bin/locus.pl?locus=NOG2)*,* [*NOP15*](http://db.yeastgenome.org/cgi-bin/locus.pl?locus=NOP15)*,* [*NOP8*](http://db.yeastgenome.org/cgi-bin/locus.pl?locus=NOP8)*,* [*NSR1*](http://db.yeastgenome.org/cgi-bin/locus.pl?locus=NSR1)*,* [*REX4*](http://db.yeastgenome.org/cgi-bin/locus.pl?locus=REX4)*,* [*RIX1*](http://db.yeastgenome.org/cgi-bin/locus.pl?locus=RIX1)*,* [*RPF1*](http://db.yeastgenome.org/cgi-bin/locus.pl?locus=RPF1)*,* [*RPF2*](http://db.yeastgenome.org/cgi-bin/locus.pl?locus=RPF2)*,* [*RPS14B*](http://db.yeastgenome.org/cgi-bin/locus.pl?locus=RPS14B)*,* [*RRP40*](http://db.yeastgenome.org/cgi-bin/locus.pl?locus=RRP40)*,* [*RSA1*](http://db.yeastgenome.org/cgi-bin/locus.pl?locus=RSA1)*,* [*RSA3*](http://db.yeastgenome.org/cgi-bin/locus.pl?locus=RSA3)*,* [*RSA4*](http://db.yeastgenome.org/cgi-bin/locus.pl?locus=RSA4)*,* [*SDA1*](http://db.yeastgenome.org/cgi-bin/locus.pl?locus=SDA1)*,* [*SPB4*](http://db.yeastgenome.org/cgi-bin/locus.pl?locus=SPB4)*,* [*SQT1*](http://db.yeastgenome.org/cgi-bin/locus.pl?locus=SQT1)*,* [*SSF1*](http://db.yeastgenome.org/cgi-bin/locus.pl?locus=SSF1)*,* [*SSF2*](http://db.yeastgenome.org/cgi-bin/locus.pl?locus=SSF2) |
| Biopolymer metabolic process | 266 out of 558 genes, 47.7% | 2.39e-16 | [*AIR1*](http://db.yeastgenome.org/cgi-bin/locus.pl?locus=AIR1)*,* [*ALK1*](http://db.yeastgenome.org/cgi-bin/locus.pl?locus=ALK1)*,* [*ASH1*](http://db.yeastgenome.org/cgi-bin/locus.pl?locus=ASH1)*,* [*BCD1*](http://db.yeastgenome.org/cgi-bin/locus.pl?locus=BCD1)*,* [*BMS1*](http://db.yeastgenome.org/cgi-bin/locus.pl?locus=BMS1)*,* [*BST1*](http://db.yeastgenome.org/cgi-bin/locus.pl?locus=BST1)*,* [*BUD21*](http://db.yeastgenome.org/cgi-bin/locus.pl?locus=BUD21)*,* [*BUD32*](http://db.yeastgenome.org/cgi-bin/locus.pl?locus=BUD32)*,* [*CBF5*](http://db.yeastgenome.org/cgi-bin/locus.pl?locus=CBF5)*,* [*CDC14*](http://db.yeastgenome.org/cgi-bin/locus.pl?locus=CDC14)*,* [*CDC20*](http://db.yeastgenome.org/cgi-bin/locus.pl?locus=CDC20)*,* [*CDC47*](http://db.yeastgenome.org/cgi-bin/locus.pl?locus=CDC47)*,* [*CGR1*](http://db.yeastgenome.org/cgi-bin/locus.pl?locus=CGR1)*,* [*CIC1*](http://db.yeastgenome.org/cgi-bin/locus.pl?locus=CIC1)*,* [*CLA4*](http://db.yeastgenome.org/cgi-bin/locus.pl?locus=CLA4)*, CMS1,* [*CSM3*](http://db.yeastgenome.org/cgi-bin/locus.pl?locus=CSM3)*,* [*CUS1*](http://db.yeastgenome.org/cgi-bin/locus.pl?locus=CUS1)*,* [*CWC2*](http://db.yeastgenome.org/cgi-bin/locus.pl?locus=CWC2)*,* [*DBP10*](http://db.yeastgenome.org/cgi-bin/locus.pl?locus=DBP10)*,* [*DBP2*](http://db.yeastgenome.org/cgi-bin/locus.pl?locus=DBP2)*,* [*DBP3*](http://db.yeastgenome.org/cgi-bin/locus.pl?locus=DBP3)*,* [*DBP6*](http://db.yeastgenome.org/cgi-bin/locus.pl?locus=DBP6)*,* [*DBP7*](http://db.yeastgenome.org/cgi-bin/locus.pl?locus=DBP7)*,* [*DBP8*](http://db.yeastgenome.org/cgi-bin/locus.pl?locus=DBP8)*,* [*DBP9*](http://db.yeastgenome.org/cgi-bin/locus.pl?locus=DBP9)*,* [*DIM1*](http://db.yeastgenome.org/cgi-bin/locus.pl?locus=DIM1)*,* [*DIP2*](http://db.yeastgenome.org/cgi-bin/locus.pl?locus=DIP2)*,* [*DPB2*](http://db.yeastgenome.org/cgi-bin/locus.pl?locus=DPB2)*,* [*DPH2*](http://db.yeastgenome.org/cgi-bin/locus.pl?locus=DPH2)*,* [*DRS1*](http://db.yeastgenome.org/cgi-bin/locus.pl?locus=DRS1)*,* [*DUS1*](http://db.yeastgenome.org/cgi-bin/locus.pl?locus=DUS1)*,* [*DUS3*](http://db.yeastgenome.org/cgi-bin/locus.pl?locus=DUS3)*,* [*DUS4*](http://db.yeastgenome.org/cgi-bin/locus.pl?locus=DUS4)*,* [*DYS1*](http://db.yeastgenome.org/cgi-bin/locus.pl?locus=DYS1)*,* [*EBP2*](http://db.yeastgenome.org/cgi-bin/locus.pl?locus=EBP2)*,* [*ECM16*](http://db.yeastgenome.org/cgi-bin/locus.pl?locus=ECM16)*,* [*EFB1*](http://db.yeastgenome.org/cgi-bin/locus.pl?locus=EFB1)*, EFG1,* [*ELP2*](http://db.yeastgenome.org/cgi-bin/locus.pl?locus=ELP2)*,* [*ELP3*](http://db.yeastgenome.org/cgi-bin/locus.pl?locus=ELP3)*,* [*EMG1*](http://db.yeastgenome.org/cgi-bin/locus.pl?locus=EMG1)*,* [*ENP1*](http://db.yeastgenome.org/cgi-bin/locus.pl?locus=ENP1)*,* [*ENP2*](http://db.yeastgenome.org/cgi-bin/locus.pl?locus=ENP2)*,* [*ERB1*](http://db.yeastgenome.org/cgi-bin/locus.pl?locus=ERB1)*,* [*ESF1*](http://db.yeastgenome.org/cgi-bin/locus.pl?locus=ESF1)*,* [*ESF2*](http://db.yeastgenome.org/cgi-bin/locus.pl?locus=ESF2)*,* [*EXG1*](http://db.yeastgenome.org/cgi-bin/locus.pl?locus=EXG1)*,* [*FAA4*](http://db.yeastgenome.org/cgi-bin/locus.pl?locus=FAA4)*,* [*FAF1*](http://db.yeastgenome.org/cgi-bin/locus.pl?locus=FAF1)*,* [*FAL1*](http://db.yeastgenome.org/cgi-bin/locus.pl?locus=FAL1)*,* [*FAP7*](http://db.yeastgenome.org/cgi-bin/locus.pl?locus=FAP7)*,* [*FCF2*](http://db.yeastgenome.org/cgi-bin/locus.pl?locus=FCF2)*,* [*FKH1*](http://db.yeastgenome.org/cgi-bin/locus.pl?locus=FKH1)*,* [*FPR4*](http://db.yeastgenome.org/cgi-bin/locus.pl?locus=FPR4)*,* [*FYV7*](http://db.yeastgenome.org/cgi-bin/locus.pl?locus=FYV7)*,* [*GAR1*](http://db.yeastgenome.org/cgi-bin/locus.pl?locus=GAR1)*,* [*GCD10*](http://db.yeastgenome.org/cgi-bin/locus.pl?locus=GCD10)*,* [*GCD14*](http://db.yeastgenome.org/cgi-bin/locus.pl?locus=GCD14)*,* [*GCR1*](http://db.yeastgenome.org/cgi-bin/locus.pl?locus=GCR1)*,* [*GCR2*](http://db.yeastgenome.org/cgi-bin/locus.pl?locus=GCR2)*,* [*GLN4*](http://db.yeastgenome.org/cgi-bin/locus.pl?locus=GLN4)*,* [*GPI13*](http://db.yeastgenome.org/cgi-bin/locus.pl?locus=GPI13)*,* [*GRC3*](http://db.yeastgenome.org/cgi-bin/locus.pl?locus=GRC3)*,* [*HAS1*](http://db.yeastgenome.org/cgi-bin/locus.pl?locus=HAS1)*,* [*HCA4*](http://db.yeastgenome.org/cgi-bin/locus.pl?locus=HCA4)*,* [*HHF2*](http://db.yeastgenome.org/cgi-bin/locus.pl?locus=HHF2)*,* [*HHT1*](http://db.yeastgenome.org/cgi-bin/locus.pl?locus=HHT1)*,* [*HIR2*](http://db.yeastgenome.org/cgi-bin/locus.pl?locus=HIR2)*,* [*HMT1*](http://db.yeastgenome.org/cgi-bin/locus.pl?locus=HMT1)*,* [*HST3*](http://db.yeastgenome.org/cgi-bin/locus.pl?locus=HST3)*,* [*HTA1*](http://db.yeastgenome.org/cgi-bin/locus.pl?locus=HTA1)*,* [*HTA2*](http://db.yeastgenome.org/cgi-bin/locus.pl?locus=HTA2)*,* [*HTB1*](http://db.yeastgenome.org/cgi-bin/locus.pl?locus=HTB1)*,* [*HTB2*](http://db.yeastgenome.org/cgi-bin/locus.pl?locus=HTB2)*,* [*HTZ1*](http://db.yeastgenome.org/cgi-bin/locus.pl?locus=HTZ1)*,* [*IFH1*](http://db.yeastgenome.org/cgi-bin/locus.pl?locus=IFH1)*,* [*IMP3*](http://db.yeastgenome.org/cgi-bin/locus.pl?locus=IMP3)*,* [*INO2*](http://db.yeastgenome.org/cgi-bin/locus.pl?locus=INO2)*,* [*IPI1*](http://db.yeastgenome.org/cgi-bin/locus.pl?locus=IPI1)*,* [*IPI3*](http://db.yeastgenome.org/cgi-bin/locus.pl?locus=IPI3)*,* [*JJJ3*](http://db.yeastgenome.org/cgi-bin/locus.pl?locus=JJJ3)*,* [*KAE1*](http://db.yeastgenome.org/cgi-bin/locus.pl?locus=KAE1)*,* [*KRR1*](http://db.yeastgenome.org/cgi-bin/locus.pl?locus=KRR1)*,* [*KTI12*](http://db.yeastgenome.org/cgi-bin/locus.pl?locus=KTI12)*,* [*KTR5*](http://db.yeastgenome.org/cgi-bin/locus.pl?locus=KTR5)*,* [*LCP5*](http://db.yeastgenome.org/cgi-bin/locus.pl?locus=LCP5)*,* [*MAK11*](http://db.yeastgenome.org/cgi-bin/locus.pl?locus=MAK11)*,* [*MAK16*](http://db.yeastgenome.org/cgi-bin/locus.pl?locus=MAK16)*,* [*MAK3*](http://db.yeastgenome.org/cgi-bin/locus.pl?locus=MAK3)*,* [*MES1*](http://db.yeastgenome.org/cgi-bin/locus.pl?locus=MES1)*,* [*MIS1*](http://db.yeastgenome.org/cgi-bin/locus.pl?locus=MIS1)*,* [*MNN2*](http://db.yeastgenome.org/cgi-bin/locus.pl?locus=MNN2)*,* [*MOT1*](http://db.yeastgenome.org/cgi-bin/locus.pl?locus=MOT1)*,* [*MPP10*](http://db.yeastgenome.org/cgi-bin/locus.pl?locus=MPP10)*,* [*MRD1*](http://db.yeastgenome.org/cgi-bin/locus.pl?locus=MRD1)*,* [*MRT4*](http://db.yeastgenome.org/cgi-bin/locus.pl?locus=MRT4)*,* [*MSH1*](http://db.yeastgenome.org/cgi-bin/locus.pl?locus=MSH1)*,* [*MSH6*](http://db.yeastgenome.org/cgi-bin/locus.pl?locus=MSH6)*,* [*MTR3*](http://db.yeastgenome.org/cgi-bin/locus.pl?locus=MTR3)*,* [*NAF1*](http://db.yeastgenome.org/cgi-bin/locus.pl?locus=NAF1)*,* [*NAN1*](http://db.yeastgenome.org/cgi-bin/locus.pl?locus=NAN1)*,* [*NCL1*](http://db.yeastgenome.org/cgi-bin/locus.pl?locus=NCL1)*,* [*NCS2*](http://db.yeastgenome.org/cgi-bin/locus.pl?locus=NCS2)*,* [*NHP2*](http://db.yeastgenome.org/cgi-bin/locus.pl?locus=NHP2)*,* [*NIP7*](http://db.yeastgenome.org/cgi-bin/locus.pl?locus=NIP7)*,* [*NOB1*](http://db.yeastgenome.org/cgi-bin/locus.pl?locus=NOB1)*,* [*NOC3*](http://db.yeastgenome.org/cgi-bin/locus.pl?locus=NOC3)*,* [*NOC4*](http://db.yeastgenome.org/cgi-bin/locus.pl?locus=NOC4)*,* [*NOG1*](http://db.yeastgenome.org/cgi-bin/locus.pl?locus=NOG1)*,* [*NOP12*](http://db.yeastgenome.org/cgi-bin/locus.pl?locus=NOP12)*,* [*NOP14*](http://db.yeastgenome.org/cgi-bin/locus.pl?locus=NOP14)*,* [*NOP2*](http://db.yeastgenome.org/cgi-bin/locus.pl?locus=NOP2)*,* [*NOP4*](http://db.yeastgenome.org/cgi-bin/locus.pl?locus=NOP4)*,* [*NOP53*](http://db.yeastgenome.org/cgi-bin/locus.pl?locus=NOP53)*,* [*NOP58*](http://db.yeastgenome.org/cgi-bin/locus.pl?locus=NOP58)*,* [*NOP6*](http://db.yeastgenome.org/cgi-bin/locus.pl?locus=NOP6)*,* [*NOP7*](http://db.yeastgenome.org/cgi-bin/locus.pl?locus=NOP7)*,* [*NOP8*](http://db.yeastgenome.org/cgi-bin/locus.pl?locus=NOP8)*,* [*NOP9*](http://db.yeastgenome.org/cgi-bin/locus.pl?locus=NOP9)*,* [*NSE4*](http://db.yeastgenome.org/cgi-bin/locus.pl?locus=NSE4)*,* [*NSR1*](http://db.yeastgenome.org/cgi-bin/locus.pl?locus=NSR1)*,* [*NUC1*](http://db.yeastgenome.org/cgi-bin/locus.pl?locus=NUC1)*,* [*NUG1*](http://db.yeastgenome.org/cgi-bin/locus.pl?locus=NUG1)*,* [*ORC1*](http://db.yeastgenome.org/cgi-bin/locus.pl?locus=ORC1)*,* [*PMT2*](http://db.yeastgenome.org/cgi-bin/locus.pl?locus=PMT2)*,* [*PNO1*](http://db.yeastgenome.org/cgi-bin/locus.pl?locus=PNO1)*,* [*POL5*](http://db.yeastgenome.org/cgi-bin/locus.pl?locus=POL5)*,* [*POP3*](http://db.yeastgenome.org/cgi-bin/locus.pl?locus=POP3)*,* [*POP6*](http://db.yeastgenome.org/cgi-bin/locus.pl?locus=POP6)*,* [*POP8*](http://db.yeastgenome.org/cgi-bin/locus.pl?locus=POP8)*,* [*PPT1*](http://db.yeastgenome.org/cgi-bin/locus.pl?locus=PPT1)*,* [*PRP19*](http://db.yeastgenome.org/cgi-bin/locus.pl?locus=PRP19)*,* [*PRP24*](http://db.yeastgenome.org/cgi-bin/locus.pl?locus=PRP24)*,* [*PRP43*](http://db.yeastgenome.org/cgi-bin/locus.pl?locus=PRP43)*,* [*PSY4*](http://db.yeastgenome.org/cgi-bin/locus.pl?locus=PSY4)*,* [*PUF6*](http://db.yeastgenome.org/cgi-bin/locus.pl?locus=PUF6)*,* [*PUS1*](http://db.yeastgenome.org/cgi-bin/locus.pl?locus=PUS1)*,* [*PUS4*](http://db.yeastgenome.org/cgi-bin/locus.pl?locus=PUS4)*,* [*PUS7*](http://db.yeastgenome.org/cgi-bin/locus.pl?locus=PUS7)*,* [*PWP1*](http://db.yeastgenome.org/cgi-bin/locus.pl?locus=PWP1)*,* [*PWP2*](http://db.yeastgenome.org/cgi-bin/locus.pl?locus=PWP2)*,* [*PXR1*](http://db.yeastgenome.org/cgi-bin/locus.pl?locus=PXR1)*,* [*RAP1*](http://db.yeastgenome.org/cgi-bin/locus.pl?locus=RAP1)*,* [*RBA50*](http://db.yeastgenome.org/cgi-bin/locus.pl?locus=RBA50)*,* [*RCL1*](http://db.yeastgenome.org/cgi-bin/locus.pl?locus=RCL1)*,* [*RET1*](http://db.yeastgenome.org/cgi-bin/locus.pl?locus=RET1)*,* [*REX4*](http://db.yeastgenome.org/cgi-bin/locus.pl?locus=REX4)*,* [*RIO1*](http://db.yeastgenome.org/cgi-bin/locus.pl?locus=RIO1)*,* [*RIX1*](http://db.yeastgenome.org/cgi-bin/locus.pl?locus=RIX1)*,* [*RLP7*](http://db.yeastgenome.org/cgi-bin/locus.pl?locus=RLP7)*,* [*RMT2*](http://db.yeastgenome.org/cgi-bin/locus.pl?locus=RMT2)*,* [*RNH201*](http://db.yeastgenome.org/cgi-bin/locus.pl?locus=RNH201)*,* [*RNT1*](http://db.yeastgenome.org/cgi-bin/locus.pl?locus=RNT1)*,* [*ROK1*](http://db.yeastgenome.org/cgi-bin/locus.pl?locus=ROK1)*,* [*ROX3*](http://db.yeastgenome.org/cgi-bin/locus.pl?locus=ROX3)*,* [*RPA12*](http://db.yeastgenome.org/cgi-bin/locus.pl?locus=RPA12)*,* [*RPA190*](http://db.yeastgenome.org/cgi-bin/locus.pl?locus=RPA190)*,* [*RPA34*](http://db.yeastgenome.org/cgi-bin/locus.pl?locus=RPA34)*,* [*RPA43*](http://db.yeastgenome.org/cgi-bin/locus.pl?locus=RPA43)*,* [*RPA49*](http://db.yeastgenome.org/cgi-bin/locus.pl?locus=RPA49)*,* [*RPB10*](http://db.yeastgenome.org/cgi-bin/locus.pl?locus=RPB10)*,* [*RPB5*](http://db.yeastgenome.org/cgi-bin/locus.pl?locus=RPB5)*,* [*RPB8*](http://db.yeastgenome.org/cgi-bin/locus.pl?locus=RPB8)*,* [*RPB9*](http://db.yeastgenome.org/cgi-bin/locus.pl?locus=RPB9)*,* [*RPC11*](http://db.yeastgenome.org/cgi-bin/locus.pl?locus=RPC11)*,* [*RPC19*](http://db.yeastgenome.org/cgi-bin/locus.pl?locus=RPC19)*,* [*RPC31*](http://db.yeastgenome.org/cgi-bin/locus.pl?locus=RPC31)*,* [*RPC34*](http://db.yeastgenome.org/cgi-bin/locus.pl?locus=RPC34)*,* [*RPC37*](http://db.yeastgenome.org/cgi-bin/locus.pl?locus=RPC37)*,* [*RPC40*](http://db.yeastgenome.org/cgi-bin/locus.pl?locus=RPC40)*,* [*RPC53*](http://db.yeastgenome.org/cgi-bin/locus.pl?locus=RPC53)*,* [*RPC82*](http://db.yeastgenome.org/cgi-bin/locus.pl?locus=RPC82)*,* [*RPF1*](http://db.yeastgenome.org/cgi-bin/locus.pl?locus=RPF1)*,* [*RPF2*](http://db.yeastgenome.org/cgi-bin/locus.pl?locus=RPF2)*,* [*RPO26*](http://db.yeastgenome.org/cgi-bin/locus.pl?locus=RPO26)*,* [*RPP1*](http://db.yeastgenome.org/cgi-bin/locus.pl?locus=RPP1)*,* [*RPS14B*](http://db.yeastgenome.org/cgi-bin/locus.pl?locus=RPS14B)*,* [*RPS19B*](http://db.yeastgenome.org/cgi-bin/locus.pl?locus=RPS19B)*,* [*RRM3*](http://db.yeastgenome.org/cgi-bin/locus.pl?locus=RRM3)*,* [*RRN11*](http://db.yeastgenome.org/cgi-bin/locus.pl?locus=RRN11)*,* [*RRN7*](http://db.yeastgenome.org/cgi-bin/locus.pl?locus=RRN7)*,* [*RRP1*](http://db.yeastgenome.org/cgi-bin/locus.pl?locus=RRP1)*,* [*RRP12*](http://db.yeastgenome.org/cgi-bin/locus.pl?locus=RRP12)*,* [*RRP15*](http://db.yeastgenome.org/cgi-bin/locus.pl?locus=RRP15)*,* [*RRP17*](http://db.yeastgenome.org/cgi-bin/locus.pl?locus=RRP17)*,* [*RRP3*](http://db.yeastgenome.org/cgi-bin/locus.pl?locus=RRP3)*, RRP36,* [*RRP40*](http://db.yeastgenome.org/cgi-bin/locus.pl?locus=RRP40)*,* [*RRP43*](http://db.yeastgenome.org/cgi-bin/locus.pl?locus=RRP43)*,* [*RRP5*](http://db.yeastgenome.org/cgi-bin/locus.pl?locus=RRP5)*,* [*RRP6*](http://db.yeastgenome.org/cgi-bin/locus.pl?locus=RRP6)*,* [*RRP8*](http://db.yeastgenome.org/cgi-bin/locus.pl?locus=RRP8)*,* [*RRP9*](http://db.yeastgenome.org/cgi-bin/locus.pl?locus=RRP9)*,* [*RRS1*](http://db.yeastgenome.org/cgi-bin/locus.pl?locus=RRS1)*,* [*RVB1*](http://db.yeastgenome.org/cgi-bin/locus.pl?locus=RVB1)*,* [*SAN1*](http://db.yeastgenome.org/cgi-bin/locus.pl?locus=SAN1)*,* [*SAS10*](http://db.yeastgenome.org/cgi-bin/locus.pl?locus=SAS10)*,* [*SCC2*](http://db.yeastgenome.org/cgi-bin/locus.pl?locus=SCC2)*,* [*SCH9*](http://db.yeastgenome.org/cgi-bin/locus.pl?locus=SCH9)*,* [*SEN34*](http://db.yeastgenome.org/cgi-bin/locus.pl?locus=SEN34)*,* [*SFG1*](http://db.yeastgenome.org/cgi-bin/locus.pl?locus=SFG1)*,* [*SHQ1*](http://db.yeastgenome.org/cgi-bin/locus.pl?locus=SHQ1)*,* [*SIK1*](http://db.yeastgenome.org/cgi-bin/locus.pl?locus=SIK1)*,* [*SIR2*](http://db.yeastgenome.org/cgi-bin/locus.pl?locus=SIR2)*,* [*SLI15*](http://db.yeastgenome.org/cgi-bin/locus.pl?locus=SLI15)*,* [*SLN1*](http://db.yeastgenome.org/cgi-bin/locus.pl?locus=SLN1)*,* [*SLX9*](http://db.yeastgenome.org/cgi-bin/locus.pl?locus=SLX9)*,* [*SMI1*](http://db.yeastgenome.org/cgi-bin/locus.pl?locus=SMI1)*,* [*SNU13*](http://db.yeastgenome.org/cgi-bin/locus.pl?locus=SNU13)*,* [*SOF1*](http://db.yeastgenome.org/cgi-bin/locus.pl?locus=SOF1)*,* [*SPB1*](http://db.yeastgenome.org/cgi-bin/locus.pl?locus=SPB1)*,* [*SPB4*](http://db.yeastgenome.org/cgi-bin/locus.pl?locus=SPB4)*,* [*STE12*](http://db.yeastgenome.org/cgi-bin/locus.pl?locus=STE12)*,* [*SUT1*](http://db.yeastgenome.org/cgi-bin/locus.pl?locus=SUT1)*,* [*SUT2*](http://db.yeastgenome.org/cgi-bin/locus.pl?locus=SUT2)*,* [*SUV3*](http://db.yeastgenome.org/cgi-bin/locus.pl?locus=SUV3)*,* [*SWI5*](http://db.yeastgenome.org/cgi-bin/locus.pl?locus=SWI5)*,* [*TAD3*](http://db.yeastgenome.org/cgi-bin/locus.pl?locus=TAD3)*,* [*THP2*](http://db.yeastgenome.org/cgi-bin/locus.pl?locus=THP2)*,* [*TOP1*](http://db.yeastgenome.org/cgi-bin/locus.pl?locus=TOP1)*,* [*TOS4*](http://db.yeastgenome.org/cgi-bin/locus.pl?locus=TOS4)*,* [*TPA1*](http://db.yeastgenome.org/cgi-bin/locus.pl?locus=TPA1)*,* [*TRF5*](http://db.yeastgenome.org/cgi-bin/locus.pl?locus=TRF5)*,* [*TRM1*](http://db.yeastgenome.org/cgi-bin/locus.pl?locus=TRM1)*,* [*TRM10*](http://db.yeastgenome.org/cgi-bin/locus.pl?locus=TRM10)*,* [*TRM11*](http://db.yeastgenome.org/cgi-bin/locus.pl?locus=TRM11)*,* [*TRM112*](http://db.yeastgenome.org/cgi-bin/locus.pl?locus=TRM112)*,* [*TRM13*](http://db.yeastgenome.org/cgi-bin/locus.pl?locus=TRM13)*,* [*TRM2*](http://db.yeastgenome.org/cgi-bin/locus.pl?locus=TRM2)*,* [*TRM3*](http://db.yeastgenome.org/cgi-bin/locus.pl?locus=TRM3)*,* [*TRM7*](http://db.yeastgenome.org/cgi-bin/locus.pl?locus=TRM7)*,* [*TRM8*](http://db.yeastgenome.org/cgi-bin/locus.pl?locus=TRM8)*,* [*TRM82*](http://db.yeastgenome.org/cgi-bin/locus.pl?locus=TRM82)*,* [*TRM9*](http://db.yeastgenome.org/cgi-bin/locus.pl?locus=TRM9)*,* [*TSR1*](http://db.yeastgenome.org/cgi-bin/locus.pl?locus=TSR1)*,* [*TSR2*](http://db.yeastgenome.org/cgi-bin/locus.pl?locus=TSR2)*,* [*TYW1*](http://db.yeastgenome.org/cgi-bin/locus.pl?locus=TYW1)*,* [*UBC9*](http://db.yeastgenome.org/cgi-bin/locus.pl?locus=UBC9)*,* [*UBP10*](http://db.yeastgenome.org/cgi-bin/locus.pl?locus=UBP10)*,* [*UNG1*](http://db.yeastgenome.org/cgi-bin/locus.pl?locus=UNG1)*,* [*URB1*](http://db.yeastgenome.org/cgi-bin/locus.pl?locus=URB1)*,* [*URB2*](http://db.yeastgenome.org/cgi-bin/locus.pl?locus=URB2)*,* [*UTP10*](http://db.yeastgenome.org/cgi-bin/locus.pl?locus=UTP10)*,* [*UTP11*](http://db.yeastgenome.org/cgi-bin/locus.pl?locus=UTP11)*,* [*UTP13*](http://db.yeastgenome.org/cgi-bin/locus.pl?locus=UTP13)*,* [*UTP14*](http://db.yeastgenome.org/cgi-bin/locus.pl?locus=UTP14)*,* [*UTP15*](http://db.yeastgenome.org/cgi-bin/locus.pl?locus=UTP15)*,* [*UTP18*](http://db.yeastgenome.org/cgi-bin/locus.pl?locus=UTP18)*,* [*UTP21*](http://db.yeastgenome.org/cgi-bin/locus.pl?locus=UTP21)*,* [*UTP22*](http://db.yeastgenome.org/cgi-bin/locus.pl?locus=UTP22)*,* [*UTP23*](http://db.yeastgenome.org/cgi-bin/locus.pl?locus=UTP23)*,* [*UTP30*](http://db.yeastgenome.org/cgi-bin/locus.pl?locus=UTP30)*,* [*UTP4*](http://db.yeastgenome.org/cgi-bin/locus.pl?locus=UTP4)*,* [*UTP5*](http://db.yeastgenome.org/cgi-bin/locus.pl?locus=UTP5)*,* [*UTP7*](http://db.yeastgenome.org/cgi-bin/locus.pl?locus=UTP7)*,* [*UTP8*](http://db.yeastgenome.org/cgi-bin/locus.pl?locus=UTP8)*,* [*UTP9*](http://db.yeastgenome.org/cgi-bin/locus.pl?locus=UTP9)*,* [*VRG4*](http://db.yeastgenome.org/cgi-bin/locus.pl?locus=VRG4)*,* [*WRS1*](http://db.yeastgenome.org/cgi-bin/locus.pl?locus=WRS1)*,* [*YEF3*](http://db.yeastgenome.org/cgi-bin/locus.pl?locus=YEF3)*,* [*YHR020W*](http://db.yeastgenome.org/cgi-bin/locus.pl?locus=YHR020W)*,* [*YNL247W*](http://db.yeastgenome.org/cgi-bin/locus.pl?locus=YNL247W)*,* [*YOX1*](http://db.yeastgenome.org/cgi-bin/locus.pl?locus=YOX1)*,* [*YRF1-6*](http://db.yeastgenome.org/cgi-bin/locus.pl?locus=YRF1-6)*,* [*YVH1*](http://db.yeastgenome.org/cgi-bin/locus.pl?locus=YVH1) |
| Primary metabolic process  Primary metabolic process (cont.) | 347 out of 558 genes, 62.2% | 3.59e-15 | [*AAH1*](http://db.yeastgenome.org/cgi-bin/locus.pl?locus=AAH1)*,* [*ADE17*](http://db.yeastgenome.org/cgi-bin/locus.pl?locus=ADE17)*,* [*ADE5,7*](http://db.yeastgenome.org/cgi-bin/locus.pl?locus=ADE5,7)*,* [*ADE6*](http://db.yeastgenome.org/cgi-bin/locus.pl?locus=ADE6)*,* [*ADE8*](http://db.yeastgenome.org/cgi-bin/locus.pl?locus=ADE8)*,* [*AIR1*](http://db.yeastgenome.org/cgi-bin/locus.pl?locus=AIR1)*,* [*ALK1*](http://db.yeastgenome.org/cgi-bin/locus.pl?locus=ALK1)*,* [*APT1*](http://db.yeastgenome.org/cgi-bin/locus.pl?locus=APT1)*,* [*ASC1*](http://db.yeastgenome.org/cgi-bin/locus.pl?locus=ASC1)*,* [*ASH1*](http://db.yeastgenome.org/cgi-bin/locus.pl?locus=ASH1)*,* [*ASP1*](http://db.yeastgenome.org/cgi-bin/locus.pl?locus=ASP1)*,* [*ATF2*](http://db.yeastgenome.org/cgi-bin/locus.pl?locus=ATF2)*,* [*AUR1*](http://db.yeastgenome.org/cgi-bin/locus.pl?locus=AUR1)*,* [*BCD1*](http://db.yeastgenome.org/cgi-bin/locus.pl?locus=BCD1)*,* [*BMS1*](http://db.yeastgenome.org/cgi-bin/locus.pl?locus=BMS1)*,* [*BST1*](http://db.yeastgenome.org/cgi-bin/locus.pl?locus=BST1)*,* [*BUD21*](http://db.yeastgenome.org/cgi-bin/locus.pl?locus=BUD21)*,* [*BUD32*](http://db.yeastgenome.org/cgi-bin/locus.pl?locus=BUD32)*,* [*CBF5*](http://db.yeastgenome.org/cgi-bin/locus.pl?locus=CBF5)*,* [*CDC14*](http://db.yeastgenome.org/cgi-bin/locus.pl?locus=CDC14)*,* [*CDC20*](http://db.yeastgenome.org/cgi-bin/locus.pl?locus=CDC20)*,* [*CDC47*](http://db.yeastgenome.org/cgi-bin/locus.pl?locus=CDC47)*,* [*CGR1*](http://db.yeastgenome.org/cgi-bin/locus.pl?locus=CGR1)*,* [*CIC1*](http://db.yeastgenome.org/cgi-bin/locus.pl?locus=CIC1)*,* [*CLA4*](http://db.yeastgenome.org/cgi-bin/locus.pl?locus=CLA4)*, CMS1,* [*CSM3*](http://db.yeastgenome.org/cgi-bin/locus.pl?locus=CSM3)*,* [*CUS1*](http://db.yeastgenome.org/cgi-bin/locus.pl?locus=CUS1)*,* [*CWC2*](http://db.yeastgenome.org/cgi-bin/locus.pl?locus=CWC2)*,* [*CYB5*](http://db.yeastgenome.org/cgi-bin/locus.pl?locus=CYB5)*,* [*DBP10*](http://db.yeastgenome.org/cgi-bin/locus.pl?locus=DBP10)*,* [*DBP2*](http://db.yeastgenome.org/cgi-bin/locus.pl?locus=DBP2)*,* [*DBP3*](http://db.yeastgenome.org/cgi-bin/locus.pl?locus=DBP3)*,* [*DBP6*](http://db.yeastgenome.org/cgi-bin/locus.pl?locus=DBP6)*,* [*DBP7*](http://db.yeastgenome.org/cgi-bin/locus.pl?locus=DBP7)*,* [*DBP8*](http://db.yeastgenome.org/cgi-bin/locus.pl?locus=DBP8)*,* [*DBP9*](http://db.yeastgenome.org/cgi-bin/locus.pl?locus=DBP9)*,* [*DIM1*](http://db.yeastgenome.org/cgi-bin/locus.pl?locus=DIM1)*,* [*DIP2*](http://db.yeastgenome.org/cgi-bin/locus.pl?locus=DIP2)*,* [*DPB2*](http://db.yeastgenome.org/cgi-bin/locus.pl?locus=DPB2)*,* [*DPH2*](http://db.yeastgenome.org/cgi-bin/locus.pl?locus=DPH2)*,* [*DRS1*](http://db.yeastgenome.org/cgi-bin/locus.pl?locus=DRS1)*,* [*DUS1*](http://db.yeastgenome.org/cgi-bin/locus.pl?locus=DUS1)*,* [*DUS3*](http://db.yeastgenome.org/cgi-bin/locus.pl?locus=DUS3)*,* [*DUS4*](http://db.yeastgenome.org/cgi-bin/locus.pl?locus=DUS4)*,* [*DUT1*](http://db.yeastgenome.org/cgi-bin/locus.pl?locus=DUT1)*,* [*DYS1*](http://db.yeastgenome.org/cgi-bin/locus.pl?locus=DYS1)*,* [*EBP2*](http://db.yeastgenome.org/cgi-bin/locus.pl?locus=EBP2)*,* [*ECM16*](http://db.yeastgenome.org/cgi-bin/locus.pl?locus=ECM16)*,* [*EFB1*](http://db.yeastgenome.org/cgi-bin/locus.pl?locus=EFB1)*. EFG1,* [*EKI1*](http://db.yeastgenome.org/cgi-bin/locus.pl?locus=EKI1)*,* [*ELP2*](http://db.yeastgenome.org/cgi-bin/locus.pl?locus=ELP2)*,* [*ELP3*](http://db.yeastgenome.org/cgi-bin/locus.pl?locus=ELP3)*,* [*EMG1*](http://db.yeastgenome.org/cgi-bin/locus.pl?locus=EMG1)*,* [*ENP1*](http://db.yeastgenome.org/cgi-bin/locus.pl?locus=ENP1)*,* [*ENP2*](http://db.yeastgenome.org/cgi-bin/locus.pl?locus=ENP2)*,* [*ERB1*](http://db.yeastgenome.org/cgi-bin/locus.pl?locus=ERB1)*,* [*ERG1*](http://db.yeastgenome.org/cgi-bin/locus.pl?locus=ERG1)*,* [*ERG11*](http://db.yeastgenome.org/cgi-bin/locus.pl?locus=ERG11)*,* [*ERG3*](http://db.yeastgenome.org/cgi-bin/locus.pl?locus=ERG3)*,* [*ERG5*](http://db.yeastgenome.org/cgi-bin/locus.pl?locus=ERG5)*,* [*ESF1*](http://db.yeastgenome.org/cgi-bin/locus.pl?locus=ESF1)*,* [*ESF2*](http://db.yeastgenome.org/cgi-bin/locus.pl?locus=ESF2)*,* [*EXG1*](http://db.yeastgenome.org/cgi-bin/locus.pl?locus=EXG1)*,* [*FAA4*](http://db.yeastgenome.org/cgi-bin/locus.pl?locus=FAA4)*,* [*FAF1*](http://db.yeastgenome.org/cgi-bin/locus.pl?locus=FAF1)*,* [*FAL1*](http://db.yeastgenome.org/cgi-bin/locus.pl?locus=FAL1)*,* [*FAP7*](http://db.yeastgenome.org/cgi-bin/locus.pl?locus=FAP7)*,* [*FCF2*](http://db.yeastgenome.org/cgi-bin/locus.pl?locus=FCF2)*,* [*FEN1*](http://db.yeastgenome.org/cgi-bin/locus.pl?locus=FEN1)*,* [*FKH1*](http://db.yeastgenome.org/cgi-bin/locus.pl?locus=FKH1)*,* [*FPR4*](http://db.yeastgenome.org/cgi-bin/locus.pl?locus=FPR4)*,* [*FUR1*](http://db.yeastgenome.org/cgi-bin/locus.pl?locus=FUR1)*,* [*FYV7*](http://db.yeastgenome.org/cgi-bin/locus.pl?locus=FYV7)*,* [*GAR1*](http://db.yeastgenome.org/cgi-bin/locus.pl?locus=GAR1)*,* [*GCD10*](http://db.yeastgenome.org/cgi-bin/locus.pl?locus=GCD10)*,* [*GCD11*](http://db.yeastgenome.org/cgi-bin/locus.pl?locus=GCD11)*,* [*GCD14*](http://db.yeastgenome.org/cgi-bin/locus.pl?locus=GCD14)*,* [*GCD2*](http://db.yeastgenome.org/cgi-bin/locus.pl?locus=GCD2)*,* [*GCN3*](http://db.yeastgenome.org/cgi-bin/locus.pl?locus=GCN3)*,* [*GCR1*](http://db.yeastgenome.org/cgi-bin/locus.pl?locus=GCR1)*,* [*GCR2*](http://db.yeastgenome.org/cgi-bin/locus.pl?locus=GCR2)*,* [*GLN1*](http://db.yeastgenome.org/cgi-bin/locus.pl?locus=GLN1)*,* [*GLN4*](http://db.yeastgenome.org/cgi-bin/locus.pl?locus=GLN4)*,* [*GPI13*](http://db.yeastgenome.org/cgi-bin/locus.pl?locus=GPI13)*,* [*GRC3*](http://db.yeastgenome.org/cgi-bin/locus.pl?locus=GRC3)*,* [*GUA1*](http://db.yeastgenome.org/cgi-bin/locus.pl?locus=GUA1)*,* [*HAS1*](http://db.yeastgenome.org/cgi-bin/locus.pl?locus=HAS1)*,* [*HCA4*](http://db.yeastgenome.org/cgi-bin/locus.pl?locus=HCA4)*,* [*HEM1*](http://db.yeastgenome.org/cgi-bin/locus.pl?locus=HEM1)*,* [*HHF2*](http://db.yeastgenome.org/cgi-bin/locus.pl?locus=HHF2)*,* [*HHT1*](http://db.yeastgenome.org/cgi-bin/locus.pl?locus=HHT1)*,* [*HIR2*](http://db.yeastgenome.org/cgi-bin/locus.pl?locus=HIR2)*,* [*HMG1*](http://db.yeastgenome.org/cgi-bin/locus.pl?locus=HMG1)*,* [*HMT1*](http://db.yeastgenome.org/cgi-bin/locus.pl?locus=HMT1)*,* [*HPT1*](http://db.yeastgenome.org/cgi-bin/locus.pl?locus=HPT1)*,* [*HST3*](http://db.yeastgenome.org/cgi-bin/locus.pl?locus=HST3)*,* [*HTA1*](http://db.yeastgenome.org/cgi-bin/locus.pl?locus=HTA1)*,* [*HTA2*](http://db.yeastgenome.org/cgi-bin/locus.pl?locus=HTA2)*,* [*HTB1*](http://db.yeastgenome.org/cgi-bin/locus.pl?locus=HTB1)*,* [*HTB2*](http://db.yeastgenome.org/cgi-bin/locus.pl?locus=HTB2)*,* [*HTZ1*](http://db.yeastgenome.org/cgi-bin/locus.pl?locus=HTZ1)*,* [*IFH1*](http://db.yeastgenome.org/cgi-bin/locus.pl?locus=IFH1)*,* [*ILV3*](http://db.yeastgenome.org/cgi-bin/locus.pl?locus=ILV3)*,* [*ILV5*](http://db.yeastgenome.org/cgi-bin/locus.pl?locus=ILV5)*,* [*IMD3*](http://db.yeastgenome.org/cgi-bin/locus.pl?locus=IMD3)*,* [*IMP3*](http://db.yeastgenome.org/cgi-bin/locus.pl?locus=IMP3)*,* [*INO2*](http://db.yeastgenome.org/cgi-bin/locus.pl?locus=INO2)*,* [*IPI1*](http://db.yeastgenome.org/cgi-bin/locus.pl?locus=IPI1)*,* [*IPI3*](http://db.yeastgenome.org/cgi-bin/locus.pl?locus=IPI3)*,* [*IZH1*](http://db.yeastgenome.org/cgi-bin/locus.pl?locus=IZH1)*,* [*JJJ3*](http://db.yeastgenome.org/cgi-bin/locus.pl?locus=JJJ3)*,* [*KAE1*](http://db.yeastgenome.org/cgi-bin/locus.pl?locus=KAE1)*,* [*KRR1*](http://db.yeastgenome.org/cgi-bin/locus.pl?locus=KRR1)*,* [*KTI12*](http://db.yeastgenome.org/cgi-bin/locus.pl?locus=KTI12)*,* [*KTR5*](http://db.yeastgenome.org/cgi-bin/locus.pl?locus=KTR5)*,* [*LCP5*](http://db.yeastgenome.org/cgi-bin/locus.pl?locus=LCP5)*,* [*LEU9*](http://db.yeastgenome.org/cgi-bin/locus.pl?locus=LEU9)*,* [*LIA1*](http://db.yeastgenome.org/cgi-bin/locus.pl?locus=LIA1)*,* [*LYS12*](http://db.yeastgenome.org/cgi-bin/locus.pl?locus=LYS12)*,* [*LYS4*](http://db.yeastgenome.org/cgi-bin/locus.pl?locus=LYS4)*,* [*MAE1*](http://db.yeastgenome.org/cgi-bin/locus.pl?locus=MAE1)*,* [*MAK11*](http://db.yeastgenome.org/cgi-bin/locus.pl?locus=MAK11)*,* [*MAK16*](http://db.yeastgenome.org/cgi-bin/locus.pl?locus=MAK16)*,* [*MAK3*](http://db.yeastgenome.org/cgi-bin/locus.pl?locus=MAK3)*,* [*MAP1*](http://db.yeastgenome.org/cgi-bin/locus.pl?locus=MAP1)*,* [*MES1*](http://db.yeastgenome.org/cgi-bin/locus.pl?locus=MES1)*,* [*MEU1*](http://db.yeastgenome.org/cgi-bin/locus.pl?locus=MEU1)*,* [*MIS1*](http://db.yeastgenome.org/cgi-bin/locus.pl?locus=MIS1)*,* [*MNN2*](http://db.yeastgenome.org/cgi-bin/locus.pl?locus=MNN2)*,* [*MOT1*](http://db.yeastgenome.org/cgi-bin/locus.pl?locus=MOT1)*,* [*MPP10*](http://db.yeastgenome.org/cgi-bin/locus.pl?locus=MPP10)*,* [*MRD1*](http://db.yeastgenome.org/cgi-bin/locus.pl?locus=MRD1)*,* [*MRT4*](http://db.yeastgenome.org/cgi-bin/locus.pl?locus=MRT4)*,* [*MSH1*](http://db.yeastgenome.org/cgi-bin/locus.pl?locus=MSH1)*,* [*MSH6*](http://db.yeastgenome.org/cgi-bin/locus.pl?locus=MSH6)*,* [*MTD1*](http://db.yeastgenome.org/cgi-bin/locus.pl?locus=MTD1)*,* [*MTR3*](http://db.yeastgenome.org/cgi-bin/locus.pl?locus=MTR3)*,* [*NAF1*](http://db.yeastgenome.org/cgi-bin/locus.pl?locus=NAF1)*,* [*NAN1*](http://db.yeastgenome.org/cgi-bin/locus.pl?locus=NAN1)*,* [*NCL1*](http://db.yeastgenome.org/cgi-bin/locus.pl?locus=NCL1)*,* [*NCS2*](http://db.yeastgenome.org/cgi-bin/locus.pl?locus=NCS2)*,* [*NHP2*](http://db.yeastgenome.org/cgi-bin/locus.pl?locus=NHP2)*,* [*NIP7*](http://db.yeastgenome.org/cgi-bin/locus.pl?locus=NIP7)*,* [*NOB1*](http://db.yeastgenome.org/cgi-bin/locus.pl?locus=NOB1)*,* [*NOC3*](http://db.yeastgenome.org/cgi-bin/locus.pl?locus=NOC3)*,* [*NOC4*](http://db.yeastgenome.org/cgi-bin/locus.pl?locus=NOC4)*,* [*NOG1*](http://db.yeastgenome.org/cgi-bin/locus.pl?locus=NOG1)*,* [*NOP12*](http://db.yeastgenome.org/cgi-bin/locus.pl?locus=NOP12)*,* [*NOP14*](http://db.yeastgenome.org/cgi-bin/locus.pl?locus=NOP14)*,* [*NOP2*](http://db.yeastgenome.org/cgi-bin/locus.pl?locus=NOP2)*,* [*NOP4*](http://db.yeastgenome.org/cgi-bin/locus.pl?locus=NOP4)*,* [*NOP53*](http://db.yeastgenome.org/cgi-bin/locus.pl?locus=NOP53)*,* [*NOP58*](http://db.yeastgenome.org/cgi-bin/locus.pl?locus=NOP58)*,* [*NOP6*](http://db.yeastgenome.org/cgi-bin/locus.pl?locus=NOP6)*,* [*NOP7*](http://db.yeastgenome.org/cgi-bin/locus.pl?locus=NOP7)*,* [*NOP8*](http://db.yeastgenome.org/cgi-bin/locus.pl?locus=NOP8)*,* [*NOP9*](http://db.yeastgenome.org/cgi-bin/locus.pl?locus=NOP9)*,* [*NSE4*](http://db.yeastgenome.org/cgi-bin/locus.pl?locus=NSE4)*,* [*NSR1*](http://db.yeastgenome.org/cgi-bin/locus.pl?locus=NSR1)*,* [*NUC1*](http://db.yeastgenome.org/cgi-bin/locus.pl?locus=NUC1)*,* [*NUG1*](http://db.yeastgenome.org/cgi-bin/locus.pl?locus=NUG1)*,* [*ORC1*](http://db.yeastgenome.org/cgi-bin/locus.pl?locus=ORC1)*,* [*PLB2*](http://db.yeastgenome.org/cgi-bin/locus.pl?locus=PLB2)*,* [*PMT2*](http://db.yeastgenome.org/cgi-bin/locus.pl?locus=PMT2)*,* [*PNO1*](http://db.yeastgenome.org/cgi-bin/locus.pl?locus=PNO1)*,* [*POL5*](http://db.yeastgenome.org/cgi-bin/locus.pl?locus=POL5)*,* [*POP3*](http://db.yeastgenome.org/cgi-bin/locus.pl?locus=POP3)*,* [*POP6*](http://db.yeastgenome.org/cgi-bin/locus.pl?locus=POP6)*,* [*POP8*](http://db.yeastgenome.org/cgi-bin/locus.pl?locus=POP8)*,* [*PPT1*](http://db.yeastgenome.org/cgi-bin/locus.pl?locus=PPT1)*,* [*PRO1*](http://db.yeastgenome.org/cgi-bin/locus.pl?locus=PRO1)*,* [*PRP19*](http://db.yeastgenome.org/cgi-bin/locus.pl?locus=PRP19)*,* [*PRP24*](http://db.yeastgenome.org/cgi-bin/locus.pl?locus=PRP24)*,* [*PRP43*](http://db.yeastgenome.org/cgi-bin/locus.pl?locus=PRP43)*,* [*PRS1*](http://db.yeastgenome.org/cgi-bin/locus.pl?locus=PRS1)*,* [*PRS2*](http://db.yeastgenome.org/cgi-bin/locus.pl?locus=PRS2)*,* [*PRS4*](http://db.yeastgenome.org/cgi-bin/locus.pl?locus=PRS4)*,* [*PSY4*](http://db.yeastgenome.org/cgi-bin/locus.pl?locus=PSY4)*,* [*PUF6*](http://db.yeastgenome.org/cgi-bin/locus.pl?locus=PUF6)*,* [*PUS1*](http://db.yeastgenome.org/cgi-bin/locus.pl?locus=PUS1)*,* [*PUS4*](http://db.yeastgenome.org/cgi-bin/locus.pl?locus=PUS4)*,* [*PUS7*](http://db.yeastgenome.org/cgi-bin/locus.pl?locus=PUS7)*,* [*PWP1*](http://db.yeastgenome.org/cgi-bin/locus.pl?locus=PWP1)*,* [*PWP2*](http://db.yeastgenome.org/cgi-bin/locus.pl?locus=PWP2)*,* [*PXR1*](http://db.yeastgenome.org/cgi-bin/locus.pl?locus=PXR1)*,* [*RAP1*](http://db.yeastgenome.org/cgi-bin/locus.pl?locus=RAP1)*,* [*RBA50*](http://db.yeastgenome.org/cgi-bin/locus.pl?locus=RBA50)*,* [*RCL1*](http://db.yeastgenome.org/cgi-bin/locus.pl?locus=RCL1)*,* [*RET1*](http://db.yeastgenome.org/cgi-bin/locus.pl?locus=RET1)*,* [*REX4*](http://db.yeastgenome.org/cgi-bin/locus.pl?locus=REX4)*,* [*RIO1*](http://db.yeastgenome.org/cgi-bin/locus.pl?locus=RIO1)*,* [*RIX1*](http://db.yeastgenome.org/cgi-bin/locus.pl?locus=RIX1)*,* [*RKI1*](http://db.yeastgenome.org/cgi-bin/locus.pl?locus=RKI1)*,* [*RLI1*](http://db.yeastgenome.org/cgi-bin/locus.pl?locus=RLI1)*,* [*RLP7*](http://db.yeastgenome.org/cgi-bin/locus.pl?locus=RLP7)*,* [*RMT2*](http://db.yeastgenome.org/cgi-bin/locus.pl?locus=RMT2)*,* [*RNH201*](http://db.yeastgenome.org/cgi-bin/locus.pl?locus=RNH201)*,* [*RNT1*](http://db.yeastgenome.org/cgi-bin/locus.pl?locus=RNT1)*,* [*ROK1*](http://db.yeastgenome.org/cgi-bin/locus.pl?locus=ROK1)*,* [*ROX3*](http://db.yeastgenome.org/cgi-bin/locus.pl?locus=ROX3)*,* [*RPA12*](http://db.yeastgenome.org/cgi-bin/locus.pl?locus=RPA12)*,* [*RPA190*](http://db.yeastgenome.org/cgi-bin/locus.pl?locus=RPA190)*,* [*RPA34*](http://db.yeastgenome.org/cgi-bin/locus.pl?locus=RPA34)*,* [*RPA43*](http://db.yeastgenome.org/cgi-bin/locus.pl?locus=RPA43)*,* [*RPA49*](http://db.yeastgenome.org/cgi-bin/locus.pl?locus=RPA49)*,* [*RPB10*](http://db.yeastgenome.org/cgi-bin/locus.pl?locus=RPB10)*,* [*RPB5*](http://db.yeastgenome.org/cgi-bin/locus.pl?locus=RPB5)*,* [*RPB8*](http://db.yeastgenome.org/cgi-bin/locus.pl?locus=RPB8)*,* [*RPB9*](http://db.yeastgenome.org/cgi-bin/locus.pl?locus=RPB9)*,* [*RPC11*](http://db.yeastgenome.org/cgi-bin/locus.pl?locus=RPC11)*,* [*RPC19*](http://db.yeastgenome.org/cgi-bin/locus.pl?locus=RPC19)*,* [*RPC31*](http://db.yeastgenome.org/cgi-bin/locus.pl?locus=RPC31)*,* [*RPC34*](http://db.yeastgenome.org/cgi-bin/locus.pl?locus=RPC34)*,* [*RPC37*](http://db.yeastgenome.org/cgi-bin/locus.pl?locus=RPC37)*,* [*RPC40*](http://db.yeastgenome.org/cgi-bin/locus.pl?locus=RPC40)*,* [*RPC53*](http://db.yeastgenome.org/cgi-bin/locus.pl?locus=RPC53)*,* [*RPC82*](http://db.yeastgenome.org/cgi-bin/locus.pl?locus=RPC82)*,* [*RPF1*](http://db.yeastgenome.org/cgi-bin/locus.pl?locus=RPF1)*,* [*RPF2*](http://db.yeastgenome.org/cgi-bin/locus.pl?locus=RPF2)*,* [*RPL13A*](http://db.yeastgenome.org/cgi-bin/locus.pl?locus=RPL13A)*,* [*RPL14A*](http://db.yeastgenome.org/cgi-bin/locus.pl?locus=RPL14A)*,* [*RPL14B*](http://db.yeastgenome.org/cgi-bin/locus.pl?locus=RPL14B)*,* [*RPL18B*](http://db.yeastgenome.org/cgi-bin/locus.pl?locus=RPL18B)*,* [*RPL20A*](http://db.yeastgenome.org/cgi-bin/locus.pl?locus=RPL20A)*,* [*RPL21B*](http://db.yeastgenome.org/cgi-bin/locus.pl?locus=RPL21B)*,* [*RPL26A*](http://db.yeastgenome.org/cgi-bin/locus.pl?locus=RPL26A)*,* [*RPL31B*](http://db.yeastgenome.org/cgi-bin/locus.pl?locus=RPL31B)*,* [*RPL33B*](http://db.yeastgenome.org/cgi-bin/locus.pl?locus=RPL33B)*,* [*RPL36A*](http://db.yeastgenome.org/cgi-bin/locus.pl?locus=RPL36A)*,* [*RPL43B*](http://db.yeastgenome.org/cgi-bin/locus.pl?locus=RPL43B)*,* [*RPL7B*](http://db.yeastgenome.org/cgi-bin/locus.pl?locus=RPL7B)*,* [*RPO26*](http://db.yeastgenome.org/cgi-bin/locus.pl?locus=RPO26)*,* [*RPP1*](http://db.yeastgenome.org/cgi-bin/locus.pl?locus=RPP1)*,* [*RPS10B*](http://db.yeastgenome.org/cgi-bin/locus.pl?locus=RPS10B)*,* [*RPS14B*](http://db.yeastgenome.org/cgi-bin/locus.pl?locus=RPS14B)*,* [*RPS19B*](http://db.yeastgenome.org/cgi-bin/locus.pl?locus=RPS19B)*,* [*RPS1A*](http://db.yeastgenome.org/cgi-bin/locus.pl?locus=RPS1A)*,* [*RPS21A*](http://db.yeastgenome.org/cgi-bin/locus.pl?locus=RPS21A)*,* [*RPS21B*](http://db.yeastgenome.org/cgi-bin/locus.pl?locus=RPS21B)*,* [*RPS26B*](http://db.yeastgenome.org/cgi-bin/locus.pl?locus=RPS26B)*,* [*RPS27A*](http://db.yeastgenome.org/cgi-bin/locus.pl?locus=RPS27A)*,* [*RPS29A*](http://db.yeastgenome.org/cgi-bin/locus.pl?locus=RPS29A)*,* [*RPS7A*](http://db.yeastgenome.org/cgi-bin/locus.pl?locus=RPS7A)*,* [*RPS7B*](http://db.yeastgenome.org/cgi-bin/locus.pl?locus=RPS7B)*,* [*RRM3*](http://db.yeastgenome.org/cgi-bin/locus.pl?locus=RRM3)*,* [*RRN11*](http://db.yeastgenome.org/cgi-bin/locus.pl?locus=RRN11)*,* [*RRN7*](http://db.yeastgenome.org/cgi-bin/locus.pl?locus=RRN7)*,* [*RRP1*](http://db.yeastgenome.org/cgi-bin/locus.pl?locus=RRP1)*,* [*RRP12*](http://db.yeastgenome.org/cgi-bin/locus.pl?locus=RRP12)*,* [*RRP15*](http://db.yeastgenome.org/cgi-bin/locus.pl?locus=RRP15)*,* [*RRP17*](http://db.yeastgenome.org/cgi-bin/locus.pl?locus=RRP17)*,* [*RRP3*](http://db.yeastgenome.org/cgi-bin/locus.pl?locus=RRP3)*, RRP36,* [*RRP40*](http://db.yeastgenome.org/cgi-bin/locus.pl?locus=RRP40)*,* [*RRP43*](http://db.yeastgenome.org/cgi-bin/locus.pl?locus=RRP43)*,* [*RRP5*](http://db.yeastgenome.org/cgi-bin/locus.pl?locus=RRP5)*,* [*RRP6*](http://db.yeastgenome.org/cgi-bin/locus.pl?locus=RRP6)*,* [*RRP8*](http://db.yeastgenome.org/cgi-bin/locus.pl?locus=RRP8)*,* [*RRP9*](http://db.yeastgenome.org/cgi-bin/locus.pl?locus=RRP9)*,* [*RRS1*](http://db.yeastgenome.org/cgi-bin/locus.pl?locus=RRS1)*,* [*RVB1*](http://db.yeastgenome.org/cgi-bin/locus.pl?locus=RVB1)*,* [*SAN1*](http://db.yeastgenome.org/cgi-bin/locus.pl?locus=SAN1)*,* [*SAS10*](http://db.yeastgenome.org/cgi-bin/locus.pl?locus=SAS10)*,* [*SCC2*](http://db.yeastgenome.org/cgi-bin/locus.pl?locus=SCC2)*,* [*SCH9*](http://db.yeastgenome.org/cgi-bin/locus.pl?locus=SCH9)*,* [*SCS7*](http://db.yeastgenome.org/cgi-bin/locus.pl?locus=SCS7)*,* [*SEN34*](http://db.yeastgenome.org/cgi-bin/locus.pl?locus=SEN34)*,* [*SFG1*](http://db.yeastgenome.org/cgi-bin/locus.pl?locus=SFG1)*,* [*SHQ1*](http://db.yeastgenome.org/cgi-bin/locus.pl?locus=SHQ1)*,* [*SIK1*](http://db.yeastgenome.org/cgi-bin/locus.pl?locus=SIK1)*,* [*SIR2*](http://db.yeastgenome.org/cgi-bin/locus.pl?locus=SIR2)*,* [*SLI15*](http://db.yeastgenome.org/cgi-bin/locus.pl?locus=SLI15)*,* [*SLN1*](http://db.yeastgenome.org/cgi-bin/locus.pl?locus=SLN1)*,* [*SLX9*](http://db.yeastgenome.org/cgi-bin/locus.pl?locus=SLX9)*,* [*SMI1*](http://db.yeastgenome.org/cgi-bin/locus.pl?locus=SMI1)*,* [*SNU13*](http://db.yeastgenome.org/cgi-bin/locus.pl?locus=SNU13)*,* [*SOF1*](http://db.yeastgenome.org/cgi-bin/locus.pl?locus=SOF1)*,* [*SPB1*](http://db.yeastgenome.org/cgi-bin/locus.pl?locus=SPB1)*,* [*SPB4*](http://db.yeastgenome.org/cgi-bin/locus.pl?locus=SPB4)*,* [*SPE4*](http://db.yeastgenome.org/cgi-bin/locus.pl?locus=SPE4)*,* [*SRO9*](http://db.yeastgenome.org/cgi-bin/locus.pl?locus=SRO9)*,* [*STE12*](http://db.yeastgenome.org/cgi-bin/locus.pl?locus=STE12)*,* [*SUR2*](http://db.yeastgenome.org/cgi-bin/locus.pl?locus=SUR2)*,* [*SUR4*](http://db.yeastgenome.org/cgi-bin/locus.pl?locus=SUR4)*,* [*SUT1*](http://db.yeastgenome.org/cgi-bin/locus.pl?locus=SUT1)*,* [*SUT2*](http://db.yeastgenome.org/cgi-bin/locus.pl?locus=SUT2)*,* [*SUV3*](http://db.yeastgenome.org/cgi-bin/locus.pl?locus=SUV3)*,* [*SWI5*](http://db.yeastgenome.org/cgi-bin/locus.pl?locus=SWI5)*,* [*TAD3*](http://db.yeastgenome.org/cgi-bin/locus.pl?locus=TAD3)*,* [*THP2*](http://db.yeastgenome.org/cgi-bin/locus.pl?locus=THP2)*,* [*TIF2*](http://db.yeastgenome.org/cgi-bin/locus.pl?locus=TIF2)*,* [*TIF3*](http://db.yeastgenome.org/cgi-bin/locus.pl?locus=TIF3)*,* [*TIF35*](http://db.yeastgenome.org/cgi-bin/locus.pl?locus=TIF35)*,* [*TIF4631*](http://db.yeastgenome.org/cgi-bin/locus.pl?locus=TIF4631)*,* [*TOP1*](http://db.yeastgenome.org/cgi-bin/locus.pl?locus=TOP1)*,* [*TOS4*](http://db.yeastgenome.org/cgi-bin/locus.pl?locus=TOS4)*,* [*TPA1*](http://db.yeastgenome.org/cgi-bin/locus.pl?locus=TPA1)*,* [*TRF5*](http://db.yeastgenome.org/cgi-bin/locus.pl?locus=TRF5)*,* [*TRM1*](http://db.yeastgenome.org/cgi-bin/locus.pl?locus=TRM1)*,* [*TRM10*](http://db.yeastgenome.org/cgi-bin/locus.pl?locus=TRM10)*,* [*TRM11*](http://db.yeastgenome.org/cgi-bin/locus.pl?locus=TRM11)*,* [*TRM112*](http://db.yeastgenome.org/cgi-bin/locus.pl?locus=TRM112)*,* [*TRM13*](http://db.yeastgenome.org/cgi-bin/locus.pl?locus=TRM13)*,* [*TRM2*](http://db.yeastgenome.org/cgi-bin/locus.pl?locus=TRM2)*,* [*TRM3*](http://db.yeastgenome.org/cgi-bin/locus.pl?locus=TRM3)*,* [*TRM7*](http://db.yeastgenome.org/cgi-bin/locus.pl?locus=TRM7)*,* [*TRM8*](http://db.yeastgenome.org/cgi-bin/locus.pl?locus=TRM8)*,* [*TRM82*](http://db.yeastgenome.org/cgi-bin/locus.pl?locus=TRM82)*,* [*TRM9*](http://db.yeastgenome.org/cgi-bin/locus.pl?locus=TRM9)*,* [*TSC10*](http://db.yeastgenome.org/cgi-bin/locus.pl?locus=TSC10)*,* [*TSR1*](http://db.yeastgenome.org/cgi-bin/locus.pl?locus=TSR1)*,* [*TSR2*](http://db.yeastgenome.org/cgi-bin/locus.pl?locus=TSR2)*,* [*TYW1*](http://db.yeastgenome.org/cgi-bin/locus.pl?locus=TYW1)*,* [*UBC9*](http://db.yeastgenome.org/cgi-bin/locus.pl?locus=UBC9)*,* [*UBP10*](http://db.yeastgenome.org/cgi-bin/locus.pl?locus=UBP10)*,* [*UNG1*](http://db.yeastgenome.org/cgi-bin/locus.pl?locus=UNG1)*,* [*URA4*](http://db.yeastgenome.org/cgi-bin/locus.pl?locus=URA4)*,* [*URA5*](http://db.yeastgenome.org/cgi-bin/locus.pl?locus=URA5)*,* [*URA7*](http://db.yeastgenome.org/cgi-bin/locus.pl?locus=URA7)*,* [*URB1*](http://db.yeastgenome.org/cgi-bin/locus.pl?locus=URB1)*,* [*URB2*](http://db.yeastgenome.org/cgi-bin/locus.pl?locus=URB2)*,* [*URK1*](http://db.yeastgenome.org/cgi-bin/locus.pl?locus=URK1)*,* [*UTP10*](http://db.yeastgenome.org/cgi-bin/locus.pl?locus=UTP10)*,* [*UTP11*](http://db.yeastgenome.org/cgi-bin/locus.pl?locus=UTP11)*,* [*UTP13*](http://db.yeastgenome.org/cgi-bin/locus.pl?locus=UTP13)*,* [*UTP14*](http://db.yeastgenome.org/cgi-bin/locus.pl?locus=UTP14)*,* [*UTP15*](http://db.yeastgenome.org/cgi-bin/locus.pl?locus=UTP15)*,* [*UTP18*](http://db.yeastgenome.org/cgi-bin/locus.pl?locus=UTP18)*,* [*UTP21*](http://db.yeastgenome.org/cgi-bin/locus.pl?locus=UTP21)*,* [*UTP22*](http://db.yeastgenome.org/cgi-bin/locus.pl?locus=UTP22)*,* [*UTP23*](http://db.yeastgenome.org/cgi-bin/locus.pl?locus=UTP23)*,* [*UTP30*](http://db.yeastgenome.org/cgi-bin/locus.pl?locus=UTP30)*,* [*UTP4*](http://db.yeastgenome.org/cgi-bin/locus.pl?locus=UTP4)*,* [*UTP5*](http://db.yeastgenome.org/cgi-bin/locus.pl?locus=UTP5)*,* [*UTP7*](http://db.yeastgenome.org/cgi-bin/locus.pl?locus=UTP7)*,* [*UTP8*](http://db.yeastgenome.org/cgi-bin/locus.pl?locus=UTP8)*,* [*UTP9*](http://db.yeastgenome.org/cgi-bin/locus.pl?locus=UTP9)*,* [*VRG4*](http://db.yeastgenome.org/cgi-bin/locus.pl?locus=VRG4)*,* [*WRS1*](http://db.yeastgenome.org/cgi-bin/locus.pl?locus=WRS1)*,* [*YEF3*](http://db.yeastgenome.org/cgi-bin/locus.pl?locus=YEF3)*,* [*YHR020W*](http://db.yeastgenome.org/cgi-bin/locus.pl?locus=YHR020W)*,* [*YNL247W*](http://db.yeastgenome.org/cgi-bin/locus.pl?locus=YNL247W)*,* [*YOX1*](http://db.yeastgenome.org/cgi-bin/locus.pl?locus=YOX1)*,* [*YRF1-6*](http://db.yeastgenome.org/cgi-bin/locus.pl?locus=YRF1-6)*,* [*YVH1*](http://db.yeastgenome.org/cgi-bin/locus.pl?locus=YVH1) |
| Cellular metabolic process | 358 out of 558 genes, 64.2% | 3.85e-15 | [*AAH1*](http://db.yeastgenome.org/cgi-bin/locus.pl?locus=AAH1)*,* [*ADE17*](http://db.yeastgenome.org/cgi-bin/locus.pl?locus=ADE17)*,* [*ADE5,7*](http://db.yeastgenome.org/cgi-bin/locus.pl?locus=ADE5,7)*,* [*ADE6*](http://db.yeastgenome.org/cgi-bin/locus.pl?locus=ADE6)*,* [*ADE8*](http://db.yeastgenome.org/cgi-bin/locus.pl?locus=ADE8)*,* [*AIR1*](http://db.yeastgenome.org/cgi-bin/locus.pl?locus=AIR1)*,* [*ALK1*](http://db.yeastgenome.org/cgi-bin/locus.pl?locus=ALK1)*,* [*APT1*](http://db.yeastgenome.org/cgi-bin/locus.pl?locus=APT1)*,* [*ASC1*](http://db.yeastgenome.org/cgi-bin/locus.pl?locus=ASC1)*,* [*ASH1*](http://db.yeastgenome.org/cgi-bin/locus.pl?locus=ASH1)*,* [*ASP1*](http://db.yeastgenome.org/cgi-bin/locus.pl?locus=ASP1)*,* [*ATF2*](http://db.yeastgenome.org/cgi-bin/locus.pl?locus=ATF2)*,* [*AUR1*](http://db.yeastgenome.org/cgi-bin/locus.pl?locus=AUR1)*,* [*BCD1*](http://db.yeastgenome.org/cgi-bin/locus.pl?locus=BCD1)*,* [*BIO2*](http://db.yeastgenome.org/cgi-bin/locus.pl?locus=BIO2)*,* [*BMS1*](http://db.yeastgenome.org/cgi-bin/locus.pl?locus=BMS1)*,* [*BST1*](http://db.yeastgenome.org/cgi-bin/locus.pl?locus=BST1)*,* [*BUD21*](http://db.yeastgenome.org/cgi-bin/locus.pl?locus=BUD21)*,* [*BUD32*](http://db.yeastgenome.org/cgi-bin/locus.pl?locus=BUD32)*,* [*CBF5*](http://db.yeastgenome.org/cgi-bin/locus.pl?locus=CBF5)*,* [*CDC14*](http://db.yeastgenome.org/cgi-bin/locus.pl?locus=CDC14)*,* [*CDC20*](http://db.yeastgenome.org/cgi-bin/locus.pl?locus=CDC20)*,* [*CDC47*](http://db.yeastgenome.org/cgi-bin/locus.pl?locus=CDC47)*,* [*CGR1*](http://db.yeastgenome.org/cgi-bin/locus.pl?locus=CGR1)*,* [*CLA4*](http://db.yeastgenome.org/cgi-bin/locus.pl?locus=CLA4)*, CMS1,* [*CSM3*](http://db.yeastgenome.org/cgi-bin/locus.pl?locus=CSM3)*,* [*CUS1*](http://db.yeastgenome.org/cgi-bin/locus.pl?locus=CUS1)*,* [*CWC2*](http://db.yeastgenome.org/cgi-bin/locus.pl?locus=CWC2)*,* [*CYB5*](http://db.yeastgenome.org/cgi-bin/locus.pl?locus=CYB5)*,* [*DBP10*](http://db.yeastgenome.org/cgi-bin/locus.pl?locus=DBP10)*,* [*DBP2*](http://db.yeastgenome.org/cgi-bin/locus.pl?locus=DBP2)*,* [*DBP3*](http://db.yeastgenome.org/cgi-bin/locus.pl?locus=DBP3)*,* [*DBP6*](http://db.yeastgenome.org/cgi-bin/locus.pl?locus=DBP6)*,* [*DBP7*](http://db.yeastgenome.org/cgi-bin/locus.pl?locus=DBP7)*,* [*DBP8*](http://db.yeastgenome.org/cgi-bin/locus.pl?locus=DBP8)*,* [*DBP9*](http://db.yeastgenome.org/cgi-bin/locus.pl?locus=DBP9)*,* [*DFR1*](http://db.yeastgenome.org/cgi-bin/locus.pl?locus=DFR1)*,* [*DIM1*](http://db.yeastgenome.org/cgi-bin/locus.pl?locus=DIM1)*,* [*DIP2*](http://db.yeastgenome.org/cgi-bin/locus.pl?locus=DIP2)*,* [*DPB2*](http://db.yeastgenome.org/cgi-bin/locus.pl?locus=DPB2)*,* [*DPH2*](http://db.yeastgenome.org/cgi-bin/locus.pl?locus=DPH2)*,* [*DRS1*](http://db.yeastgenome.org/cgi-bin/locus.pl?locus=DRS1)*,* [*DUS1*](http://db.yeastgenome.org/cgi-bin/locus.pl?locus=DUS1)*,* [*DUS3*](http://db.yeastgenome.org/cgi-bin/locus.pl?locus=DUS3)*,* [*DUS4*](http://db.yeastgenome.org/cgi-bin/locus.pl?locus=DUS4)*,* [*DUT1*](http://db.yeastgenome.org/cgi-bin/locus.pl?locus=DUT1)*,* [*DYS1*](http://db.yeastgenome.org/cgi-bin/locus.pl?locus=DYS1)*,* [*EBP2*](http://db.yeastgenome.org/cgi-bin/locus.pl?locus=EBP2)*,* [*ECM16*](http://db.yeastgenome.org/cgi-bin/locus.pl?locus=ECM16)*,* [*EFB1*](http://db.yeastgenome.org/cgi-bin/locus.pl?locus=EFB1)*, EFG1,* [*EKI1*](http://db.yeastgenome.org/cgi-bin/locus.pl?locus=EKI1)*,* [*ELP2*](http://db.yeastgenome.org/cgi-bin/locus.pl?locus=ELP2)*,* [*ELP3*](http://db.yeastgenome.org/cgi-bin/locus.pl?locus=ELP3)*,* [*EMG1*](http://db.yeastgenome.org/cgi-bin/locus.pl?locus=EMG1)*,* [*ENP1*](http://db.yeastgenome.org/cgi-bin/locus.pl?locus=ENP1)*,* [*ENP2*](http://db.yeastgenome.org/cgi-bin/locus.pl?locus=ENP2)*,* [*ERB1*](http://db.yeastgenome.org/cgi-bin/locus.pl?locus=ERB1)*,* [*ERG1*](http://db.yeastgenome.org/cgi-bin/locus.pl?locus=ERG1)*,* [*ERG11*](http://db.yeastgenome.org/cgi-bin/locus.pl?locus=ERG11)*,* [*ERG3*](http://db.yeastgenome.org/cgi-bin/locus.pl?locus=ERG3)*,* [*ERG5*](http://db.yeastgenome.org/cgi-bin/locus.pl?locus=ERG5)*,* [*ESF1*](http://db.yeastgenome.org/cgi-bin/locus.pl?locus=ESF1)*,* [*ESF2*](http://db.yeastgenome.org/cgi-bin/locus.pl?locus=ESF2)*,* [*EXG1*](http://db.yeastgenome.org/cgi-bin/locus.pl?locus=EXG1)*,* [*FAA4*](http://db.yeastgenome.org/cgi-bin/locus.pl?locus=FAA4)*,* [*FAF1*](http://db.yeastgenome.org/cgi-bin/locus.pl?locus=FAF1)*,* [*FAL1*](http://db.yeastgenome.org/cgi-bin/locus.pl?locus=FAL1)*,* [*FAP7*](http://db.yeastgenome.org/cgi-bin/locus.pl?locus=FAP7)*,* [*FCF2*](http://db.yeastgenome.org/cgi-bin/locus.pl?locus=FCF2)*,* [*FEN1*](http://db.yeastgenome.org/cgi-bin/locus.pl?locus=FEN1)*,* [*FKH1*](http://db.yeastgenome.org/cgi-bin/locus.pl?locus=FKH1)*,* [*FOL1*](http://db.yeastgenome.org/cgi-bin/locus.pl?locus=FOL1)*,* [*FPR4*](http://db.yeastgenome.org/cgi-bin/locus.pl?locus=FPR4)*,* [*FUR1*](http://db.yeastgenome.org/cgi-bin/locus.pl?locus=FUR1)*,* [*FYV7*](http://db.yeastgenome.org/cgi-bin/locus.pl?locus=FYV7)*,* [*GAR1*](http://db.yeastgenome.org/cgi-bin/locus.pl?locus=GAR1)*,* [*GCD10*](http://db.yeastgenome.org/cgi-bin/locus.pl?locus=GCD10)*,* [*GCD11*](http://db.yeastgenome.org/cgi-bin/locus.pl?locus=GCD11)*,* [*GCD14*](http://db.yeastgenome.org/cgi-bin/locus.pl?locus=GCD14)*,* [*GCD2*](http://db.yeastgenome.org/cgi-bin/locus.pl?locus=GCD2)*,* [*GCN3*](http://db.yeastgenome.org/cgi-bin/locus.pl?locus=GCN3)*,* [*GCR1*](http://db.yeastgenome.org/cgi-bin/locus.pl?locus=GCR1)*,* [*GCR2*](http://db.yeastgenome.org/cgi-bin/locus.pl?locus=GCR2)*,* [*GLN1*](http://db.yeastgenome.org/cgi-bin/locus.pl?locus=GLN1)*,* [*GLN4*](http://db.yeastgenome.org/cgi-bin/locus.pl?locus=GLN4)*,* [*GPI13*](http://db.yeastgenome.org/cgi-bin/locus.pl?locus=GPI13)*,* [*GRC3*](http://db.yeastgenome.org/cgi-bin/locus.pl?locus=GRC3)*,* [*GUA1*](http://db.yeastgenome.org/cgi-bin/locus.pl?locus=GUA1)*,* [*HAS1*](http://db.yeastgenome.org/cgi-bin/locus.pl?locus=HAS1)*,* [*HCA4*](http://db.yeastgenome.org/cgi-bin/locus.pl?locus=HCA4)*,* [*HEM1*](http://db.yeastgenome.org/cgi-bin/locus.pl?locus=HEM1)*,* [*HHF2*](http://db.yeastgenome.org/cgi-bin/locus.pl?locus=HHF2)*,* [*HHT1*](http://db.yeastgenome.org/cgi-bin/locus.pl?locus=HHT1)*,* [*HIR2*](http://db.yeastgenome.org/cgi-bin/locus.pl?locus=HIR2)*,* [*HMG1*](http://db.yeastgenome.org/cgi-bin/locus.pl?locus=HMG1)*,* [*HMT1*](http://db.yeastgenome.org/cgi-bin/locus.pl?locus=HMT1)*,* [*HPT1*](http://db.yeastgenome.org/cgi-bin/locus.pl?locus=HPT1)*,* [*HST3*](http://db.yeastgenome.org/cgi-bin/locus.pl?locus=HST3)*,* [*HTA1*](http://db.yeastgenome.org/cgi-bin/locus.pl?locus=HTA1)*,* [*HTA2*](http://db.yeastgenome.org/cgi-bin/locus.pl?locus=HTA2)*,* [*HTB1*](http://db.yeastgenome.org/cgi-bin/locus.pl?locus=HTB1)*,* [*HTB2*](http://db.yeastgenome.org/cgi-bin/locus.pl?locus=HTB2)*,* [*HTZ1*](http://db.yeastgenome.org/cgi-bin/locus.pl?locus=HTZ1)*,* [*IFH1*](http://db.yeastgenome.org/cgi-bin/locus.pl?locus=IFH1)*,* [*ILV3*](http://db.yeastgenome.org/cgi-bin/locus.pl?locus=ILV3)*,* [*ILV5*](http://db.yeastgenome.org/cgi-bin/locus.pl?locus=ILV5)*,* [*IMD3*](http://db.yeastgenome.org/cgi-bin/locus.pl?locus=IMD3)*,* [*IMP3*](http://db.yeastgenome.org/cgi-bin/locus.pl?locus=IMP3)*,* [*INO2*](http://db.yeastgenome.org/cgi-bin/locus.pl?locus=INO2)*,* [*IPI1*](http://db.yeastgenome.org/cgi-bin/locus.pl?locus=IPI1)*,* [*IPI3*](http://db.yeastgenome.org/cgi-bin/locus.pl?locus=IPI3)*,* [*JJJ3*](http://db.yeastgenome.org/cgi-bin/locus.pl?locus=JJJ3)*,* [*KAE1*](http://db.yeastgenome.org/cgi-bin/locus.pl?locus=KAE1)*,* [*KCS1*](http://db.yeastgenome.org/cgi-bin/locus.pl?locus=KCS1)*,* [*KRR1*](http://db.yeastgenome.org/cgi-bin/locus.pl?locus=KRR1)*,* [*KTI12*](http://db.yeastgenome.org/cgi-bin/locus.pl?locus=KTI12)*,* [*KTR5*](http://db.yeastgenome.org/cgi-bin/locus.pl?locus=KTR5)*,* [*LCP5*](http://db.yeastgenome.org/cgi-bin/locus.pl?locus=LCP5)*,* [*LEU9*](http://db.yeastgenome.org/cgi-bin/locus.pl?locus=LEU9)*,* [*LIA1*](http://db.yeastgenome.org/cgi-bin/locus.pl?locus=LIA1)*,* [*LYS12*](http://db.yeastgenome.org/cgi-bin/locus.pl?locus=LYS12)*,* [*LYS4*](http://db.yeastgenome.org/cgi-bin/locus.pl?locus=LYS4)*,* [*MAE1*](http://db.yeastgenome.org/cgi-bin/locus.pl?locus=MAE1)*,* [*MAK11*](http://db.yeastgenome.org/cgi-bin/locus.pl?locus=MAK11)*,* [*MAK16*](http://db.yeastgenome.org/cgi-bin/locus.pl?locus=MAK16)*,* [*MAK3*](http://db.yeastgenome.org/cgi-bin/locus.pl?locus=MAK3)*,* [*MAP1*](http://db.yeastgenome.org/cgi-bin/locus.pl?locus=MAP1)*,* [*MES1*](http://db.yeastgenome.org/cgi-bin/locus.pl?locus=MES1)*,* [*MEU1*](http://db.yeastgenome.org/cgi-bin/locus.pl?locus=MEU1)*,* [*MIS1*](http://db.yeastgenome.org/cgi-bin/locus.pl?locus=MIS1)*,* [*MNN2*](http://db.yeastgenome.org/cgi-bin/locus.pl?locus=MNN2)*,* [*MOT1*](http://db.yeastgenome.org/cgi-bin/locus.pl?locus=MOT1)*,* [*MPP10*](http://db.yeastgenome.org/cgi-bin/locus.pl?locus=MPP10)*,* [*MRD1*](http://db.yeastgenome.org/cgi-bin/locus.pl?locus=MRD1)*,* [*MRT4*](http://db.yeastgenome.org/cgi-bin/locus.pl?locus=MRT4)*,* [*MSH1*](http://db.yeastgenome.org/cgi-bin/locus.pl?locus=MSH1)*,* [*MSH6*](http://db.yeastgenome.org/cgi-bin/locus.pl?locus=MSH6)*,* [*MTD1*](http://db.yeastgenome.org/cgi-bin/locus.pl?locus=MTD1)*,* [*MTR3*](http://db.yeastgenome.org/cgi-bin/locus.pl?locus=MTR3)*,* [*NAF1*](http://db.yeastgenome.org/cgi-bin/locus.pl?locus=NAF1)*,* [*NAN1*](http://db.yeastgenome.org/cgi-bin/locus.pl?locus=NAN1)*,* [*NCL1*](http://db.yeastgenome.org/cgi-bin/locus.pl?locus=NCL1)*,* [*NCS2*](http://db.yeastgenome.org/cgi-bin/locus.pl?locus=NCS2)*,* [*NHP2*](http://db.yeastgenome.org/cgi-bin/locus.pl?locus=NHP2)*,* [*NIP7*](http://db.yeastgenome.org/cgi-bin/locus.pl?locus=NIP7)*,* [*NOB1*](http://db.yeastgenome.org/cgi-bin/locus.pl?locus=NOB1)*,* [*NOC3*](http://db.yeastgenome.org/cgi-bin/locus.pl?locus=NOC3)*,* [*NOC4*](http://db.yeastgenome.org/cgi-bin/locus.pl?locus=NOC4)*,* [*NOG1*](http://db.yeastgenome.org/cgi-bin/locus.pl?locus=NOG1)*,* [*NOP12*](http://db.yeastgenome.org/cgi-bin/locus.pl?locus=NOP12)*,* [*NOP14*](http://db.yeastgenome.org/cgi-bin/locus.pl?locus=NOP14)*,* [*NOP2*](http://db.yeastgenome.org/cgi-bin/locus.pl?locus=NOP2)*,* [*NOP4*](http://db.yeastgenome.org/cgi-bin/locus.pl?locus=NOP4)*,* [*NOP53*](http://db.yeastgenome.org/cgi-bin/locus.pl?locus=NOP53)*,* [*NOP58*](http://db.yeastgenome.org/cgi-bin/locus.pl?locus=NOP58)*,* [*NOP6*](http://db.yeastgenome.org/cgi-bin/locus.pl?locus=NOP6)*,* [*NOP7*](http://db.yeastgenome.org/cgi-bin/locus.pl?locus=NOP7)*,* [*NOP8*](http://db.yeastgenome.org/cgi-bin/locus.pl?locus=NOP8)*,* [*NOP9*](http://db.yeastgenome.org/cgi-bin/locus.pl?locus=NOP9)*,* [*NSE4*](http://db.yeastgenome.org/cgi-bin/locus.pl?locus=NSE4)*,* [*NSR1*](http://db.yeastgenome.org/cgi-bin/locus.pl?locus=NSR1)*,* [*NUC1*](http://db.yeastgenome.org/cgi-bin/locus.pl?locus=NUC1)*,* [*NUG1*](http://db.yeastgenome.org/cgi-bin/locus.pl?locus=NUG1)*,* [*ORC1*](http://db.yeastgenome.org/cgi-bin/locus.pl?locus=ORC1)*,* [*PHO11*](http://db.yeastgenome.org/cgi-bin/locus.pl?locus=PHO11)*,* [*PHO3*](http://db.yeastgenome.org/cgi-bin/locus.pl?locus=PHO3)*,* [*PHO5*](http://db.yeastgenome.org/cgi-bin/locus.pl?locus=PHO5)*,* [*PLB2*](http://db.yeastgenome.org/cgi-bin/locus.pl?locus=PLB2)*,* [*PMT2*](http://db.yeastgenome.org/cgi-bin/locus.pl?locus=PMT2)*,* [*PNO1*](http://db.yeastgenome.org/cgi-bin/locus.pl?locus=PNO1)*,* [*POL5*](http://db.yeastgenome.org/cgi-bin/locus.pl?locus=POL5)*,* [*POP3*](http://db.yeastgenome.org/cgi-bin/locus.pl?locus=POP3)*,* [*POP6*](http://db.yeastgenome.org/cgi-bin/locus.pl?locus=POP6)*,* [*POP8*](http://db.yeastgenome.org/cgi-bin/locus.pl?locus=POP8)*,* [*PPT1*](http://db.yeastgenome.org/cgi-bin/locus.pl?locus=PPT1)*,* [*PRO1*](http://db.yeastgenome.org/cgi-bin/locus.pl?locus=PRO1)*,* [*PRP19*](http://db.yeastgenome.org/cgi-bin/locus.pl?locus=PRP19)*,* [*PRP24*](http://db.yeastgenome.org/cgi-bin/locus.pl?locus=PRP24)*,* [*PRP43*](http://db.yeastgenome.org/cgi-bin/locus.pl?locus=PRP43)*,* [*PRS1*](http://db.yeastgenome.org/cgi-bin/locus.pl?locus=PRS1)*,* [*PRS2*](http://db.yeastgenome.org/cgi-bin/locus.pl?locus=PRS2)*,* [*PRS4*](http://db.yeastgenome.org/cgi-bin/locus.pl?locus=PRS4)*,* [*PSY4*](http://db.yeastgenome.org/cgi-bin/locus.pl?locus=PSY4)*,* [*PUF6*](http://db.yeastgenome.org/cgi-bin/locus.pl?locus=PUF6)*,* [*PUS1*](http://db.yeastgenome.org/cgi-bin/locus.pl?locus=PUS1)*,* [*PUS4*](http://db.yeastgenome.org/cgi-bin/locus.pl?locus=PUS4)*,* [*PUS7*](http://db.yeastgenome.org/cgi-bin/locus.pl?locus=PUS7)*,* [*PWP1*](http://db.yeastgenome.org/cgi-bin/locus.pl?locus=PWP1)*,* [*PWP2*](http://db.yeastgenome.org/cgi-bin/locus.pl?locus=PWP2)*,* [*PXR1*](http://db.yeastgenome.org/cgi-bin/locus.pl?locus=PXR1)*,* [*RAP1*](http://db.yeastgenome.org/cgi-bin/locus.pl?locus=RAP1)*,* [*RBA50*](http://db.yeastgenome.org/cgi-bin/locus.pl?locus=RBA50)*,* [*RCL1*](http://db.yeastgenome.org/cgi-bin/locus.pl?locus=RCL1)*,* [*RET1*](http://db.yeastgenome.org/cgi-bin/locus.pl?locus=RET1)*,* [*REX4*](http://db.yeastgenome.org/cgi-bin/locus.pl?locus=REX4)*,* [*RHR2*](http://db.yeastgenome.org/cgi-bin/locus.pl?locus=RHR2)*,* [*RIO1*](http://db.yeastgenome.org/cgi-bin/locus.pl?locus=RIO1)*,* [*RIX1*](http://db.yeastgenome.org/cgi-bin/locus.pl?locus=RIX1)*,* [*RKI1*](http://db.yeastgenome.org/cgi-bin/locus.pl?locus=RKI1)*,* [*RLI1*](http://db.yeastgenome.org/cgi-bin/locus.pl?locus=RLI1)*,* [*RLP7*](http://db.yeastgenome.org/cgi-bin/locus.pl?locus=RLP7)*,* [*RMT2*](http://db.yeastgenome.org/cgi-bin/locus.pl?locus=RMT2)*,* [*RNH201*](http://db.yeastgenome.org/cgi-bin/locus.pl?locus=RNH201)*,* [*RNT1*](http://db.yeastgenome.org/cgi-bin/locus.pl?locus=RNT1)*,* [*ROK1*](http://db.yeastgenome.org/cgi-bin/locus.pl?locus=ROK1)*,* [*ROX3*](http://db.yeastgenome.org/cgi-bin/locus.pl?locus=ROX3)*,* [*RPA12*](http://db.yeastgenome.org/cgi-bin/locus.pl?locus=RPA12)*,* [*RPA190*](http://db.yeastgenome.org/cgi-bin/locus.pl?locus=RPA190)*,* [*RPA34*](http://db.yeastgenome.org/cgi-bin/locus.pl?locus=RPA34)*,* [*RPA43*](http://db.yeastgenome.org/cgi-bin/locus.pl?locus=RPA43)*,* [*RPA49*](http://db.yeastgenome.org/cgi-bin/locus.pl?locus=RPA49)*,* [*RPB10*](http://db.yeastgenome.org/cgi-bin/locus.pl?locus=RPB10)*,* [*RPB5*](http://db.yeastgenome.org/cgi-bin/locus.pl?locus=RPB5)*,* [*RPB8*](http://db.yeastgenome.org/cgi-bin/locus.pl?locus=RPB8)*,* [*RPB9*](http://db.yeastgenome.org/cgi-bin/locus.pl?locus=RPB9)*,* [*RPC11*](http://db.yeastgenome.org/cgi-bin/locus.pl?locus=RPC11)*,* [*RPC19*](http://db.yeastgenome.org/cgi-bin/locus.pl?locus=RPC19)*,* [*RPC31*](http://db.yeastgenome.org/cgi-bin/locus.pl?locus=RPC31)*,* [*RPC34*](http://db.yeastgenome.org/cgi-bin/locus.pl?locus=RPC34)*,* [*RPC37*](http://db.yeastgenome.org/cgi-bin/locus.pl?locus=RPC37)*,* [*RPC40*](http://db.yeastgenome.org/cgi-bin/locus.pl?locus=RPC40)*,* [*RPC53*](http://db.yeastgenome.org/cgi-bin/locus.pl?locus=RPC53)*,* [*RPC82*](http://db.yeastgenome.org/cgi-bin/locus.pl?locus=RPC82)*,* [*RPF1*](http://db.yeastgenome.org/cgi-bin/locus.pl?locus=RPF1)*,* [*RPF2*](http://db.yeastgenome.org/cgi-bin/locus.pl?locus=RPF2)*,* [*RPL13A*](http://db.yeastgenome.org/cgi-bin/locus.pl?locus=RPL13A)*,* [*RPL14A*](http://db.yeastgenome.org/cgi-bin/locus.pl?locus=RPL14A)*,* [*RPL14B*](http://db.yeastgenome.org/cgi-bin/locus.pl?locus=RPL14B)*,* [*RPL18B*](http://db.yeastgenome.org/cgi-bin/locus.pl?locus=RPL18B)*,* [*RPL20A*](http://db.yeastgenome.org/cgi-bin/locus.pl?locus=RPL20A)*,* [*RPL21B*](http://db.yeastgenome.org/cgi-bin/locus.pl?locus=RPL21B)*,* [*RPL26A*](http://db.yeastgenome.org/cgi-bin/locus.pl?locus=RPL26A)*,* [*RPL31B*](http://db.yeastgenome.org/cgi-bin/locus.pl?locus=RPL31B)*,* [*RPL33B*](http://db.yeastgenome.org/cgi-bin/locus.pl?locus=RPL33B)*,* [*RPL36A*](http://db.yeastgenome.org/cgi-bin/locus.pl?locus=RPL36A)*,* [*RPL43B*](http://db.yeastgenome.org/cgi-bin/locus.pl?locus=RPL43B)*,* [*RPL7B*](http://db.yeastgenome.org/cgi-bin/locus.pl?locus=RPL7B)*,* [*RPO26*](http://db.yeastgenome.org/cgi-bin/locus.pl?locus=RPO26)*,* [*RPP1*](http://db.yeastgenome.org/cgi-bin/locus.pl?locus=RPP1)*,* [*RPS10B*](http://db.yeastgenome.org/cgi-bin/locus.pl?locus=RPS10B)*,* [*RPS14B*](http://db.yeastgenome.org/cgi-bin/locus.pl?locus=RPS14B)*,* [*RPS19B*](http://db.yeastgenome.org/cgi-bin/locus.pl?locus=RPS19B)*,* [*RPS1A*](http://db.yeastgenome.org/cgi-bin/locus.pl?locus=RPS1A)*,* [*RPS21A*](http://db.yeastgenome.org/cgi-bin/locus.pl?locus=RPS21A)*,* [*RPS21B*](http://db.yeastgenome.org/cgi-bin/locus.pl?locus=RPS21B)*,* [*RPS26B*](http://db.yeastgenome.org/cgi-bin/locus.pl?locus=RPS26B)*,* [*RPS27A*](http://db.yeastgenome.org/cgi-bin/locus.pl?locus=RPS27A)*,* [*RPS29A*](http://db.yeastgenome.org/cgi-bin/locus.pl?locus=RPS29A)*,* [*RPS7A*](http://db.yeastgenome.org/cgi-bin/locus.pl?locus=RPS7A)*,* [*RPS7B*](http://db.yeastgenome.org/cgi-bin/locus.pl?locus=RPS7B)*,* [*RRM3*](http://db.yeastgenome.org/cgi-bin/locus.pl?locus=RRM3)*,* [*RRN11*](http://db.yeastgenome.org/cgi-bin/locus.pl?locus=RRN11)*,* [*RRN7*](http://db.yeastgenome.org/cgi-bin/locus.pl?locus=RRN7)*,* [*RRP1*](http://db.yeastgenome.org/cgi-bin/locus.pl?locus=RRP1)*,* [*RRP12*](http://db.yeastgenome.org/cgi-bin/locus.pl?locus=RRP12)*,* [*RRP15*](http://db.yeastgenome.org/cgi-bin/locus.pl?locus=RRP15)*,* [*RRP17*](http://db.yeastgenome.org/cgi-bin/locus.pl?locus=RRP17)*,* [*RRP3*](http://db.yeastgenome.org/cgi-bin/locus.pl?locus=RRP3)*, RRP36,* [*RRP40*](http://db.yeastgenome.org/cgi-bin/locus.pl?locus=RRP40)*,* [*RRP43*](http://db.yeastgenome.org/cgi-bin/locus.pl?locus=RRP43)*,* [*RRP5*](http://db.yeastgenome.org/cgi-bin/locus.pl?locus=RRP5)*,* [*RRP6*](http://db.yeastgenome.org/cgi-bin/locus.pl?locus=RRP6)*,* [*RRP8*](http://db.yeastgenome.org/cgi-bin/locus.pl?locus=RRP8)*,* [*RRP9*](http://db.yeastgenome.org/cgi-bin/locus.pl?locus=RRP9)*,* [*RRS1*](http://db.yeastgenome.org/cgi-bin/locus.pl?locus=RRS1)*,* [*RVB1*](http://db.yeastgenome.org/cgi-bin/locus.pl?locus=RVB1)*,* [*SAN1*](http://db.yeastgenome.org/cgi-bin/locus.pl?locus=SAN1)*,* [*SAS10*](http://db.yeastgenome.org/cgi-bin/locus.pl?locus=SAS10)*,* [*SCC2*](http://db.yeastgenome.org/cgi-bin/locus.pl?locus=SCC2)*,* [*SCH9*](http://db.yeastgenome.org/cgi-bin/locus.pl?locus=SCH9)*,* [*SCS7*](http://db.yeastgenome.org/cgi-bin/locus.pl?locus=SCS7)*,* [*SEN34*](http://db.yeastgenome.org/cgi-bin/locus.pl?locus=SEN34)*,* [*SFG1*](http://db.yeastgenome.org/cgi-bin/locus.pl?locus=SFG1)*,* [*SHQ1*](http://db.yeastgenome.org/cgi-bin/locus.pl?locus=SHQ1)*,* [*SIK1*](http://db.yeastgenome.org/cgi-bin/locus.pl?locus=SIK1)*,* [*SIR2*](http://db.yeastgenome.org/cgi-bin/locus.pl?locus=SIR2)*,* [*SLI15*](http://db.yeastgenome.org/cgi-bin/locus.pl?locus=SLI15)*,* [*SLN1*](http://db.yeastgenome.org/cgi-bin/locus.pl?locus=SLN1)*,* [*SLX9*](http://db.yeastgenome.org/cgi-bin/locus.pl?locus=SLX9)*,* [*SMI1*](http://db.yeastgenome.org/cgi-bin/locus.pl?locus=SMI1)*,* [*SNU13*](http://db.yeastgenome.org/cgi-bin/locus.pl?locus=SNU13)*,* [*SOF1*](http://db.yeastgenome.org/cgi-bin/locus.pl?locus=SOF1)*,* [*SPB1*](http://db.yeastgenome.org/cgi-bin/locus.pl?locus=SPB1)*,* [*SPB4*](http://db.yeastgenome.org/cgi-bin/locus.pl?locus=SPB4)*,* [*SPE4*](http://db.yeastgenome.org/cgi-bin/locus.pl?locus=SPE4)*,* [*SRO9*](http://db.yeastgenome.org/cgi-bin/locus.pl?locus=SRO9)*,* [*STE12*](http://db.yeastgenome.org/cgi-bin/locus.pl?locus=STE12)*,* [*SUR2*](http://db.yeastgenome.org/cgi-bin/locus.pl?locus=SUR2)*,* [*SUR4*](http://db.yeastgenome.org/cgi-bin/locus.pl?locus=SUR4)*,* [*SUT1*](http://db.yeastgenome.org/cgi-bin/locus.pl?locus=SUT1)*,* [*SUT2*](http://db.yeastgenome.org/cgi-bin/locus.pl?locus=SUT2)*,* [*SUV3*](http://db.yeastgenome.org/cgi-bin/locus.pl?locus=SUV3)*,* [*SVF1*](http://db.yeastgenome.org/cgi-bin/locus.pl?locus=SVF1)*,* [*SWI5*](http://db.yeastgenome.org/cgi-bin/locus.pl?locus=SWI5)*,* [*TAD3*](http://db.yeastgenome.org/cgi-bin/locus.pl?locus=TAD3)*,* [*THI21*](http://db.yeastgenome.org/cgi-bin/locus.pl?locus=THI21)*,* [*THP2*](http://db.yeastgenome.org/cgi-bin/locus.pl?locus=THP2)*,* [*TIF2*](http://db.yeastgenome.org/cgi-bin/locus.pl?locus=TIF2)*,* [*TIF3*](http://db.yeastgenome.org/cgi-bin/locus.pl?locus=TIF3)*,* [*TIF35*](http://db.yeastgenome.org/cgi-bin/locus.pl?locus=TIF35)*,* [*TIF4631*](http://db.yeastgenome.org/cgi-bin/locus.pl?locus=TIF4631)*,* [*TOP1*](http://db.yeastgenome.org/cgi-bin/locus.pl?locus=TOP1)*,* [*TOS4*](http://db.yeastgenome.org/cgi-bin/locus.pl?locus=TOS4)*,* [*TPA1*](http://db.yeastgenome.org/cgi-bin/locus.pl?locus=TPA1)*,* [*TRF5*](http://db.yeastgenome.org/cgi-bin/locus.pl?locus=TRF5)*,* [*TRM1*](http://db.yeastgenome.org/cgi-bin/locus.pl?locus=TRM1)*,* [*TRM10*](http://db.yeastgenome.org/cgi-bin/locus.pl?locus=TRM10)*,* [*TRM11*](http://db.yeastgenome.org/cgi-bin/locus.pl?locus=TRM11)*,* [*TRM112*](http://db.yeastgenome.org/cgi-bin/locus.pl?locus=TRM112)*,* [*TRM13*](http://db.yeastgenome.org/cgi-bin/locus.pl?locus=TRM13)*,* [*TRM2*](http://db.yeastgenome.org/cgi-bin/locus.pl?locus=TRM2)*,* [*TRM3*](http://db.yeastgenome.org/cgi-bin/locus.pl?locus=TRM3)*,* [*TRM7*](http://db.yeastgenome.org/cgi-bin/locus.pl?locus=TRM7)*,* [*TRM8*](http://db.yeastgenome.org/cgi-bin/locus.pl?locus=TRM8)*,* [*TRM82*](http://db.yeastgenome.org/cgi-bin/locus.pl?locus=TRM82)*,* [*TRM9*](http://db.yeastgenome.org/cgi-bin/locus.pl?locus=TRM9)*,* [*TSC10*](http://db.yeastgenome.org/cgi-bin/locus.pl?locus=TSC10)*,* [*TSR1*](http://db.yeastgenome.org/cgi-bin/locus.pl?locus=TSR1)*,* [*TSR2*](http://db.yeastgenome.org/cgi-bin/locus.pl?locus=TSR2)*,* [*TYW1*](http://db.yeastgenome.org/cgi-bin/locus.pl?locus=TYW1)*,* [*UBC9*](http://db.yeastgenome.org/cgi-bin/locus.pl?locus=UBC9)*,* [*UBP10*](http://db.yeastgenome.org/cgi-bin/locus.pl?locus=UBP10)*,* [*UNG1*](http://db.yeastgenome.org/cgi-bin/locus.pl?locus=UNG1)*,* [*URA4*](http://db.yeastgenome.org/cgi-bin/locus.pl?locus=URA4)*,* [*URA5*](http://db.yeastgenome.org/cgi-bin/locus.pl?locus=URA5)*,* [*URA7*](http://db.yeastgenome.org/cgi-bin/locus.pl?locus=URA7)*,* [*URB1*](http://db.yeastgenome.org/cgi-bin/locus.pl?locus=URB1)*,* [*URB2*](http://db.yeastgenome.org/cgi-bin/locus.pl?locus=URB2)*,* [*URK1*](http://db.yeastgenome.org/cgi-bin/locus.pl?locus=URK1)*,* [*UTP10*](http://db.yeastgenome.org/cgi-bin/locus.pl?locus=UTP10)*,* [*UTP11*](http://db.yeastgenome.org/cgi-bin/locus.pl?locus=UTP11)*,* [*UTP13*](http://db.yeastgenome.org/cgi-bin/locus.pl?locus=UTP13)*,* [*UTP14*](http://db.yeastgenome.org/cgi-bin/locus.pl?locus=UTP14)*,* [*UTP15*](http://db.yeastgenome.org/cgi-bin/locus.pl?locus=UTP15)*,* [*UTP18*](http://db.yeastgenome.org/cgi-bin/locus.pl?locus=UTP18)*,* [*UTP21*](http://db.yeastgenome.org/cgi-bin/locus.pl?locus=UTP21)*,* [*UTP22*](http://db.yeastgenome.org/cgi-bin/locus.pl?locus=UTP22)*,* [*UTP23*](http://db.yeastgenome.org/cgi-bin/locus.pl?locus=UTP23)*,* [*UTP30*](http://db.yeastgenome.org/cgi-bin/locus.pl?locus=UTP30)*,* [*UTP4*](http://db.yeastgenome.org/cgi-bin/locus.pl?locus=UTP4)*,* [*UTP5*](http://db.yeastgenome.org/cgi-bin/locus.pl?locus=UTP5)*,* [*UTP7*](http://db.yeastgenome.org/cgi-bin/locus.pl?locus=UTP7)*,* [*UTP8*](http://db.yeastgenome.org/cgi-bin/locus.pl?locus=UTP8)*,* [*UTP9*](http://db.yeastgenome.org/cgi-bin/locus.pl?locus=UTP9)*,* [*VRG4*](http://db.yeastgenome.org/cgi-bin/locus.pl?locus=VRG4)*,* [*WRS1*](http://db.yeastgenome.org/cgi-bin/locus.pl?locus=WRS1)*,* [*YAR1*](http://db.yeastgenome.org/cgi-bin/locus.pl?locus=YAR1)*,* [*YEF3*](http://db.yeastgenome.org/cgi-bin/locus.pl?locus=YEF3)*,* [*YHR020W*](http://db.yeastgenome.org/cgi-bin/locus.pl?locus=YHR020W)*,* [*YML082W*](http://db.yeastgenome.org/cgi-bin/locus.pl?locus=YML082W)*,* [*YNL247W*](http://db.yeastgenome.org/cgi-bin/locus.pl?locus=YNL247W)*,* [*YOX1*](http://db.yeastgenome.org/cgi-bin/locus.pl?locus=YOX1)*,* [*YRF1-6*](http://db.yeastgenome.org/cgi-bin/locus.pl?locus=YRF1-6)*,* [*YVH1*](http://db.yeastgenome.org/cgi-bin/locus.pl?locus=YVH1)*,* [*ZRC1*](http://db.yeastgenome.org/cgi-bin/locus.pl?locus=ZRC1) |
| Macromolecule metabolic process | 312 out of 558 genes, 55.9% | 4.30e-15 | [*AIR1*](http://db.yeastgenome.org/cgi-bin/locus.pl?locus=AIR1)*,* [*ALK1*](http://db.yeastgenome.org/cgi-bin/locus.pl?locus=ALK1)*,* [*ASC1*](http://db.yeastgenome.org/cgi-bin/locus.pl?locus=ASC1)*,* [*ASH1*](http://db.yeastgenome.org/cgi-bin/locus.pl?locus=ASH1)*,* [*BCD1*](http://db.yeastgenome.org/cgi-bin/locus.pl?locus=BCD1)*,* [*BMS1*](http://db.yeastgenome.org/cgi-bin/locus.pl?locus=BMS1)*,* [*BRX1*](http://db.yeastgenome.org/cgi-bin/locus.pl?locus=BRX1)*,* [*BST1*](http://db.yeastgenome.org/cgi-bin/locus.pl?locus=BST1)*,* [*BUD21*](http://db.yeastgenome.org/cgi-bin/locus.pl?locus=BUD21)*,* [*BUD32*](http://db.yeastgenome.org/cgi-bin/locus.pl?locus=BUD32)*,* [*CBF5*](http://db.yeastgenome.org/cgi-bin/locus.pl?locus=CBF5)*,* [*CDC14*](http://db.yeastgenome.org/cgi-bin/locus.pl?locus=CDC14)*,* [*CDC20*](http://db.yeastgenome.org/cgi-bin/locus.pl?locus=CDC20)*,* [*CDC47*](http://db.yeastgenome.org/cgi-bin/locus.pl?locus=CDC47)*,* [*CGR1*](http://db.yeastgenome.org/cgi-bin/locus.pl?locus=CGR1)*,* [*CIC1*](http://db.yeastgenome.org/cgi-bin/locus.pl?locus=CIC1)*,* [*CLA4*](http://db.yeastgenome.org/cgi-bin/locus.pl?locus=CLA4)*, CMS1,* [*CSM3*](http://db.yeastgenome.org/cgi-bin/locus.pl?locus=CSM3)*,* [*CUS1*](http://db.yeastgenome.org/cgi-bin/locus.pl?locus=CUS1)*,* [*CWC2*](http://db.yeastgenome.org/cgi-bin/locus.pl?locus=CWC2)*,* [*DBP10*](http://db.yeastgenome.org/cgi-bin/locus.pl?locus=DBP10)*,* [*DBP2*](http://db.yeastgenome.org/cgi-bin/locus.pl?locus=DBP2)*,* [*DBP3*](http://db.yeastgenome.org/cgi-bin/locus.pl?locus=DBP3)*,* [*DBP6*](http://db.yeastgenome.org/cgi-bin/locus.pl?locus=DBP6)*,* [*DBP7*](http://db.yeastgenome.org/cgi-bin/locus.pl?locus=DBP7)*,* [*DBP8*](http://db.yeastgenome.org/cgi-bin/locus.pl?locus=DBP8)*,* [*DBP9*](http://db.yeastgenome.org/cgi-bin/locus.pl?locus=DBP9)*,* [*DIM1*](http://db.yeastgenome.org/cgi-bin/locus.pl?locus=DIM1)*,* [*DIP2*](http://db.yeastgenome.org/cgi-bin/locus.pl?locus=DIP2)*,* [*DPB2*](http://db.yeastgenome.org/cgi-bin/locus.pl?locus=DPB2)*,* [*DPH2*](http://db.yeastgenome.org/cgi-bin/locus.pl?locus=DPH2)*,* [*DRS1*](http://db.yeastgenome.org/cgi-bin/locus.pl?locus=DRS1)*,* [*DUS1*](http://db.yeastgenome.org/cgi-bin/locus.pl?locus=DUS1)*,* [*DUS3*](http://db.yeastgenome.org/cgi-bin/locus.pl?locus=DUS3)*,* [*DUS4*](http://db.yeastgenome.org/cgi-bin/locus.pl?locus=DUS4)*,* [*DYS1*](http://db.yeastgenome.org/cgi-bin/locus.pl?locus=DYS1)*,* [*EBP2*](http://db.yeastgenome.org/cgi-bin/locus.pl?locus=EBP2)*,* [*ECM16*](http://db.yeastgenome.org/cgi-bin/locus.pl?locus=ECM16)*,* [*EFB1*](http://db.yeastgenome.org/cgi-bin/locus.pl?locus=EFB1)*, EFG1,* [*ELP2*](http://db.yeastgenome.org/cgi-bin/locus.pl?locus=ELP2)*,* [*ELP3*](http://db.yeastgenome.org/cgi-bin/locus.pl?locus=ELP3)*,* [*EMG1*](http://db.yeastgenome.org/cgi-bin/locus.pl?locus=EMG1)*,* [*ENP1*](http://db.yeastgenome.org/cgi-bin/locus.pl?locus=ENP1)*,* [*ENP2*](http://db.yeastgenome.org/cgi-bin/locus.pl?locus=ENP2)*,* [*ERB1*](http://db.yeastgenome.org/cgi-bin/locus.pl?locus=ERB1)*,* [*ESF1*](http://db.yeastgenome.org/cgi-bin/locus.pl?locus=ESF1)*,* [*ESF2*](http://db.yeastgenome.org/cgi-bin/locus.pl?locus=ESF2)*,* [*EXG1*](http://db.yeastgenome.org/cgi-bin/locus.pl?locus=EXG1)*,* [*FAA4*](http://db.yeastgenome.org/cgi-bin/locus.pl?locus=FAA4)*,* [*FAF1*](http://db.yeastgenome.org/cgi-bin/locus.pl?locus=FAF1)*,* [*FAL1*](http://db.yeastgenome.org/cgi-bin/locus.pl?locus=FAL1)*,* [*FAP7*](http://db.yeastgenome.org/cgi-bin/locus.pl?locus=FAP7)*,* [*FCF2*](http://db.yeastgenome.org/cgi-bin/locus.pl?locus=FCF2)*,* [*FKH1*](http://db.yeastgenome.org/cgi-bin/locus.pl?locus=FKH1)*,* [*FPR4*](http://db.yeastgenome.org/cgi-bin/locus.pl?locus=FPR4)*,* [*FYV7*](http://db.yeastgenome.org/cgi-bin/locus.pl?locus=FYV7)*,* [*GAR1*](http://db.yeastgenome.org/cgi-bin/locus.pl?locus=GAR1)*,* [*GCD10*](http://db.yeastgenome.org/cgi-bin/locus.pl?locus=GCD10)*,* [*GCD11*](http://db.yeastgenome.org/cgi-bin/locus.pl?locus=GCD11)*,* [*GCD14*](http://db.yeastgenome.org/cgi-bin/locus.pl?locus=GCD14)*,* [*GCD2*](http://db.yeastgenome.org/cgi-bin/locus.pl?locus=GCD2)*,* [*GCN3*](http://db.yeastgenome.org/cgi-bin/locus.pl?locus=GCN3)*,* [*GCR1*](http://db.yeastgenome.org/cgi-bin/locus.pl?locus=GCR1)*,* [*GCR2*](http://db.yeastgenome.org/cgi-bin/locus.pl?locus=GCR2)*,* [*GLN4*](http://db.yeastgenome.org/cgi-bin/locus.pl?locus=GLN4)*,* [*GPI13*](http://db.yeastgenome.org/cgi-bin/locus.pl?locus=GPI13)*,* [*GRC3*](http://db.yeastgenome.org/cgi-bin/locus.pl?locus=GRC3)*,* [*HAS1*](http://db.yeastgenome.org/cgi-bin/locus.pl?locus=HAS1)*,* [*HCA4*](http://db.yeastgenome.org/cgi-bin/locus.pl?locus=HCA4)*,* [*HHF2*](http://db.yeastgenome.org/cgi-bin/locus.pl?locus=HHF2)*,* [*HHT1*](http://db.yeastgenome.org/cgi-bin/locus.pl?locus=HHT1)*,* [*HIR2*](http://db.yeastgenome.org/cgi-bin/locus.pl?locus=HIR2)*,* [*HMT1*](http://db.yeastgenome.org/cgi-bin/locus.pl?locus=HMT1)*,* [*HST3*](http://db.yeastgenome.org/cgi-bin/locus.pl?locus=HST3)*,* [*HTA1*](http://db.yeastgenome.org/cgi-bin/locus.pl?locus=HTA1)*,* [*HTA2*](http://db.yeastgenome.org/cgi-bin/locus.pl?locus=HTA2)*,* [*HTB1*](http://db.yeastgenome.org/cgi-bin/locus.pl?locus=HTB1)*,* [*HTB2*](http://db.yeastgenome.org/cgi-bin/locus.pl?locus=HTB2)*,* [*HTZ1*](http://db.yeastgenome.org/cgi-bin/locus.pl?locus=HTZ1)*,* [*IFH1*](http://db.yeastgenome.org/cgi-bin/locus.pl?locus=IFH1)*,* [*IMP3*](http://db.yeastgenome.org/cgi-bin/locus.pl?locus=IMP3)*,* [*INO2*](http://db.yeastgenome.org/cgi-bin/locus.pl?locus=INO2)*,* [*IPI1*](http://db.yeastgenome.org/cgi-bin/locus.pl?locus=IPI1)*,* [*IPI3*](http://db.yeastgenome.org/cgi-bin/locus.pl?locus=IPI3)*,* [*JJJ3*](http://db.yeastgenome.org/cgi-bin/locus.pl?locus=JJJ3)*,* [*KAE1*](http://db.yeastgenome.org/cgi-bin/locus.pl?locus=KAE1)*,* [*KRR1*](http://db.yeastgenome.org/cgi-bin/locus.pl?locus=KRR1)*,* [*KTI12*](http://db.yeastgenome.org/cgi-bin/locus.pl?locus=KTI12)*,* [*KTR5*](http://db.yeastgenome.org/cgi-bin/locus.pl?locus=KTR5)*,* [*LCP5*](http://db.yeastgenome.org/cgi-bin/locus.pl?locus=LCP5)*,* [*MAK11*](http://db.yeastgenome.org/cgi-bin/locus.pl?locus=MAK11)*,* [*MAK16*](http://db.yeastgenome.org/cgi-bin/locus.pl?locus=MAK16)*,* [*MAK21*](http://db.yeastgenome.org/cgi-bin/locus.pl?locus=MAK21)*,* [*MAK3*](http://db.yeastgenome.org/cgi-bin/locus.pl?locus=MAK3)*,* [*MAP1*](http://db.yeastgenome.org/cgi-bin/locus.pl?locus=MAP1)*,* [*MES1*](http://db.yeastgenome.org/cgi-bin/locus.pl?locus=MES1)*,* [*MIS1*](http://db.yeastgenome.org/cgi-bin/locus.pl?locus=MIS1)*,* [*MNN2*](http://db.yeastgenome.org/cgi-bin/locus.pl?locus=MNN2)*,* [*MOT1*](http://db.yeastgenome.org/cgi-bin/locus.pl?locus=MOT1)*,* [*MPP10*](http://db.yeastgenome.org/cgi-bin/locus.pl?locus=MPP10)*,* [*MRD1*](http://db.yeastgenome.org/cgi-bin/locus.pl?locus=MRD1)*,* [*MRT4*](http://db.yeastgenome.org/cgi-bin/locus.pl?locus=MRT4)*,* [*MSH1*](http://db.yeastgenome.org/cgi-bin/locus.pl?locus=MSH1)*,* [*MSH6*](http://db.yeastgenome.org/cgi-bin/locus.pl?locus=MSH6)*,* [*MTR3*](http://db.yeastgenome.org/cgi-bin/locus.pl?locus=MTR3)*,* [*NAF1*](http://db.yeastgenome.org/cgi-bin/locus.pl?locus=NAF1)*,* [*NAN1*](http://db.yeastgenome.org/cgi-bin/locus.pl?locus=NAN1)*,* [*NCL1*](http://db.yeastgenome.org/cgi-bin/locus.pl?locus=NCL1)*,* [*NCS2*](http://db.yeastgenome.org/cgi-bin/locus.pl?locus=NCS2)*,* [*NHP2*](http://db.yeastgenome.org/cgi-bin/locus.pl?locus=NHP2)*,* [*NIP7*](http://db.yeastgenome.org/cgi-bin/locus.pl?locus=NIP7)*,* [*NMD3*](http://db.yeastgenome.org/cgi-bin/locus.pl?locus=NMD3)*,* [*NOB1*](http://db.yeastgenome.org/cgi-bin/locus.pl?locus=NOB1)*,* [*NOC2*](http://db.yeastgenome.org/cgi-bin/locus.pl?locus=NOC2)*,* [*NOC3*](http://db.yeastgenome.org/cgi-bin/locus.pl?locus=NOC3)*,* [*NOC4*](http://db.yeastgenome.org/cgi-bin/locus.pl?locus=NOC4)*,* [*NOG1*](http://db.yeastgenome.org/cgi-bin/locus.pl?locus=NOG1)*,* [*NOG2*](http://db.yeastgenome.org/cgi-bin/locus.pl?locus=NOG2)*,* [*NOP12*](http://db.yeastgenome.org/cgi-bin/locus.pl?locus=NOP12)*,* [*NOP14*](http://db.yeastgenome.org/cgi-bin/locus.pl?locus=NOP14)*,* [*NOP15*](http://db.yeastgenome.org/cgi-bin/locus.pl?locus=NOP15)*,* [*NOP2*](http://db.yeastgenome.org/cgi-bin/locus.pl?locus=NOP2)*,* [*NOP4*](http://db.yeastgenome.org/cgi-bin/locus.pl?locus=NOP4)*,* [*NOP53*](http://db.yeastgenome.org/cgi-bin/locus.pl?locus=NOP53)*,* [*NOP58*](http://db.yeastgenome.org/cgi-bin/locus.pl?locus=NOP58)*,* [*NOP6*](http://db.yeastgenome.org/cgi-bin/locus.pl?locus=NOP6)*,* [*NOP7*](http://db.yeastgenome.org/cgi-bin/locus.pl?locus=NOP7)*,* [*NOP8*](http://db.yeastgenome.org/cgi-bin/locus.pl?locus=NOP8)*,* [*NOP9*](http://db.yeastgenome.org/cgi-bin/locus.pl?locus=NOP9)*,* [*NSE4*](http://db.yeastgenome.org/cgi-bin/locus.pl?locus=NSE4)*,* [*NSR1*](http://db.yeastgenome.org/cgi-bin/locus.pl?locus=NSR1)*,* [*NUC1*](http://db.yeastgenome.org/cgi-bin/locus.pl?locus=NUC1)*,* [*NUG1*](http://db.yeastgenome.org/cgi-bin/locus.pl?locus=NUG1)*,* [*ORC1*](http://db.yeastgenome.org/cgi-bin/locus.pl?locus=ORC1)*,* [*PMT2*](http://db.yeastgenome.org/cgi-bin/locus.pl?locus=PMT2)*,* [*PNO1*](http://db.yeastgenome.org/cgi-bin/locus.pl?locus=PNO1)*,* [*POL5*](http://db.yeastgenome.org/cgi-bin/locus.pl?locus=POL5)*,* [*POP3*](http://db.yeastgenome.org/cgi-bin/locus.pl?locus=POP3)*,* [*POP6*](http://db.yeastgenome.org/cgi-bin/locus.pl?locus=POP6)*,* [*POP8*](http://db.yeastgenome.org/cgi-bin/locus.pl?locus=POP8)*,* [*PPT1*](http://db.yeastgenome.org/cgi-bin/locus.pl?locus=PPT1)*,* [*PRP19*](http://db.yeastgenome.org/cgi-bin/locus.pl?locus=PRP19)*,* [*PRP24*](http://db.yeastgenome.org/cgi-bin/locus.pl?locus=PRP24)*,* [*PRP43*](http://db.yeastgenome.org/cgi-bin/locus.pl?locus=PRP43)*,* [*PSY4*](http://db.yeastgenome.org/cgi-bin/locus.pl?locus=PSY4)*,* [*PUF6*](http://db.yeastgenome.org/cgi-bin/locus.pl?locus=PUF6)*,* [*PUS1*](http://db.yeastgenome.org/cgi-bin/locus.pl?locus=PUS1)*,* [*PUS4*](http://db.yeastgenome.org/cgi-bin/locus.pl?locus=PUS4)*,* [*PUS7*](http://db.yeastgenome.org/cgi-bin/locus.pl?locus=PUS7)*,* [*PWP1*](http://db.yeastgenome.org/cgi-bin/locus.pl?locus=PWP1)*,* [*PWP2*](http://db.yeastgenome.org/cgi-bin/locus.pl?locus=PWP2)*,* [*PXR1*](http://db.yeastgenome.org/cgi-bin/locus.pl?locus=PXR1)*,* [*RAP1*](http://db.yeastgenome.org/cgi-bin/locus.pl?locus=RAP1)*,* [*RBA50*](http://db.yeastgenome.org/cgi-bin/locus.pl?locus=RBA50)*,* [*RCL1*](http://db.yeastgenome.org/cgi-bin/locus.pl?locus=RCL1)*,* [*RET1*](http://db.yeastgenome.org/cgi-bin/locus.pl?locus=RET1)*,* [*REX4*](http://db.yeastgenome.org/cgi-bin/locus.pl?locus=REX4)*,* [*RIO1*](http://db.yeastgenome.org/cgi-bin/locus.pl?locus=RIO1)*,* [*RIX1*](http://db.yeastgenome.org/cgi-bin/locus.pl?locus=RIX1)*,* [*RKI1*](http://db.yeastgenome.org/cgi-bin/locus.pl?locus=RKI1)*,* [*RLI1*](http://db.yeastgenome.org/cgi-bin/locus.pl?locus=RLI1)*,* [*RLP7*](http://db.yeastgenome.org/cgi-bin/locus.pl?locus=RLP7)*,* [*RMT2*](http://db.yeastgenome.org/cgi-bin/locus.pl?locus=RMT2)*,* [*RNH201*](http://db.yeastgenome.org/cgi-bin/locus.pl?locus=RNH201)*,* [*RNT1*](http://db.yeastgenome.org/cgi-bin/locus.pl?locus=RNT1)*,* [*ROK1*](http://db.yeastgenome.org/cgi-bin/locus.pl?locus=ROK1)*,* [*ROX3*](http://db.yeastgenome.org/cgi-bin/locus.pl?locus=ROX3)*,* [*RPA12*](http://db.yeastgenome.org/cgi-bin/locus.pl?locus=RPA12)*,* [*RPA190*](http://db.yeastgenome.org/cgi-bin/locus.pl?locus=RPA190)*,* [*RPA34*](http://db.yeastgenome.org/cgi-bin/locus.pl?locus=RPA34)*,* [*RPA43*](http://db.yeastgenome.org/cgi-bin/locus.pl?locus=RPA43)*,* [*RPA49*](http://db.yeastgenome.org/cgi-bin/locus.pl?locus=RPA49)*,* [*RPB10*](http://db.yeastgenome.org/cgi-bin/locus.pl?locus=RPB10)*,* [*RPB5*](http://db.yeastgenome.org/cgi-bin/locus.pl?locus=RPB5)*,* [*RPB8*](http://db.yeastgenome.org/cgi-bin/locus.pl?locus=RPB8)*,* [*RPB9*](http://db.yeastgenome.org/cgi-bin/locus.pl?locus=RPB9)*,* [*RPC11*](http://db.yeastgenome.org/cgi-bin/locus.pl?locus=RPC11)*,* [*RPC19*](http://db.yeastgenome.org/cgi-bin/locus.pl?locus=RPC19)*,* [*RPC31*](http://db.yeastgenome.org/cgi-bin/locus.pl?locus=RPC31)*,* [*RPC34*](http://db.yeastgenome.org/cgi-bin/locus.pl?locus=RPC34)*,* [*RPC37*](http://db.yeastgenome.org/cgi-bin/locus.pl?locus=RPC37)*,* [*RPC40*](http://db.yeastgenome.org/cgi-bin/locus.pl?locus=RPC40)*,* [*RPC53*](http://db.yeastgenome.org/cgi-bin/locus.pl?locus=RPC53)*,* [*RPC82*](http://db.yeastgenome.org/cgi-bin/locus.pl?locus=RPC82)*,* [*RPF1*](http://db.yeastgenome.org/cgi-bin/locus.pl?locus=RPF1)*,* [*RPF2*](http://db.yeastgenome.org/cgi-bin/locus.pl?locus=RPF2)*,* [*RPL13A*](http://db.yeastgenome.org/cgi-bin/locus.pl?locus=RPL13A)*,* [*RPL14A*](http://db.yeastgenome.org/cgi-bin/locus.pl?locus=RPL14A)*,* [*RPL14B*](http://db.yeastgenome.org/cgi-bin/locus.pl?locus=RPL14B)*,* [*RPL18B*](http://db.yeastgenome.org/cgi-bin/locus.pl?locus=RPL18B)*,* [*RPL20A*](http://db.yeastgenome.org/cgi-bin/locus.pl?locus=RPL20A)*,* [*RPL21B*](http://db.yeastgenome.org/cgi-bin/locus.pl?locus=RPL21B)*,* [*RPL26A*](http://db.yeastgenome.org/cgi-bin/locus.pl?locus=RPL26A)*,* [*RPL31B*](http://db.yeastgenome.org/cgi-bin/locus.pl?locus=RPL31B)*,* [*RPL33B*](http://db.yeastgenome.org/cgi-bin/locus.pl?locus=RPL33B)*,* [*RPL36A*](http://db.yeastgenome.org/cgi-bin/locus.pl?locus=RPL36A)*,* [*RPL43B*](http://db.yeastgenome.org/cgi-bin/locus.pl?locus=RPL43B)*,* [*RPL7B*](http://db.yeastgenome.org/cgi-bin/locus.pl?locus=RPL7B)*,* [*RPO26*](http://db.yeastgenome.org/cgi-bin/locus.pl?locus=RPO26)*,* [*RPP1*](http://db.yeastgenome.org/cgi-bin/locus.pl?locus=RPP1)*,* [*RPS10B*](http://db.yeastgenome.org/cgi-bin/locus.pl?locus=RPS10B)*,* [*RPS14B*](http://db.yeastgenome.org/cgi-bin/locus.pl?locus=RPS14B)*,* [*RPS19B*](http://db.yeastgenome.org/cgi-bin/locus.pl?locus=RPS19B)*,* [*RPS1A*](http://db.yeastgenome.org/cgi-bin/locus.pl?locus=RPS1A)*,* [*RPS21A*](http://db.yeastgenome.org/cgi-bin/locus.pl?locus=RPS21A)*,* [*RPS21B*](http://db.yeastgenome.org/cgi-bin/locus.pl?locus=RPS21B)*,* [*RPS26B*](http://db.yeastgenome.org/cgi-bin/locus.pl?locus=RPS26B)*,* [*RPS27A*](http://db.yeastgenome.org/cgi-bin/locus.pl?locus=RPS27A)*,* [*RPS29A*](http://db.yeastgenome.org/cgi-bin/locus.pl?locus=RPS29A)*,* [*RPS7A*](http://db.yeastgenome.org/cgi-bin/locus.pl?locus=RPS7A)*,* [*RPS7B*](http://db.yeastgenome.org/cgi-bin/locus.pl?locus=RPS7B)*,* [*RRM3*](http://db.yeastgenome.org/cgi-bin/locus.pl?locus=RRM3)*,* [*RRN11*](http://db.yeastgenome.org/cgi-bin/locus.pl?locus=RRN11)*,* [*RRN7*](http://db.yeastgenome.org/cgi-bin/locus.pl?locus=RRN7)*,* [*RRP1*](http://db.yeastgenome.org/cgi-bin/locus.pl?locus=RRP1)*,* [*RRP12*](http://db.yeastgenome.org/cgi-bin/locus.pl?locus=RRP12)*,* [*RRP15*](http://db.yeastgenome.org/cgi-bin/locus.pl?locus=RRP15)*,* [*RRP17*](http://db.yeastgenome.org/cgi-bin/locus.pl?locus=RRP17)*,* [*RRP3*](http://db.yeastgenome.org/cgi-bin/locus.pl?locus=RRP3)*, RRP36,* [*RRP40*](http://db.yeastgenome.org/cgi-bin/locus.pl?locus=RRP40)*,* [*RRP43*](http://db.yeastgenome.org/cgi-bin/locus.pl?locus=RRP43)*,* [*RRP5*](http://db.yeastgenome.org/cgi-bin/locus.pl?locus=RRP5)*,* [*RRP6*](http://db.yeastgenome.org/cgi-bin/locus.pl?locus=RRP6)*,* [*RRP8*](http://db.yeastgenome.org/cgi-bin/locus.pl?locus=RRP8)*,* [*RRP9*](http://db.yeastgenome.org/cgi-bin/locus.pl?locus=RRP9)*,* [*RRS1*](http://db.yeastgenome.org/cgi-bin/locus.pl?locus=RRS1)*,* [*RSA1*](http://db.yeastgenome.org/cgi-bin/locus.pl?locus=RSA1)*,* [*RSA3*](http://db.yeastgenome.org/cgi-bin/locus.pl?locus=RSA3)*,* [*RSA4*](http://db.yeastgenome.org/cgi-bin/locus.pl?locus=RSA4)*,* [*RVB1*](http://db.yeastgenome.org/cgi-bin/locus.pl?locus=RVB1)*,* [*SAN1*](http://db.yeastgenome.org/cgi-bin/locus.pl?locus=SAN1)*,* [*SAS10*](http://db.yeastgenome.org/cgi-bin/locus.pl?locus=SAS10)*,* [*SCC2*](http://db.yeastgenome.org/cgi-bin/locus.pl?locus=SCC2)*,* [*SCH9*](http://db.yeastgenome.org/cgi-bin/locus.pl?locus=SCH9)*,* [*SDA1*](http://db.yeastgenome.org/cgi-bin/locus.pl?locus=SDA1)*,* [*SEN34*](http://db.yeastgenome.org/cgi-bin/locus.pl?locus=SEN34)*,* [*SFG1*](http://db.yeastgenome.org/cgi-bin/locus.pl?locus=SFG1)*,* [*SHQ1*](http://db.yeastgenome.org/cgi-bin/locus.pl?locus=SHQ1)*,* [*SIK1*](http://db.yeastgenome.org/cgi-bin/locus.pl?locus=SIK1)*,* [*SIR2*](http://db.yeastgenome.org/cgi-bin/locus.pl?locus=SIR2)*,* [*SLI15*](http://db.yeastgenome.org/cgi-bin/locus.pl?locus=SLI15)*,* [*SLN1*](http://db.yeastgenome.org/cgi-bin/locus.pl?locus=SLN1)*,* [*SLX9*](http://db.yeastgenome.org/cgi-bin/locus.pl?locus=SLX9)*,* [*SMI1*](http://db.yeastgenome.org/cgi-bin/locus.pl?locus=SMI1)*,* [*SNU13*](http://db.yeastgenome.org/cgi-bin/locus.pl?locus=SNU13)*,* [*SOF1*](http://db.yeastgenome.org/cgi-bin/locus.pl?locus=SOF1)*,* [*SPB1*](http://db.yeastgenome.org/cgi-bin/locus.pl?locus=SPB1)*,* [*SPB4*](http://db.yeastgenome.org/cgi-bin/locus.pl?locus=SPB4)*,* [*SQT1*](http://db.yeastgenome.org/cgi-bin/locus.pl?locus=SQT1)*,* [*SRO9*](http://db.yeastgenome.org/cgi-bin/locus.pl?locus=SRO9)*,* [*SSF1*](http://db.yeastgenome.org/cgi-bin/locus.pl?locus=SSF1)*,* [*SSF2*](http://db.yeastgenome.org/cgi-bin/locus.pl?locus=SSF2)*,* [*STE12*](http://db.yeastgenome.org/cgi-bin/locus.pl?locus=STE12)*,* [*SUT1*](http://db.yeastgenome.org/cgi-bin/locus.pl?locus=SUT1)*,* [*SUT2*](http://db.yeastgenome.org/cgi-bin/locus.pl?locus=SUT2)*,* [*SUV3*](http://db.yeastgenome.org/cgi-bin/locus.pl?locus=SUV3)*,* [*SWI5*](http://db.yeastgenome.org/cgi-bin/locus.pl?locus=SWI5)*,* [*TAD3*](http://db.yeastgenome.org/cgi-bin/locus.pl?locus=TAD3)*,* [*THP2*](http://db.yeastgenome.org/cgi-bin/locus.pl?locus=THP2)*,* [*TIF2*](http://db.yeastgenome.org/cgi-bin/locus.pl?locus=TIF2)*,* [*TIF3*](http://db.yeastgenome.org/cgi-bin/locus.pl?locus=TIF3)*,* [*TIF35*](http://db.yeastgenome.org/cgi-bin/locus.pl?locus=TIF35)*,* [*TIF4631*](http://db.yeastgenome.org/cgi-bin/locus.pl?locus=TIF4631)*,* [*TOP1*](http://db.yeastgenome.org/cgi-bin/locus.pl?locus=TOP1)*,* [*TOS4*](http://db.yeastgenome.org/cgi-bin/locus.pl?locus=TOS4)*,* [*TPA1*](http://db.yeastgenome.org/cgi-bin/locus.pl?locus=TPA1)*,* [*TRF5*](http://db.yeastgenome.org/cgi-bin/locus.pl?locus=TRF5)*,* [*TRM1*](http://db.yeastgenome.org/cgi-bin/locus.pl?locus=TRM1)*,* [*TRM10*](http://db.yeastgenome.org/cgi-bin/locus.pl?locus=TRM10)*,* [*TRM11*](http://db.yeastgenome.org/cgi-bin/locus.pl?locus=TRM11)*,* [*TRM112*](http://db.yeastgenome.org/cgi-bin/locus.pl?locus=TRM112)*,* [*TRM13*](http://db.yeastgenome.org/cgi-bin/locus.pl?locus=TRM13)*,* [*TRM2*](http://db.yeastgenome.org/cgi-bin/locus.pl?locus=TRM2)*,* [*TRM3*](http://db.yeastgenome.org/cgi-bin/locus.pl?locus=TRM3)*,* [*TRM7*](http://db.yeastgenome.org/cgi-bin/locus.pl?locus=TRM7)*,* [*TRM8*](http://db.yeastgenome.org/cgi-bin/locus.pl?locus=TRM8)*,* [*TRM82*](http://db.yeastgenome.org/cgi-bin/locus.pl?locus=TRM82)*,* [*TRM9*](http://db.yeastgenome.org/cgi-bin/locus.pl?locus=TRM9)*,* [*TSR1*](http://db.yeastgenome.org/cgi-bin/locus.pl?locus=TSR1)*,* [*TSR2*](http://db.yeastgenome.org/cgi-bin/locus.pl?locus=TSR2)*,* [*TYW1*](http://db.yeastgenome.org/cgi-bin/locus.pl?locus=TYW1)*,* [*UBC9*](http://db.yeastgenome.org/cgi-bin/locus.pl?locus=UBC9)*,* [*UBP10*](http://db.yeastgenome.org/cgi-bin/locus.pl?locus=UBP10)*,* [*UNG1*](http://db.yeastgenome.org/cgi-bin/locus.pl?locus=UNG1)*,* [*URB1*](http://db.yeastgenome.org/cgi-bin/locus.pl?locus=URB1)*,* [*URB2*](http://db.yeastgenome.org/cgi-bin/locus.pl?locus=URB2)*,* [*UTP10*](http://db.yeastgenome.org/cgi-bin/locus.pl?locus=UTP10)*,* [*UTP11*](http://db.yeastgenome.org/cgi-bin/locus.pl?locus=UTP11)*,* [*UTP13*](http://db.yeastgenome.org/cgi-bin/locus.pl?locus=UTP13)*,* [*UTP14*](http://db.yeastgenome.org/cgi-bin/locus.pl?locus=UTP14)*,* [*UTP15*](http://db.yeastgenome.org/cgi-bin/locus.pl?locus=UTP15)*,* [*UTP18*](http://db.yeastgenome.org/cgi-bin/locus.pl?locus=UTP18)*,* [*UTP21*](http://db.yeastgenome.org/cgi-bin/locus.pl?locus=UTP21)*,* [*UTP22*](http://db.yeastgenome.org/cgi-bin/locus.pl?locus=UTP22)*,* [*UTP23*](http://db.yeastgenome.org/cgi-bin/locus.pl?locus=UTP23)*,* [*UTP30*](http://db.yeastgenome.org/cgi-bin/locus.pl?locus=UTP30)*,* [*UTP4*](http://db.yeastgenome.org/cgi-bin/locus.pl?locus=UTP4)*,* [*UTP5*](http://db.yeastgenome.org/cgi-bin/locus.pl?locus=UTP5)*,* [*UTP7*](http://db.yeastgenome.org/cgi-bin/locus.pl?locus=UTP7)*,* [*UTP8*](http://db.yeastgenome.org/cgi-bin/locus.pl?locus=UTP8)*,* [*UTP9*](http://db.yeastgenome.org/cgi-bin/locus.pl?locus=UTP9)*,* [*VRG4*](http://db.yeastgenome.org/cgi-bin/locus.pl?locus=VRG4)*,* [*WRS1*](http://db.yeastgenome.org/cgi-bin/locus.pl?locus=WRS1)*,* [*YEF3*](http://db.yeastgenome.org/cgi-bin/locus.pl?locus=YEF3)*,* [*YHR020W*](http://db.yeastgenome.org/cgi-bin/locus.pl?locus=YHR020W)*,* [*YNL247W*](http://db.yeastgenome.org/cgi-bin/locus.pl?locus=YNL247W)*,* [*YOX1*](http://db.yeastgenome.org/cgi-bin/locus.pl?locus=YOX1)*,* [*YRF1-6*](http://db.yeastgenome.org/cgi-bin/locus.pl?locus=YRF1-6)*,* [*YVH1*](http://db.yeastgenome.org/cgi-bin/locus.pl?locus=YVH1) |
| Protein-RNA complex assembly | 45 out of 558 genes, 8.1% | 1.56e-14 | [*BMS1*](http://db.yeastgenome.org/cgi-bin/locus.pl?locus=BMS1)*,* [*BRX1*](http://db.yeastgenome.org/cgi-bin/locus.pl?locus=BRX1)*,* [*CUS1*](http://db.yeastgenome.org/cgi-bin/locus.pl?locus=CUS1)*,* [*DBP10*](http://db.yeastgenome.org/cgi-bin/locus.pl?locus=DBP10)*,* [*DBP3*](http://db.yeastgenome.org/cgi-bin/locus.pl?locus=DBP3)*,* [*DBP6*](http://db.yeastgenome.org/cgi-bin/locus.pl?locus=DBP6)*,* [*DBP7*](http://db.yeastgenome.org/cgi-bin/locus.pl?locus=DBP7)*,* [*DBP9*](http://db.yeastgenome.org/cgi-bin/locus.pl?locus=DBP9)*,* [*DRS1*](http://db.yeastgenome.org/cgi-bin/locus.pl?locus=DRS1)*,* [*GCD10*](http://db.yeastgenome.org/cgi-bin/locus.pl?locus=GCD10)*,* [*GCD11*](http://db.yeastgenome.org/cgi-bin/locus.pl?locus=GCD11)*,* [*GCD2*](http://db.yeastgenome.org/cgi-bin/locus.pl?locus=GCD2)*,* [*GCN3*](http://db.yeastgenome.org/cgi-bin/locus.pl?locus=GCN3)*,* [*IPI1*](http://db.yeastgenome.org/cgi-bin/locus.pl?locus=IPI1)*,* [*IPI3*](http://db.yeastgenome.org/cgi-bin/locus.pl?locus=IPI3)*,* [*MAK11*](http://db.yeastgenome.org/cgi-bin/locus.pl?locus=MAK11)*,* [*MAK21*](http://db.yeastgenome.org/cgi-bin/locus.pl?locus=MAK21)*,* [*MIS1*](http://db.yeastgenome.org/cgi-bin/locus.pl?locus=MIS1)*,* [*NIP7*](http://db.yeastgenome.org/cgi-bin/locus.pl?locus=NIP7)*,* [*NMD3*](http://db.yeastgenome.org/cgi-bin/locus.pl?locus=NMD3)*,* [*NOC2*](http://db.yeastgenome.org/cgi-bin/locus.pl?locus=NOC2)*,* [*NOG2*](http://db.yeastgenome.org/cgi-bin/locus.pl?locus=NOG2)*,* [*NOP15*](http://db.yeastgenome.org/cgi-bin/locus.pl?locus=NOP15)*,* [*NOP8*](http://db.yeastgenome.org/cgi-bin/locus.pl?locus=NOP8)*,* [*NSR1*](http://db.yeastgenome.org/cgi-bin/locus.pl?locus=NSR1)*,* [*PRP24*](http://db.yeastgenome.org/cgi-bin/locus.pl?locus=PRP24)*,* [*REX4*](http://db.yeastgenome.org/cgi-bin/locus.pl?locus=REX4)*,* [*RIX1*](http://db.yeastgenome.org/cgi-bin/locus.pl?locus=RIX1)*,* [*RLI1*](http://db.yeastgenome.org/cgi-bin/locus.pl?locus=RLI1)*,* [*RPF1*](http://db.yeastgenome.org/cgi-bin/locus.pl?locus=RPF1)*,* [*RPF2*](http://db.yeastgenome.org/cgi-bin/locus.pl?locus=RPF2)*,* [*RPS14B*](http://db.yeastgenome.org/cgi-bin/locus.pl?locus=RPS14B)*,* [*RRP40*](http://db.yeastgenome.org/cgi-bin/locus.pl?locus=RRP40)*,* [*RSA1*](http://db.yeastgenome.org/cgi-bin/locus.pl?locus=RSA1)*,* [*RSA3*](http://db.yeastgenome.org/cgi-bin/locus.pl?locus=RSA3)*,* [*RSA4*](http://db.yeastgenome.org/cgi-bin/locus.pl?locus=RSA4)*,* [*SDA1*](http://db.yeastgenome.org/cgi-bin/locus.pl?locus=SDA1)*,* [*SPB4*](http://db.yeastgenome.org/cgi-bin/locus.pl?locus=SPB4)*,* [*SQT1*](http://db.yeastgenome.org/cgi-bin/locus.pl?locus=SQT1)*,* [*SSF1*](http://db.yeastgenome.org/cgi-bin/locus.pl?locus=SSF1)*,* [*SSF2*](http://db.yeastgenome.org/cgi-bin/locus.pl?locus=SSF2)*,* [*TIF2*](http://db.yeastgenome.org/cgi-bin/locus.pl?locus=TIF2)*,* [*TIF3*](http://db.yeastgenome.org/cgi-bin/locus.pl?locus=TIF3)*,* [*TIF35*](http://db.yeastgenome.org/cgi-bin/locus.pl?locus=TIF35)*,* [*TIF4631*](http://db.yeastgenome.org/cgi-bin/locus.pl?locus=TIF4631) |
| Ribosomal large subunit assembly and maintenance | 24 out of 558 genes, 4.3% | 4.02e-14 | [*BRX1*](http://db.yeastgenome.org/cgi-bin/locus.pl?locus=BRX1)*,* [*DBP10*](http://db.yeastgenome.org/cgi-bin/locus.pl?locus=DBP10)*,* [*DBP3*](http://db.yeastgenome.org/cgi-bin/locus.pl?locus=DBP3)*,* [*DBP6*](http://db.yeastgenome.org/cgi-bin/locus.pl?locus=DBP6)*,* [*DBP7*](http://db.yeastgenome.org/cgi-bin/locus.pl?locus=DBP7)*,* [*DBP9*](http://db.yeastgenome.org/cgi-bin/locus.pl?locus=DBP9)*,* [*DRS1*](http://db.yeastgenome.org/cgi-bin/locus.pl?locus=DRS1)*,* [*IPI1*](http://db.yeastgenome.org/cgi-bin/locus.pl?locus=IPI1)*,* [*IPI3*](http://db.yeastgenome.org/cgi-bin/locus.pl?locus=IPI3)*,* [*MAK11*](http://db.yeastgenome.org/cgi-bin/locus.pl?locus=MAK11)*,* [*MAK21*](http://db.yeastgenome.org/cgi-bin/locus.pl?locus=MAK21)*,* [*NIP7*](http://db.yeastgenome.org/cgi-bin/locus.pl?locus=NIP7)*,* [*NMD3*](http://db.yeastgenome.org/cgi-bin/locus.pl?locus=NMD3)*,* [*NOP8*](http://db.yeastgenome.org/cgi-bin/locus.pl?locus=NOP8)*,* [*RIX1*](http://db.yeastgenome.org/cgi-bin/locus.pl?locus=RIX1)*,* [*RPF1*](http://db.yeastgenome.org/cgi-bin/locus.pl?locus=RPF1)*,* [*RPF2*](http://db.yeastgenome.org/cgi-bin/locus.pl?locus=RPF2)*,* [*RSA1*](http://db.yeastgenome.org/cgi-bin/locus.pl?locus=RSA1)*,* [*RSA3*](http://db.yeastgenome.org/cgi-bin/locus.pl?locus=RSA3)*,* [*RSA4*](http://db.yeastgenome.org/cgi-bin/locus.pl?locus=RSA4)*,* [*SPB4*](http://db.yeastgenome.org/cgi-bin/locus.pl?locus=SPB4)*,* [*SQT1*](http://db.yeastgenome.org/cgi-bin/locus.pl?locus=SQT1)*,* [*SSF1*](http://db.yeastgenome.org/cgi-bin/locus.pl?locus=SSF1)*,* [*SSF2*](http://db.yeastgenome.org/cgi-bin/locus.pl?locus=SSF2) |
| tRNA modification | 26 out of 558 genes, 4.7% | 1.97e-13 | [*DUS1*](http://db.yeastgenome.org/cgi-bin/locus.pl?locus=DUS1)*,* [*DUS3*](http://db.yeastgenome.org/cgi-bin/locus.pl?locus=DUS3)*,* [*DUS4*](http://db.yeastgenome.org/cgi-bin/locus.pl?locus=DUS4)*,* [*ELP2*](http://db.yeastgenome.org/cgi-bin/locus.pl?locus=ELP2)*,* [*ELP3*](http://db.yeastgenome.org/cgi-bin/locus.pl?locus=ELP3)*,* [*GCD10*](http://db.yeastgenome.org/cgi-bin/locus.pl?locus=GCD10)*,* [*GCD14*](http://db.yeastgenome.org/cgi-bin/locus.pl?locus=GCD14)*,* [*KTI12*](http://db.yeastgenome.org/cgi-bin/locus.pl?locus=KTI12)*,* [*MIS1*](http://db.yeastgenome.org/cgi-bin/locus.pl?locus=MIS1)*,* [*NCL1*](http://db.yeastgenome.org/cgi-bin/locus.pl?locus=NCL1)*,* [*PUS1*](http://db.yeastgenome.org/cgi-bin/locus.pl?locus=PUS1)*,* [*PUS4*](http://db.yeastgenome.org/cgi-bin/locus.pl?locus=PUS4)*,* [*PUS7*](http://db.yeastgenome.org/cgi-bin/locus.pl?locus=PUS7)*,* [*TAD3*](http://db.yeastgenome.org/cgi-bin/locus.pl?locus=TAD3)*,* [*TRM1*](http://db.yeastgenome.org/cgi-bin/locus.pl?locus=TRM1)*,* [*TRM10*](http://db.yeastgenome.org/cgi-bin/locus.pl?locus=TRM10)*,* [*TRM11*](http://db.yeastgenome.org/cgi-bin/locus.pl?locus=TRM11)*,* [*TRM112*](http://db.yeastgenome.org/cgi-bin/locus.pl?locus=TRM112)*,* [*TRM13*](http://db.yeastgenome.org/cgi-bin/locus.pl?locus=TRM13)*,* [*TRM2*](http://db.yeastgenome.org/cgi-bin/locus.pl?locus=TRM2)*,* [*TRM3*](http://db.yeastgenome.org/cgi-bin/locus.pl?locus=TRM3)*,* [*TRM7*](http://db.yeastgenome.org/cgi-bin/locus.pl?locus=TRM7)*,* [*TRM8*](http://db.yeastgenome.org/cgi-bin/locus.pl?locus=TRM8)*,* [*TRM82*](http://db.yeastgenome.org/cgi-bin/locus.pl?locus=TRM82)*,* [*TRM9*](http://db.yeastgenome.org/cgi-bin/locus.pl?locus=TRM9)*,* [*TYW1*](http://db.yeastgenome.org/cgi-bin/locus.pl?locus=TYW1) |
| Ribosomal subunit assembly | 26 out of 558 genes, 4.7% | 2.21e-12 | [*BRX1*](http://db.yeastgenome.org/cgi-bin/locus.pl?locus=BRX1)*,* [*DBP10*](http://db.yeastgenome.org/cgi-bin/locus.pl?locus=DBP10)*,* [*DBP3*](http://db.yeastgenome.org/cgi-bin/locus.pl?locus=DBP3)*,* [*DBP6*](http://db.yeastgenome.org/cgi-bin/locus.pl?locus=DBP6)*,* [*DBP7*](http://db.yeastgenome.org/cgi-bin/locus.pl?locus=DBP7)*,* [*DBP9*](http://db.yeastgenome.org/cgi-bin/locus.pl?locus=DBP9)*,* [*DRS1*](http://db.yeastgenome.org/cgi-bin/locus.pl?locus=DRS1)*,* [*IPI1*](http://db.yeastgenome.org/cgi-bin/locus.pl?locus=IPI1)*,* [*IPI3*](http://db.yeastgenome.org/cgi-bin/locus.pl?locus=IPI3)*,* [*MAK11*](http://db.yeastgenome.org/cgi-bin/locus.pl?locus=MAK11)*,* [*MAK21*](http://db.yeastgenome.org/cgi-bin/locus.pl?locus=MAK21)*,* [*NIP7*](http://db.yeastgenome.org/cgi-bin/locus.pl?locus=NIP7)*,* [*NMD3*](http://db.yeastgenome.org/cgi-bin/locus.pl?locus=NMD3)*,* [*NOP8*](http://db.yeastgenome.org/cgi-bin/locus.pl?locus=NOP8)*,* [*NSR1*](http://db.yeastgenome.org/cgi-bin/locus.pl?locus=NSR1)*,* [*RIX1*](http://db.yeastgenome.org/cgi-bin/locus.pl?locus=RIX1)*,* [*RPF1*](http://db.yeastgenome.org/cgi-bin/locus.pl?locus=RPF1)*,* [*RPF2*](http://db.yeastgenome.org/cgi-bin/locus.pl?locus=RPF2)*,* [*RPS14B*](http://db.yeastgenome.org/cgi-bin/locus.pl?locus=RPS14B)*,* [*RSA1*](http://db.yeastgenome.org/cgi-bin/locus.pl?locus=RSA1)*,* [*RSA3*](http://db.yeastgenome.org/cgi-bin/locus.pl?locus=RSA3)*,* [*RSA4*](http://db.yeastgenome.org/cgi-bin/locus.pl?locus=RSA4)*,* [*SPB4*](http://db.yeastgenome.org/cgi-bin/locus.pl?locus=SPB4)*,* [*SQT1*](http://db.yeastgenome.org/cgi-bin/locus.pl?locus=SQT1)*,* [*SSF1*](http://db.yeastgenome.org/cgi-bin/locus.pl?locus=SSF1)*,* [*SSF2*](http://db.yeastgenome.org/cgi-bin/locus.pl?locus=SSF2) |
| tRNA metabolic process | 37 out of 558 genes, 6.6% | 3.21e-11 | [*AIR1*](http://db.yeastgenome.org/cgi-bin/locus.pl?locus=AIR1)*,* [*DUS1*](http://db.yeastgenome.org/cgi-bin/locus.pl?locus=DUS1)*,* [*DUS3*](http://db.yeastgenome.org/cgi-bin/locus.pl?locus=DUS3)*,* [*DUS4*](http://db.yeastgenome.org/cgi-bin/locus.pl?locus=DUS4)*,* [*ELP2*](http://db.yeastgenome.org/cgi-bin/locus.pl?locus=ELP2)*,* [*ELP3*](http://db.yeastgenome.org/cgi-bin/locus.pl?locus=ELP3)*,* [*GCD10*](http://db.yeastgenome.org/cgi-bin/locus.pl?locus=GCD10)*,* [*GCD14*](http://db.yeastgenome.org/cgi-bin/locus.pl?locus=GCD14)*,* [*GLN4*](http://db.yeastgenome.org/cgi-bin/locus.pl?locus=GLN4)*,* [*KTI12*](http://db.yeastgenome.org/cgi-bin/locus.pl?locus=KTI12)*,* [*MES1*](http://db.yeastgenome.org/cgi-bin/locus.pl?locus=MES1)*,* [*MIS1*](http://db.yeastgenome.org/cgi-bin/locus.pl?locus=MIS1)*,* [*NCL1*](http://db.yeastgenome.org/cgi-bin/locus.pl?locus=NCL1)*,* [*POP3*](http://db.yeastgenome.org/cgi-bin/locus.pl?locus=POP3)*,* [*POP6*](http://db.yeastgenome.org/cgi-bin/locus.pl?locus=POP6)*,* [*POP8*](http://db.yeastgenome.org/cgi-bin/locus.pl?locus=POP8)*,* [*PUS1*](http://db.yeastgenome.org/cgi-bin/locus.pl?locus=PUS1)*,* [*PUS4*](http://db.yeastgenome.org/cgi-bin/locus.pl?locus=PUS4)*,* [*PUS7*](http://db.yeastgenome.org/cgi-bin/locus.pl?locus=PUS7)*,* [*RPP1*](http://db.yeastgenome.org/cgi-bin/locus.pl?locus=RPP1)*,* [*SEN34*](http://db.yeastgenome.org/cgi-bin/locus.pl?locus=SEN34)*,* [*TAD3*](http://db.yeastgenome.org/cgi-bin/locus.pl?locus=TAD3)*,* [*TRM1*](http://db.yeastgenome.org/cgi-bin/locus.pl?locus=TRM1)*,* [*TRM10*](http://db.yeastgenome.org/cgi-bin/locus.pl?locus=TRM10)*,* [*TRM11*](http://db.yeastgenome.org/cgi-bin/locus.pl?locus=TRM11)*,* [*TRM112*](http://db.yeastgenome.org/cgi-bin/locus.pl?locus=TRM112)*,* [*TRM13*](http://db.yeastgenome.org/cgi-bin/locus.pl?locus=TRM13)*,* [*TRM2*](http://db.yeastgenome.org/cgi-bin/locus.pl?locus=TRM2)*,* [*TRM3*](http://db.yeastgenome.org/cgi-bin/locus.pl?locus=TRM3)*,* [*TRM7*](http://db.yeastgenome.org/cgi-bin/locus.pl?locus=TRM7)*,* [*TRM8*](http://db.yeastgenome.org/cgi-bin/locus.pl?locus=TRM8)*,* [*TRM82*](http://db.yeastgenome.org/cgi-bin/locus.pl?locus=TRM82)*,* [*TRM9*](http://db.yeastgenome.org/cgi-bin/locus.pl?locus=TRM9)*,* [*TYW1*](http://db.yeastgenome.org/cgi-bin/locus.pl?locus=TYW1)*,* [*WRS1*](http://db.yeastgenome.org/cgi-bin/locus.pl?locus=WRS1)*,* [*YHR020W*](http://db.yeastgenome.org/cgi-bin/locus.pl?locus=YHR020W)*,* [*YNL247W*](http://db.yeastgenome.org/cgi-bin/locus.pl?locus=YNL247W) |
| tRNA methylation | 13 out of 558 genes, 0.2% | 2.21e-10 | [*GCD10*](http://db.yeastgenome.org/cgi-bin/locus.pl?locus=GCD10)*,* [*GCD14*](http://db.yeastgenome.org/cgi-bin/locus.pl?locus=GCD14)*,* [*NCL1*](http://db.yeastgenome.org/cgi-bin/locus.pl?locus=NCL1)*,* [*TRM1*](http://db.yeastgenome.org/cgi-bin/locus.pl?locus=TRM1)*,* [*TRM10*](http://db.yeastgenome.org/cgi-bin/locus.pl?locus=TRM10)*,* [*TRM11*](http://db.yeastgenome.org/cgi-bin/locus.pl?locus=TRM11)*,* [*TRM112*](http://db.yeastgenome.org/cgi-bin/locus.pl?locus=TRM112)*,* [*TRM13*](http://db.yeastgenome.org/cgi-bin/locus.pl?locus=TRM13)*,* [*TRM3*](http://db.yeastgenome.org/cgi-bin/locus.pl?locus=TRM3)*,* [*TRM7*](http://db.yeastgenome.org/cgi-bin/locus.pl?locus=TRM7)*,* [*TRM8*](http://db.yeastgenome.org/cgi-bin/locus.pl?locus=TRM8)*,* [*TRM82*](http://db.yeastgenome.org/cgi-bin/locus.pl?locus=TRM82)*,* [*TRM9*](http://db.yeastgenome.org/cgi-bin/locus.pl?locus=TRM9) |

1.	Gasch AP, Spellman PT, Kao CM, Carmel-Harel O, Eisen MB, Storz G, Botstein D & Brown PO (2000) Genomic expression programs in the response of yeast cells to environmental changes. *Mol Biol Cell* **11**, 4241-4257.

2.	Causton HC, Ren B, Koh SS, Harbison CT, Kanin E, Jennings EG, Lee TI, True HL, Lander ES & Young RA (2001) Remodeling of yeast genome expression in response to environmental changes. *Mol Biol Cell* **12**, 323-337.
